# Regioselective Glycosylation Strategies for the Synthesis of Group Ia and Ib *Streptococcus* Related Glycans Enable Elucidating Unique Conformations of the Capsular Polysaccharides

**DOI:** 10.1002/chem.201903527

**Published:** 2019-11-04

**Authors:** Linda Del Bino, Ilaria Calloni, Davide Oldrini, Maria Michelina Raso, Rossella Cuffaro, Ana Ardá, Jeroen D. C. Codée, Jesús Jiménez‐Barbero, Roberto Adamo

**Affiliations:** ^1^ GSK Via Fiorentina 1 53100 Siena Italy; ^2^ CIC bioGUNE Bizkaia Technology Park, Building 800 48160 Derio Spain; ^3^ Department of Bioorganic Synthesis Leiden University 2333 Leiden The Netherlands; ^4^ Basque Foundation for Science IKERBASQUE 8009 Bilbao Spain; ^5^ Department of Organic Chemistry II Faculty of Science and Technology University of the Basque Country 48940 Leioa Spain

**Keywords:** carbohydrates, conformation analysis, glycosylation, regioselectivity, therapeutics

## Abstract

Group B *Streptococcus* serotypes Ia and Ib capsular polysaccharides are key targets for vaccine development. In spite of their immunospecifity these polysaccharides share high structural similarity. Both are composed of the same monosaccharide residues and differ only in the connection of the Neu5Acα2‐3Gal side chain to the GlcNAc unit, which is a β1‐4 linkage in serotype Ia and a β1‐3 linkage in serotype Ib. The development of efficient regioselective routes for GlcNAcβ1‐3[Glcβ1‐4]Gal synthons is described, which give access to different group B *Streptococcus* (GBS) Ia and Ib repeating unit frameshifts. These glycans were used to probe the conformation and molecular dynamics of the two polysaccharides, highlighting the different presentation of the protruding Neu5Acα2‐3Gal moieties on the polysaccharide backbones and a higher flexibility of Ib polymer relative to Ia, which can impact epitope exposure.

## Graphical abstract

**Glycosylation**: A panel of GBS serotype Ia and Ib fragments was prepared by combined regioselective‐stereoselective approaches and used to unravel polysaccharide conformations responsible for immune specificity.

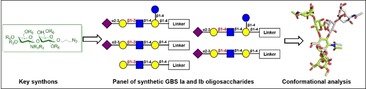

## Introduction

**Figure 1:**
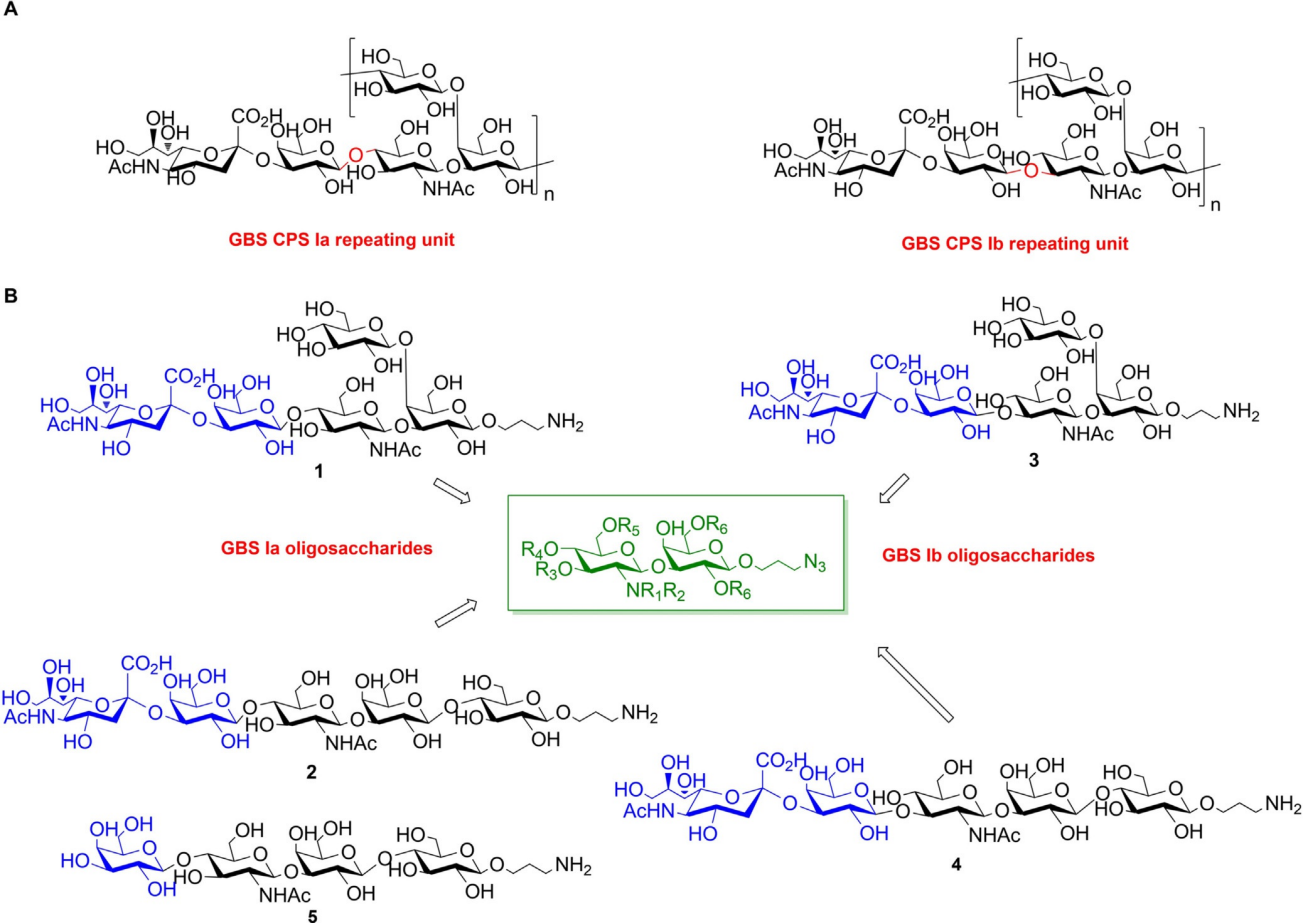
A) Chemical structure of GBS CPS Ia and Ib. B) Chemical structure of the target fragments from GBS CPS Ia (**1** and **2**) and CPS Ib (**3** and **4**). Synthesis of an unsialylated form of CPS Ia repeating units **5** was also envisaged.

Group *B Streptococcus* (GBS) is a leading cause of pneumonia, sepsis, meningitis, and death in neonates.^1^ It has also been associated with high rates of invasive diseases in the elderly.^1^ On the basis of variation in polysaccharide composition, the GBS sialic acid‐rich capsular polysaccharides (CPSs) are divided into ten serotypes (Ia, Ib, and II–IX).^2^ GBS CPSs are key virulence factors and considered the prime vaccine candidate to combat GBS infections.^3^ Monovalent conjugate vaccines prepared with GBS type‐specific polysaccharides representing the most frequent disease‐causing serotypes (Ia, Ib, II, III, and V), as well as a trivalent combination (Ia, Ib, III), have been tested in phase I/II clinical trials^4^ with the ultimate goal of developing a maternal vaccination strategy.^1^, ^5^ Multivalent formulations with six different serotypes are currently under clinical testing.^6^ GBS serotypes Ia, Ib, and III account for the majority of GBS related diseases.^7^ CPS Ia and Ib are structurally very similar. Both are composed of the same monosaccharide residues and differ only in the linkage between the Neu5Acα2‐3Gal side chain and the GlcNAc unit: a β1‐4 linkage in type Ia and a β1‐3 linkage in type Ib.^8^ This difference is critical in determining the immunospecificity (Figure 1).^3^, ^9^

The repeating units of CPS Ia and Ib can be described by the branched **1**, **3** and linear **2**, **4** frameshifts depicted in Figure 1. Intriguingly, the latter pentasaccharides **3** and **4** share identical monosaccharide composition with milk oligosaccharides, which have recently been proposed to inhibit GBS colonization.^10^ The availability of well‐defined GBS CPS glycans is key to explore interactions with serotype‐specific monoclonal antibodies in order to identify relevant glycoepitopes for elucidating the mechanism of action of the polysaccharide conjugates and for the development of synthetic carbohydrate‐based vaccines.^11^ The most studied of GBS polysaccharides is type III. This CPS is known to form a helical structure,^12^ and this feature has an impact on epitope exposure.^13^ Our group has recently synthesized CPSIII oligosaccharides that were used along with fragments obtained from CPS depolymerization to map a sialylated structural epitope spanning two repeating units.^14^ Considering that neither chemical nor enzymatic depolymerization reactions are available for CPS Ia and Ib, chemical synthesis is the only approach to obtain homogeneous oligosaccharides from the CPS.

Although the synthesis of the GBS CPS Ia repeating unit has been reported,^15^ the preparation of the pentasaccharide repeating unit of GBS CPS Ib has not been achieved. When approaching the synthesis of CPS Ia and Ib fragments, we envisaged the formation of the disaccharide GlcNAcβ1‐3Gal motif as a key step to enable convergent syntheses of a variety of structures depicted in Figure 1. Typically, installation of the GlcNAcβ1‐3Gal disaccharide within more complex glycans has been achieved with the 4‐hydroxyl group of the Gal acceptor either protected^16^, ^17^ or already engaged in a glycosidic linkage.^18^ Particularly, in the preparation of CPS Ia repeating units^15^ a 4,6‐*O*‐benzylidene‐protected Gal acceptor was used for glycosylation with a glucosamine trichloroacetimidate donor, and subsequent regioselective ring opening before further glycosylation of position 4 for the construction of the trisaccharide GlcNAcβ1‐3[Glcβ1‐4]Gal could take place. There is need of expeditious procedures for the construction of complex glycans, and regio‐ and stereoselective reactions distinguishing among diverse deprotected hydroxyls are highly desirable to simplify the oligosaccharide assembly.^19^ We reasoned that regioselective glycosylation of Gal 3‐OH would be the key for accelerating the synthesis of the GlcNAcβ1‐3Gal disaccharide and rendering the 4‐OH available for further glycosylation without the need of tedious protection/deprotection sequences.^20^

Herein, we report tactics to achieve regioselective syntheses of protected GlcNAcβ1‐3Gal building blocks and the use of these key synthons in convergent routes towards a series of fragments from CPS Ia and Ib repeating units with a built‐in aminopropyl linker amenable for future conjugation to carrier proteins (Figure 1). Furthermore, combination of NMR data from the synthetic GBS CPS Ia and Ib repeating units in their branched form **1** and **3**, respectively, and molecular dynamics simulation allowed to shed light on how the variation of a single sugar connection dramatically affects the conformational properties of CPS Ia and Ib polysaccharides, and hence exposition of potential epitopes for antibody recognition.

## Results and Discussion

### Optimization of regioselective glycosylation of galactose

According to our retrosynthetic design (Figure 1), the target glycans **1**–**4** can be obtained through a [2+3] convergent strategy based on the glycosylation of a suitable trisaccharide acceptor with a Neu5Acα2‐3Gal donor. This approach envisages the challenging stereoselective α sialylation of the upstream galactose at an early stage of the synthesis.^21^ Alternative use of a Gal donor would enable the synthesis of **5**. In this design, faster and efficient access to a GlcNAcβ1‐3Gal disaccharide building block plays a central role to obtain the trisaccharide acceptor without a temporary protection at position 4 for further assembly of GBS CPS Ia fragments. To achieve its regioselective synthesis, we investigated the effect of arming benzyl and disarming benzoyl groups^22^, ^23^ at position 2 and 6 of the Gal acceptors in tuning the reactivity of the 3‐ and 4‐OH, respectively, in combination with various protecting and leaving groups in the glucosamine donors. Despite the expected higher reactivity of the equatorial 3‐OH versus the axial 4‐OH, regioselective glycosylation of position 3 has been shown not to be trivial.^16^ Accordingly, we synthesized a series of glucosamine thioglycoside and trichloroacetimidate donors with the amine protected by the participating phthalimido (Phth) or trichloroethyl carbamate (Troc) group (experimental procedures are provided as Supporting Information).

**Scheme 1:**
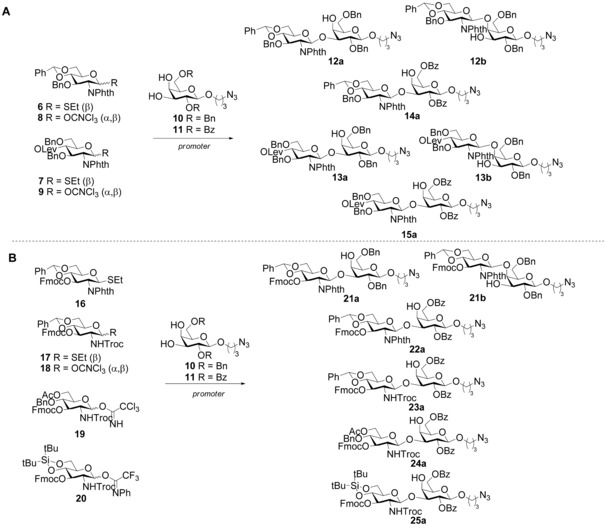
A) Preparation of disaccharide intermediates **12**–**15** for the synthesis of GBS CPS Ia repeating unit. Promoters and conditions are described in Table 1. B) Preparation of disaccharide intermediates **21**–**25** for the synthesis of GBS PSIb repeating unit. Promoters and conditions are described in Table 2.

Levulinoyl (Lev) and fluorenylmethyloxycarbonyl (Fmoc) were selected for temporary protection of either position 3 or 4. Alternatively, a 4,6‐*O*‐benzylidene was used to lock the 4‐ and 6‐hydroxyls to be subjected to regioselective ring opening delivering the 4‐OH at a later stage of the synthesis (Scheme 1). The prepared donors and acceptors were then coupled under several glycosylation conditions (Table 1 and Scheme 1) to optimize the synthesis of the GlcNAcβ1‐3Gal building block. The most efficient routes proved to be the combination of the 2,6‐di‐*O*‐benzoyl acceptor **11** with both donor **6** or **7** under *N*‐iodosuccinimide (NIS)/AgOTf‐mediated activation (Table 1, entries 7 and 8), which gave **14 a** and **15 a** in yields of 53 and 65 %, respectively, or the imidate **9** and acceptor **11** (Table 1, entry 9), which enabled the attainment of **14 a** in 77 % yield.

**Table 1:** Reaction of glucosamine donors **6**–**9** with Gal acceptors **10** and **11**.

Entry	Donor	Acceptor	Promoter, temperature [°C]	Yield [%]
1	**6**	**10**	NIS/TfOH, −30	n.d.^[a]^
2	**6**	**10**	NIS/Ag(OTf), 30	**12 a** (43)
				**12 b** (26)
3	**7**	**10**	NIS/Ag(OTf), −30	**13 a** (40)
				**13 b** ^[b]^ (28)
4	**8**	**10**	TMSOTf, −10	**12 a** (31)
5	**9**	**10**	TMSOTf, −10	**13 a** (45)
6	**6**	**11**	NIS/TfOH, −30	n.d.^[a]^
7	**6**	**11**	NIS/Ag(OTf), −30	**14 a** (53)
8	**7**	**11**	NIS/Ag(OTf), −30	**15 a** (65)
9	**8**	**11**	TMSOTf, −10	**14 a** (77)
10	**9**	**11**	TMSOTf, −10	**15 a** (33)

[a] CH_2_Cl_2_ was the solvent in all tested conditions; n.d.=not determined, product could not be detected; [b] The formation of the β1‐4 linkage was confirmed by acetylation of **13 b**. In the ^1^H NMR spectrum a shift from 3.32 to 4.69 ppm of the H‐3 signal of Gal, appearing as a doublet of doublets with *J*_2,3_=10.3 Hz and *J*_3,4_=2.5 Hz was observed, confirming occurrence of glycosylation at position 4.

Similarly, conditions for the preparation of a GlcNAcβ1‐3Gal synthon with a temporary group at its C3′‐OH, to allow the ensuing assembly of GBS CPSIb fragments, were explored (Table 2 and Scheme 1). The glycosylation of di‐*O*‐benzyl acceptor **10** with donor **16** by using NIS with either TfOH or AgOTf as co‐promoters gave variable mixtures of the β1‐3 **21 a** and β1‐4 **21 b** disaccharides (Table 2, entries 1 and 2). Again, the di‐*O*‐benzoyl acceptor **11** in the presence of NIS/AgOTf activation at −30 °C allowed achieving a yield of 68 % (Table 2, entry 4), which confirms the improved capacity of the benzoyl substituents to govern the regioselectivity of the reaction compared with benzyl substituents. These conditions were also efficient for the GlcNTroc donor **17**, which gave **23 a** in 65 % yield (Table 2, entry 6). When the trichloroacetimidate **18** was used, the yield was increased up to 70 % (Table 2, entry 7), which corroborates the potential of this type of donor for the regioselective control of the reaction. Finally, trifluoroacetimidate glucosamine **20** bearing a 4,6‐*O*‐silylidene protection in the presence of TMSOTf as promoter afforded the target disaccharide **25 a** in 62 % yield.^24^ The slighty higher flexibility or lower hindering effect of the silylidene relative to that of the benzylidene group favored the reaction. Overall, these results indicate that the regioselectivity of the glycosylation benefits from the decreased nucleophilicity of the axial 4‐hydroxyl, which is intrinsically less reactive than the 3‐hydroxyl group, induced by the electron‐withdrawing effect of the 2,6‐*O*‐benzoyl as compared with 2,6‐di‐*O*‐benzyl substituents in the Gal acceptor.

**Table 2:** Reaction of glucosamine donors **16**–**20** with Gal acceptors **10** and **11**.

Entry	Donor	Acceptor	Promoter, temperature [°C]	Yield [%]
1	**16**	**10**	NIS/TfOH, −30	**21 a** (30)
				**21 b** (<5)
2	**16**	**10**	NIS/AgOTf, −30	**21 a** (38)
				**21 b** (26)
3	**16**	**11**	NIS/TfOH, −30	**22 a** (40)
4	**16**	**11**	NIS/AgOTf, −30	**22 a** (68)
5	**17**	**11**	NIS/TfOH, −30	n.d.^[a]^
6	**17**	**11**	NIS/AgOTf, −30	**23 a** (65)
7	**18**	**11**	TMSOTf, −10	**23 a** (70)
8	**19**	**11**	TMSOTf, −10	**24 a** (50)
9	**20**	**11**	TMSOTf, −10	**25 a** (62)

[a] CH_2_Cl_2_ was the solvent in all tested conditions.

In addition, mild activation conditions (NIS/AgOTf) for the thioglycoside donor or the torsional disarming effect of the benzylidene/silylidene group for the imidate donors appears to favor the regioselectivity of glycosylation at position 3.

### Synthesis of GBS CPS Ia linear and branched repeating units

**Scheme 2:**
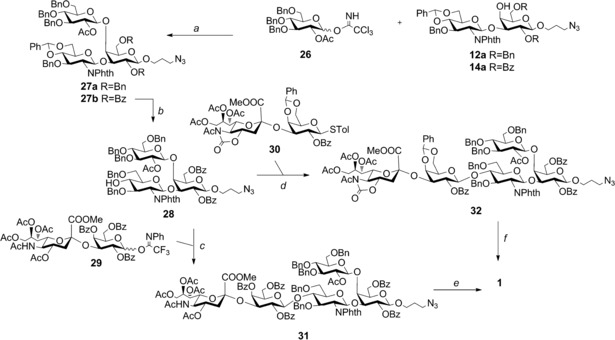
Assembly of GBS CPS Ia repeating unit. Reagents and conditions: a) TMSOTf, CH_2_Cl_2_ dry, −10 °C, (β‐) 75 % from **12 a**, 68 % (β‐) from **14 a**; b) Me_3_N**⋅**BH_3_, BF_3_ **⋅**Et_2_O, MeCN, 0 °C, 70 %; c) TMSOTf, CH_2_Cl_2_ dry, 0 °C, (β‐) 75 %; d) TfOH, NIS, CH_2_Cl_2_ dry, −40 °C, (β‐) 73 %; e) LiI, Py, 120 °C; H_2_NCH_2_CH_2_NH_2_, EtOH, 90 °C; Ac_2_O, Py; MeONa, MeOH; H_2_, Pd‐C, 40 % (over five steps); f) 3 m NaOH, THF, reflux; Ac_2_O, MeOH; H_2_, Pd‐C, 45 % (over three steps).

Having identified the two glycosylation partners giving the GlcNAcβ1‐3Gal motif in a regioselective fashion, we elongated the disaccharide building block to assemble the pentasaccharide repeating unit of GBS CPS Ia. To this end, reactions of glucose donor **26** 25 with disaccharide donors **12 a** and **14 a** were performed to furnish trisaccharides **27 a** and **27 b** in 75 and 68 % yield, respectively (Scheme 2). The newly formed glycosidic bond was in β configuration, as expected by the presence of a participating group.

Despite the deactivating effect of the 6‐*O*‐benzoyl ester relative to that of the 6‐*O*‐benzyl ether, the reaction proceeded with almost identical efficiency (Scheme 2), whereas a peracetylated trichloroacetimidate glucose donor with TMSOTf activation was ineffective for glycosylation of the 4‐OH. Considering the higher regioselectivity and yield achieved in synthesizing disaccharide **14 a**, the resulting trisaccharide **27 b** was advanced in the GBS CPS Ia repeating unit construction and subjected to regioselective opening of the 4,6‐*O*‐benzylidene acetal with BF_3_ **⋅**Et_2_O and Me_3_N**⋅**BH_3_ to provide the acceptor **28** (70 %).

In order to complete the pentasaccharide construction, the sialo‐galactosyl trifluoroacetimidate donor **29** 14a, 26 and thioglycoside **30** 27 were tested. Of these two disaccharides, **30** can be prepared with a higher α stereoselectivity, whereas **29** is easily accessible from a commercial disaccharide precursor.14a

Glycosylation of trisaccharide **28** with **29** under TMSOTf activation gave the protected pentasaccharide **31** in 75 % yield, and the use of disaccharide **30** in the presence of NIS/TfOH led to the protected pentasaccharide **32** in a similar yield (73 %). Compound **30** was deprotected by a four‐step procedure,^18^ including 1) saponification of the methyl ester of Neu5Ac with lithium iodide in pyridine; 2) reaction with ethylenediamine in ethanol heated to reflux for concomitant removal of the *O*‐acetates and the NPhth protecting group; 3) reacetylation with acetic anhydride/pyridine to install the acetamide group of the GlcNAc residue along with acetyl esters; 4) methanolysis and final catalytic hydrogenation over Pd/charcoal to provide the target branched pentasaccharide **1**.

Pentasaccharide **32** was first subjected to saponification with NaOH in THF heated to reflux, followed by amine reacetylation with a 2:3 acetic anhydride/methanol mixture.

Hydrogenation over Pd/charcoal afforded the target branched pentasaccharide **1** equipped with the aminopropyl linker suitable for conjugation. After purification by size exclusion chromatography, the final compound was obtained in 40 % overall yield from **31** and 45 % overall yield from **32**, respectively (Scheme 2).

**Scheme 3:**
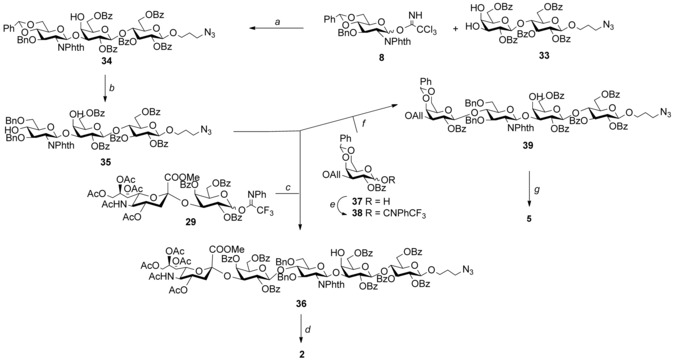
Assembly of linear GBS PS Ia fragments **2**. Reagents and conditions: a) TMSOTf, CH_2_Cl_2_ dry, −10 °C, (β‐) 68 %; b) Me_3_N**⋅**BH_3_, BF_3_ **⋅**Et_2_O, MeCN, 0 °C, 65 %; c) TMSOTf, CH_2_Cl_2_ dry, 0 °C, (β‐) 65 %; d) LiI, Py, 120 °C; H_2_NCH_2_CH_2_NH_2_, EtOH, 90 °C; Ac_2_O, Py; MeONa, MeOH; H_2_, Pd‐C, 33 % (over five steps); e) TFACl, Cs_2_CO_3_, CH_2_Cl_2_, 61 %; f) TMSOTf, −20 °C, CH_2_Cl_2_, (β‐) 72 %; g) PdCl_2_, MeOH; H_2_NCH_2_CH_2_NH_2_, EtOH, 90 °C; Ac_2_O, Py; MeONa, MeOH; H_2_, Pd‐C, 42 % (over five steps).

Next, we extended the same regioselective approach to the synthesis of the linear frameshift **2** of the serotype Ia repeating unit (Scheme 3). In this case, the benzoylated lactose **33** and the glucosamine donor **8** were chosen as glycosylation partners affording the linear trisaccharide acceptor **34** in 68 % yield with complete regioselectivity. Following benzylidene opening, the trisaccharide acceptor **35** was glycosylated with the two donors **29** and **30**. The first glycosylation promoted by TMSOTf at 0 °C afforded the target linear pentasaccharide **36** in 65 % yield, with β stereo‐ and regioselectivity at C‐4 of GlcNAc over the C‐4 of Gal. The presence of the free galactose 4‐OH throughout all stages of the synthesis, from trisaccharide **34** to pentasaccharide **36**, was monitored by following the signal of the Gal H‐4, which appeared at 3.97 ppm (d, *J=*2.7 Hz) in the ^1^H NMR and HSQC spectra of all synthetic intermediates. This confirmed the regioselectivity of the two glycosylations performed. Unexpectedly, reaction of **35** with the tolyl thioglycoside **30** under NIS/TfOH activation at −40 °C yielded only traces of the corresponding pentasaccharide, whereas mainly decomposition of the glycosyl donor was observed, as revealed by LC‐MS analysis. The linear pentasaccharide **36** was subjected to the five‐step deprotection protocol previously described for compound **31**. The target oligosaccharide **2** was purified by size exclusion chromatography and obtained in 33 % overall yield (Scheme 3). NMR data of the synthesized fragments were in excellent agreement with those of the CPS Ia samples.^8^

From acceptor **35** a desialylated CPS Ia linear fragment for future mapping studies was also obtained by glycosylation (72 % yield) with the trifluoroacetimidate **38**, prepared from the known 1‐OH compound **37**.^18^ After global deprotection tetrasaccharide **5** was obtained in 42 % yield (Scheme 3).

### Synthesis of GBS CPS Ib linear and branched repeating unit

Differently than the GBS CPS Ia pentasaccharides, the two Ib frameshifts **3** and **4** required a glucosamine building block bearing a temporary protecting group at its C3‐OH and the creation of the Galβ1‐3GlcNAc linkage, which had a strong impact on our synthetic design. Initial attempts to prepare the branched pentasaccharide **3** by using a NPhth‐protected trisaccharide acceptor, similarly as done for the CPS Ia branched unit, were unsuccessful (Supporting Information, Scheme S9).

**Scheme 4:**
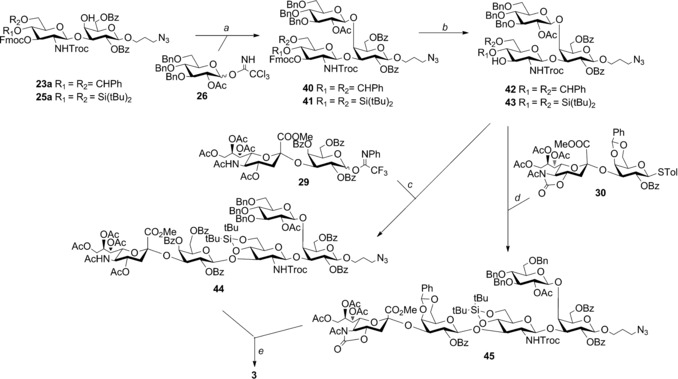
Assembly of GBS PSIb pentasaccharide branched unit **3**. Reagents and conditions: a) TMSOTf, CH_2_Cl_2_ dry, 0 °C, (β‐) 63 % for **41**, (β‐) 70 % or **42**; b) piperidine, CH_2_Cl_2_ dry, 92 %; c) TMSOTf, CH_2_Cl_2_ dry, 0 °C, (β‐) 80 %; d) TfOH, NIS, CH_2_Cl_2_ dry, −40 °C, (β‐) 65 %; e) HF/pyridine, 0 °C; 3 m NaOH, THF, reflux; Ac_2_O/MeOH; H_2_/Pd‐C, 40 %.

The C3‐OH of the glucosamine appeared significantly less reactive than the C4‐OH, which is likely due to the presence of the bulky NPhth group that could hinder the glycosylation reaction at the C3−OH. We anticipated that its replacement with a Troc protection would result in a higher nucleophilicity of the vicinal hydroxyl. Disaccharides **23 a** and **25 a**, which differ only in the cyclic protecting group blocking the glucosamine C4,6−OH groups, were selected to be elongated to the branched pentasaccharide **3** (Scheme 4). Glycosylation of the two acceptors with the armed Glc donor **26** under TMSOTf activation at 0 °C afforded the trisaccharides **40** and **41** in 63 and 70 % yield, respectively, as β anomers. After Fmoc removal by treatment with 10 % piperidine in CH_2_Cl_2_ (92 %), glycosylation with the sialogalactoside donor **29** of the two acceptors **42** and **43** was tested.

Reaction of the 4,6‐*O*‐benzylidene trisaccharide **42** and **29** with TMSOTf as a promoter failed to afford the target pentasaccharide, leading to complete recovery of the unreacted acceptor. In contrast, reaction of acceptor **43**, bearing the more flexible 4,6‐*O*‐silylidene ketal, with **29** in the presence of TMSOTf gave the target pentasaccharide **44** in 80 % yield (Scheme 4). This result suggests that the glycosylation of **42** was prevented by the steric and torsional constrain of the 4,6‐*O*‐benzylidene ring. Trisaccharide **43** was also efficiently β‐glycosylated with disaccharide donor **30** by NIS/TfOH activation, affording **45** in 65 % yield (Scheme 4). Despite a slightly lower yield in this step, the overall efficiency of the synthesis of the GBS serotype Ib branched repeating unit was superior when using the thioglycoside **30** with respect to the imidate **29** because of the better α stereoselectivity of the glycosylation leading to **30**.^26^, ^27^ Pentasaccharides **44** and **45** were then deprotected by a four‐step protocol (Scheme 4): 1) desilylation by treatment with HF**⋅**pyridine, 2) saponification with NaOH in THF heated to reflux, for concomitant hydrolysis of the acyl esters, the Troc group, and the 5‐*N*,4‐*O*‐oxazolidinone protecting group and Neu5Ac methyl ester, 3) reacetylation of the amines by a 2:3 acetic anhydride/methanol mixture, 4) hydrogenation over Pd/charcoal. The target branched pentasaccharide **3** was obtained in 40 % yield.

**Scheme 5:**
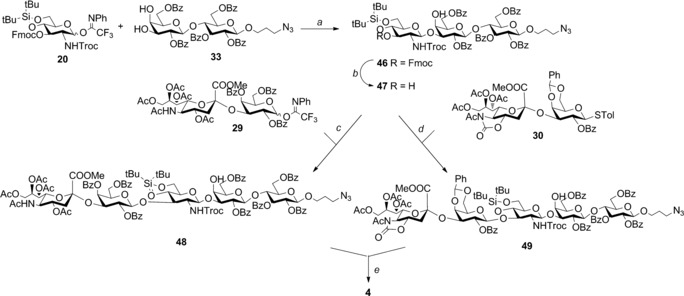
Synthetic route to type Ib linear repeating unit. Reagents and conditions: a) TMSOTf, CH_2_Cl_2_ dry, 0 °C, (β‐) 55 %; b) piperidine, CH_2_Cl_2_, 90 %; c) TMSOTf, CH_2_Cl_2_ dry, 0 °C, (β‐) 66 %; d) TfOH, NIS, CH_2_Cl_2_ dry, −40 °C, (β‐) 40 %; e) HF/pyridine; 3 m NaOH, THF, reflux; Ac_2_O/MeOH; H_2_/Pd‐C, 40 %.

Finally, we extended our regioselective approach to the synthesis of the linear frameshift **4** of the GBS serotype Ib repeating unit (Scheme 5). For this purpose, benzoylated lactose acceptor **33** was glycosylated with the 4,6‐*O*‐silylidene glucosamine imidate **20** under TMSOTf activation to give the target trisaccharide **46** with full β1‐3 stereo‐ and regioselectivity (55 %). Following Fmoc deprotection with piperidine in CH_2_Cl_2_, the obtained acceptor **47** was β‐glycosylated with imidate **29** to attain the linear protected pentasaccharide **48** (66 %). Reaction with thioglycoside **30** in TfOH and NIS reaction conditions also provided the analogous pentasaccharide **49** (40 %). The obtained pentasaccharides were deprotected and purified as described above. NMR spectroscopic data of the synthesized CPS Ib fragments were in excellent agreement with NMR spectroscopic data from samples of the bacterial polysaccharide.8a

### Conformational analysis

The conformational properties of the CPS Ia and Ib branched repeating unit pentasaccharides **1** and **3** were studied by a combination of NMR spectroscopy and modeling tools,^28^ and compared with those of the corresponding polysaccharides. Interglycosidic interproton distances for **1** and **3** were estimated from ROESY spectra. The obtained experimental distances were compared with those derived from a 200 ns molecular dynamics (MD) simulation. Table 3 gathers the results for the CPS Ia pentasaccharide **1**. The comparison reflects a good agreement between the NMR‐ and the MD‐derived distances for the glycosidic linkages GlcNAcβ1‐3Gal and Glcβ1‐4Gal (defined by the interproton distances H1GlcNAc‐H3Gal and H1Glc‐H4Gal, respectively). The *Φ*/*Ψ* population analysis from the MD simulation showed a single population for *Φ* fulfilling the *exo*‐anomeric effect (*exo*‐*syn*‐*Φ*),^29^, ^30^ and two populations around *ψ* for both linkages (Supporting Information, Figure S1).

**Table 3:** Interglycosidic interproton [Å] distances for the CPS Ia pentasaccharide (**1**).

	NMR	MD			
		total average	180/−30	−60/20	−60/−50
H3Gal‐H3eqNeuNAc	none	4.2	3.4	4.4	4.6
H3Gal‐H3axNeuNAc	2.7	3.6	2.1	4.3	4.0
H3Gal‐H8NeuNAc	very weak	3.9	4.8	3.7	3.4
H1Gal‐H4GlcNAc	2.4	2.4			
H1Gal‐H6GlcNAc	2.7	3.0			
H1Gal‐H6'GlcNAc	3.1	3.9			
H1Glc‐H4Gal	2.6	2.5			
H1GlcNAc‐H3Gal	2.5	2.4

For the Galβ1‐4GlcNAc linkage, there is a perfect agreement for the H1Gal‐H4GlcNAc distance with slight discrepancies for the H1Gal‐H6,H6′GlcNAc ones, which is probably due to the MD bias around the GlcNAc ω torsion angle (Supporting Information, Figure S2). These data support the existence of a single population around the *exo*‐*syn*‐*Φ*/syn*Ψ* conformation, as predicted by the MD simulation.

**Figure 2:**
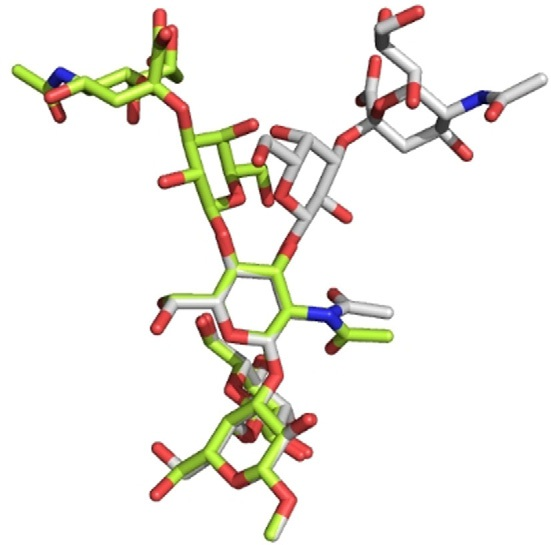
Superimposition of the major conformations for pentasaccharides **1** (lime) and **3** (grey), with the *exo*‐anti‐*Φ* geometry around the Neu5Acα2‐3Gal linkage.

For the Neu5Acα2‐3Gal linkage, MD simulations predict three different populations, 180°/−30°, −60°/−20°, and −60°/−50°. The interglycosidic interproton distances for each population are gathered in Table 3. There is a remarkable difference for the H3Gal‐H3axNeuNAc distance, being shorter according to NMR spectroscopy, which indicates that the MD simulation has a bias for the conformational ensemble towards *exo*‐*syn*‐*Φ* populations. Indeed, according the NOE‐derived distance, the *exo*‐*anti*‐*Φ* population should be the major one, representing around 75 % of the total ensemble. Two representative conformations for the CPS Ia pentasaccharide **1** are shown in the Supporting Information, differing in the Neu5Acα2‐3Gal linkage (Figure S3). The analysis for the CPS Ib pentasaccharide **3** yielded similar results (Supporting Information, Table S3), although the GlcNAcβ1‐3Gal linkage could not be fully characterized because of the overlapping between the H1GlcNAc and H3Gal protons. The linkage between Gal and GlcNAc, now β1‐3 instead of β1‐4, populates a minimum around the *exo*‐*syn*‐*Φ*/*syn(*−*)*‐*Ψ* conformation (Supporting Information, Figure S4). Two representative conformations for the CPS Ib pentasaccharide **3** are shown in the Supporting Information, which also differ in the orientation around the Neu5Acα2‐3Gal *Φ* torsion (Supporting Information, Figure S5). A superimposition of representative 3D structures for the CPS Ia and Ib pentasaccharides **1** and **3**, with the major conformation *exo*‐*anti*‐*Φ* around the Neu5Acα2‐3Gal linkage is shown in Figure 2. The conformational behavior of the polysaccharides was then analyzed following a similar protocol. A model for the polysaccharide was built with 10 repeating units (50 monosaccharides) and MD simulations were run for 2.5 μs.

**Figure 3:**
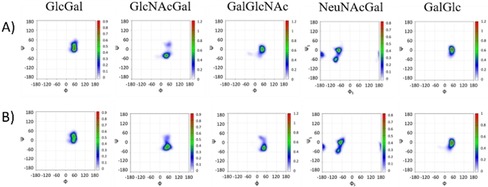
Glycosidic linkage analysis for GBS Ia (A) and Ib (B) polysaccharides: *Φ*/*Ψ* plots for representative glycosidic bonds of a 10 repeating unit model along the 2.5 μs MD simulation.

The analysis of the glycosidic linkages was carried out for the 49 glycosidic bonds, revealing that the behavior for every glycosidic bond type is reproducible along the polysaccharide (Figure 3).

**Figure 4:**
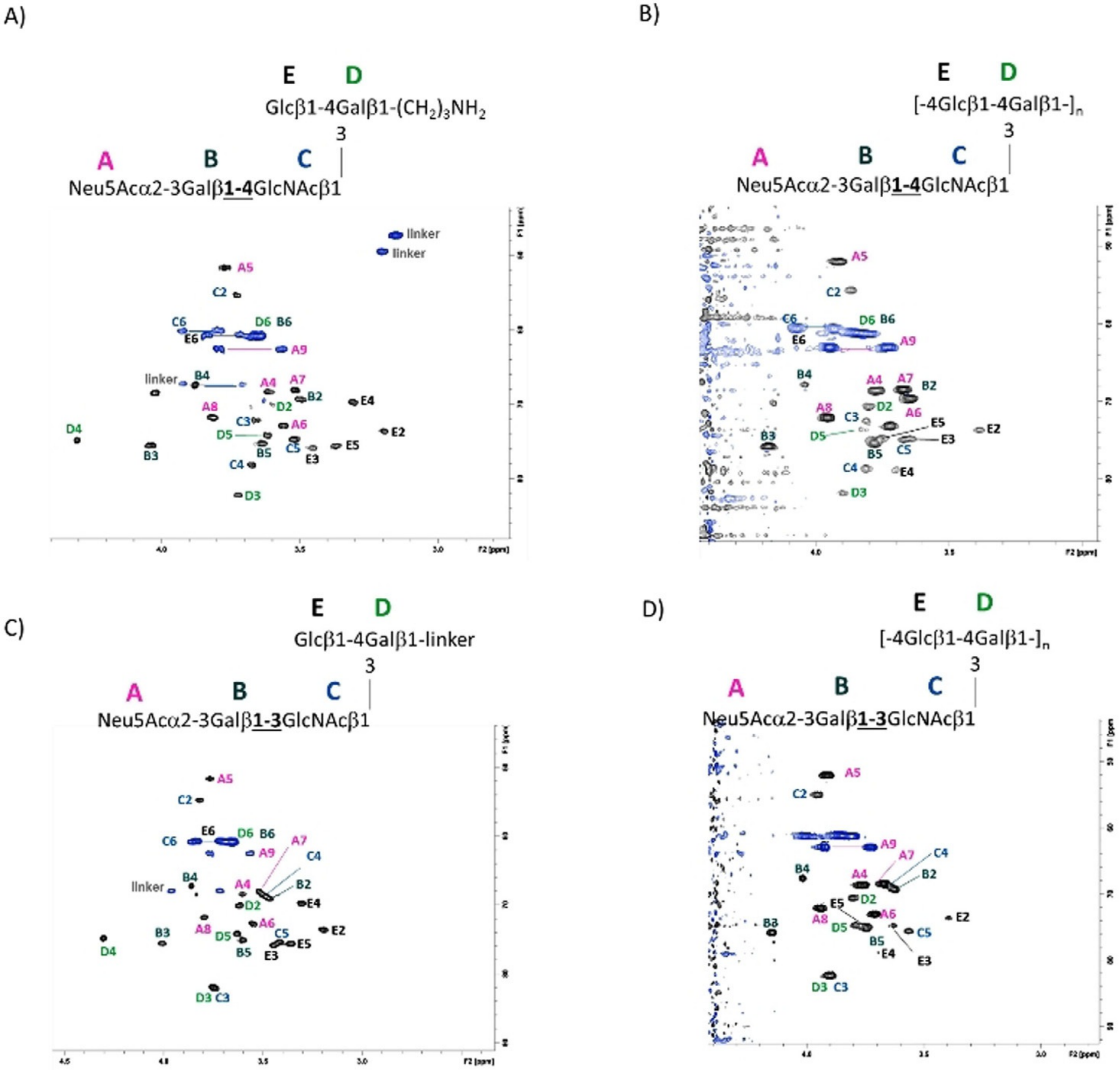
^1^H‐^13^C‐HSQC spectrum recorded for the pentasaccharide repeating unit of GBS Ia at 600 MHz, 298 K, D_2_O (A and B) and for GBS CPS Ib at 800 MHz, 318 K, D_2_O, showing the assignment of the ^1^H and ^13^C NMR signals. As expected, the matching is excellent except for some signals of residue E.

**Figure 5:**
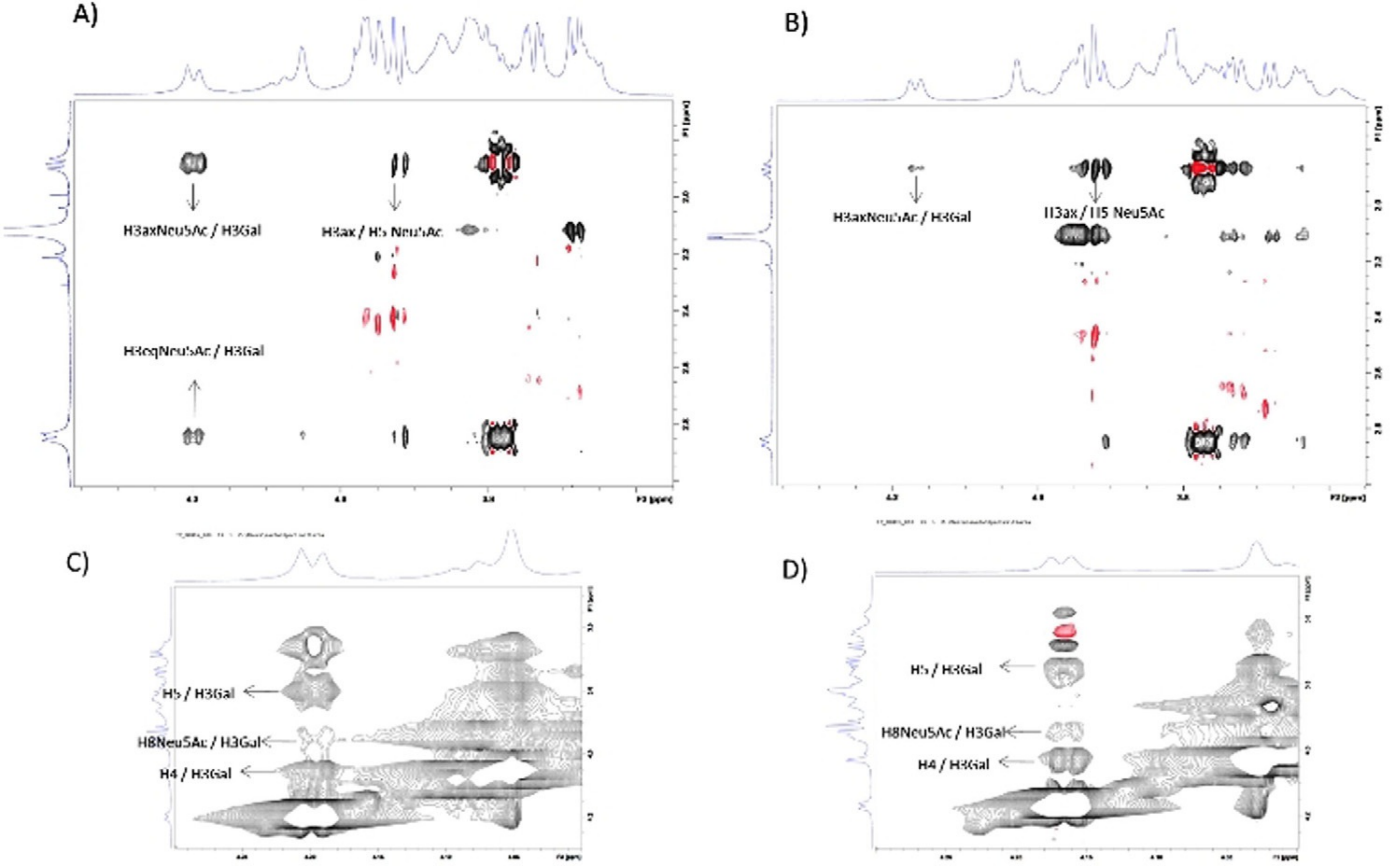
The key regions of the NOESY spectra recorded for the GBS CPS Ia (A and C) and GBS CPS Ib (B and D) polysaccharides showing the essential inter‐residue cross peaks.

**Figure 6:**
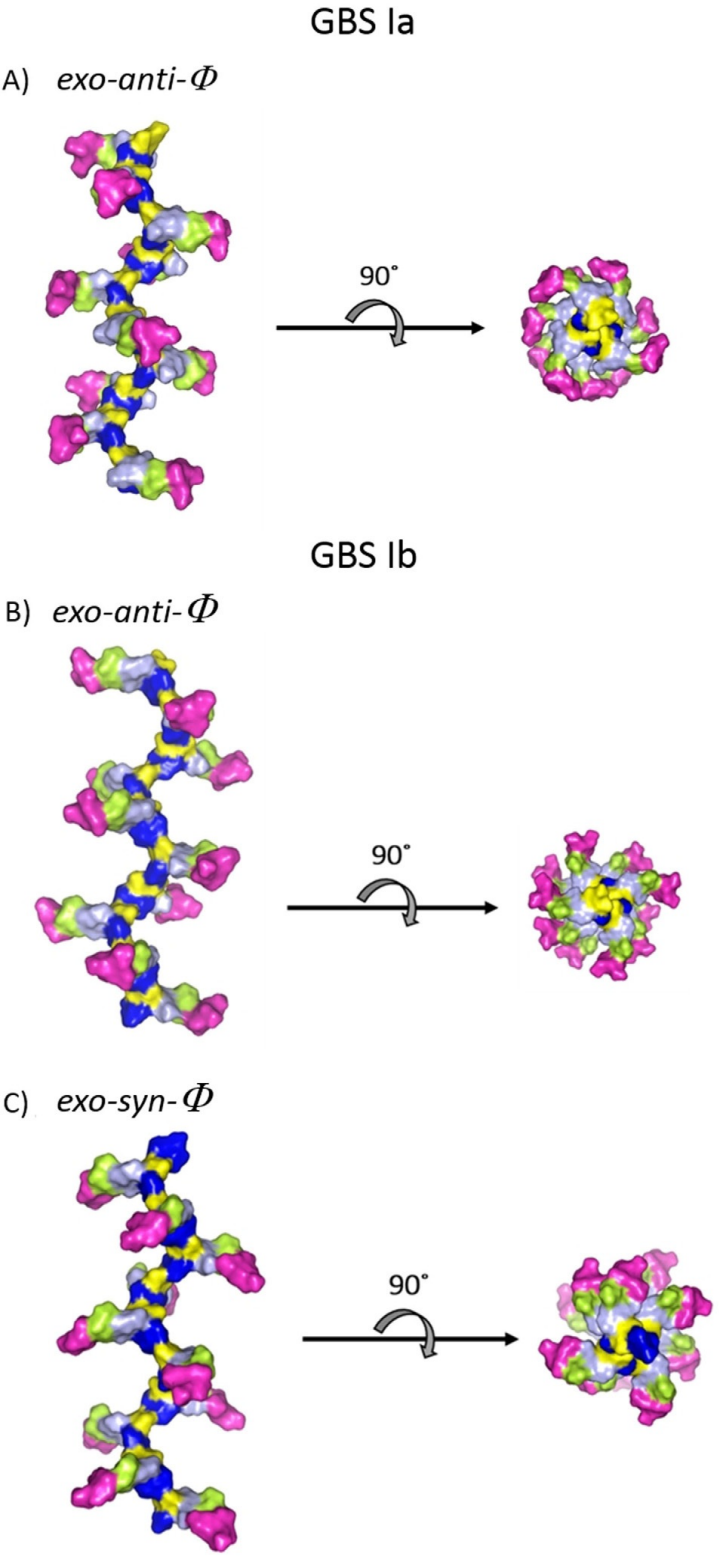
Model structures for the GBS Ia and Ib polysaccharides: A) Ia with the major *exo*‐*anti*‐*Φ* conformation around all the Neu5Acα2‐3Gal linkages B) Ib with *exo*‐*anti*‐*Φ* conformation around all Neu5Acα2‐3Gal linkages, and C) Ib with the major *exo*‐*syn*‐*Φ* conformation around all Neu5Acα2‐3Gal linkages.

These populations are comparable to those of the corresponding pentasaccharide for every glycosidic linkage, and thus, the resulting interglycosidic interproton distances are very similar (Supporting Information, Table S1‐2). Remarkably, for both GBS serotype Ia and Ib, the HSQC spectra of the polysaccharide and the pentasaccharide were very similar, with the obvious exception for the Glcβ1‐4 linked moiety (E), which is not glycosylated at O4 in the pentasaccharides (Figure 4). The analysis of the interglycosidic NOE (from NOESY spectra at 20 ms mixing time) was consistent with the MD‐derived populations. The only discrepancies arose again for the Neu5Acα2‐3Gal linkages. Interestingly, for the Ia polysaccharide the NOE‐derived distance for H3axNeu5Ac‐H3Gal is 2.4 Å, shorter than that in the pentasaccharide. At the same time, there is a clear NOE between H3eqNeu5Ac‐H3Gal, not observed for the pentasaccharide. On the contrary, for the Ib polysaccharide the distance H3axNeu5Ac‐H3Gal is longer, 3.3 Å, whereas the NOE between H3eqNeu5Ac‐H3Gal does not exist (Figure 5 A, B). At the same time, the distance H8Neu5Ac‐H3Gal is slightly shorter for the Ib than that for the Ia polysaccharide (Figure 5 C, D). These data suggest that for the Ia polysaccharide, the major conformation around the Neu5Acα2‐3Gal fragment is the *exo*‐*anti*‐*Φ* (ca. 85 %), whereas for the Ib polysaccharide, there is a larger flexibility, with a major *exo*‐*syn*‐*Φ* form (ca. 55 %). The model structures for the polysaccharides with all Neu5Acα2‐3Gal linkages in *exo*‐*anti*‐*Φ* (Ia) and *exo*‐*syn*‐*Φ* (Ib) are displayed in Figure 6, showing different preferential shapes for the two polysaccharides. The Neu5Acα2‐3Gal branches of GBS PSIII have been shown to be strongly engaged in antibody recognition.14b The favored presentation of the different epitopes for the major conformation is rather different. However, given their intrinsic flexibility, especially for Ib, both molecules could be accommodated to interact with the monoclonal binding pockets without a major entropy penalty.^31^

## Conclusions

To have fast access to homogeneous oligosaccharide antigens from GBS serotypes Ia and Ib and to gain insights into the conformational difference among these structurally similar polymers, we developed a highly convergent synthetic strategy based on the regioselective glycosylation of a galactose C3,4‐diol to obtain GlcNAcβ1‐3Gal disaccharide building blocks. Investigation of the different reactivities of the C3‐ and C4‐hydroxyls allowed us to reduce the number of protective group manipulations and synthetic steps to the final fragments, therefore simplifying the overall synthetic design.

Particularly, the use of a 2,6‐*O*‐benzoyl galactose diol resulted in improved regioselectively relative to that of the 2,6‐di‐*O*‐benzyl counterpart. In addition, mild activation conditions (NIS/AgOTf) for the glucosamine thiol donors or the torsional disarming effect of the benzylidene group for the trichloroacetimidate donors appear to favor the glycosylation reaction. The regioselective glucosamine incorporation was successfully applied to the synthesis of GBS CPS Ia and Ib branched repeating units (**1** and **2**). Their linear frameshifts (**3** and **4**) and a non‐sialylated CPS Ia form (**5**) were also synthesized to achieve an additional regioselective glycosylation of the Gal C3‐OH over the C4‐OH residue.

These results support the general applicability of the method to a variety of medically relevant glycans. Importantly, the structures synthesized through regioselective glycosylation appear extendible at the 4‐OH position of the Gal residue, thus potentially enabling the synthesis of longer and more complex GBS oligosaccharide structures.

Conformation analysis studies of the prepared oligosaccharides by NMR spectroscopy and MD simulations showed the impact of the GlcNAcβ1‐3Gal versus GlcNAcβ1‐4Gal connectivity in the orientation of the Neu5Acα2‐3Gal branching. The model, established from the single synthetic pentasaccharide repeating units, was used to study the conformational behavior of the GBS Ia and Ib polysaccharides, showing a different preferential shape for each polysaccharide with the Neu5Acα2‐3Gal linkages in *exo*‐*anti*‐*Φ* for Ia and *exo*‐*syn*‐*Φ* for Ib. These unique structural features are expected to influence antibody recognition and immunospecificity. Studies are ongoing to map the relevant glycoepitopes. Moreover, all glycans were designed with a chemical handle for conjugation to carrier proteins for immunological evaluation. Results on structural and immunogenic studies will be reported in due course.

## Conflict of interest

L.D.B., D.O., M.M.R., R.C., and R.A. are employees of GSK groups companies. L.D.B. and R.A. are inventors of a patent related to the topic. R.A. is owner of GSK stocks.

## Supporting information

As a service to our authors and readers, this journal provides supporting information supplied by the authors. Such materials are peer reviewed and may be re‐organized for online delivery, but are not copy‐edited or typeset. Technical support issues arising from supporting information (other than missing files) should be addressed to the authors.

Supplementary
