# Supplementary material for: Regioselective Glycosylation Strategies for the Synthesis of Group Ia and Ib Streptococcus Related Glycans Enable Elucidating Unique Conformations of the Capsular Polysaccharides
Source: Chemistry. 2019 Nov 4;25(71):16277–87. doi: 10.1002/chem.201903527 (PMC6972993; doi:10.1002/chem.201903527)
Supplement: Supplementary file 1 — Supplementary [file CHEM-25-16277-s001.pdf]

CHEMISTRY
A European Journal

Supporting Information

Regioselective Glycosylation Strategies for the Synthesis of Group Ia and Ib *Streptococcus* Related Glycans Enable Elucidating Unique Conformations of the Capsular Polysaccharides

Linda Del Bino,[a] Ilaria Calloni,[b] Davide Oldrini,[a] Maria Michelina Raso,[a] Rossella Cuffaro,[a] Ana Ardá,[b] Jeroen D. C. Codée,[c] Jesús Jiménez-Barbero,[b, d, e] and Roberto Adamo*[a]

chem_201903527_sm_miscellaneous_information.pdf

Author Contributions

Conceptualization, R.A., J.J.B., J.D.C.C., L.D.B., A.A., I.C.; Methodology, L.D.B., I.C., D.O., M.M.R. R.C.; Writing-Original Draft Preparation, R.A., J.J.B., J.D.C.C., L.D.B, A.A., I.C.; Writing-Review & Editing, all authors.

Table of contents

1. Synthetic schemes S2

2. Experimental S5

2.1 General Methods S5

2.2 Synthetic procedures S6

2.3 Synthesis of building blocks: S6

2.4 Preparations of disaccharides S18

2.5 Syntheses of trisaccharides S25

2.6 Synthesis of GBS PSIa repeating units **1** and **2** S34

2.7 Synthesis of tetrasaccharide **5** S38

2.8 Synthesis of GBS PSIb repeating units **3** and **4** S40

3. NMR Spectra of synthesized compounds S45

4. Conformational studies S100

5. References S106

1. Synthetic schemes

Syntheses of the trichloroacetimidate donors **7** and **8**

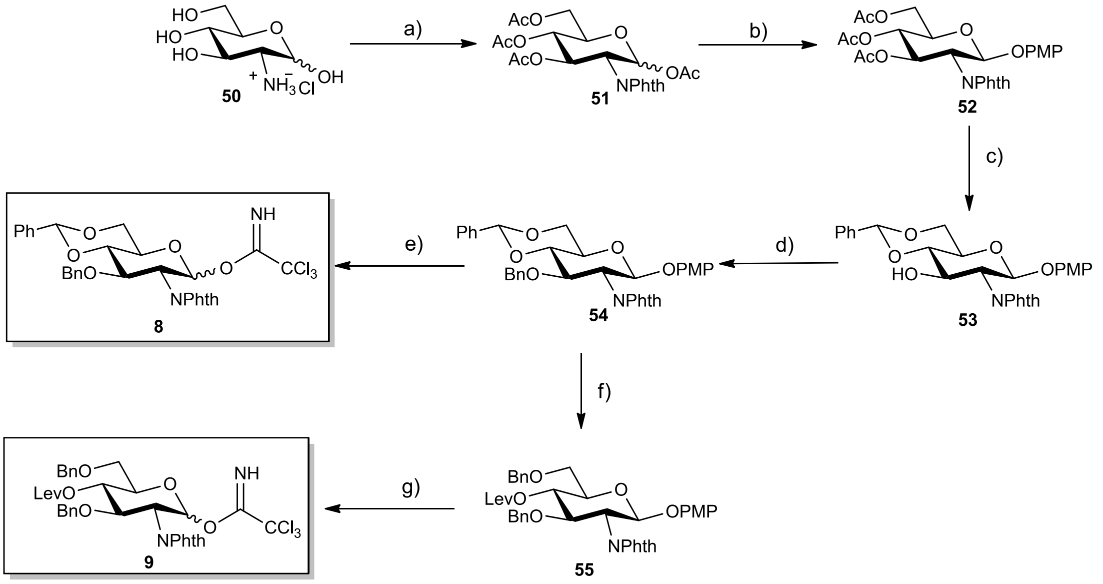

Scheme S1. *Reagents and conditions:* a) $(Phth)_2O$, TEA, MeOH; Ac_2O, Py, DMAP; 75% over two steps; b) *p*-methoxyphenol, $BF_3{\cdot}Et_2O$, DCM dry, 0°C, 89%; c) NaOMe, MeOH; $PhCH(OMe)_2$, PTSA, CH_3CN, 84% over two steps; d) BnBr, NaH, TBAI, DMF, 0°C, 82%; e) CAN, CH_3CN/H_2O, 0°C; CCl_3CN, DBU, DCM dry, 70% over two steps; f) $Me_3N{\cdot}BH_3$, $BF_3{\cdot}Et_2O$, ACN, 0°C; Levulinic acid, DCC, DMAP, dry DCM, 70% over two steps; g) CAN, CH_3CN/H_2O, 0°C; CCl_3CN, DBU, DCM dry, 70% over two steps.

Syntheses of the thioglycoside donors **6**, **7** and **16**[1]

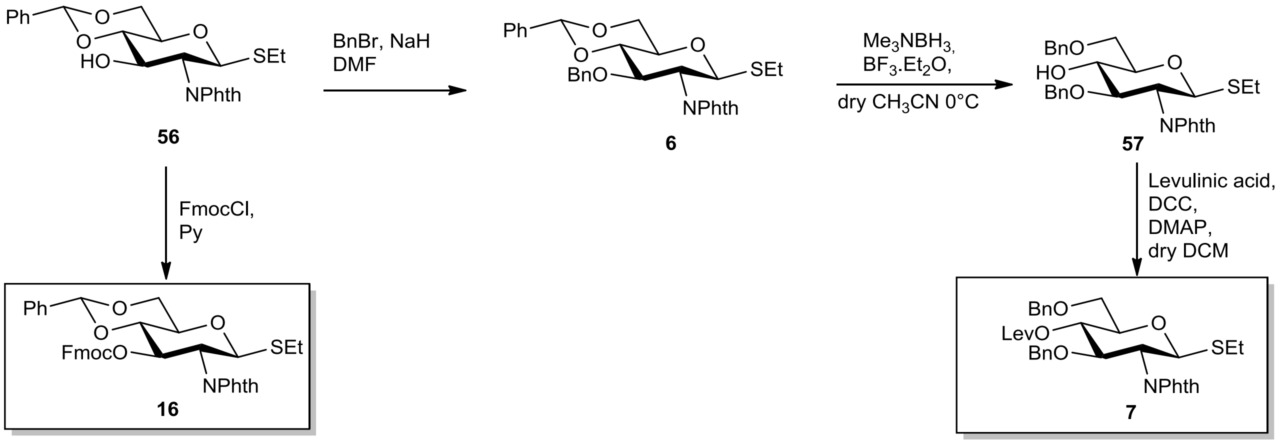

Scheme S2. *Reagents and conditions:* a) Fmoc-Cl, Py, DCM, 64%; b) BnBr, NaH, TBAI, DMF, 0°C, 82%; c) $Me_3N{\cdot}BH_3$, $BF_3{\cdot}Et_2O$, ACN, 0°C; 78% d) Levulinic acid, DCC, DMAP, dry DCM, 77%.

Syntheses of the thioglycoside donor **17**[2]

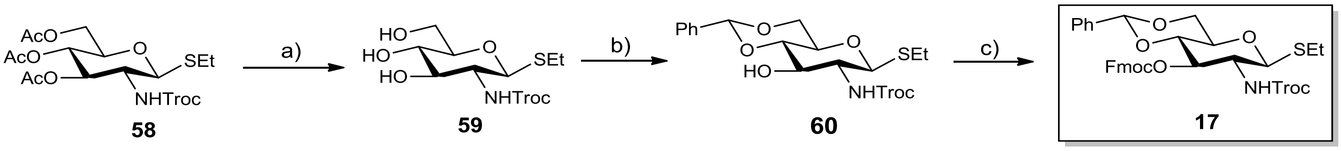

Scheme S3. *Reagents and conditions:* a) MeONa, MeOH, quantitative; b) $PhCH(OMe)_2$, PTSA, CH_3CN, 84%; c) Fmoc-Cl, Py, DCM, 94%.

Syntheses of the trichloroacetoimidate **18** and **19**

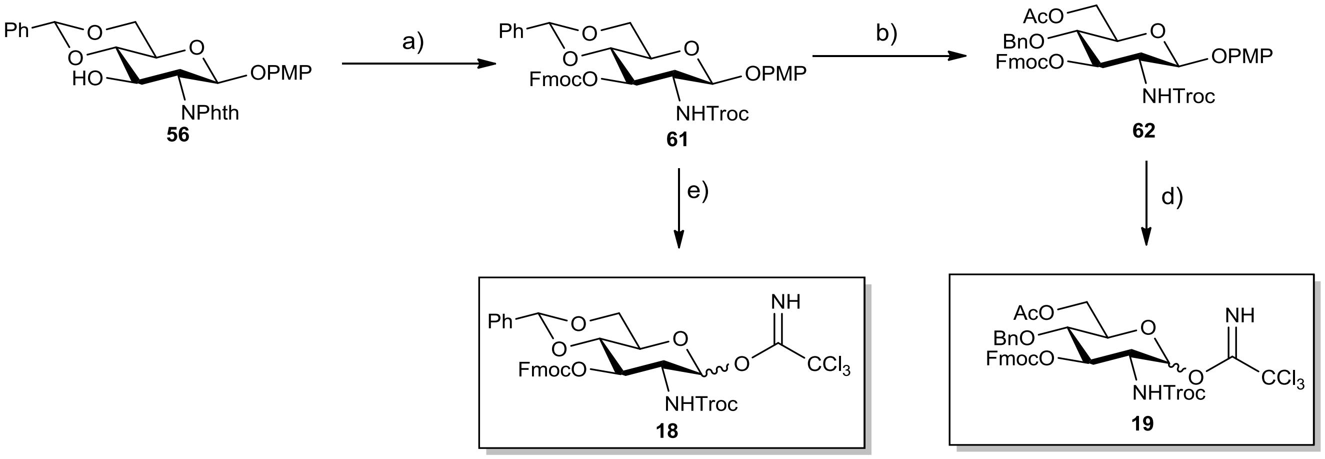

Scheme S4. *Reagent and Conditions*: a) $H_2NCH_2CH_2NH_2$, EtOH, 70° C; $NaHCO_3$, Troc-Cl, H_2O/Et_2O; Fmoc-Cl, DCM/Py 10:1, 60% yield over three steps; b) $PhBCl_2$, Et_3SiH, DCM dry, -78°C; Ac_2O/Py, 0° to rt, 50% yield over two steps; c) CAN, CH_3CN/H_2O, 0°C; CCl_3CN, DBU, DCM dry, 50% over two steps; d) CAN, CH_3CN/H_2O, 0°C; CCl_3CN, DBU, DCM dry, 41% over two steps.

Synthesis of the trifluoroacetoimidate **20**[3]

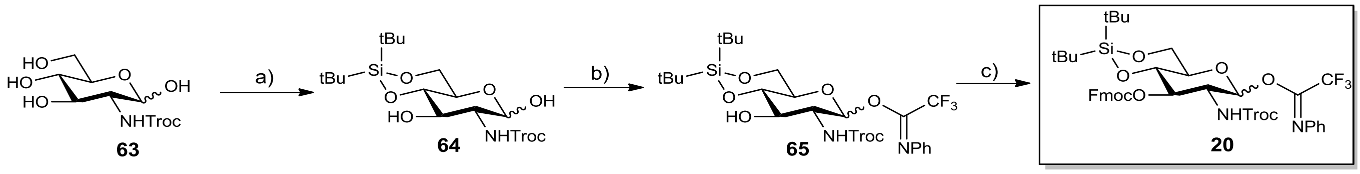

Scheme S5. *Reagents and conditions:* a) $(tBu)_2Si(OTf)_2$, DMF, -30°C, 90% b) TFA-Cl, Cs_2CO_3, DCM dry, 85% c) Fmoc-Cl, Py, DCM, 94%.

Syntheses of acceptors **10** and **11**[4]

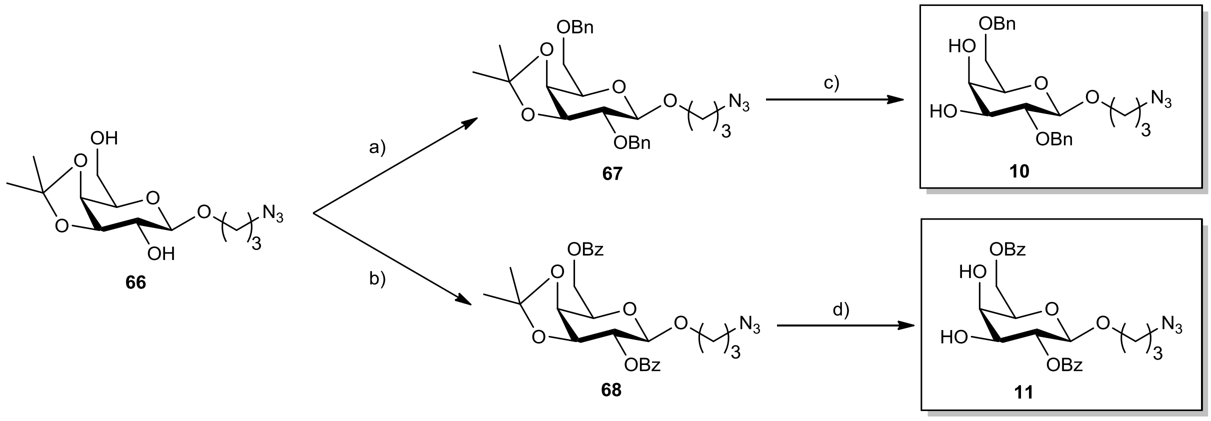

Scheme S6. *Reagent and conditions*: a) BnBr, NaH, DMF, 0°C to rt, 90%; b) BzCl, Py, 0° to rt, 95%; c) AcOH/H_2O, 70°C, 92%; d) AcOH/H_2O, 70°C, 92%.

Synthesis of acceptor **33**[5]

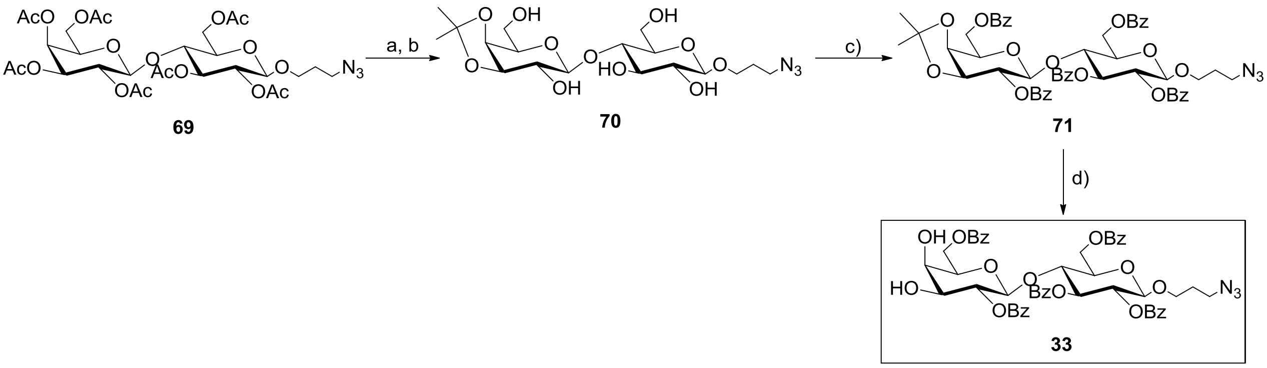

Scheme S7. *Reagent and conditions*: a) MeONa/MeOH, 85%; b) 9:1 2,2-dimethoxypropane/DMF, PTSA, 50°C; MeOH/H2O 9:1, 90°C, 57%; c) BzCl, Py, 0° to rt, 71%; d) AcOH/H_2O 4:1, 90°C, 70%.

Synthesis of donor **38**[6]

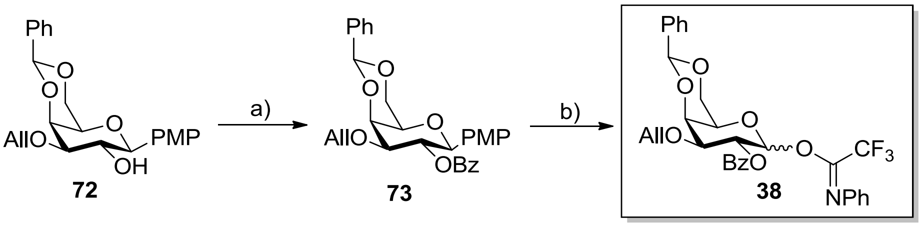

Scheme S8. *Reagent and conditions*: a) BzCl, Py, 0° to rt, 40%; b) CAN, ACN/water 4:1, 0°C, 77%; TFA-Cl, Cs_2CO_3, DCM dry, 0°C, 55%.

Initial route to GBS CPS Ib branched repeating unit **3**.

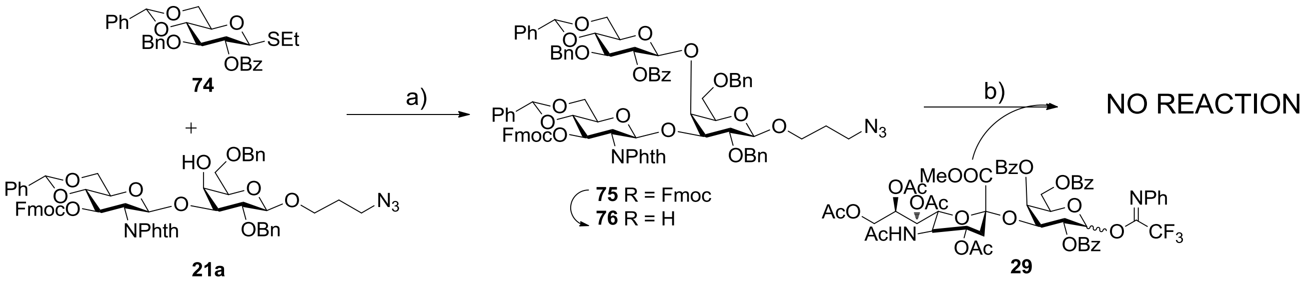

Scheme S9. *Reagent and conditions:* a) TfOH, NIS, DCM dry, 80%; b) TMSOTf, DCM dry, 0°C.

2. Experimental

2.1 General Methods

Reactions were monitored by thin-layer chromatography (TLC) on Silica Gel 60 F254 (Sigma Aldrich); after exam under UV light, compounds were visualized by heating with 10% (v/v) ethanolic H_2SO_4. In the work up procedures, organic solutions were washed with the amounts of the indicated aqueous solutions, then dried with anhydrous Na_2SO_4, and concentrated under reduced pressure at 30–50°C on a water bath. Column chromatography was performed on Silica Gel 60 (Sigma Aldrich, 0.040–0.063 nm) or using pre-packed silica cartridges RediSep (Teledyne-Isco, 0.040–0.063 nm) or Biotage SNAP Ultra (Biotage, silica 0.050 nm). Unless otherwise specified, a gradient 0 → 100% of the elution mixture was applied in a Combiflash Rf (Teledyne-Isco) or Biotage Isolera instrument. Solvent mixtures less polar than those used for TLC were used at the onset of separation. ^{1}H NMR spectra were measured at 400 MHz and 298 K with a Bruker AvanceIII 400 spectrometer; δH values are reported in ppm, relative to internal Me_4Si (δH = 0.00, $CDCl_3$); solvent peak for D_2O was calibrated at 4.79 ppm. ^{13}C NMR spectra were measured at 100 MHz and 298 K with a Bruker AvanceIII 400 spectrometer; δC values are reported in ppm relative to the signal of $CDCl_3$ (δC = 77.0, $CDCl_3$). Assignments of NMR signals were made by homonuclear and heteronuclear 2-dimensional correlation spectroscopy, run with the software supplied with the spectrometer. Assignment of ^{13}C NMR spectra of some compounds was aided by comparison with spectra of related substances reported previously from this laboratory or elsewhere. When reporting assignments of NMR signals, sugar residues in oligosaccharides are indicated with capital letters. Exact masses were measured by electron spray ionization cut-off spectroscopy, using a Q-Tof micro Macromass (Waters) instrument. Structures of these compounds follow unequivocally from the mode of synthesis, NMR data and *m/z* values found in their mass spectra.

2.2 Synthetic procedures

2.3 Synthesis of building blocks

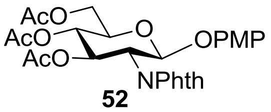

52

***p*-Methoxyphenyl 3,4,6-tri-*O*-acetyl-2-deoxy-2-phthalimido-*β*-D-glucopyranoside 52.** Compound **51** (17 g, 35.6 mmol) was dissolved in dry DCM (60.0 mL) at 0°C with 4Å activated molecular sieves (40 g) and stirred for 10 min under nitrogen. p-Methoxyphenol (25 g, 201.4 mmol) and boron trifluoride etherate (24 mL, 194.5 mmol) were added at 0°C. After 1 h the mixture was allowed to warm up to room temperature. Stirring was continued for further 24 h, when TLC showed complete reaction (7:3 cyclohexane/EtOAc). TEA was added, solid was filtered off and the solvent removed at reduced pressure. The crude was purified by flash chromatography (cyclohexane/EtOAc) giving **52** (18 g, 89%) as a brown oil. $[\alpha]_D^{25}$ = +63.04˚ (c 1.3, $CHCl_3$). ESI HR-MS ($C_{27}H_{27}NO_{11}$) *m/z* $[M+Na]^+$ found 564.1473; calcd 564.1482.

^{1}H NMR (400 MHz, CDCl3) δ 7.80-6.66 (m, 8H, H-Ar), 5.81 (m, 2H, H-1, H-3) , 5.19 (t, *J* = 9.7, 1H, H-4), 4.50 (dd, $J_{1,2}$ = 8.6 Hz, $J_{2,3}$ = 10.6 Hz, 1H, H-2), 4.29 (dd, $J_{5,6a}$ = 5.3 Hz, $J_{6a,6b}$ = 12.3 Hz, 1H, H-6a) , 4.16 (dd, $J_{5,6b}$ = 1.9, 1H, H-6b), 3.90-3.86 (m, 1H, H-5), 3.66 (s, 3H, OCH_3) , 2.04, 1.98, 1.82 (3 x s, 3H each, 3 x CH_3CO).

^{13}C NMR (101 MHz, $CDCl_3$) δ 170.8, 170.2, 169.5 (3 x CO), 134.4-114.4 (C-Ar), 97.5 (C-1), 72.0 (C-5), 70.7 (C-3), 68.9 (C-4), 62.0 (C-6), 55.6 (OCH_3), 54.5 (C-2), 20.8, 20.7, 20.5 (3 x $COCH_3$).

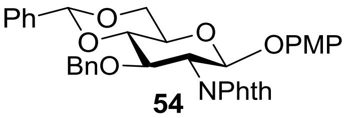

54

***p*-Methoxyphenyl 3-*O*-benzyl-4,6-*O*-benzylidene-2-deoxy-2-phthalimido-β-D-glucopyranoside 54.** Sodium methoxide was added to a stirred mixture of compound **52** (18.0 g, 35.6 mmol) in methanol (40 mL) until pH 9. After 20 h the reaction was quenched with Dowex 50WX2. After the filtration of the resin, the filtrate was evaporated under reduced pressure.

To the crude material acetonitrile (30 mL), benzaldehyde dimethyl acetal (6.9 mL, 68 mmol) and *p*-toluenesulfonic acid (0.470 g, 2.73 mmol) were added. After 3 h the reaction was quenched with triethylamine (4.7 mL), and the mixture was evaporated under reduced pressure. The crude was purified by flash chromatography (cyclohexane/EtOAc) to afford **53** (6.3 g, 84 % yield) as a yellow solid.

Sodium hydride (0.148 g, 3.7 mmol) was added to a stirred solution of compound **53** (0.930 g, 1.85 mmol) in N,N-dimethylformamide (7.0 mL) at 0°C under nitrogen. After 15 min benzyl bromide (0.66 mL, 5.55 mmol) was added, and the mixture was allowed warming to room temperature. After

2 h methanol (10 mL) was added, and the mixture was evaporated under reduced pressure. The product was dissolved in EtOAc and washed with $NaHCO_3$ (x2), dried (Na_2SO_4) and evaporated under reduced pressure. The crude was purified by flash chromatography (cyclohexane/EtOAc) to afford **54** (0.900 g, 82% yield) as a yellow solid. $[\alpha]_D^{25}$ = +65.17° (c 1.1, $CHCl_3$). ESI-HR MS ($C_{35}H_{31}NO_8$) *m/z* $[M+Na]^+$ found 616.1866; calcd 616.1947.

^{1}H NMR (400 MHz, $CDCl_3$) δ 7.78- 6.74 (m, 18H, H-Ar), 5.77 (d, $J_{1,2}$ = 7.9 Hz, 1H, H-1), 5.68 (s, 1H, C*H*Ph), 4.86 (d, ^2J=12.4 Hz, 1H, C*H*HPh), 4.57 (d, ^2J = 12.4 Hz, 1H, CH*H*Ph), 4.53-4.48 (m, 2H, H-3, H-2), 4.45 (dd, $J_{5,6a}$ = 4.9 Hz, $J_{6a,6b}$ = 10.4 Hz, 1H, H-6a), 3.98-3.88 (m, 2H, H-4, H-6b), 3.80-3.74 (m, 1H, H-5), 3.73 (s, 3H, OCH_3)

^{13}C NMR (101 MHz, $CDCl_3$) δ 134.00-114.53 (C-Ar), 101.40 (*C*HPh), 98.00 (C-1), 83.00 (C-4), 74.20 (*C*H_2Ph), 74.51 (C-3), 68.74 (C-6), 55.74 (C-2), 66.30 (C-5), 55.60 (O*C*H_3).

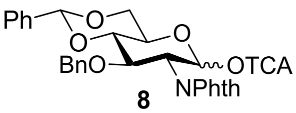

8

3-*O*-benzyl-4,6-*O*-benzylidene-2-deoxy-2-phthalimido-*α,β*-D-glucopyranosyl trichloroacetimidate 8. Cerium ammonium nitrate (5.110 g, 9.32 mmol) was added to a stirred solution of compound **54** (3 g, 4.66 mmol) in 4:1 acetonitrile: water (50 mL) at 0°C. After 3 h, TLC (7:3 cyclohexane/EtOAc) showed the disappearance of the starting material and the formation of one major spot. The reaction was washed with a solution of $NaHCO_3$ (x 2) and the combined organic phases were dried with Na_2SO_4 and evaporated under reduced pressure. The crude (1.681 g, 3.45 mmol) was dissolved in DCM (10 mL) dry under nitrogen and trichloroacetonitrile (1.730 mL, 17.25 mmol) and 1,8-diazobicyclo[5.4.0]undec-7-ene (0.152 mL, 1.03 mmol) were added. After stirring for 2h at rt, TLC (7:3 cyclohexane/EtOAc) showed complete reaction. The solvent was removed at reduced pressure and the crude was purified by flash chromatography (cyclohexane/EtOAc) to afford **8** (1.524 g) in 70% yield in 2:1 α/β ratio. $[\alpha]_D^{25}$ = +64.25° (c 4.15, $CHCl_3$). ESI MS ($C_{30}H_{25}Cl_3N_2O_7$) *m/z* $[M+H]^+$ found 632.06; calcd 631.89.

^{1}H NMR (400 MHz, $CDCl_3$) δ 8.5 (s, 1H, NH), 7.62-6.80 (m, 14H, H-Ar), 6.42 (d, $J_{1,2}$ = 8.4 Hz, H-1_β), 6.30 (d, $J_{1,2}$ = 3.8 Hz, H-1_α), 5.60 (s, 1H, C*H*Ph_α), 5.57 (s, 1H, C*H*Ph_β), 5.46 (t, J = 9.0 Hz, H-3_α), 4.95 (d, ^2J= 11.1 Hz, 1H, C*H*HPh_α), 4.75 (d, ^2J = 12.4 Hz, 1H, C*H*HPh_β), 4.62 (d, ^2J = 11.1 Hz, 1H, CH*H*Ph_α), 4.58-4-52 (m, H-2_α) , 4.94-4.36 (m, H-2_β, H-3_β, CH*H*Ph_β, H-$6a_\beta$), 4.33-4.31 (m, H-$6a_\alpha$), 4.18-4.12 (m, H-5_α), 3.86-3.76 (m, H-4_α, H-$6b_\alpha$, H-4_β, H-5_β, H-$6b_\beta$)

^{13}C NMR (101 MHz, $CDCl_3$) δ 134.0-123.4 (C-Ar), 101.4 (*C*HPh_β), 101.3 (*C*HPh_α), 95.4 (C-1_α), 94.3 (C-1_β), 83.4, 82.5 (C-4), 74.7, 74.3, 74.2 (C-3_β), 72.4 (C-3_α), 68.5, 66.9 (C-5_β), 65.4 (C-5_α), 54.7 (C-2).

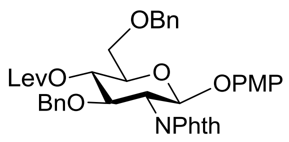

55

***p*-Methoxyphenyl 3,6-di-*O*-benzyl-2-deoxy-4-*O*-levulinoyl-2-phthalimido-*β*-D-glucopyranoside 55.** A solution of **54** (0.500 g, 0.9 mmol) in AcCN (5 mL) was cooled at 0°C. $Me_3N{\cdot}BH_3$ (274 mg, 3.76 mmol) and $BF_3{\cdot}OEt_2$ (0.464 mL, 3.76 mmol) were added, and the reaction was stirred for 2 h under nitrogen. TLC (7:3 cyclohexane/EtOAc) showed complete reaction. TEA was added, until neutral pH, followed by MeOH. The solvent was removed at reduced pressure and the crude was purified by flash chromatography (cyclohexane/EtOAc).

To the obtained product (0.400 g, 0.67 mmol) dissolved in DCM (5 mL), N-N-ethylcarbodiimide hydrochloride (0.206 g, 1.0 mmol), 4-dimethylaminopyridine (0.122 g, 1.0 mmol) and levulinic acid (0.156 g, 1.34 mmol) were added. The mixture was stirred overnight at rt. The solvent was removed by rotary evaporation, and the resulting crude material was purified by flash chromatography (cyclohexane/EtOAc) to give the compound **55** (325 mg) in 70% yield. $[\alpha]_D^{25}$ = +78.37° (c 3.25, $CHCl_3$). ESI HR-MS ($C_{40}H_{39}NO_{10}$) *m/z* $[M+Na]^+$ found 716.2447; calcd 716.2472.

^1H NMR (400 MHz, $CDCl_3$) δ 7.62-6.58 (m, 18H, H-Ar), 5.57 (d, $J_{1,2}$ =8.8 Hz, H-1), 5.14 (t, J = 8.9 Hz, 1H, H-4), 4.62 (d, ^2J = 11.9 Hz, 1H, C*H*Ph), 4.46 -4.42 (m, 3H, CH_2PH, H-3, H-2), 4.28 (d, ^2J = 11.9 Hz, 1H, C*H*HPh), 3.82-3.67 (m, 1H, H-5), 3.61 (s, 3H, OCH_3), 3.58-3.54 (m, 2H, H-6), 2.59 (t, J = 6.4 Hz, 2H, CH_2CO), 2.41 (t, J = 6.4Hz, 2H, CH_2COO), 2.07 (s, 3H, CH_3).

^{13}C NMR (101 MHz, $CDCl_3$) δ 206.2, 171.5, 160.8 (3 x CO), 137.0-114.0 (C-Ar), 97.4 (C-1), 72.9 (C-4), 74.2 (CH_2Ph), 73.59 (CH_2Ph), 77.24 (C-3), 55.41 (C-2), 73.8 (C-5), 69.56 (C-6), 55.55 (OCH_3), 37.77 (CH_2CO), 29.83 (CH_3), 27.94 (CH_2COO).

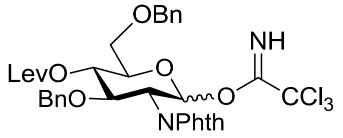

9

3.6-Di-*O*-benzyl-2-deoxy-4-*O*-levulinoyl-2-phthalimido-*β*-D-glucopyranosyl trichloroacetimidate 9. Cerium ammonium nitrate (515 mg, 0.94 mmol) was added to a stirred solution of compound **55** (325 mg, 0.47 mmol) in 4:1 acetonitrile: water (25 mL) at 0°C. After 3 h, a TLC (1:1 cyclohexane/EtOAc) showed the disappearance of the starting material and the formation of one major spots. The reaction was washed 2 times with a solution of $NaHCO_3$ and the organic phase was dried with Na_2SO_4 and evaporated under reduced pressure.

The crude was dissolved in DCM dry (10 mL) under nitrogen and trichloroacetonitrile (0.368 g, 2.55 mmol) and 1,8-diazobicyclo[5.4.0]undec-7-ene (0.023 g, 0.153 mmol) were respectively added. After stirring for 2h at rt, TLC showed complete reaction (1:1 cyclohexane/EtOAc). The solvent was removed at reduced pressure and the crude was purified by flash chromatography (cyclohexane/EtOAc) to afford **9** (261 mg, yield 70%). $[\alpha]_D^{25}$ = +68.49° (c 0.55, $CHCl_3$). ESI HR-MS ($C_{35}H_{33}Cl_3N_2O_9$) *m/z* $[M+Na]^+$ found 732.0040; calcd 732.0035.

^{1}H NMR (400 MHz, $CDCl_3$) δ 7.62-6.85 (m, 14H, H-Ar), 6.36 (d, $J_{1,2}$ = 7.6 Hz, H-1$_\beta$), 5.22 (t, J = 9.0 Hz, 1H, H-4), 4.62 (d, ^{2}J = 12.3 Hz, 1H, C*H*HPh$_b$), 4.53-4.39 (m, 4H, H-2, H-3, C*H*HPh$_a$, CH*H*Ph$_a$), 4.30 (d, ^{2}J = 12.3 Hz, 1H, CH*H*Ph$_b$), 3.92-3.85 (m, 1H, H-5), 3.66-3.53 (m, 2H, H-6), 2.65-2.48 (m, 2H, CH_2CO), 2.47-2.29 (m, 2H, CH_2COO), 2.06 (s, 3H, CH_3).
^{13}C NMR (101 MHz, $CDCl_3$) δ 206.4, 171.6, 160.8 (3 x CO), 133.9-123.3 (C-Ar), 93.9 (C-1), 76.7 (C-3), 74.44 (C-5), 74.11, 73.48, 72.17 (C-4), 68.89 (C-6), 54.46 (C-2), 37.69 (*C*H_2CO), 33.96, 29.79 (CH_3), 27.91 (*C*H_2COO), 25.62, 24.95.

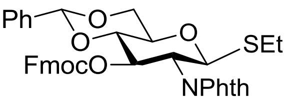

16

Ethylthio 4,6-*O*-benzylidene-2-deoxy-3-*O*-(9*H*-fluoren-9-ylmethylcarbonate)-2-phthalimido-*β*-D-glucopyranoside 16. The known compound **56** (200 mg, 0.45 mmol) was dissolved in dry DCM (10 mL) and Fmoc (351 mg, 1.36 mmol), pyridine (0.182 mL, 2.25 mmol) was added at 0°C, and the reaction stirred rt for 1 h. TLC (4:1 cyclohexane:EtOAc) showed complete reaction, the solvent was removed under reduced pressure and the crude was purified by flash chromatography (8: 2 cyclohexane/EtOAc) to afford **16** (202 mg) in 64% yield as pale yellow oil. $[\alpha]_D^{25}$= +13.53° (c 2.5, $CHCl_3$). ESI HR-MS ($C_{38}H_{33}NO_8S$) *m/z* $[M+Na]^+$ found 686.1807; calcd 686.1825.
^{1}H NMR (400 MHz, $CDCl_3$) δ 7.16-7.95 (m, 17H, H-Ar), 5.88 (t, J= 9.5 Hz, 1H, H-3), 5.59-5.65 (m, 2H, C*H*Ph, H-1), 4.58 (t, J= 10.3 Hz, 1H, H-2), 4.47-4.52 (m, 1H, CH_{2a}^{Fmoc}), 4.09-4.17 (m, 2H, H-6), 3.92-4.00 (m, 2H, CH^{Fmoc}, H-4), 3.84-3.92 (m, 2H, CH_{2b}^{Fmoc}, H-5), 2.65-2.85 (m, 2H, SC*H*$_2$), 1.25 (t, J = 7.3 Hz, 3H, SCH_2CH_3).
^{13}C NMR (101 MHz, $CDCl_3$) δ 167.9, 167.2, 154.5 (3 x CO), 143.1-119.9 (C-Ar), 101.8 (*C*HPh), 81.9 (C-1), 79.2 (C-4), 74.4 (C-3), 70.5 (C-5), 70.3, 68.6 (C-6), 55.4, 54.1 (C-2), 46.3, 26.9, 24.4 (S*C*H_2), 14.9 (SCH_2CH_3).

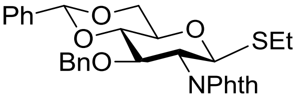

6

Ethylthio 3-benzyl-4,6-*O*-benzylidene-2-deoxy-2-phthalimido-*β*-D-glucopyranoside 6. The compound **56** (4 g, 9 mmol) was dissolved in dry DMF (15 mL) under nitrogen atmosphere. The solution was cooled at 0°C, and 60 % NaH (0.726 g, 30 mmol) was added portion wise. After 20 min BnBr (3.14 mL, 26 mmol) and TBAI (200 mg) were added. The reaction was stirred overnight at rt, then quenched adding MeOH and solvent removed at reduced pressure. The crude was dissolved in EtOAc washed 3 times with aq $NaHCO_3$. The organic phase was collected, dried with Na_2SO_4 and evaporated under reduced pressure. The crude was purified by flash chromatography (cyclohexane:/EtOAc) to afford **6** (3.9 g) in 82% yield. NMR data were in agreement with those reported in the literature.[1]

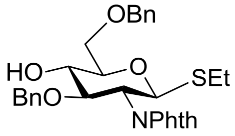

57

Ethylthio 3,6-di-*O*-benzyl-2-deoxy-2-phthalimido-*β*-D-glucopyranoside 57. To a solution of **6** (500 mg, 0.94 mmol) in AcCN (10 mL) was cooled at 0°C. Me_3NBH_3 (274 mg, 3.76 mmol) and $BF_3{\cdot}OEt_2$ (3.76 mmol, 0.464 mL) were added and the reaction was stirred for 2 h under nitrogen. TLC showed complete reaction (7:3 cyclohexane:EtOAc). First TEA and then MeOH were added until neutral pH. The solvent removed at reduced pressure and the crude was purified by flash chromatography (cyclohexane:EtOAc) to afford **57** (388 mg, 78%). NMR spectra were in agreement with those reported in literature.[1]

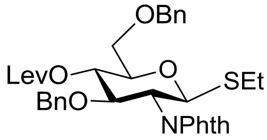

7

Ethylthio 3,6-di-*O*-benzyl-2-deoxy-4-*O*-levulinoyl-2-phthalimido-*β*-D-glucopyranoside 7. Compound **57** (0.388 g, 0.73 mmol) was dissolved in dry DCM (10 mL). Levulinic acid (170 mg, 1.46 mmol), DCC (225 mg, 1.09 mmol) and DMAP (132 mg, 1.09 mmol) were added and the reaction stirred at rt for 3 h. The solvent was removed under reduced pressure, and the crude purified by flash chromatography (cyclohexane/EtOAc) to afford **7** (353 mg, 77% yield). The NMR data agreed with those described in the literature.[7]

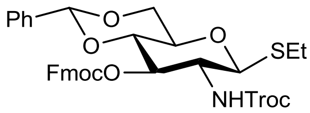

17

Ethylthio 4,6-*O*-benzylidene-2-deoxy-3-*O*-(9*H*-fluoren-9-ylmethylcarbonate)-2-(2,2,2-trichloroethoxycarbonylamino)-*β*-D-glucopyranoside 17. Compound **60** (219 mg, 0.45 mmol) was dissolved in 5.0 mL of dry DCM and the resulting solution was cooled down to 0°C. Pyridine (0.250 mL) was added, followed by FmocCl (0.310 mL, 1.125 mmol). The reaction mixture was stirred for 30 minutes. The crude was purified by column chromatography (7:3 cyclohexane/EtOAc) to afford **17** (300 mg, 94% yield) as a white solid. $[\alpha]_D^{25}$= -14.18° (c 0.025, $CHCl_3$). ESI HR-MS ($C_{33}H_{32}Cl_3NO_8S$) *m/z* $[M+Na]^+$ found 730.0852; calcd 730.0812.

^{1}H NMR (400 MHz, $CDCl_3$) δ 7.81-7.19 (m, 13H, Ar-H), 5.59 (s, 1H, C*H*Ph), 5.31 (d, $J_{N,H}$ = 9.2 Hz, 1H, NH), 5.20 (t, *J* = 9.8 Hz, 1H, H-3), 4.7 (d, $J_{1,2}$ = 10.2 Hz, 1H, H-1), 4.65 (d, ^2J = 2.54 Hz, 2H, Cl_3CCH_2), 4.45-4.35 (m, 3H, H-6, CH_2^{Fmoc}), 4.26 (t, *J* = 7.5 Hz, 1H, CH^{Fmoc}), 3.92 (t, *J* = 9.9 Hz, 1 H, H-2), 3.88-3.80 (m, 2H, H-6', H-4), 3.65-3.57 (m, 1H, H-5), 2.70-2.78 (m, 2H, CH_2SEt), 1.28 (t, 3H, *J* = 7.4 Hz, CH_3SEt).

^{13}C NMR (101 MHz, $CDCl_3$) δ 129.3-120.2 (C-Ar), 101.8 (*C*HPh), 85.5 (C-1), 78.7 (C-4), 76.5 (C-3), 74.8 (Cl_3C*C*H_2), 71.0 (*C*H_2^{Fmoc}), 70.7 (C-6), 68.7 (C-5), 56.1 (C-2), 46.7 (*C*H^{Fmoc}), 24.6 (*C*H_2 SEt), 14.9 (*C*H_3SEt).

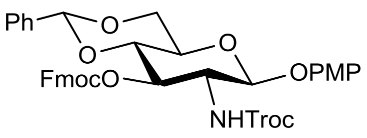

61

***p*-Methoxyphenyl 4,6-*O*-benzylidene-2-deoxy-3-*O*-(9*H*-fluoren-9-ylmethylcarbonate)-2-(2,2,2-trichloroethoxycarbonylamino)-*β*-D-glucopyranoside 61.** Compound **56** (2.014 g, 3.64 mmol) was dissolved in 20.0 mL of ethanol and ethylenediamine (1.2 mL, 18.22 mmol) was added. The resulting reaction mixture was stirred at 70°C for 6 h. After 6 h TLC (1:1 cyclohexane/EtOAc) showed full conversion of the starting material and formation of a new spot on the baseline. The reaction mixture was evaporated under reduced pressure affording a white solid. The crude was resuspended in methanol and filtered under vacuum. The filtrate was evaporated to dryness affording a yellow syrup, which was re-dissolved in 20.0 mL of a 1:1 Et_2O/H_2O mixture. The resulting solution was cooled down to 0°C and $NaHCO_3$ (3.06 mg, 36.4 mmol) was added, followed by Troc-Cl (2.5 mL, 18.2 mmol). The resulting reaction mixture was stirred at 0°C for 45 min, then checked by TLC (7:3 cyclohexane/EtOAc), which showed complete consumption of the starting material. The reaction mixture was quenched by addition of aqueous $NaHCO_3$, then extracted with DCM (3 x 10 mL). The organic phase was dried over Na_2SO_4, filtered and evaporated under reduced pressure affording a pale solid as a crude. The crude was purified by flash column chromatography using a gradient from 0 to 100% of EtOAc in cyclohexane. Clean fractions were collected and evaporated under reduced pressure affording the product as a white solid (1.419 g, 66% yield). The compound (1.2 g, 2.03 mmol) was dissolved in 10.0 mL of dry DCM. The resulting solution was cooled down to 0°C in an ice-water bath, then Fmoc-Cl (1.3 g, 5.078 mmol) and pyridine (0.5 mL) were added. The reaction mixture was stirred for 1 h at room temperature, then checked by TLC (cyclohexane/EtOAc), which showed full conversion of starting material. The crude was evaporated under reduced pressure and purified by flash column chromatography. Pure fractions were collected and evaporated to dryness affording compound **61** (1.3 g, 60% yield over three steps). $[\alpha]_D^{25}$ = -1.19° (c 1.1, $CHCl_3$). ESI HR-MS ($C_{38}H_{34}Cl_3NO_{10}$) *m/z* $[M+NH_4]^+$ found 787.1596; calcd 787.1587.

^{1}H NMR (400 MHz, $CDCl_3$) δ 7.60-2.22 (m, Ar-H), 5.58 (s, 1H, C*H*Ph), 5.48 (d, $J_{N,H}$ = 8.7 Hz, 1H, NH), 5.33 (t, J = 10.0 Hz, 1H, H-3), 5.15 (d, $J_{1,2}$ = 8.2 Hz, 1H, H-1), 4.70 (d, ^2J = 11.8 Hz, 1H, C*H*HCCl3), 4.63 (d, 1H, CH*H*CCl3), 4.47-4.33 (m, C*H*H^{Fmoc}, 3H, H-6), 4.25 (t, J = 7.2 Hz, 1H, CH^{Fmoc}), 3.97 (t, J = 8.9 Hz, 1H, H-2), 3.90-3.82 (m, 2H, C*H*H^{Fmoc}, H-4), 3.77 (s, 3H, OCH_3), 3.64-3.56 (m, 1H, H-5).

^{13}C NMR (101 MHz, $CDCl_3$) δ 155.8, 155.1 (2 x CO), 154.2 (C_q), 151.0 (C_q), 144.3-114.6 (C-Ar), 101.6 (*C*HPh), 100.9 (C-1), 78.5 (C-4), 74.9 (C-3), 74.5 (*C*H_2CCl_3), 70.5 (C-6), 68.5 (*C*H_2^{Fmoc}), 66.3 (C-5), 57.1 (C-2), 55.7 (O*C*H_3), 46.5 (*C*H^{Fmoc}).

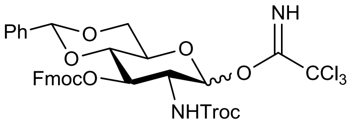

18

4,6-*O*-benzylidene-3-*O*-(9*H*-fluoren-9-ylmethylcarbonate)-2-deoxy-2-(2,2,2-trichloroethoxycarbonylamino)-*α,β*-D-glucopyranosyl trichloroacetimidate 18. Compound **61** (1.550 g, 2.06 mmol) was dissolved in 15 mL of a 4:1 ACN/H_2O mixture and the resulting suspension was cooled down to 0°C. Cerium ammonium nitrate (2.16 g, 4.12 mmol) was added and the reaction was stirred for 2 h at 0°C. After 2 h, TLC (6:4 cyclohexane:EtOAc) showed disappearance of the starting material. The reaction mixture was diluted with DCM and washed twice with iced aqueous $NaHCO_3$. The organic phase was dried over Na_2SO_4, filtered and evaporated under reduced pressure. The crude was purified by column chromatography (cyclohexane:EtOAc). Pure fractions were collected and evaporated affording the target 1-OH intermediate (1.1 g, 80% yield) as colorless oil.

The 1-OH intermediate (0.212 g, 0.319 mmol) was dissolved in a 1:1 DCM:CCl_3CN mixture under nitrogen atmosphere and cooled down to 0°C. NaH (1.28 mg, 0.032 mmol) was added and the resulting solution was stirred for 3 h and let slowly to go to room temperature. After 3 h TLC (6:4 cyclohexane/EtOAc) showed full conversion of the starting material; the reaction mixture was evaporated under reduced pressure and the crude was purified on column chromatography with a gradient from 0 to 100% of EtOAc in hexane (containing 3% of TEA). Pure fractions were collected and evaporated under reduced pressure affording the glucosamine imidate **18** as a colorless oil (0.125 g, 50% yield,) primarily as α anomer. $[\alpha]_D^{25}$ = +31.33˚ (c 0.2, $CHCl_3$). ESI HR-MS ($C_{33}H_{28}Cl_6N_2O_9$) *m/z* $[M+H]^+$ found 806.0878; calcd 806.9926.

^{1}H NMR (400 MHz, $CDCl_3$) δ 8.74 (s, 1H, C=NH); 7.54-7.14 (m, 13H, Ar-H); 6.39 (d, $J_{1,2}$ = 3.6 Hz, 1H, H-1); 5.57 8s, 1H, C*H*Ph); 5.38 (d, $J_{N.H}$ = 9.7 Hz, 1H, NH), 5.28 (t, *J* = 10.1 Hz, 1H, H-3), 4.67 (d, ^2J = 12.0 Hz, 1H, C*H*HCCl_3), 4.48 (d, 1H, CH*H*CCl_3), 4.41-4.30 (m, 3H, incl. H-2, H-6); 4.25-4.19 (m, 1H, CH^{Fmoc}), 4.09-4.02 (m, 1H, H-5), 3.91 (t, 1H, *J* = 9.7 Hz, H-4), 3.79 (t, *J* = 10.4 Hz, 1H, H-6b).

^{13}C NMR (101 MHz, $CDCl_3$) δ 160.7 (CO), 143.1, 143.0, 129.6-120.0 (C-Ar), 101.7 (*C*HPh), 95.1 (C-1), 78.3 (C-4), 77.2, 74.7 (*C*H_2CCl_3), 73.4 (C-3), 70.7 (*C*H_2^{Fmoc}), 70.1 (*C*H_2Ph), 68.5 (C-6), 65.4 (C-5), 65.2, 54.6 (C-2), 50.4, 46.4 (*C*H^{Fmoc}).

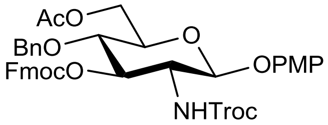

62

***p*-Methoxyphenyl 6-*O*-acetyl-4-*O*-benzyl-2-deoxy-3-*O*-(9*H*-fluoren-9-ylmethylcarbonate)-2-(2,2,2-trichloroethoxycarbonylamino)-*β*-D-glucopyranoside 62.** Compound **61** (370 mg, 0.49 mmol) was dissolved in 5.0 mL of dry DCM under nitrogen atmosphere in presence of 4 Å activated molecular sieves. The resulting suspension was cooled down to -78°C and dichlorophenylborane (0.229 mL, 1.764 mmol) and Et_3SiH (0.549 mL, 3.43 mmol) were added. The resulting reaction mixture was stirred and

allowed to slowly reach room temperature. TLC (3:2 cyclohexane/EtOAc) showed consumption of the starting material, the reaction was quenched by addition of saturated aqueous $NaHCO_3$ and extracted with DCM (3 x 10 mL). The organic phase was dried over Na_2SO_4, filtered and evaporated under reduced pressure. The crude was dissolved in a 1:1 solution of Ac_2O/pyridine. The resulting reaction mixture was stirred at room temperature. After 16 h TLC (3:2 cyclohexane/EtOAc) showed complete reaction, then the crude mixture was coevaporated with toluene and the residue was purified by column chromatography (cyclohexane/EtOAc). Pure fractions were collected and evaporated to dryness affording glucosamine **62** (185 mg, 50% yield over two steps). $[\alpha]_D^{25}$ = -11.07° (c 0.025, $CHCl_3$). ESI HR-MS ($C_{41}H_{40}Cl_3NO_{10}$) *m/z* $[M+Na]^+$ found 834.1625; calcd 834.1615.

^{1}H NMR (400 MHz, $CDCl_3$) δ 7.80-6.76 (m, 22H, Ar-H); 5.36 (d, $J_{N,H}$ = 9.0 Hz, 1H, NH), 5.18 (dd, $J_{3,4}$ = 10.5 Hz, $J_{2,3}$ = 8.8 Hz, 1H, H-3), 4.99 (d, $J_{1,2}$ = 8.2 Hz, 1H, H-1), 4.72-4.62 (m, 3H, CH_2CCl$_3$, C*H*HPh), 4.54 (d, ^2J = 11.5 Hz, 1H, CH*H*Ph), 4.51-4.44 (m, 1H, C*H*H^{Fmoc}), 4.41-4.32 (m, 2H, H-6a, CH*H*^{Fmoc}), 4.27-4-20 (m, 2H, CH^{Fmoc}, H-6b), 3.94 (t, 1H, J = 8.9 Hz, H-2), 3.79-3.65 (m, 2H, H-4, H-5), 3.76 (s, 3H, OCH_3), 2.04 (s, 3H, CH_3CO).

^{13}C NMR (101 MHz, $CDCl_3$) δ 170.6, 155.8, 155.3 (3 x CO), 154.2-114.5 (C-Ar), 100.4 (C-1), 78.7 (C-3), 75.3 (C-5), 74.8 (*C*H$_2$CCl$_3$), 72.8 (C-4), 70.5 (*C*H$_2^{Fmoc}$), 62.6 (C-6), 56.5 (C-2), 55.7 (O*C*H$_3$), 46.7 (CH^{Fmoc}), 20.8 (*C*H$_3$CO).

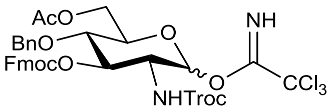

19

6-*O*-Acetyl-4-*O*-benzyl-2-deoxy-3-*O*-(9*H*-fluoren-9-ylmethylcarbonate)-2-(2,2,2-trichloroethoxycarbonylamino)-α,β-D-glucopyranosyl trichloroacetimidate 19. Compound **62** (185 mg, 0.227 mmol) was dissolved in 10 mL of a 4:1 acetonitrile/water mixture and the resulting solution was cooled down to 0°C using a water/ice bath. Cerium ammonium nitrate (373 mg, 0.680 mmol) was added and the resulting reaction mixture was stirred at 0°C. After 2 h TLC (3:2 cyclohexane/EtOAc) showed full consumption of the starting material. The reaction mixture was poured into iced $NaHCO_3$ and extracted with DCM (3 x 20 mL). The organic phases were dried over Na_2SO_4 and evaporated under reduced pressure affording a crude product which was directly dissolved in 2.0 mL of a 1:1 CCl_3CN/DCM mixture. The resulting solution was cooled down to 0°C, NaH (60% dispersion in mineral oil, 1.0 mg, 0.0212 mmol) and trichloroacetonitrile (2 mL) were added and the resulting reaction mixture was stirred for 2 h, letting slowly reach room temperature.

After 2 h, TLC (6:4 cyclohexane/EtOAc) showed full conversion of the starting material, so the reaction was quenched by addition of Et_3N and the reaction mixture was evaporated to dryness. The crude was purified by column chromatography with a gradient of EtOAc in cyclohexane (containing 1% of TEA). Pure fractions were collected and evaporated to dryness affording the target compound

19 as a clear oil (0.085 g, 41% yield over two steps, only α). $[\alpha]_D^{25}$ = +9.63° (c 0.85, CHCl₃). ESI HR-MS ($C_{36}H_{34}Cl_6N_2O_9$) *m/z* $[M+Na]^+$ found 871.0300; calcd 871.0293.

^{1}H NMR (400 MHz, $CDCl_3$) δ 8.79 (s, 1H, $NHCCl_3$); 7.84-7.24 (m, 13H, Ar-H); 6.42 (d, $J_{1,2}$ = 3.4 Hz, 1H, H-1), 5.37 (d, $J_{N,H}$ = 9.2 Hz, 1H, NH), 5.30 (dd, $J_{3,4}$ = 11.4 Hz, $J_{2,3}$ = 9.6 Hz, 1H, H-3); 4.78 (d, ^2J = 11.0 Hz, 1H, C*H*HPh), 4.71-4.50 (m, 4H, CH_2CCl_3, CH*H*Ph, CH^{Fmoc}), 4.56-4.24 (m, 5H, H-2, H-6, CH_2^{Fmoc}), 4.09 (dt, 1H, $J_{4,5}$ =9.9 Hz, $J_{5,6}$ = 2.9 Hz, H-5); 3.99 (t, 1H, *J* = 9.5 Hz, H-4), 2.06 (s, 3H, CH_3CO).

^{13}C NMR (101 MHz, $CDCl_3$) δ 170.6 (CNH), 163.7, 160.6, 155.6 (CO), 143.2-120.1 (C-Ar), 100.0 (Cq), 94.8 (C-1), 77.2 (C-3), 75.1 (*C*H₂Ph), 74.6 (*C*H₂CCl₃), 74.1 (C-4), 71.4 (C-5), 70.7 (CH_2^{Fmoc}), 62.0 (C-6), 54.2 (C-2), 46.5 (CH^{Fmoc}), 20.8 (*C*H₃CO).

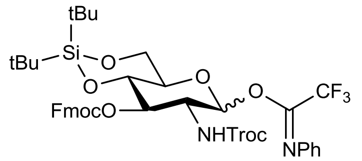

20

4,6-*O*-di-*tert*-butylsilylidene-2-deoxy-3-*O*-(9*H*-fluoren-9-ylmethylcarbonate)-2-(2,2,2-trichloroethoxycarbonylamino)-*α,β*-D-glucopyranosyl *N*-phenyl-trifluoroacetimidate 20. Compound **65** (2.278 g, 3.42 mmol) was dissolved in 10.0 mL of dry DCM and the resulting solution was cooled down to 0°C. Pyridine (1.0 mL) was added, followed by Fmoc-Cl (2.2 g, 8.55 mmol). The reaction mixture was stirred for 1 hour at room temperature. The crude was purified by column chromatography (9:1 cyclohexane/EtOAc) to afford **20** (2.0 g, 70% yield) as a white solid. $[\alpha]_D^{25}$ = +27.87° (c 0.05, CHCl₃). ESI HR-MS ($C_{40}H_{44}Cl_3F_3N_2O_9Si$) *m/z* $[M+Na-CNPhCF_3]^+$ found 737.3012; calcd 738.1430.

^{1}H NMR (400 MHz, $CDCl_3$) δ 7.82-6.78 (m, 13H, Ar-H); 5.99 (bs, 1H, H-1), 5.26 (d, $J_{N,H}$ = 8.9 Hz, 1H, NH), 5.10-5.01 (m, 1H, H-3), 4.73 (d, ^2J = 12.1 Hz, 1H, C*H*HCCl₃), 4.62 (d, 1H, CH*H*CCl₃); 4.47 (dd, *J* = 9.9, 7.9 Hz, 1H, CHH^{Fmoc}), 4.40-4.33 (m, 1H, CHH^{Fmoc}), 4.31-4.15 (m, 3H, H-2, CH^{Fmoc}, H-6a); 4.10-3.91 (m, 3H, H-6b, H-4, H-5), 1.05 (s, 9H, tBu), 0.95 (s, 9H, tBu).

^{13}C NMR (101 MHz, $CDCl_3$) δ 155.3, 154.0 (CO), 143.2 (C_q), 141.3 (C_q), 129.4-119.2 (C-Ar), 94.9 (CCl₃), 93.6 (C-1), 77.6 (C-3), 74.6 (C-4, C-5), 75.4 (*C*H₂CCl₃), 71.4 (C_q), 70.6 (CH_2^{Fmoc}), 65.9 (C-6), 55.6 (C-2), 46.5 (CH^{Fmoc}), 27.3 (tBu), 26.8 (tBu), 22.7 (*C*(CH₃)₃), 20.0 (*C*(CH₃)₃).

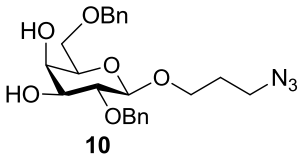

10

3-Azidopropyl 2,6-di-*O*-benzyl-*β*-D-galactopyranoside 10. A suspension of compound **67**[4] (3.0 g, 5.7 mmol) in 80% aqueous AcOH (20 mL) was stirred at 70°C for 2 h when TLC (7:3 cyclohexane/EtOAc) showed complete conversion of the starting material to a slower moving spot. Solvents were evaporated in vacuo, coevaporated with toluene to remove traces of AcOH. The residue was purified by flash chromatography using cyclohexane: EtOAc as eluent to give the pure product

10 (2.5 g, 92%) as yellow oil. $[\alpha]_D^{25}$ = +12.78° (c 1.05, $CHCl_3$). ESI HR-MS ($C_{23}H_{29}N_3O_6$) *m/z* $[M+Na]^+$ found 466.2023; calcd 466.1954.

^{1}H NMR (400 MHz, $CDCl_3$) δ 7.46-7.25 (m, 10H, H-Ar), 4.95 (d, ^2J= 11.6 Hz, 1H, C*H*HPh), 4.69 (d, ^2J = 11.6 Hz, 1H, CH*H*Ph), 4.62 (s, 2H, CH_2Ph), 4.38 (d, $J_{1,2}$ = 7.7 Hz, 1H, H-1), 4.07-4.00 (m, 2H, OCH_{2b}, H-4), 3.83-3.73 (m, 2H, H-6), 3.69-3.58 (m, 3H, OCH_{2a}, H-3, H-2), 3.55-3.49 (m, 1H, H-5), 3.44 (t, J = 5.4 Hz, 2H, CH_2N_3), 1.93 (m, 2H, C$H_2CH_2N_3$).

^{13}C NMR (101 MHz, $CDCl_3$) δ 128.6-127.7 (C-Ar), 103.6 (C-1), 79.16 (C-5), 74.73 (*C*H_2Ph), 73.72 (*C*H_2Ph), 73.28 (C-2), 73.13 (C-3), 69.34 (C-6), 68.94 (C-4), 66.54 (O*C*H_2), 48.37 (*C*H_2N_3), 29.27 (*C*$H_2CH_2N_3$).

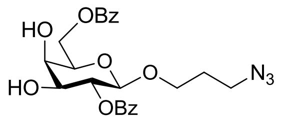

11

3-Azidopropyl 2,6-di-*O*-benzoyl-*β*-D-galactopyranoside 11. A suspension of compound **68**[4] (3.0 g, 5.7 mmol) in 80% aqueous AcOH (20 mL) was stirred at 70°C for 2 h when TLC (7:3 cyclohexane/EtOAc) showed complete conversion of the starting material to a slower moving spot. Solvents were evaporated in vacuo, coevaporated with toluene to remove traces of AcOH. The residue was purified by flash chromatography using cyclohexane/EtOAc as eluent to give the pure product **11** (2.5 g, 92%). $[\alpha]_D^{25}$ = -3.94° (c 0.45, $CHCl_3$). ESI HR-MS ($C_{23}H_{25}N_3O_8$) *m/z* $[M+Na]^+$ found 494.1591; calcd 494.1539.

^{1}H NMR (400 MHz, $CDCl_3$) δ 8.06-7.38 (m, 10H, H-Ar), 5.14 (t, J = 8.9 Hz, 1H, H-2), 4.69-4.64 (m, 1H, H-6a), 4.56-4.50 (m, 2H, H-1, H-6b), 3.98 (d, $J_{3,4}$ = 2.5 Hz, 1H, H-4), 3.97-3.88 (m, 1H, OCH_{2a}), 3.86-3.83 (m, 1H, H-5), 3.81-3.78 (m, 1H, H-3), 3.59-3.53 (m, 1H, OCH_{2b}), 3.21 (t, J = 6.5 Hz, 2H, CH_2N_3), 1.82-1.65 (m, 2H, C$H_2CH_2N_3$).

^{13}C NMR (101 MHz, $CDCl_3$) δ 167.29, 166.62 (2 x CO), 133.7-128.4 (C-Ar), 101.09 (C-1), 99.9, 74.31 (C-2), 72.78 (C-3), 72.21 (C-5), 68.59 (C-4), 66.38 (O*C*H_2), 62.77 (C-6), 47.95 (*C*H_2N_3), 29.03 (*C*$H_2CH_2N_3$).

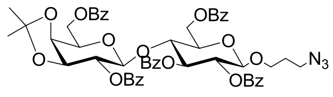

71

3-Azidopropyl 2,6-di-*O*-benzoyl-3,4-di-*O*-isopropylidene-*β*-D-galactopyranosyl-(1→4)-2,3,6-tri-*O*-benzoyl-*β*-D-glucopyranoside 71. Lactose **69** (5.4 g, 7.6 mmol), prepared according to the literature[5], was dissolved in MeOH (40 mL) and then MeONa/MeOH was added dropwise until pH = 9. After 16 h (TLC 9:1 DCM/MeOH) the reaction was quenched by addition of Dowex, then the solution was filtered and the solvent evaporated under reduced pressure, affording the lactose linker deacetylated. (2.7 g, 6.4 mmol, 85%).

The resulting compound (2.7 g, 6.4 mmol) was dissolved in 30 mL of 9:1 mixture of 2,2-dimethoxypropane:DMF. Catalytic PTSA (0.7 g, 4.5 mmol) was added and the reaction warmed at

50°C for 3 h. After 3 h (TLC 9:1 DCM/MeOH) the reaction was quenched with TEA until neutral pH, and the solvent removed under reduced pressure. The crude was dissolved in 55 mL of 9:1 MeOH/H_2O and stirred at 90°C for 2 h, when the presence of one major spot was detected at TLC. The solvent was removed under reduced pressure, and the crude purified by chromatography (DCM/MeOH) to give the isopropylinated lactose **70** in 57% yield (1.7 g, 3.7 mmol).

Lactose **70** (1.7 g, 3.7 mmol) was dissolved in 20 mL of pyridine, then the resulting solution was cooled down to 0°C in a water/ice bath and BzCl (4.3 mL, 37.2 mmol) was added dropwise. The reaction stirred at room temperature for 2 h (TLC 6:4 cyclohexane/EtOAc), then the crude mixture was purified on silica gel affording the desired compound **71** (2.6 g, 2.6 mmol, 71% yield). $[\alpha]_D^{25}$ = +39.86° (c 1.7, $CHCl_3$). ESI HR-MS ($C_{53}H_{51}N_3O_{16}$) *m/z* $[M+Na]^+$ found 1008.3178; calcd 1008.3167.
^{1}H-NMR (400 MHz, $CDCl_3$) δ 8.11-7.91 (m, 10H, H-Ar) 7.66-7.26 (m, 15H, H-Ar), 5.71 (t, J = 9.4 Hz, 1H, H3^A), 5.39 (dd , $J_{1,2}$ = 7.9 Hz, $J_{2,3}$ = 9.7 Hz, 1H, H-2^A), 5.13 (t, J = 7.6 Hz, 1H, H2^B), 4.64-4.57 (m, 3H, H6a^A, H1^A, H1^B) 4.45 (dd, $J_{5,6a}$ = 4.6 Hz, $J_{6a,6b}$ = 12.1 Hz, 1H, H6b^A) 4.27-4.08 (m, 3H, H5^A, H3^B, H6a^B), 4.07 (dd, $J_{3,4}$ = 2.0, $J_{4,5}$ =5.5, 1H, H-4^A), 3.91-3.77 (m, 3H, H4^B, H5^B, OCH_{2a}), 3.66 (dd, $J_{5,6b}$ = 7.4 Hz, $J_{6a,6b}$ = 11.4 Hz, 1H, H6b^B), 3.53-3.43 (m, 1H, OCH_{2b}), 3.21-3.13 (m, 2H, CH_2N_3), 1.82-1.45 (m, 5H, $CH_2CH_2N_3$, CH_3), 1.26-1.23 (m, 3H, CH_3).
^{13}C-NMR (101 MHz, $CDCl_3$) δ 166.0, 165.9, 165.6, 165.2, 164.9 (5 x CO), 133.5-128.6 (C-Ar), 100.6 (C1^A, C1^B), 76.9 (C-3^B), 75.4 (C-5^A), 73.6 (C-2^B),73.0 (C-4^A), 72.6 (C-3^A, C-5^B), 71.9 (C-2^A), 71.7 (C-4^B), 66.8 (OCH_2), 62.8 (C-6^B), 62.5 (C-6^A), 47.9 (CH_2N_3), 29.2 ($CH_2CH_2N_3$), 27.4 (CH_3), 26.2 (CH_3).

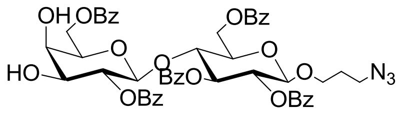

33

3-Azidopropyl 2,6-di-*O*-benzoyl-*β*-D-galactopyranosyl-(1→4)-2,3,6-tri-*O*-benzoyl-*β*-D-glucopyranoside 33. Lactose **71** (2.6 g, 2.6 mmol) was dissolved in 20.0 mL of a 4:1 AcOH/H_2O mixture and the resulting reaction mixture was heated to 90°C. After 2 h (TLC 8:2 Toluene/EtOAc) the solvent was coevaporated with toluene. The crude was purified by column chromatography affording compound **33** (1.6 g, 1.7 mmol, 70% yield). $[\alpha]_D^{25}$ = +37.04°(c 0.7, $CHCl_3$). ESI- HR MS **(**$C_{50}H_{47}N_3O_{16}$) *m/z* $[M+H]^+$ found 946.30, calcd 946.30.
^{1}H-NMR (400 MHz, $CDCl_3$) δ 8.11-7.29 (m, 25H, H-Ar), 5.69 (t, J = 9.4 Hz, 1H, H-3^A), 5.44-5.36 (dd, $J_{1,2}$ = 7.9 Hz, $J_{2,3}$ = 9.8 Hz, 1H, H-2^A), 5.30-5.23 (dd, $J_{1,2}$ = 7.9 Hz, $J_{3,4}$ = 9.7 Hz, 1H, H-2^B), 4.65-4.56 (m, 3H, H-1^A, H-1^B, H-6a^A), 4.54-4.48 (dd, $J_{5,6a}$ = 5.1 Hz, $J_{6a,6b}$ = 12.2 Hz, 1H, H-6b^A), 4.16-4.09 (t, J = 8.8 Hz, 1H, H-4^A), 4.06-3.99 (dd, $J_{5,6a}$ = 6.6 Hz, $J_{6a,6b}$ =11.3 Hz, 1H, H6$^B{}_a$), 3.90-3.76 (m, 3H, OCH_{2a}, H-5^A, H-4^B), 3.71-3.63 (d, J = 9.3 Hz, 1H, H-3^B), 3.61-3.43 (m, 3H, OCH_{2b}, H-5^B, H-6b^B), 3.29-3.11 (m, 2H, CH_2N_3), 1.85-1.59 (m, 2H, $CH_2CH_2N_3$).

^{13}C-NMR (101 MHz, $CDCl_3$) δ 166.5, 166.2, 166.0, 165.8, 165.2 (5 x CO), 133.4-128.5 (C-Ar), 101.0, 100.8 (C-1^A, C-1^B), 76.1 (C-4^A), 73.7 (C-2^B), 73.0, 72.9 (C-3^A, C-4^B), 72.5, 72.6 (C-3^B, C-5^B), 71.6 (C-2^A), 68.4 (C-5^A), 66.6 (O*C*H_2), 62.5 (C-6^A), 61.7 (C-6^B), 47.8 (*C*H_2N_3), 28.9 (CH_2*C*H_2N_3).

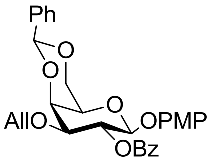

73

***p*-Metoxyphenyl 3-*O*-allyl-2-*O*-benzyl-4,6-*O*-benzylidene-*β*-D-galactopyranoside 73.** Compound **72** (1.0 g, 2.4 mmol) was dissolved in 20 mL of 1:4 pyridine and DCM mixture, then the resulting solution was cooled down to 0°C in a water/ice bath and BzCl (1.0 g, 7.2 mmol) was added dropwise. The reaction stirred at room temperature overnight, then the reaction was checked by TLC (4:6 cyclohexane:EtOAc), which showed full conversion of the starting material. The solvent was co-evaporated with toluene and purified by column chromatography (cyclohexane/EtOAc) affording **73** (500 mg, 40% yield). $[\alpha]_D^{25}$ = +2.69° (c 0.2, $CHCl_3$). ESI HR-MS ($C_{30}H_{30}O_8$) *m/z* $[M+H]^+$ found 519.20; calcd 519.20.

^{1}H-NMR (400 MHz, $CDCl_3$) δ 8.15-6.7 (m, 14H, H-Ar), 5.86-5.73 (m, 2H, H-2, C*H*=CH_2), 5.59 (s, 1H, C*H*Ph), 5.21 (dd, 1H, J = 1.5 Hz, J =17.1 Hz, CH_2=CH), 5.09 (dd, 1H, J = 1.3 Hz, J = 10.4 Hz, CH_2=CH), 5.05 (d, 1H, $J_{1,2}$ = 8.0 Hz, H-1), 4.44-4.33 (m, 2H, H-4, H-6a), 4.20-4.05 (m, 3H, CH_2=CH, H-6b), 3.81 (dd, $J_{3,4}$ = 3.5, $J_{2,3}$ = 10.1 Hz, 1H, H-3), 3.71 (s, 3H, OCH_3), 3.57 (s, 1H, H-5).

^{13}C-NMR (101 MHz, $CDCl_3$) δ 171.6 (CO), 165.1-126.5 (C-Ar), 119.34 (Cq), 117.5 (CH=*C*H_2), 114.33 (*C*H=CH_2), 101.53 (C-1), 101.31 (*C*H_2Ph), 77.10 (C-3), 73.58 (C-4), 70.81 (*C*H_2-CH=CH_2, C-2), 69.12 (C-6), 66.88 (C-5), 55.59 (O*C*H_3).

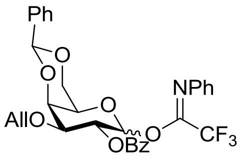

38

***p*-Metoxyphenyl 3-*O*-allyl-2-*O*-benzyl-4,6-*O*-benzylidene-*β*-D-galactopyranosyl trifluoroacetimidate 38.** Compound **73** (0.4 g, 0.8 mmol) was dissolved in 10 mL of a 4:1 mixture of acetonitrile and water and the resulting solution was cooled down to 0°C. Cerium ammonium nitrate (0.8 g, 1.5 mmol) was added and the resulting reaction mixture stirred for two hours at 0°C. After two hours analytical TLC (3:2 cyclohexane/EtOAc) showed full conversion of the starting material and formation of a new spot with lower R_f. The reaction mixture was poured into iced aqueous $NaHCO_3$ and extracted with DCM (5 x). The organic phase was dried over Na_2SO_4, filtered and evaporated under reduced pressure. The crude was purified by column chromatography (cyclohexane/EtOAc), affording the target compound **37** in 77% yield.

Then compound **37** (0.2 g, 0.5 mmol) and 2,2,2-trifluoro-*N*-phenyl-acetoimidoyl-chloride (0.4 g, 1.8 mmol) were dissolved in 30.0 mL of dry DCM under nitrogen atmosphere. The resulting solution was cooled down to 0°C and Cs_2CO_3 (0.2 mmol, 0.6 mmol) was added. The resulting reaction mixture

was stirred at room temperature for 2 hours, then analytical TLC (6:4 cyclohexane:EtOAc) showed complete conversion of the starting material and formation of a new spot with higher R_f. The reaction mixture was quenched with TEA and the solid was filtered off, then the reaction mixture was concentrated in vacuum and applied to a chromatograhy column (cyclohexane:EtOAc). Pure fractions were collected and evaporated under reduced pressure affording the trifluoroacetimidate **38** (0.260 g, 55%). $[\alpha]_D^{25}$ = +60.93° (c 1.65, $CHCl_3$). ESI HR-MS ($C_{31}H_{28}$ F_3NO_7) *m/z* $[M\text{-}CNPhCF_3\text{+}Na]^+$ found 435.1413; calcd 435.1414.

^{1}H-NMR (400 MHz, $CDCl_3$) δ 8.13-7.01 (m, 14H, H-Ar), 6.72 (s, 2H, H-1, H-Ar), 5.86-5.72 (m, 2H, H-2, C*H*=CH_2), 5.58 (s, 1H, C*H*Ph), 5.22 (dd, 1H, *J* = 1.2 Hz, *J* = 17.3 Hz, C*H*$_2$=CH), 5.12 (dd, 1H, *J* = 0.8 Hz, *J* = 9.3 Hz, C*H*$_2$=CH), 4.44-4.29 (m, 2H, H-4, H-6a), 4.21-4.04 (m, 3H, C*H*$_2$-CH=CH_2, H6b), 3.91-3.41 (m, 2H, H-3, H-5).

^{13}C-NMR (101 MHz, $CDCl_3$) δ 164.9 (C=O), 143.38-124.30, (C-Ar), 119.28 (*C*H=CH_2), 117.80 (CH=*C*H$_2$), 101.27 (*C*H$_2$Ph), 73.30 (C-4), 70.94 (*C*H$_2$-CH=CH_2), 69.93 (C-2), 68.79 (C-6), 67.69 (C-5).

2.4 Preparations of disaccharides

Procedure A for glycosylation with thioglycoside donors with NIS/TfOH.

Donor (0.11 mmol) and acceptor (0.1 mmol) with activated 4 Å molecular sieves (0.1 g) were added at the solution of dry DCM (5 mL) and stirred for 20 min under nitrogen. NIS (0.2 mmol) and TfOH (0.02 mmol) were added at –30°C. The reaction was stirred for 2 and then allowed to warm up to room temperature. Stirring was continued for 12 h, monitoring by TLC (Tol:EtOAc or cyclohexane:EtOAc). The reaction was stirred for 12 h monitoring by (Tol:EtOAc or cyclohexane:EtOAc). the reaction was quenched with TEA, the solid filter off and the solvent removed at reduced pressure. The crude was purified by flash chromatography (cyclohexane:EtOAc) to give the purified products.

Procedure B for glycosylation with thioglycoside donors with NIS/AgOTf.

A solution of donor (0.11 mmol) and acceptor (0.1 mmol) with activated 4 Å molecular sieves (0.1 g) in dry DCM (5 mL) was stirred for 20 min under nitrogen. NIS (0.2 mmol) and AgOTf (0.02 mmol) were added at –30°C. The reaction was stirred in the dark allowing to warm up to room temperature. After TLC (Tol/EtOAc or cyclohexane/EtOAc) showed complete reaction, the mixture was quenched with TEA, the solid filter off and the solvent removed at reduced pressure. The crude was purified by flash chromatography (cyclohexane:EtOAc) to give the purified products.

Procedure C for glycosylation with trichloroacetimidate/trifluoroacetimidate donors

A solution of donor (0.11 mmol) and acceptor (0.1 mmol) with activated 4 Å molecular sieves (0.1 g) in dry DCM (5 mL) was stirred for 20 min under nitrogen. TMSOTf (0.02 mmol) was added at –10°C. After 4 h (TLC; Tol/EtOAc or cyclohexane/EtOAc) the reaction was quenched with TEA, the solid filter off and the solvent removed at reduced pressure. The crude was purified by flash chromatography (Tol:EtOAc or cyclohexane:EtOAc) to afford the purified products.

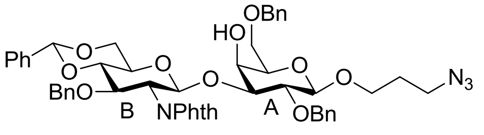

12a

3-Azidopropyl 4,6-*O*-benzilidene-3-*O*-benzyl-2-deoxy-2-phthalimido-*β*-D-glucopyranosyl-(1→3)-2,6-di-*O*-benzyl-*β*-D-galactopyranoside 12a.

Protocol A: **12a** not detected

Protocol B. After flash chromatography (cyclohexane/EtOAc) a 3:2 mixture (61% yield) of disaccharide **12a** and the β-(1→4) product, which could not be isolated as a clean compound, was obtained.

Protocol C **12a**, 31% yield. $[\alpha]_D^{25}$ = +14.39° (c 0.25, $CHCl_3$). ESI HR-MS ($C_{51}H_{52}N_4O_{12}$) *m/z* $[M+Na]^+$ found 935.3396; calcd 935.3479.

^{1}H NMR (400 MHz, $CDCl_3$) δ 7.55-6.87 (m, 24H, 24H-Ar) 5.65 (s, 1H, C*H*Ph), 5.48 (d, $J_{1,2}$ = 10.2 Hz, 1H, H-1^B), 4.81 (d, ^2J = 12.2 Hz, 1H, C*H*HPh), 4.59 (s, 2H, C*HH*Ph), 4.50 (d, ^2J = 12.2 Hz, 1H, CH*H*Ph), 4.45-4.43 (m, 1H, C*H*HPh), 4.34-4.32 (m, 2H, H-6a^B, H-2^B), 4.23-4.20 (m, 2H, CH*H*Ph, H-1^A), 4.05 (d, $J_{3,4}$ = 2.8 Hz, 1H, H-4^A), 3.89-3.78 (m, 4H, H-6b^B,OCH_{2a}, H-6a^A, H-5^B), 3.74-3.67 (m, 2H, H-6b^A, H-3^B), 3.60-3.57 (m, 3H, H-4^B, H-3^A, H-5^A), 3.48-3.40 (m, 2H, OCH_{2b}, H-2^A), 3.16 (dt, *J* = 3.2, 6.5 Hz, 2H, CH_2N_3), 1.70 (dt, *J* = 6.6, 13.3 Hz, 2H, C$H_2CH_2N_3$).

^{13}C NMR (101 MHz, $CDCl_3$) δ 129.13-123.28 (C-Ar), 103.34 (C-1^A), 101.40 (*C*HPh), 99.68 (C-1^B), 82.86, 82.79 (C-5^B), 77.59 (C-2^A), 74.44, 74.30 (*C*H_2Ph), 74.16 (*C*H_2Ph), 73.67 (*C*H_2Ph), 69.07 (C-6^A), 68.67 (C-6^B), 68.19 (C-4^A), 66.42 (O*C*H_2), 66.22 (C-3^B), 55.87 (C-2^B), 48.12 (*C*H_2N_3), 29.07 (*C*$H_2CH_2N_3$).

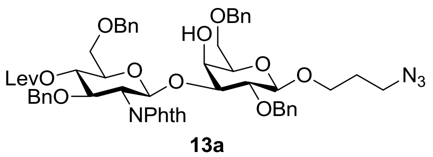

13a

3-Azidopropyl 3,6-*O*-benzyl-2-deoxy-4-*O*-levulinoyl-2-phthalimido-*β*-D-glucopyranosyl-(1→3)-2,6-di-*O*-benzyl-*β*-D-galactopyranoside 13a.

Protocol A. No reaction observed.

Protocol B. **13a** and **13b** were obtained in 40% and 28% yield, respectively.

Protocol C. **13a** was purified in 31% yield.

$[\alpha]_D^{25}$ = + 43.28 °(c 0.65, $CHCl_3$). ESI HR-MS ($C_{56}H_{60}N_4O_{14}$) *m/z* $[M+Na]^+$ found 1035.3871; calcd 1035.7878.

^{1}H NMR (400 MHz, $CDCl_3$) δ 7.51-6. 85 (m, 24H, H-Ar), 5.41 (d, $J_{1,2}$ = 8.3 Hz, 1H, H-1^B), 5.12 (t, J = 9.3 Hz, 1H, H-4^B), 4.66 (d, J= 12.4 Hz, 1H, C*H*HPh$_a$), 4.59-4.36 (m, 7H, 5 x C*H*HPh, H-3^B, H-2^B), 4.33 (d, ^2J = 12.4 Hz, 1H, CH*H*Ph), 4.23 (d, ^2J = 11.1 Hz, 1H, C*H*HPh), 4.20 (d, $J_{1,2}$ = 7.5 Hz, 1H, H-1^A), 4.08 (d, $J_{3,4}$ = 2.9 Hz, 1H, H-4^A), 3.90-3.80 (m, 1H, OCH_{2a}),3.73-3.42 (m, 9H, H-5^B, H-6a,b^B, H-6a,b^A, H-2,5,3^A, OCH_{2b}) 3.18 (t, J = 6.9 Hz, C$H_2$$N_3$), 2.71-2.68 (m, 2H, CH_2^{Lev}), 2.59-2.43 (m, 2H, CH_2^{Lev}), 2.18 (s, 3H, CH_3^{Lev}), 1.76-1.67 (m, 2H, C$H_2$$CH_2N_3$).

^{13}C NMR (101 MHz, $CDCl_3$) δ 206.2, 171.6, 128.42-123.29 (C-Ar), 103.15 (C-1^A), 98.59 (C-1^B), 83.55 (C-3^A), 77.5 (C-5^A), 76.9 (C-3^B), 74.47 (CH_2Ph), 74.10 (CH_2Ph), 74.16 (CH_2Ph), 73.57 (CH_2Ph), 73.45 (C-5^B), 73.18 (C-2^A), 72.45 (C-4^B), 69.66 (C-6^B), 69.46 (C-6^A), 67.84 (C-4^A), 66.32 (O*C*H_2), 55.42(C-2^B) , 48.17 (*C*H_2N_3), 37.70 (*C*H_2^{Lev}), 29.79 (*C*H_3^{Lev}), 29.10 (*C*$H_2CH_2N_3$), 27.88 (*C*H_2^{Lev}).

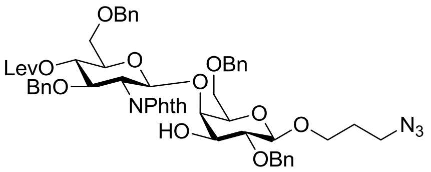

13b

3-Azidopropyl 3,6-*O*-benzyl-2-deoxy-4-*O*-levulinoyl-2-phthalimido-*β*-D-glucopyranosyl-(1→4)-2,6-di-*O*-benzyl-*β*-D-galactopyranoside 13b. $[\alpha]_D^{25}$ = + 18.87° (c 1.9, $CHCl_3$). ESI HR-MS ($C_{56}H_{60}N_4O_{14}$) *m/z* $[M+Na]^+$ found 1035.3914; calcd 1035.3878.

^{1}H NMR (400 MHz, $CDCl_3$) δ) δ 7.23-6.83 (m, 24H, H-Ar), 5.23 (d, $J_{1,2}$ = 8.4 Hz, 1H, H-1^B), 5.09 (t, J = 9.7 Hz, 1H, H-4^B), 4.59 (d, J = 9.5 Hz, 1H, C*H*HPh), 4.54-4.22 (m, 9H, 7 x each C*H*HPh, H-3^B, H-2^B), 4.07 (d, $J_{1,2}$ = 7.7 Hz,1H, H-1^A), 3.84 (d, $J_{3,4}$ = 2.7 Hz,1H, H-4^A), 3.80-3.42 (m, 8H, OCH_2, H-6a,b^A, H-6a,b^B, H-5^A, H-5^B), 3.32 (dd, $J_{2,3}$ = 9.7 Hz, 1H, H-3^A), 3.24 (t, J = 6.8 Hz, 2H, C$H_2$$N_3$), 2.87 (t, J = 8.6 Hz, 1H, H-2^A), 2.54-2.35 (m, 4 H, $CH_2CH_2^{Lev}$), 2.08 (s, 1H, CH_3^{Lev}), 1.80-1.67 (m, 2H, C$H_2$$CH_2N_3$).

^{13}C NMR (101 MHz, $CDCl_3$) δ 133.6-122.9 (C-Ar), 103.08 (C-1^A), 99.70(C-1^B), 79.89 (C-2^A), 76.8 (C-3^B), 76.6 (C-4^A), 73.79 (C-5^A), 73.46 (*C*H_2Ph) ,73.34 (2 x *C*H_2Ph), 72.86 (*C*H_2Ph), 72.70 (C-3^A), 72.64 (C-5^B), 69.84 (C-6$^{A/B}$), 69.71 (C-6$^{A/B}$), 66.02 (O*C*H_2), 55.68 (C-2^B), 48.35 (*C*H_2N_3), 37.74 (*C*H_2^{Lev}), 29.80 (*C*H_2^{Lev}), 29.17 (*C*$H_2CH_2N_3$), 27.95 (*C*H_3^{Lev}).

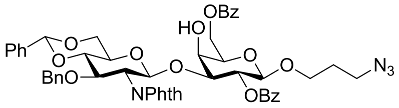

14a

3-Azidopropyl 4,6-*O*-benzilidene-3-*O*-benzyl-2-deoxy-2-phthalimido-*β*-D-glucopyranosyl-(1→3)-2,6-di-*O*-benzoyl-*β*-D-galactopyranoside 14a

Procotol B. **14a,** 53% yield.

Protocol C. **14a**, 77% yield.

$[\alpha]_D^{25}$ = + 44.98° (c 0.4, $CHCl_3$). ESI HR-MS ($C_{51}H_{48}N_4O_{14}$) *m/z* = $[M+Na]^+$ found 963.3200; calcd 963.3065.

^{1}H NMR (400 MHz, $CDCl_3$) δ 8.08-6.64 (m, 24H, H-Ar), 5.53 (s, 1H, C*H*Ph), 5.32 (d, $J_{1,2}$ = 8.2 Hz, 1H, H-1^B), 5.22 (t, *J* = 8.9 Hz,1H, H-2^A), 4.67-4.49 (m, 3H, H-6^A, C*H*HPh), 4.37-4.29 (m, 2H, CH*H*Ph, H-1^A), 4.29-4.22 (m, 2H, H-6a^B, H-3^B), 4.18 (dd, $J_{1,2}$ = 8.3 Hz, $J_{2,3}$ = 10.1 Hz, 1H, H-2^B), 4.11 (dd, 1H, $J_{3,4}$ = 2.8 Hz, H-4^A), 3.83-3.68 (m, 5H, H-5^A, H-3^A, H-4^B, H-6b^B, OCH_{2a}), 3.62-3.53 (m, 1H, H-5^B), 3.31 (dt, *J* = 4.3, 8.6 Hz, 1H, OCH_{2b}), 3.01-2.85 (m, 2H, C$H_2$$N_3$), 1.66-1.42 (m, 2H, C$H_2$$CH_2N_3$).

^{13}C NMR (101 MHz, $CDCl_3$) δ 166.4-164.5 (2 x C=O), 137.7-122.7 (C-Ar), 101.4 (*C*HPh), 101.2 (C-1^A), 99.9 (C-1^B), 82.7 (C-4^B), 80.8 (C-3^A), 74.2 (C-3^B), 74.0 (*C*H_2Ph), 71.9 (C-5^A), 70.5 (C-2^A), 68.6 (C-6^B), 68.5 (C-4^A), 66.3 (C-5^B), 65.9 (O*C*H_2), 63.5 (C-6^A), 55.5 (C-2^B), 47.8 (*C*H_2N_3), 28.9 (*C*$H_2CH_2N_3$).

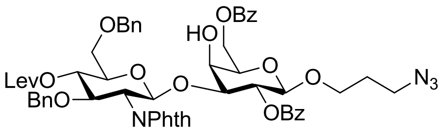

15a

3-Azidopropyl 3,6-*O*-benzyl-2-deoxy-4-*O*-levulinoyl-2-phthalimido-*β*-D-glucopyranosyl-(1→3)-2,6-di-*O*-benzoyl-*β*-D-galactopyranoside 15a.

Protocol A. No product formation.

Protocol B. **15a,** 65% yield.

Protocol C. **15a**, 33% yield.

$[\alpha]_D^{25}$ = + 62.78° (c 1.4, $CHCl_3$). ESI HR-MS ($C_{56}H_{56}N_4O_{16}$) *m/z* $[M+Na]^+$ found 1063.3577; calcd 1063.3589.

^{1}H NMR (400 MHz, $CDCl_3$) δ 8.05-6.84 (m, 24H, H-Ar), 5.36 (d, $J_{1,2}$ = 8.3 Hz, 1H, H-1^B), 5.31 (d, *J* = 8.9 Hz, 1H, H-4^B), 5.08 (t, *J* = 9.1 Hz, 1H, H-2^A), 4.61-4.44 (m, 5H, H-6^A, 3 x C*H*HPh), 4.39 (d, $J_{1,2}$ = 8.0 Hz, 1H, H-1^A), 4.36-4.34 (m, 1H, H-3^B), 4.31-4.29 (m, 1H, H-2^B), 4.27-4.24 (m, 2H, H-4^A, CH*H*Ph), 3.86-3.79 (m, 4H, OCH_{2a}, H-3,5^A, H-5^B), 3.60-3.58 (m, 2H, H-6^B), 3.41 (dt, *J* = 4.5, 9.2 Hz, 1H, OCH_{2b}), 3.20 (t, *J* = 6.5 Hz, 2H, C$H_2$$N_3$), 3.10-2.65 (m, 4H, *$CH_2CH_2$*^{Lev}), 2.16 (s, 3H, CH_3^{Lev}), 1.64-1.56 (m, 2H, C$H_2$$CH_2N_3$).

^{13}C NMR (101 MHz, $CDCl_3$) δ 166.8, 164.0 (C=O), 133.1-127.3 (C-Ar), 101.13 (C-1^A), 101.40, 98.82 (C-1^B), 81.05, 73.91, 73.49, 72.33, 72.04 (C-2^A), 70.49 (C-4^B), 69.52, 68.07, 65.84 (O*C*H_2),

63.73, 55.10 (C-2^{B}), 47.79 (CH_2N_3, CH_2^{Lev}), 37.68 (CH_2^{Lev}), 29.78 (CH_3^{Lev}), 28.86 ($CH_2CH_2N_3$), 27.84 (CH_2^{Lev}).

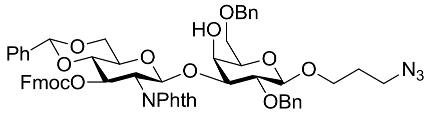

21a

3-Azidopropyl 4,6-*O*-benzylidene-2-deoxy-3-*O*-(9-fluorenylmethyloxycarbonyl)-2-phthalimido-*β*-D-glucopyranosyl-(1→3)-2,6-di-*O*-benzyl-*β*-D-galactopyranoside 21a.

Protocol A. After flash chromatography (Tol/EtOAc) **21a** and **21b** were purified in 30% and <5% yield, respectively.

Protocol B. **21a**, 38% yield; **21b**, 26% yield.

$[\alpha]_D^{25}$ = +10.37° (c 0.9, $CHCl_3$). ESI HR-MS ($C_{59}H_{56}N_4O_{14}$) *m/z* $[M+Na]^+$ found 1067.3629; calcd 1067.3691.

^{1}H NMR (400 MHz, $CDCl_3$) δ 7.63-6.85 (m, 27 H, H-Ar), 5.71 (t, J = 10.1 Hz, 1H, H-3^{B}), 5.62 (d, $J_{1,2}$ = 8.7 Hz, 1H, H-1^{B}), 5.51 (s, 1H, C*H*Ph), 4.51, 4.48 (2 d, ^2J = 12.3 Hz, 2H, 2 x C*HH*Ph), 4.47 (dd, $J_{2,3}$ = 10.4 Hz, 1H, H-2^{B}), 4.38 (d, ^2J = 11.7 Hz, 1H, C*H*HPh), 4.31 (dd, $J_{5a,6a}$ = 4.6 Hz, $J_{6a,6b}$ = 10.2 Hz, 1H, H-6^{A}), 4.17-4.13 (m, 2H, H-1^{A}, CH*H*Ph), 4.01-3.99 (m, 2H, H-4^{A}, CH_2^{Fmoc}), 3.87-3.81 (m, 2H, H-4^{B}, CH^{Fmoc}), 3.79-3.61 (m, 5H, H-6$^{B}_{a,b}$, H-6b^{A}, OCH_{2a}, H-5^{B}), 3.57 (dd, $J_{3,4}$ = 3.3 Hz, $J_{2,3}$ = 9.5 Hz, 1H, H-3^{A}), 3.51 (t, J = 6.0 Hz, 1H, H-2^{A}), 3.39 (dd, $J_{4,5}$ = 7.7 Hz, $J_{5,6a}$ = 9.2 Hz, 1H, H-5^{A}), 3.36-3.33 (m, 1H, OCH_{2b}), 3.06 (dt, J = 3.5, 6.8 Hz, 2H, CH_2N$_3$), 1.64-1.57 (m, 2H, CH_2CH$_2$N$_3$).

^{13}C NMR (101 MHz, $CDCl_3$) δ 134.04-119.88 (C-Ar), 103.42 (C-1^{A}), 101.83 (*C*HPh), 99.45 (C-1^{B}), 83.06 (C-3^{A}), 78.91 (C-4^{B}), 77.55 (C-5^{A}), 74.27 (*C*H$_2$Ph), 73.69 (C-3^{B}), 73.49 (*C*H$_2$Ph), 73.38, 72.73 (C-2^{A}), 70.36, 69.02 (C-6^{B}), 68.57 (C-6^{A}), 68.20 (C-4^{A}), 66.48 (O*C*H$_2$), 66.29 (C-5^{B}), 60.42, 55.25 (C-2^{B}), 48.36, 48.12 (CH_2N$_3$), 46.32 (*C*H^{Fmoc}), 29.08 (CH_2CH$_2$N$_3$), 28.25, 21.07, 14.21.

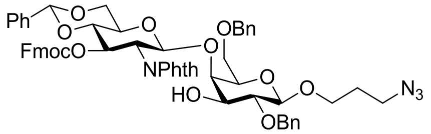

21b

3-Azidopropyl 4,6-*O*-benzylidene-2-deoxy-3-*O*-(9-fluorenylmethyloxycarbonyl)-2-phthalimido-*β*-D-glucopyranosyl-(1→4)-2,6-di-*O*-benzyl-*β*-D-galactopyranoside 21b. $[\alpha]_D^{25}$ = -8.99° (c 0.85, $CHCl_3$). ESI HR-MS ($C_{59}H_{56}N_4O_{14}$) *m/z* $[M+Na]^+$ found 1067.3680; calcd 1067.3691.

^{1}H NMR (400 MHz, $CDCl_3$) δ 7.70-6.94 (m, 27 H, H-Ar), 5.89 (t, J = 9.3Hz, 1H, H-3^{B}), 5.51 (s, 1H, C*H*Ph), 5.47 (d, $J_{1,2}$ = 7.8 Hz,1H, H-1^{B}), 4.55-4.48 (m, 3H, 2 CH*H*Ph, H-2^{B}), 4.16-4.08 (m, 3H, 2 CH*H*Ph, includ. d, 4.12, $J_{1,2}$ = 7.7 Hz, H-1^{A}), 3.95 (m, 2H, CH^{Fmoc}, H-4^{A}), 3.89-3.62 (m, 7H, includ. H-4,5,6^{B}, H-6^{A}, OCH_{2a}), 3.58 (m, 1H, OCH_{2b}), 3.47 (m, 1H, H-5^{A}), 3.36 (dd, $J_{3,4}$ = 2.9, $J_{2,3}$ = 7.2 Hz,

1H, H-3^A^), 3.30 (m, 2H, CH_2N_3), 3.00 (dd, $J_{1,2}$ = 7.7 Hz, $J_{2,3}$ = 9.8 Hz, 1H, H-2^A), 1.80 (m, 2H, C*H*$_2$CH$_2$N$_3$).

^{13}C NMR (101 MHz, $CDCl_3$) δ 134.0-119.9 (C-Ar), 103.27 (C-1^A), 101.7 (C*H*Ph), 100.4 (C-1^B), 79.9 (C-2^A), 79.1 (C-4^B), 77.2 (C-4^A), 74.9 (*C*H$_2$Ph), 73.6 (C-3^B), 73.4 (*C*H$_2$Ph), 72.8 (C-3^A), 70.2 (C-5$^{A/B}$), 68.8, 68.6 (C-6^{A,B}), 66.2 (O*C*H$_2$), 65.4 (C-5$^{A/B}$), 55.3 (C-2^B), 48.4 (*C*H$_2$N$_3$), 46.4 (*C*H^{Fmoc}), 29.2 (*C*H$_2$CH$_2$N$_3$).

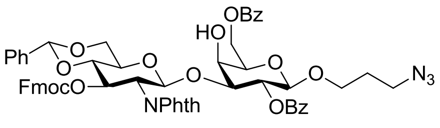

22a

3-Azidopropyl 4,6-*O*-benzylidene-2-deoxy-2-phthalimido-3-*O*-(9-fluorenylmethyloxycarbonyl)-*β*-D-glucopyranosyl-(1→3)-2,6-di-*O*-benzoyl-*β*-D-galactopyranoside 22a.

Protocol A. **22a**, 40% yield.

Protocol B. **22a**, 68% yield.

$[\alpha]_D^{25}$ = + 36.44° (c 0.65, $CHCl_3$). ESI HR-MS ($C_{59}H_{52}N_4O_{16}$) *m/z* $[M+Na]^+$ found 1095.3247; calcd 1095.3276.

^{1}H NMR (400 MHz, $CDCl_3$) δ 8.01-7.07 (m, 27 H, H-Ar), 5.62-5.57 (m, 1H, H-3^B), 5.56 (d, $J_{1,2}$ = 8.5 Hz, 1H, H-1^B), 5.50 (s, 1H, C*H*Ph), 5.27 (t, J = 9.1 Hz, 1H, H-2^A), 4.63 (dd, $J_{5,6a}$ = 11.0 Hz, $J_{6a,6b}$ = 5.0 Hz, 1H, H-6a^A), 4.55 (dd, $J_{5,6a}$ = 11.0 Hz, $J_{6a,6b}$ = 4.9 Hz, 1H, H-6b^A), 4.41 (t, J = 9.4 Hz, 1H, H-2^B), 4.34 (d, $J_{1,2}$ = 8.0 Hz, 1H, H-1^A), 4.31-4.27 (d, $J_{6a,6b}$ = 12.0 Hz, 1H, H-6a^B), 4.15 (d, $J_{3,4}$ = 3.2 Hz, 1H, H-4^A), 3.93 (d, J = 7.8 Hz, 2H, CH$_2^{Fmoc}$), 3.85-3.59 (m, 7H, H-3,5^A, H-4,5^B, H-6b^B, CH^{Fmoc}, OCH$_{2a}$), 3.36-3.30 (m, 1H, OCH$_{2b}$), 3.01-2.87 (m, 2H, C*H*$_2$N$_3$), 1.63-1.43 (m, 2H, C*H*$_2$CH$_2$N$_3$).

^{13}C NMR (101 MHz, $CDCl_3$) δ 166.4, 164.6, 154.3 (3 x C=O), 133.78-119.84 (C-Ar), 101.84 (*C*H$_2$Ph), 101.24 (C-1^A), 99.67 (C-1^B), 81.09 (C-4^B), 78.77 (C-3^A), 73.23, 71.88 (C-5^A), 70.48 (*C*H$_2^{Fmoc}$), 70.33 (C-2^B), 68.52 (C-4^A), 68.47 (C-6^B), 66.34 (C-5^B), 65.99 (O*C*H$_2$), 63.38 (C-6^A), 54.90 (C-2^B), 47.76 (*C*H$_2$N$_3$), 46.25 (*C*H^{Fmoc}), 28.86 (*C*H$_2$CH$_2$N$_3$).

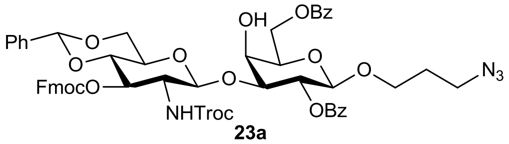

23a

3-Azidopropyl 4,6-*O*-benzylidene-2-deoxy-3-*O*-(9H-fluoren-9-ylmethylcarbonate)-2-(2,2,2-trichloroethoxycarbonylamino)-*β*-D-glucopyranosyl-(1→3)-2,6-di-*O*-benzoyl-*β*-D-galactopyranoside 23a.

Protocol B. **23a,** 65% yield.

Protocol C. **23a**, 70% yield.

$[\alpha]_D^{25}$ = -10.29° (c 0.55, $CHCl_3$). ESI HR-MS ($C_{54}H_{51}Cl_3N_4O_{16}$) *m/z* $[M+Na]^+$ found 1134.2173; calcd (1134.2138).

^{1}H NMR (400 MHz, $CDCl_3$) δ 8.17-7.10 (m, 23H, Ar-H), 5.56-5.48 (m, 2H, C*H*Ph, H-2^A), 5.24 (t, *J* = 10.0 Hz, 1H, H-3^B), 5.09 (d, $J_{N,H}$ = 8.3 Hz, 1H, NH), 4.99 (d, $J_{1,2}$ = 7.8 Hz, 1H, H-1^B), 4.73 (dd, *J* = 4.9, 11.4 Hz, 1H, C*H*HCCl$_3$), 4.65 (dd, 1H, CH*H*CCl$_3$), 4.55 (d, $J_{1,2}$ = 8.0 Hz, 1H, H-1^A), 4.37-4.25 (m, 4H, CH_2^{Fmoc}, H-6a^A, H-6a^B), 4.25-4.15 (m, 2H, CH^{Fmoc}, H-5^B), 4.09 (d, $J_{6a,6b}$ = 12.0 Hz, 1H, H-6b^A), 4.03-3.91 (m, 3H, incl. OCH_{2a}, H-3^A, H-4^A), 3.86-3.70 (m, 3H, H-2^B, H-6b^B, H-4^B), 3.69-3.48 (m, 2H, H-5^A ,OCH_{2b}), 3.26-3.13 (m, 2H, CH_2N_3), 2.05-1.51 (m, 2H, C*H*$_2$CH$_2$N$_3$).
^{13}C NMR (101 MHz, $CDCl_3$) δ 165.3, 155.0 (2 x CO), 143.0-120.1 (C-Ar), 101.6 (*C*HPh), 101.6 (C-1^A), 101.3 (C-1^B), 80.6 (C-4^A), 78.3 (C-4^B), 74.1 (C-3^B), 73.9 (C-6^A), 72.1 (C-3^A), 70.9 (C-2^A), 70.4 (*C*H$_2^{Fmoc}$), 68.7 (C-5^B), 68.4 (C-6^B), 66.4 (O*C*H$_2$), 63.4 (*C*H$_2$CCl$_3$), 57.2 (C-5^A), 47.9 (*C*H$_2$N$_3$), 46.5 (*C*H^{Fmoc}), 29.6 (*C*H$_2$CH$_2$N$_3$).

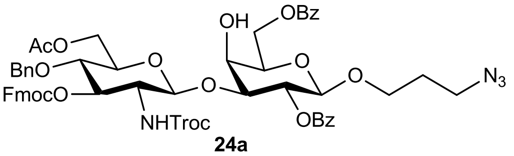

24a

3-Azidopropyl 6-*O*-acetyl-4-*O*-benzyl-2-deoxy-3-*O*-(9H-fluoren-9-ylmethyl carbonate)-2-(2,2,2-trichloroethoxycarbonylamino)-*β*-D-glucopyranosyl-(1→3)-2,6-di-*O*-benzoyl-*β*-D-galactopyranoside 24a.

Protocol C: **24a**, 50% yield.

$[\alpha]_D^{25}$ = +2.96° (c 0.6, $CHCl_3$). ESI HR-MS ($C_{56}H_{55}Cl_3N_4O_{17}$) *m/z* $[M+K]^+$ found 1199.2210; calcd 1199.2259.

^{1}H NMR (400 MHz, $CDCl_3$) δ 8.14-7.31 (m, 23H, Ar-H), 5.52 (dd, $J_{2,3}$ = 9.4 Hz, $J_{3,4}$ = 8.0 Hz, 1H, H-2^A), 5.19-5.08 (m, 2H, NH, H-3^B), 4.86 (d, $J_{1,2}$ = 8.3 Hz, 1H, H-1^B), 4.76-4.63 (m, 4H, C*H*$_2$Ph, C*H*HCl$_3$), 4.56 (d, $J_{1,2}$ = 8.0 Hz, 1H, H-1^A), 4.51 (d, *J* = 11.3 Hz, 1H, C*H*HCl$_3$), 4.44-4.30 (m, 4H), 4.24 (d, $J_{3,4}$ = 2.6 Hz, 1H, H-4^A), 4.18-4.12 (m, 2H), 4.04 (d, $J_{6a,6b}$ = 12.3 Hz, 1H, H-6), 4.00-3.90 (m, 3H), 3.66-3.51 (m, 4H), 3.25-3.17 (m, 2H, CH_2N_3), 2.06 (s, 3H, CH_3CO), 1.83-1.66 (m, 2H, C*H*$_2$CH$_2$N$_3$).

^{13}C NMR (101 MHz, $CDCl_3$) δ 133.4-120.2 (C-Ar), 101.1 (C-1^A), 100.6 (C-1 ^{B}), 81.4, 78.6 (C-2^A), 74.7, 73.9, 72.3, 70.8 (C-3^B), 70.3, 68.3 (C-4^A), 66.2, 63.5, 62.5, 62.0, 48.1 (OCH_2), 46.7 (CH^{Fmoc}), 29.2 (*C*H$_2$CH$_2$N$_3$), 21.6 ($COCH_3$).

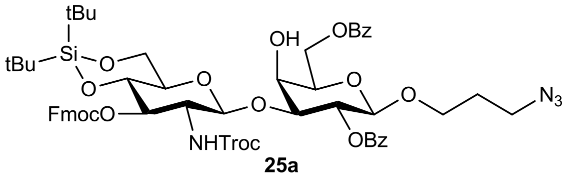

25a

3-Azidopropyl 4,6-*O*-di-*tert*-butylsilylidene-2-deoxy-3-*O*-(9H-fluoren-9-ylmethylcarbonate)-2-(2,2,2-trichloroethoxycarbonylamino)-*β*-D-glucopyranosyl-(1→3)-2,6-di-O-benzoyl-*β*-D-galactopyranoside 25a. A solution of donor **20** (0.330 g, 0.38 mmol) and acceptor **11** (150 mg, 0.316 mmol) with activated 4 Å molecular sieves (0.2 g) in dry DCM (5 mL) was stirred for 20 min under

nitrogen. TMSOTf (11.4 μL, 0.063 mmol) was added at 0°C. After 4 h (TLC 4:1 Tol/EtOAc) the reaction was quenched with TEA, the solid filtered off and the solvent removed at reduced pressure. The crude was purified by flash chromatography (4:1 Tol/EtOAc) to afford disaccharide **25a** (220 mg, 62% yield) as a pale solid.

$[\alpha]_D^{25}$ = -33.62° (c 0.2, $CHCl_3$). ESI HR-MS ($C_{55}H_{63}Cl_3N_4O_{16}Si$) *m/z* $[M+Na]^+$ found 1191.2927; calcd 1191.2966.

^1H NMR (400 MHz, $CDCl_3$) δ 8.12-7.28 (m, 18H, Ar-H), 5.49 (dd, $J_{1,2}$ = 9.6 Hz, $J_{2,3}$ = 8.1 Hz, 1H, H-2^A), 5.09-5.00 (m, 2H, H-3^B, NH), 4.99-4.81 (m, 2H, incl. H-1^B, C*H*HCCl$_3$), 4.73-4.56 (m, 3H, incl. CH_2^{Fmoc} CH*H*CCl$_3$), 4.53 (d, 1H, $J_{1,2}$ = 8.1 Hz, H-1^A), 4.41-4.19 (m, 4H, incl. CH^{Fmoc}, H-6a^B, H-6b^B), 4.17 (d, $J_{3,4}$ = 2.9 Hz, 1H, H-4^A), 4.13 (dd, 1H, $J_{5,6a}$ = 5.4 Hz, $J_{6a,6b}$ = 10.6 Hz, H-6), 4.06-3.81 (m, 5H, incl. H-3^A, CH_{2a}), 3.66-3.41 (m, 3H, incl. H-2^B, CH_{2b}), 3.31-3.15 (m, 2H, CH_2N_3), 1.86-1.62 (m, 2H, C*H*$_2$CH$_2$N$_3$), 1.03 (s, 9H, tBu), 0.92 (s, 9H, tBu).

^{13}C NMR (101 MHz, $CDCl_3$) δ 166.4, 165.2, 155.1, 154.6 (4 x CO), 143.4-120.1 (C-Ar), 101.3 (C-1^A), 101.1 (C-1^B), 100.0 (C_q), 80.3, 77.9, 77.2, 75.5, 75.1, 74.9, 73.8, 73.7, 72.0, 71.9, 70.6, 70.4, 68.6, 66.4, 66.2, 66.0, 63.4, 56.6 (C-2^B), 47.9 (O*C*H$_2$), 46.5 (*C*H^{Fmoc}), 29.0 (*C*H$_2$CH$_2$N$_3$), 27.3 (tBu), 26.8 (tBu), 22.7 (*C*(CH$_3$)$_3$), 19.9 (*C*(CH$_3$)$_3$).

2.5 Syntheses of trisaccharides

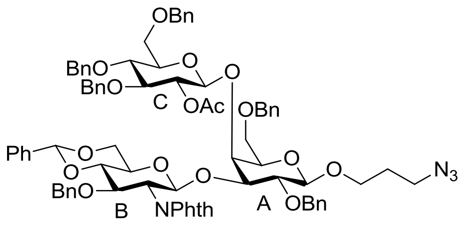

27a

3-Azidopropyl 2-*O*-acetyl-3,4,6-tri-*O*-benzyl-*β*-D-glucopyranosyl)-(1→4)-[3-*O*-benzyl-4,6-*O*-benzylidene-2-deoxy-2-phthalimido-β-D-glucopyranosyl-(1→3)]-2,6-di-*O*-benzyl-*β*-D-galactopyranoside 27a. A solution of donor **26** (50 mg, 0.08 mmol) and disaccharide acceptor **12a** (61 mg, 0.067) with activated 4 Å molecular sieves in dry DCM (4 mL) was stirred for 20 min under nitrogen. TMSOTf (3 μL, 0.013 mmol) was added at – 10°C. After 4 h (TLC 9:1 Tol/EtOAc) the reaction was quenched with TEA, the solid filter off and the solvent removed under pressure. The crude was purified by flash chromatography (Tol/EtOAc) to afford the thrisaccharide **27a** in 75% yield (68 mg) as a pale yellow solid. $[\alpha]_D^{25}$ = +24.86° (c 0.8, $CHCl_3$). ESI HR-MS ($C_{80}H_{82}N_4O_{18}$): *m/z* = $[M+ Na]^+$ found 1409.5419; calcd 1409.5522.

^1H NMR (400 MHz, $CDCl_3$) δ 7.55-6.85 (m, 39H, H-Ar), 5.66 (s, 1H, C*H*Ph), 5.56 (d, $J_{1,2}$ = 8.8Hz, 1H, H-1^B), 5.00-4.98 (m, 2H, H-1,2^C), 4.91-4.81 (m, 3H, 3 C*H*HPh), 4.60 (d, ^2J = 10.3 Hz, 1H, C*H*HPh), 4.53-4.36 (m, 8H, 7 C*H*HPh, H-6a^C), 4.28 (t, J = 9.2 Hz, 1H, H-2^B), 4.19 (d, $J_{1,2}$ = 7.6 Hz, 1H, H-1^A), 4.16 (d, $J_{3,4}$ = 2.3 Hz, 1H, H-4^A), 4.11 (d, ^2J = 11.5 Hz, 1H, C*H*HPh), 3.94-3.89 (m, 1H,

H-3^C), 3.86-3.49 (m, 13H, H-3^{A,B}, H-4^{B,C}, H-5^{A-C}, H-6^{A,B}, H-6b^C, OCH_{2a}), 3.42-3.37 (m, 1H, OCH_{2b}), 3.30 (t, J = 8.8 Hz, 1H, H-2^A), 3.13-3.10 (m, 2H, CH_2N_3), 1.79 (s, 3H, CH_3CO), 1.73-1.62 (m, 2H, $CH_2C\mathit{H_2}N_3$).

^{13}C NMR (101 MHz, $CDCl_3$) δ 169.9 (CO), 133.7-123.1 (C-Ar), 103.5 (C-1^A), 101.3 (Ph*C*H), 100.3 (C-1^C), 100.2 (C-1^B), 83.4 (C-3^C), 83.1, 81.2, 78.6 (C-2^A), 77.8, 75.2, 75.0, 74.9 (3 x Ph*C*H_2), 74.5, 74.4 (C-4^A), 74.1, 73.7, 73.5, 73.4, 73.1 (3 $PhCH_2$), 69.8, 69.1, 68.7 (C-6^{A-C}), 66.3 (O*C*H_2), 65.9, 56.3 (C-2^B), 48.2 (*C*H_2N_3), 29.1 (*C*$H_2CH_2N_3$), 20.8 (*C*H_3CO).

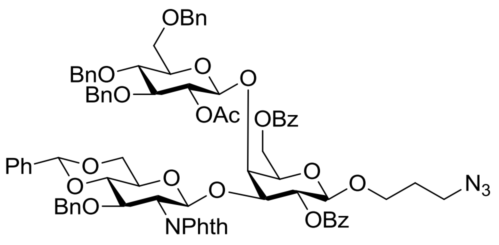

27b

3-Azidopropyl 2-*O*-acetyl-3,4,6-tri-*O*-benzyl-*β*-D-glucopyranosyl-(1→4)-[4,6-*O*-benzylidene-3-*O*-benzyl-2-deoxy-2-phthalimido-*β*-D-glucopyranosyl-(1→3)]-2,6-di-*O*-benzoyl-*β*-D-galactopyranoside 27b. A solution of donor **26** (48 mg, 0.077 mmol) and disaccharide acceptor **14a** (60m g, 0.064 mmol) with activated 4 Å molecular sieves (0.100 g) in dry DCM (4 mL) was stirred for 20 min under nitrogen. TMSOTf (2 µL, 0.013 mmol) was added at – 10°C. After 4 h (TLC 9:1 Tol/EtOAc) the reaction was quenched with TEA, the solid filter off and the solvent removed under pressure. The crude was purified by flash chromatography (Tol/EtOAc) to afford the thrisaccharide **27b** in 68% yield (58 mg) as a pale yellow solid. $[\alpha]_D^{25}$ = +18.18° (c 1.45, $CHCl_3$). ESI HR-MS ($C_{80}H_{78}N_4O_{20}$) *m/z* $[M+Na]^+$ found 1437.5027; calcd 1437.5107.

^{1}H NMR (400 MHz, $CDCl_3$) δ 8.08-6.80 (m, 39H, H-Ar), 5.66 (s, 1H, C*H*Ph), 5.43 (d, $J_{1,2}$ = 8.4 Hz, 1H, H-1^B), 5.15 (t, J = 8.3 Hz, 1H, H-2^A), 5.03 (t, J = 7.4 Hz, 1H, H-2^C), 4.99 (d, $J_{1,2}$ = 8.4 Hz, 1H, H-1^C), 4.91 (br. s, 2H, 2 C*H*HPh), 4.86 (d, ^2J = 10.7 Hz, C*H*HPh), 4.75 (d, ^2J = 10.7 Hz, C*H*HPh), 4.71 (d, $J_{5,6a}$ = 4.5 Hz, 12.3 Hz, 1H, H-6a^A), 4.62-4.37 (m, 6H, 4 C*H*HPh, H-6b^A, H-6a^B), 4.35 (d, J = 2.7 Hz, 1H, H-4^A), 4.32 (t, J = 8.5 Hz, 1H, H-3^B), 4.22 (t, J = 9.3 Hz, 1H, H-2^B), 3.94 (t, J = 9.5 Hz, 1H, H-3^C), 3.90-3.61 (m, 9H, H-3^A, H-4^{B,C}, H-5^{A,B}, H-6b^B, H-6^C, OCH_{2a}), 3.55-3.52 (m, 1H, H-5^C), 3.44-3.39 (m, 1H, OCH_{2b}), 3.08-2.96 (m, 2H, CH_2N_3), 2.02 (s, 3H, CH_3CO), 1.71-1.55 (m, 2H, C*H_2*CH_2N_3).

^{13}C NMR (101 MHz, $CDCl_3$) δ 171.2, 166.4, 164.7 (3 x CO), 133.5-122.9 (C-Ar), 101.3 (Ph*C*H), 101.1 (C-1^A), 100.4 (C-1^C), 100.3 (C-1^B), 83.4 (C-3^C), 83.0 (C-4^B), 80.0 (C-3^A), 78.0, 75.5 (C-5^C), 75.4, 75.3, 74.3 (3 x *C*H_2Ph), 74.2 (C-3^B), 74.0 (C-4^A), 73.5 (Ph*C*H_2), 73.3 (C-2^C), 72.2, 70.6 (C-2^A), 69.2, 68.7 (C-6^{A,B}), 66.1 (C-5^A), 65.3 (O*C*H_2), 64.4 (C-6^C), 55.9 (C-2^B), 47.9 (*C*H_2N_3), 28.9 (*C*$H_2CH_2N_3$), 20.7 (CH_3CO).

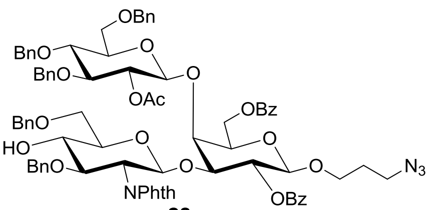

28

3-Azidopropyl 2-*O*-acetyl-3,4,6-tri-*O*-benzyl-*β*-D-glucopyranosyl-(1→4)-[3,6-di-*O*-benzyl-2-deoxy-2-phthalimido-β-D-glucopyranosyl-(1→3)]-2,6-di-*O*-benzoyl-*β*-D-galactopyranoside 28. A solution of trisaccharide **27b** (258 mg, 0.181 mmol) in acetonitrile was cooled down to 0°C. Trimethylamino borane complex (53 mg, 0.72 mmol) was added, followed by $BF_3{\cdot}Et_2O$ (0.090 mL, 0.72 mmol). The reaction mixture was stirred at 0°C for 3 h, when TLC (4:1 Tol/EtOAc) showed formation of a new spot with lower R_f. The reaction was quenched by addition of Et_3N and MeOH, then evaporated under vacuum. The crude was purified by column chromatography (Tol/EtOAc). Clean fractions were collected and evaporated to dryness affording trisaccharide **28** (0.180 g, 70% yield) as a colorless oil. $[\alpha]_D^{25}$ = + 204.32° (c 0.39, $CHCl_3$). ESI HR-MS ($C_{80}H_{80}N_4O_{20}$) *m/z* ([*M*+ Na]$^+$ found 1439.5051; calcd 1439.5264.

^{1}H NMR (400 MHz, $CDCl_3$) δ 8.15-6.54 (m, 39H, H-Ar), 5.29 (d, $J_{1,2}$ = 7.4 Hz, 1H, H-1^B), 5.08 (t, *J* = 9.1 Hz, 1H, H-2^A), 5.18-4.9 (m, 2H, incl. H-1^C, H-2^C), 4.81 (s, 2H, C*H*$_2$Ph), 4.76 (d, ^2J = 11.1, 1H, PhC*H*H), 4.66-4.56 (m, 2H, incl. PhC*H*H, H-6a^A), 4.56-4.33 (m, 7H, incl. H-6b^A), 4.32-4.26 (m, 2H, incl. H-1^A, H-4^A), 4.13-4.01 (m, 2H, incl. H-2^B, H-3^B), 3.87-3.66 (m, 6H, incl. H-3^C, H-3^A, H-5^A, H-4^B, H-6$^B_{a,b}$, OCH_{2a}), 3.66-3.52 (m, 4H, incl. H-4^C, H-5^B, H-6a,b^C), 3.49-3.40 (m, 1H, H-5^C), 3.40-3.29 (m, 1H, OCH_{2b}), 3.02-2.85 (m, 2H, CH_2N_3), 1.87 (s, 3H, CH_3CO), 1.66-1.42 (m, 2H, C*H*$_2CH_2N_3$).

^{13}C NMR (101 MHz, $CDCl_3$) δ 170.5, 166.4, 164.5 (3 x CO), 138.7-122.9 (C-Ar), 101.0 (C-1^A), 100.5 (C-1^C), 99.7 (C-1^B), 83.4 (C-3^C), 79.7 (C-3^A), 78.5 (C-3^B), 77.9 (C-4^C), 75.3 (C-5^C), 75.2, 75.0, 74.6 (3 x *C*H_2Ph), 74.3 (C-4^A), 74.2 (C-5^A), 73.7 (C-5^B), 73.7, 73.5 (2 x *C*H_2Ph), 73.1 (C-2^C), 72.3 (C-4^B), 70.7 (C-6^B), 70.6 (C-2^A), 69.2 (C-6^C), 65.1 (O*C*H_2), 64.8 (C-6^A), 55.5 (C-2^B), 47.9 (*C*H_2N_3), 28.9 (*C*$H_2CH_2N_3$), 20. 6 (*C*H_3CO).

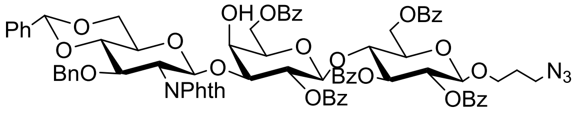

34

3-Azidopropyl 4,6-*O*-benzilidene-3-*O*-benzyl-2-deoxy-2-phthalimido-*β*-D-glucopyranosyl-(1→3)-2,6-di-*O*-benzoyl-*β*-D-galactopyranosyl-(1→4)-2,3,6-tri-*O*-benzoyl-*β*-D-glucopyranoside 34. A solution of donor **8** (450 mg, 0.7 mmol) and lactose acceptor **33** (518 mg, 0.548 mmol) with activated 4 Å molecular sieves (500 mg) in dry DCM (7 mL) was stirred for 20 min under nitrogen. TMSOTf (19 µL, 0.1 mmol) was added at –10°C. After 4 h (TLC: 9:1 DCM/EtOAc) the reaction was quenched with TEA, the solid filtered off and the solvent removed under pressure. The crude was purified by

flash chromatography (DCM/EtOAc) to afford the trisaccharide **34** in 68% yield. $[\alpha]_D^{25}$ = + 36.64° (c 0.1, $CHCl_3$). ESI HR-MS ($C_{78}H_{70}N_4O_{22}$) *m/z* $[M+H]^+$ found 1415.46; calcd 1415.45.

^{1}H-NMR (400 MHz, CDCl3) δ 8.14-6.72 (m, 39H, H-Ar), 5.62 (t, *J* = 9.5 Hz, 1H, H-3^A), 5.59 (s, 1H, C*H*Ph), 5.35 (dd, 1H, $J_{1,2}$ = 7.9 Hz, $J_{2,3}$ = 9.6 Hz, H-2^A), 5.31 (d, *J* = 7.5 Hz, 1H, H-1^C), 5.26 (dd, $J_{2,3}$ = 8.1 Hz, $J_{3,4}$ = 9.5 Hz, 1H, H-2^B), 4.70 (d, ^2J = 12.4, 1H, C*H*HPh), 4.55 (d, $J_{1,2}$ = 7.7 Hz, 1H, H-1^A), 4.45 (d, $J_{1,2}$ =8.0 Hz, 1H, H-1^B), 4.39 (d, *J* = 12.4 Hz, 1H, CH*H*Ph), 4.42-4.10 (m, 6H, incl. H-6a^A, H-6b^A, H-6a^B, H-6a^C, H-3^C, H-2^C), 4.05 (t, *J* = 9.5 Hz, 1H, H-4^A), 3.97 (d, *J* = 3.0 Hz, 1H, H-4^B), 3.85-3.72 (m, 3H, incl. H-5^A, H-6b^C, OCH_{2a}), 3.69 (dd, J = 3.3, 9.8 Hz, 1H, H-3^B), 3.66-3.55 (m, 3H, incl. H-4^C, H-6^{B}b, H-5^C), 3.55-3.49 (m, 1H, H-5^B), 3.49-3.41 (m, 1H, OCH_{2b}), 3.21-3.09 (m, 2H, CH_2N_3), 1.80-1.58 (m, 2H, C*H*$_2$$CH_2N_3$).

^{13}C-NMR (101 MHz, $CDCl_3$) δ 166.0, 165.8, 165.5, 165.2, 164.0 (5 x CO), 137.6-126.1 (m, C-Ar), 101.3 (CHPh), 101.0 (C-1^A), 100.6 (C-1^B), 99.8 (C1-^{C}), 82.6, 80.6, 75.3, 74.2, 74.0, 72.9, 72.5, 72.1, 71.7, 70.6, 68.5, 68.3, 66.5, 66.1, 62.6, 62.3, 55.5 (C-2^C), 47.8 (*C*H_2N_3), 28.9 (*C*$H_2CH_2N_3$).

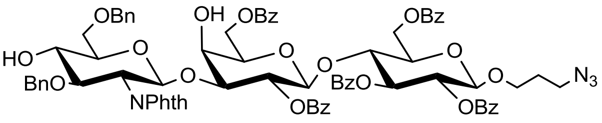

35

3-Azidopropyl 3,6-di-*O*-benzyl-2-deoxy-2-phtalimido-*β*-D-glucopyranosyl-(1→3)-[2,6-di-*O*-benzoyl-*β*-D-galactopyranosyl-(1→4)]-2,3,6-tri-*O*-benzoyl-*β*-D-glucopyranoside 35.

Trisaccharide **34** (100 mg, 0.071 mmol) was dissolved in 5.0 mL of acetonitrile and the resulting solution was cooled down to 0°C. Trimethylamino borane complex (26 mg, 0.355 mmol) was added, followed by $BF_3 \cdot Et_2O$ (43 µL, 0.355 mmol). The reaction mixture was stirred at 0°C for 3 h (TLC 4:1 Tol/EtOAc), then quenched by addition of Et_3N and MeOH, evaporated and purified by flash chromatography (Tol/EtOAc) affording trisaccharide **35** (65% yield). $[\alpha]_D^{25}$ = -4.99° (c 0.05, $CHCl_3$). ESI HR-MS ($C_{78}H_{72}N_4O_{22}$) *m/z* $[M+Na]^+$ found 1439.45; calcd 1439.45.

^{1}H-NMR (400 MHz, $CDCl_3$) δ 8.14-6.87 (m, 39H, H-Ar), 5.62 (t, *J* = 9.4 Hz, 1H, H-3^A), 5.34 (dd, $J_{1,2}$ = 7.8 Hz, 1H, H-2^A), 5.28-5.21 (m, 2H, H-2^B, H-1^C), 4.64-4.50 (m, 2H, H-1^A, C*H*$_2$Ph), 4.50-4.34 (m, 6H, H-6a^A, 2 x C*H*$_2$Ph, H-1^B), 4.22 (dd, ^2J = 4.7 Hz, 12.2 Hz, 1H, H-6b^A), 4.18-4.01 (m, 4H, incl. H-2^C, H-6a^B, H-4^A), 3.98 (d, 1H, $J_{3,4}$ = 3.3 Hz, H-4^B), 3.84-3.76 (m, 1H, OCH_{2a}), 3.75-3.40 (m, 9H, incl. H-3^{B,C}, H-5^B, H-6^C, H-6b^B, OCH_{2b}), 3.20-3.10 (m, 2H, CH_2N_3), 1.79-1.58 (m, 2H, C*H*$_2$$CH_2N_3$).

^{13}C-NMR (101 MHz, $CDCl_3$) δ 165.9, 165.8, 165.4, 165.2, 163.9, 137.8 (7 x CO), 133.4-125.3 (C-Ar), 101.0 (C-1^A), 100.0 (C-1^B), 98.9 (C-1^C), 80.9, 78.4, 75.2, 74.2 (*C*H_2Ph), 73.8 (*C*H_2Ph), 73.6, 73.5 (*C*H_2Ph), 72.9, 72.5 (C-3^A), 72.1, 71.7 (C-2^A), 70.6 (C-2^B), 70.2 (C-6^C), 67.8 (C-4^B), 66.5 (O*C*H_2), 62.8 (C-6^B), 62.3 (C-6^A), 54.9 (C-2^C), 47.8 (*C*H_2N_3), 29.7 (*C*$H_2CH_2N_3$).

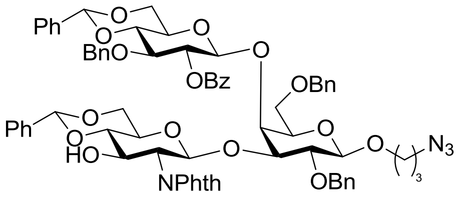

76

3-Azidopropyl 3-*O*-benzyl-4,6-O-benzylidene-2-*O*-benzoyl-*β*-D-glucopyranosyl-(1-4)-[4,6-*O*-benzylidene-2-deoxy-2-phthalimido-*β*-D-glucopyranosyl-(1-3)-]-2,6-di-*O*-benzoyl-*β*-D-galactopyranoside 76. Compound **74** (41 mg, 0.08 mmol) and **21a** (62 mg, 0.06 mmol) were dissolved in dry DCM (4 mL) with 4 Å activated molecular sieves and the mixture was stirred for 15 min under nitrogen. NIS (36 mg, 0.16 mmol) and TfOH (1.7 mg, 0.18 mmol) were added at -40°C, and the reaction was stirred overnight at rt, when TLC (7:3 Tol/EtOAc) showed complete reaction. The reaction was quenched with TEA, molecular sieves were filtered off and the solvent was removed at reduced pressure. The crude was purified by flash chromatography (Tol/EtOAc) to afford **75** (73 mg, 81% yield).

Trisaccharide **75** (73 mg, 0.05 mmol) was dissolved in dry DCM (4 mL) and 10% of piperidine (0.4 mL) were added at the solution. 10 minutes later, TLC (Tol/EtOAc) showed complete conversion, and the reaction was concentrated under reduced pressure. Purification of the crude material by flash chromatography (Tol/EtOAc) gave **76** (57 mg) in 90% yield. $[\alpha]_D^{25}$ = -7.89° (c 0.05, $CHCl_3$). ESI HR-MS ($C_{62}H_{59}N_4O_{17}$) *m/z* $[M+H]^+$ found 1267.3391; calcd 1267.3315.

^{1}H NMR (400 MHz, $CDCl_3$) δ 7.86-6.63 (m, 34 H, H-Ar), 5.55 (s, 1H, C*H*Ph), 5.52 (s, 1H, C*H*Ph), 5.43 (d, $J_{1,2}$ = 8.4 Hz, 1H, H-1^B), 5.40 (d, $J_{1,2}$ = 7.9 Hz, 1H, H-1^C), 5.24 (t, J = 7.9 Hz, 1H, H-2^C), 4.87 (d, ^2J = 12.7 Hz, 1H, C*H*HPh), 4.73 (d, ^2J = 12.7 Hz, 1H, CH*H*Ph), 4.57 (t, J = 9.4 Hz, 1H H-3^B), 4.47 (s, 2H, CH_2Ph), 4.31-4.21 (m, 5H, H-2^B, H-4^A, H-$6a^A$, CH_2Ph), 4.10 (t, J = 8.7 Hz, 1H, H-3^C), 4.02 (d, J = 7.7 Hz, 1H, H-1^A), 3.79 (t, J = 9.2 Hz, 1H, H-4^C), 3.72-3.51 (m, 10H, H-3^A, H-4^B, H-5^B, H-5^C, H-$6b^A$, H-6^B, H-6^C OCH_{2a}), 3.44 (dd, $J_{4,5}$ = 2.5, $J_{5,6a}$ = 8.8 Hz, 1H, H-5^A), 3.22-3.16 (m, 1H, OCH_{2b}), 2.98-2.86 (m, 3H, H-2^A, CH2N_3), 1.54-1.41 (m, 2H, C$H_2CH_2N_3$).

^{13}C NMR (101 MHz, $CDCl_3$) δ 165.0 (C=O), 134.1-123.4 (C-Ar), 103.3 (C-1^A), 102.02 (*C*HPh), 101.4 (*C*HPh), 100.4 (C-1^B), 99.9 (C-1^C), 82.1, 81.8, 78.9, 78.3, 74.2, 73.8, 73.7, 73.6, 72.8, 72.57, 69.0, 68.9, 68.6, 68.3, 66.4, 66.1, 65.7, 57.0 (C-2^C), 48.1 (*C*H_2N_3), 29.0 (*C*$H_2CH_2N_3$).

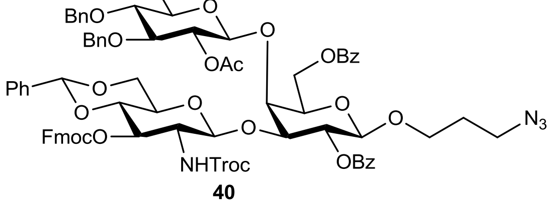

40

3-Azidopropyl 2-*O*-acetyl-3,4,6-tri-*O*-benzyl-*β*-D-glucopyranosyl-(1→4)-[4,6-*O*-benzylidene-3-*O*-(9H-fluoren-9-ylmethylcarbonate)-2-deoxy-2--(2,2,2-trichloroethoxycarbonyl]amino]-*β*-D-glucopyranosyl-(1→3)-2,6-di-*O*-benzoyl-*β*-D-galactopyranoside 40. A solution of trichloroacetoimidate donor **26** (50 mg, 0.078 mmol) and acceptor **23a** (0.073 g, 0.065 mmol) with 4 Å molecular sieves (100 mg) in dry DCM (5.0 mL) was

stirred for 20 min under nitrogen. TMSOTf (2.4 µL, 0.013) was added at -20°C. After 4 h (TLC 4:1 Tol/EtOAc) the reaction was quenched with TEA, the solid filtered off and the solvent removed under reduced pressure. The crude was purified by flash chromatography (Tol/EtOAc) to afford trisaccharide **40** in 63% yield (0.130 g). $[\alpha]_D^{25}$ = +16.32° (c 0.25, $CHCl_3$). ESI HR-MS ($C_{83}H_{81}Cl_3N_4O_{22}$) *m/z* $[M+Na]^+$ found 1613.4491; calcd 1613.4306.

^{1}H NMR (400 MHz, $CDCl_3$) δ 8.17-7.08 (m, 38H, H-Ar), 5.55 (s, 1H, C*H*Ph), 5.40 (dd, $J_{1,2}$ = 8.0 Hz, $J_{2,3}$ = 10.1, 1H, H-2^A), 5.34 (t, *J* = 10.1, 1H, H-2^C), 5.06 (d, $J_{1,2}$ = 8.1 Hz, 1H, H-1^B), 5.00 (d, *J* = 8.2 Hz, 1H, H-1^C), 4.96 (t, *J* = 8.7 Hz, 1H, H-2^C), 4.91-4.78 (m, 3H, CH_2Ph, CH*H*Ph), 4.74 (dd, 1H, *J* = 12.2 Hz, *J* = 4.2 Hz, C*H*HCCl$_3$), 4.62 (d, *J* = 10.7 Hz, 1H, C*H*HPh), 4.59-4.48 (m, 4H, incl. H-1^A, CH*H*CCl$_3$, CH_2Ph), 4.40-4.33 (m, 4H, incl. CH_2^{Fmoc}, H-4^A, H-6a^B), 4.33-4.26 (m, 1H, H-6a^A), 4.26-4.16 (m, 1H, CH^{Fmoc}), 4.01-3.8 (m, 5H, incl. H-3^C, H-3^A, H-6b^A, H-4^C, OCH_{2a}), 3.8-3.6 (m, 5H, incl. H-6b^B, 2H-6^C, H-4^B, H-5^A, OCH_{2a}), 3.62-3.40 (m, 5H, incl. H-2^B, OCH_{2b}, H-5^C, H-5^B), 3.30-3.08 (m, 2H, CH_2N_3), 2.24 (s, 3H, CH_3CO), 1.86-1.64 (m, 2H, C$H_2$$CH_2N_3$).

^{13}C NMR (101 MHz, $CDCl_3$) δ 170.5, 166.4, 164.9, 154.7, 153.5 (5 x CO), 143.2-120.0 (C-Ar), 102.1 (C-1^C), 101.6 (*C*HPh), 101.1 (C-1^A), 100.0 (C-1^B), 82.6 (C-3^C), 80.3 (C-3^A), 78.7 (C-5^A), 78.2 (C-4^B), 75.3 (C-5^B), 75.2, 74.9 (2 x *C*H_2Ph), 74.2 (C-4^A), 74.1 (C-3^B), 74.0 (C-4^A), 73.7 (C-2^C), 73.5 (*C*H_2Ph), 72.3 (C-4^C), 70.8 (C-2^A), 70.3 (*C*H_2^{Fmoc}), 69.2 (C-6^B), 68.4 (C-6^C), 66.2 (C-5^C), 65.6 (O*C*H_2), 64.5 (*C*H_2CCl_3), 57.5 (C-2^B), 48.0 (*C*H_2N_3), 46.5 (*C*H^{Fmoc}), 29.0 (CH_2*C*H_2N_3), 21.2 (*C*H_3 CO).

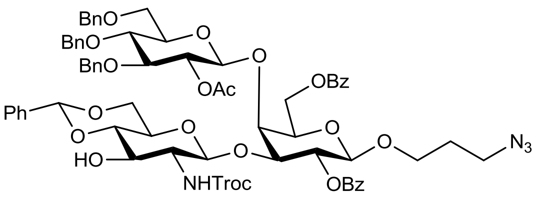

42

3-Azidopropyl 2-*O*-acetyl-3,4,6-tri-*O*-benzyl-*β*-D-glucopyranosyl-(1→4)-[4,6-*O*-benzylidene-2-deoxy-2-(2,2,2-trichloroethoxycarbonylamino-*β*-D-glucopyranosyl-(1→3)]-2,6-di-*O*-benzoyl-*β*-D-galactopyranoside 42. Trisaccharide **40** was (60 mg, 0.038 mmol) was dissolved in 2.0 mL of dry DCM and piperidine (0.2 mL) was added. After 1 h (TLC 4:1 Tol/EtOAc) the solvent was evaporated under reduced pressure and the crude was purified by flash chromatography (Tol/EtOAc) affording compound **42** (48 mg, 92% yield). $[\alpha]_D^{25}$ = -59.72° (c 0.155, $CHCl_3$). ESI HR-MS ($C_{68}H_{71}Cl_3N_4O_{20}$) *m/z* $[M+Na]^+$ found 1391.2691; calcd 1391.2695.

^{1}H NMR (400 MHz, $CDCl_3$) δ 8.12-7.01 (m, 30H, Ar-H), 5.54 (s, 1H, C*H*Ph), 5.35 (dd, $J_{1,2}$ = 8.0 Hz, $J_{2,3}$ = 10.1 Hz, 1H, H-2^A), 5.00-4.86 (m, 3H, incl. H-1^C, H-2^C, H-1^B), 4.85-4.67 (m, 4H, incl. CH_2Ph, C*H*HPh, C*H*HCCl$_3$), 4.61-4.43 (m, 5H, incl. H-1^A, 3 x CH*H*Ph, CH*H*CCl$_3$), 4.43-4.34 (m, 1H, H-6a^A), 4.34-4.26 (m, 2H, incl. H-4^A, H-6a^B), 4.15 (t, 1H, J=8.99 Hz, H-3^B), 3.95-3.81 (m, 4H, incl. H-3^C, H-3^A, H-4^C, OCH_{2a}), 3.75-3.60 (m, 5H, incl. H-5^C, H-6^C, H-6b^B, H-6b^A), 3.57-3.38 (m, 4H, incl.

H-5^{A}, H-4^{B}, H-5^{B}, OCH_{2b}), 3.25-3.07 (m, 3H, incl. H-2^{B}, CH_2N_3), 2.18 (s, 3H, CH_3CO), 1.80-1.57 (m, 2H, C*H*$_2$$CH_2N_3$).

^{13}C NMR (101 MHz, $CDCl_3$) δ 170.4, 166.4, 165.1 (3 x CO), 138.3-126.3 (C-Ar), 101.9 (*C*HPh), 101.8 (C-1^{B}), 101.1 (C-1^{A}), 100.3 (C1-^{C}), 82.6 (C-3^{C}), 81.4 (C-5^{A}), 80.0 (C-3^{A}), 78.1 (C-5^{C}), 75.3 (C-4^{B}), 75.2, 75.00 (2 x *C*H_2Ph), 74.6 (C-4^{A}), 74.0 (C-2^{C}), 73.8 (*C*H_2Ph), 73.5(C-6^{A}), 72.3 (C-4^{C}), 71.0 (C-2^{A}), 69.7 (C-3^{B}), 69.1 (C-6^{C}), 68.5 (C-6^{B}), 66.1 (C-5^{B}), 65.6 (O*C*H_2), 64.5 (*C*H_2CCl_3), 59.4 (C-2^{B}), 47.9 (*C*H_2N_3), 29.0 (*C*$H_2CH_2N_3$), 21.1 (CO*C*H_3).

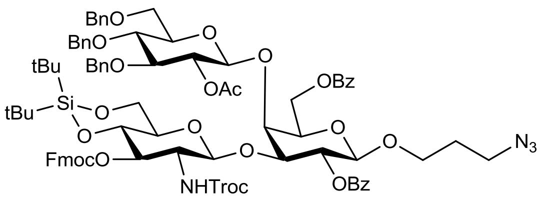

41

3-Azidopropyl 2-O-acetyl-3,4,6-tri-O-benzyl-β-D-glucopyranosyl-(1→4)-[4,6-*O*-di-*tert*-butylsilylidene-3-O-(9H-fluoren-9-ylmethylcarbonate)-2-deoxy-2-(2,2,2-trichloroethoxycarbonylamino]-*β*-D-glucopyranosyl-(1→3)]-2,6-di-*O*-benzoyl-*β*-D-galactopyranoside 41. A solution of acceptor **25a** (175 mg, 0.150 mmol) and donor **26** (190 mg, 0.300 mmol) in dry DCM (5.0 mL) with 4 Å molecular sieves was stirred for 20 min under nitrogen atmosphere. The resulting suspension was then cooled down to 0° C and TMSOTf (5.4 µL, 0.03 mmol) was added. The reaction was stirred for 2 h and left to slowly reach room temperature. After 2 h (TLC 9:1 Tol/EtOAc) the reaction was quenched by addition of TEA, then evaporated to dryness and the crude purified by medium pressure column chromatography using a gradient from 0 to 70% of EtOAc in toluene. Pure fractions were collected and evaporated under reduced pressure affording the target compound **41** (173 mg, 70% yield) as a clear syrup. $[\alpha]_D^{25}$ = -17.01° (c 0.4, $CHCl_3$). ESI HR-MS ($C_{84}H_{93}Cl_3N_4O_{22}Si$) *m/z* $[M+Na]^+$ found 1665.4995; calcd 1665.5009.

^{1}H NMR (400 MHz, $CDCl_3$) δ 8.08-7.28 (m, 33H, Ar-H); 5.36 (dt, $J_{2.3}$ = 10.0 Hz, $J_{3.4}$ = 7.9 Hz, 1H, H-3^{A}), 5.09 (t, 1H, J = 9.9 Hz, H-3^{B}), 5.01 (d, $J_{N,H}$ = 7.5 Hz, 1H, NH), 4.97-4.88 (m, 3H, H-1^{B}, H-1^{C}, H-2^{C}), 4.86-4.73 (m, 3H, CH_2CCl_3, C*H*HPh), 4.69 (dd, $J_{5,6a}$ = 4.2 Hz, $J_{6a,6b}$ = 12.1, Hz, 1H, H-6a^{A}), 4.62-4.37 (m, 7H, incl. H-6$^{A}_{b}$, CH_2Ph, H-1^{A}), 4.27 (d, $J_{3,4}$ = 2.1 Hz, 1H, H-4^{A}), 4.24-4.15 (m, 4H); 3.96-3.82 (m, 7H, incl. H-4^{B}, OCH_{2a}), 3.73-3.62 (m, 2H, incl. OCH_{2b}), 3.56-3.38 (m, 4H, incl. H-3^{C}, H-2^{B}), 3.23-3.08 (m, 2H, CH_2N_3), 2.18 (s, 3H, CH_3CO), 1.80-1.65 (m, 2H, C*H*$_2$$CH_2N_3$), 1.03 (s, 9H, tBu), 0.94 (s, 9H, tBu).

^{13}C NMR (101 MHz, $CDCl_3$) δ 170.5, 166.4, 164.9, 154.9, 153.5 (5 x CO), 143.3-120.1 (C-Ar), 101.1 (C-1^{B}, C-1^{C}), 100.2 (C-1^{A}), 82.5, 78.1, 75.5, 75.3, 75.2, 75.0, 74.9, 74.4 (C-4^{A}), 74.0, 73.7, 73.4, 72.3, 70.8 (C-3^{A}), 70.6, 70.3, 69.0, 66.2, 65.6 (O*C*H_2), 64.4 (C-6^{A}), 56.7 (C-2^{B}), 47.9 (*C*H_2N_3), 46.6 (*C*H^{Fmoc}), 29.0 (*C*$H_2CH_2N_3$) 27.4 (tBu), 26.9 (tBu), 22.6 (*C*H_3CO), 21.1 (*C*$(CH_3)_3$), 20.0 (*C*$(CH_3)_3$).

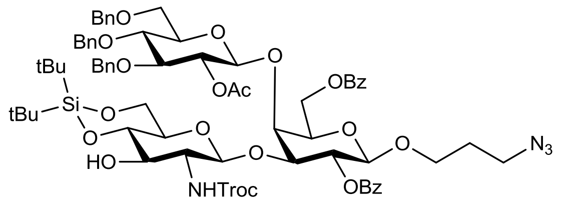

43

3-Azidopropyl 2-*O*-acetyl-3,4,6-tri-*O*-benzyl-β-D-glucopyranosyl-(1→4)-[4,6-*O*-di-*tert*-butylsilylidene-3-*O*-(9H-fluoren-9-ylmethyl carbonate)-2-deoxy-2-(2,2,2-trichloroethoxycarbonylamino]-*β*-D-glucopyranosyl-(1→3)-2,6-di-*O*-benzoyl-*β*-D-galactopyranoside 43. Compound **41** (173 mg, 0.105 mmol) was dissolved in 5.0 mL of DCM and 500 μL of piperidine were added. After 30 minutes, analytical TLC (2:1 Tol/EtOAc) showed full consumption of the starting material. The reaction was evaporated under reduced pressure and the crude was purified by column chromatography using a a gradient from 0 to 70% of EtOAc in toluene. Pure fractions were collected and evaporated to dryness, affording compound **43** as a pale solid (137 mg, 92% yield). $[\alpha]_D^{25}$ = -5.96 (c 1.25, $CHCl_3$). ESI HR-MS ($C_{69}H_{83}Cl_3N_4O_{20}Si$) *m/z* $[M+Na]^+$ found 1443.4331; calcd 1443.4328.

^{1}H NMR (400 MHz, $CDCl_3$) δ 8.08-7.11 (m, 25H, Ar-H), 5.33 (dd, $J_{2,3}$ = 7.9 Hz, $J_{3,4}$ = 9.7 Hz, 1H, H-3^A), 5.05 (d, $J_{N,H}$ = 7.4 Hz, 1H, NH), 4.92-4.80 (m, 2H, H-1^C, H-2^C), 4.82 (d, $J_{1,2}$ = 8.2 Hz, 1H,H-1^B), 4.80-4.68 (m, 3H, CH_2CCl_3, C*H*HPh), 4.65 (dd, $J_{5,6a}$ = 4.2 Hz, $J_{6a,6b}$ = 12.1, 1H, H-6$^A{}_a$), 4.57-4.39 (m, 6H, incl. H-1^A, H-6b^A, CH_2Ph), 4.20 (d, $J_{3,4}$ = 2.3 Hz, 1H, H-4^A), 4.14 (dd, 1H, J = 10.0, 4.9 Hz), 3.91-3.75 (m, 7H, incl. CH*H*N_3, H-3^A, H-3^C, H-3^B), 3.70-3.57 (m, 4H), 3.53-3.44 (m, 1H), 3.44-3.34 (m, 2H), 3.20-3.06 (m, 3H, H-2^B, CH_2N_3), 2.14 (s, 3H, CH_3CO), 1.78-1.59 (m, 2H, C*H*$_2CH_2N_3$), 1.04 (s, 9H, tBu), 0.95 (s, 9H, tBu).

^{13}C NMR (101 MHz, $CDCl_3$) δ 170.4, 166.4, 165.0, 153.9 (4 x CO), 138.3-127.5 (C-Ar), 101.5 (C-1^B), 101.1 (C-1^A), 100.4 (C-1^C), 82.6, 79.7, 78.1, 77.7, 75.2, 75.0, 74.9, 74.6 (C-4^A), 73.9 (C-2^C), 73.5, 72.8, 72.3, 71.0 (C-2A), 70.4, 69.0, 66.2 (O*C*H_2), 65.5 (C-6^B), 64.4 (C-6A), 58.3 (C-2^B), 48.0 (*C*H_2N_3), 29.0 (*C*$H_2CH_2N_3$), 27.5 (tBu), 27.0 (tBu), 22.7 (*C*$(CH_3)_3$), 21.1 (CH_3CO), 20.0 (*C*$(CH_3)_3$).

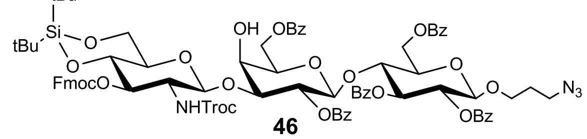

46

3-Azidopropyl 4,6-*O*-di-*tert*-butylsilylidene-3-*O*-deoxy--(9H-fluoren-9-ylmethylcarbonate)-2-(2,2,2-trichloroethoxyarbonylamino]-*β*-D-glucopyranosyl-(1→3)-2,6-di-*O*-benzoyl-*β*-D-galactopyranosyl-(1→4)-2,3,6-tri-*O*-benzoyl-*β*-D-glucopyranoside 46. A solution of donor **20** (0.337 g, 0.380 mmol) and acceptor **33** (200 mg, 0.211 mmol) in dry DCM (5.0 mL) in presence of 4 Å molecular sieves was stirred under nitrogen atmosphere for 20 minutes.

The reaction mixture was the cooled down to -10°C and TMSOTf (7.6 uL, 0.0422 mmol) was added dropwise. The resulting reaction mixture was stirred and allowed slowly to reach 5°C, when analytical TLC (9:1 Tol/EtOAc) showed formation of a new spot with intermediate. The reaction mixture was evaporated under reduced pressure and the crude was purified by medium pressure column chromatography using a gradient from 0 to 30% of EtOAc in toluene. Pure fractions were collected and evaporated under reduced pressure affording compound **46** as a pale solid (190 mg, 55% yield). $[\alpha]_D^{25}$ = -12.99° (c 0.15, $CHCl_3$). ESI HR-MS ($C_{82}H_{85}Cl_3N_4O_{24}Si$) *m/z* $[M+Na]^+$ found 1665.4262; calcd 1665.4281.

^{1}H NMR (400 MHz, $CDCl_3$) δ 8.14-7.20 (m, 33H, Ar-H), 5.68 (t, *J* = 9.2 Hz, 1H, H-3^A), 5.42-5.33 (m, 2H, H-2^A, H-2^B), 4.97-4.85 (m, 2H, incl. H-3^C), 4.77 (d, *J* = 7.8 Hz, 1H, H-1^C), 4.59 (d, $J_{1,2}$ = 7.9 Hz, 1H, H-1^B), 4.54 (d, $J_{1,2}$ =7.9 Hz, 1H, H-1^A), 4.51-4.45 (m, 1H), 4.42-4.24 (m, 3H), 4.22-4.07 (m, 4H, incl. H-4^A), 4.07-3.98 (m, 2H), 3.91-3.77 (m, 4H), 3.77-3.69 (m, 2H, incl. H-3^B), 3.64 (dd, *J* = 11.1, 7.1 Hz, 1H), 3.54-3.32 (m, 4H, incl. H-2^C), 3.21-3.08 (m, 2H, CH_2N_3), 1.79-1.56 (m, 2H, C$H_2$$CH_2N_3$), 0.97 (s, 9H, tBu), 0.87 (s, 9H, tBu).

^{13}C NMR (101 MHz, $CDCl_3$) δ 166.0, 165.5, 165.2, 164.6, 155.0 (5 x CO), 143.3-120.0 (C-Ar), 101.1 (C-1^C), 100.6 (C-1^B), 100.0 (C-1^A), 77.2, 75.5, 74.0, 73.8, 73.0, 72.6 (H-3^A), 72.2, 71.8, 71.0, 70.5, 70.3, 68.2, 66.6, 65.9, 62.5, 56.5 (H-2^C), 47.8 (*C*H_2N_3), 46.5 (CH^{Fmoc}), 28.9 (*C*$H_2CH_2N_3$), 27.3 (tBu), 26.8 (tBu), 22.6 (*C*$(CH_3)_3$), 19.9 (*C*$(CH_3)_3$).

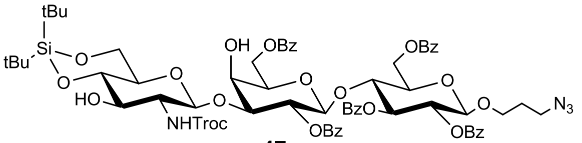

47

3-Azidopropyl 4,6-*O*-di-*tert*-butylsilylidene-2-deoxy-2-(2,2,2-trichloroethoxycarbonylamino)-β-D-glucopyranosyl-(1→3)-2,6-di-*O*-benzoyl-β-D-galactopyranosyl-(1→4)-2,3,6-tri-*O*-benzoyl-β-D-glucopyranoside 47. Compound **46** (140 mg, 0.085 mmol) was dissolved in 5.0 mL of DCM and 500 µL of piperidine were added. The resulting reaction mixture was stirred for 30 minutes at room temperature, when analytical TLC (85:15 Tol/EtOAc) showed full consumption of the starting material. The reaction mixture was evaporated under reduced pressure and the crude was purified by medium pressure column chromatography using a gradient from 0 to 80% of EtOAc in toluene. Pure fractions were collected and evaporated under reduced pressure affording compound **47** a pale solid (110 mg, 90% yield). $[\alpha]_D^{25}$ = -13.62° (c 0.2, $CHCl_3$). ESI HR-MS ($C_{82}H_{85}Cl_3N_4O_{24}Si$) *m/z* ([M+ Na]$^+$ found 1443.3568; calcd 1443.3600.

^{1}H NMR (400 MHz, $CDCl_3$) δ 8.08-7.21 (m, 25H, Ar-H); 5.68 (t, *J* = 9.3 Hz, 1H, H-3^A), 5.42-5.32 (m, 2H, H-2^A, H-2^B), 5.02 (bs, 1H, NH), 4.75 (d, 1H, $J_{1,2}$ = 7.3 Hz, H-1^C), 4.58 (d, $J_{1,2}$ = 7.8 Hz, 1H, H-1^B), 4.54 (d, $J_{1,2}$ = 7.90 Hz, 1H, H-1^A), 4.43-4.35 (m, 2H, CH*H*CCl_3), 4.17-4.06 (m, 2H, H-4^A),

4.03-3.94 (m, 2H), 3.91-3.66 (m, 6H, incl. H-3^{B,C}), 3.66-3.54 (m, 2H), 3.54-3.42 (m, 2H), 3.28 (dt, 1H, J = 9.7, 5.0 Hz), 3.20-3.09 (m, 3H, CH_2N_3, H-2^C), 1.79-1.62 (m, 2H, $CH_2CH_2N_3$), 0.99 (s, 9H, tBu), 0.90 (s, 9H, tBu).
^{13}C NMR (101 MHz, $CDCl_3$) δ 166.0, 165.9, 165.6, 165.3, 164.7 (5 x CO), 133.5-128.2 (C-Ar), 101.1 (C-1^B), 100.6 (C-1^C), 100.0 (C-1^A), 79.9, 77.3, 75.6, 73.9, 73.1, 72.6 (C-3A), 72.3, 71.8, 71.2, 70.3, 68.3, 66.6, 65.9, 62.5, 57.8 (C-2C), 47.8 (*C*H_2N_3), 28.9 (*C*$H_2CH_2N_3$), 27.4 (tBu), 26.9 (tBu), 22.6 (*C*$(CH_3)_3$), 19.9 (*C*$(CH_3)_3$).

2.6 Synthesis of GBS PSIa repeating units

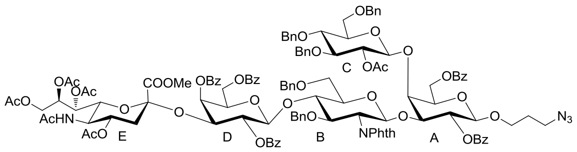

31

3-Azidopropyl 2-*O*-acetyl-3,4,6-tri-*O*-benzyl-β-D-glucopyranosyl-(1→4)-[2,4,6-tri-*O*-benzoyl-3-*O*-(methyl 4,7,8,9-tetra-*O*-acetyl-*N*-5-acetamido-3,5-dideoxy-D-glycero-α-D-galacto-non-2-ulopyranosylonate)-*β*-D-galactopyranosyl-(1→4)-3,6-di-*O*-benzyl-2-deoxy-2-phthalimido-*β*-D-glucopyranosyl-(1→3)]-2,6-di-*O*-benzoyl-*β*-D-galactopyranoside 31. A solution of disaccharide donor **29** (124 mg, 0.109 mmol) and acceptor **28** (110 mg, 0.078 mmol) with 4 Å molecular sieves (0.200 g) in dry DCM (5.0 mL) was stirred for 20 min under nitrogen. TMSOTf (2.8 μL, 0.0156 mmol) was added at -20°C. After 4 h (TLC 3:2 Tol/Acetone) the reaction was quenched with TEA, the solid filtered off and the solvent removed under reduced pressure. The crude was purified by flash chromatography (Tol/Acetone) to afford pentasaccharide **31** in 75% yield (138 mg). $[\alpha]_D^{25}$ = +41.05° (c 0.75, $CHCl_3$). ESI HR-MS ($C_{127}H_{129}N_5O_{40}$) *m/z* $[M+Na]^+$ found 2386.8462; calcd 2386.8106.
^{1}H NMR (400 MHz, $CDCl_3$) δ 8.39-6.56 (m, 54H, Ar-H), 5.77-5.70 (m, 1H, H-8^E), 5.51 (dd, $J_{1,2}$ = 7.8 Hz, $J_{2,3}$ =9.8 Hz, 1H, H-2^D), 5.34 (d, $J_{3,4}$ = 3.0 Hz, 1H, H-4^D), 5.26-5.19 (m, 2H, H-2^C, H-1^C), 5.12 (d, 1H, H-1^D), 5.09 (t, J = 9.8, 8.3 Hz, 1H, H-2^A), 4.97-4.72 (m, 7H, incl. H-1^B, C*H*HPh), 4.70-4.42 (m, 7H, C*H*HPh), 4.42-4.30 (m, 2H, C*H*HPh), 4.30-4.09 (m, 7H, incl. H-1^A, H-6^E), 4.08-3.92 (m, 2H), 3.92-3.55 (m, 14, incl. $COOCH_3$, H-5^E, H-2^B, OCH_{2a}), 3.54-3.32 (m, 3H, incl. OCH_{2b}), 3.14-2.88 (m, 2H, CH_2N_3), 2.51-2.41 (dd, 1H, $J_{3e,4}$ = 12.7 Hz, $J_{3a,3e}$ = 4.3 Hz, H-3e^E), 2.13, 2.01, 1.92, 1.87, 1.79 (5 x s, 3H each, 5 x CH_3CO), 1.73-1.57 (m, 3H, $CH_2CH_2N_3$, H-3a^E) , 1.50 (s, 3H, CH_3CO).
^{13}C NMR (101 MHz, $CDCl_3$) δ 170.8, 170.7, 170.5, 170.4, 170.3, 170.2, 168.2, 167.6, 167.0, 165.6, 165.5, 165.0, 164.5 (14 x CO), 138.1-122.8 (C-Ar), 100.9, 100.7, 100.4, 99.4, 97.0, 83.4, 79.5, 77.8, 75.2, 75.1, 74.9, 74.7, 74.6, 74.4, 73.5, 73.4, 73.1, 72.7, 72.3, 72.0, 71.9, 71.8 (C-2^A), 71.6, 71.5, 71.3, 70.6, 70.6, 69.4, 68.9, 68.7, 68.1 (C-4^D), 67.5, 67.1 (C-8^E), 66.7 (C-2^D), 65.0, 64.8, 62.5, 61.6, 55.9,

53.2, 48.8, 47.9 (CH_2N_3), 37.4 (C-3^E), 28.9 ($CH_2CH_2N_3$), 21.5, 21.3, 20.8, 20.7, 20.6, 20.4 (6 x $COCH_3$).

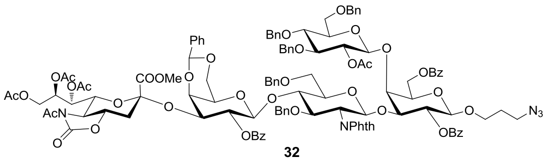

32

3-Azidopropyl 2-*O*-acetyl-3,4,6-tri-O-benzyl-*β*-D-glucopyranosyl-(1→4)-[4,6-*O*-benzylidene-2-*O*-benzoyl-3-*O*-(methyl 7,8,9-tri-*O*-acetyl-5-*N*-acetamido,4-*O*-oxazolidinone-3,5-dideoxy-D-glycero-*α*-D-galacto-non-2-ulopyranosylonate)-*β*-D-galactopyranosyl-(1→4)-3,6-di-*O*-benzyl-2-deoxy-2-phthalimido-*β*-D-glucopyranosyl-(1→3)]-2,6-di-*O*-benzoyl-*β*-D-galactopyranoside 32. A solution of donor **30** (60 mg, 0.063 mmol) and acceptor **28** (60 mg, 0.042 mmol) with 4 Å molecular sieves in dry DCM (3.0 mL) was stirred for 20 min under nitrogen. *N*-Iodosuccinimide (0.019 g, 0.084 mmol) and TfOH (0.7 µL, 0.0084 mmol) were added at -40°C. After 3 h (TLC 3:2 Tol/Acetone) the reaction was quenched with TEA, the solid was filtered off and the solvent removed under reduced pressure. The crude was purified by flash chromatography (Tol/Acetone) to afford pentasaccharide **32** in 73% yield (68 mg). $[\alpha]_D^{25}$ = +0.70° (c 0.25, $CHCl_3$). ESI HR-MS ($C_{119}H_{121}N_5O_{38}$) *m/z* $[M+K]^+$ found 2267.7361; calcd 2266.7321.

^{1}H NMR (400 MHz, $CDCl_3$) δ 8.17-6.57 (m, 49H, Ar-H), 5.61-5.55 (m, 2H), 5.52 (dd, $J_{1,2}$ = 7.7 Hz, $J_{2,3}$ = 9.8, Hz, 1H, H-2^D), 5.33 (s, 1H, C*H*Ph), 5.21 (d, $J_{1,2}$ = 7.2 Hz, 1H, H-1^B), 5.05 (dd, $J_{1,2}$ = 8.1 Hz, $J_{2,3}$ = 9.9, Hz, 1H, H-2^A), 5.02-4.95 (m, 2H, incl. H-2^C), 4.91 (d, $J_{1,2}$ = 7.9 Hz, 1H, H-1^C), 4.91 (d, $J_{1,2}$ = 7.9 Hz, 1H, H-1^D), 4.83 (s, 2H, CH_2Ph), 4.78 (d, ^2J = 10.9 Hz, 1H, C*H*HPh), 4.58-4.51 (m, 2H), 4.50-4.42 (m, 6H), 4.42-4.29 (m, 4H, incl. H-1^A), 4.28-4.23 (m, 2H, incl. H-2^B), 4.21-3.95 (m, 7H), 3.90-3.61 (m, 9H), 3.58-3.31 (m, 10H), 3.05-2.85 (m, 3H, H-3e^E, OCH_2), 2.46 (s, 3H, $NCOCH_3$), 2.18, 1,99, 1,90, 1.83 (5 x s, 3H each, 5 x CH_3CO), 1.79, (t, $J_{3a,3e}$ = 6.2 Hz, 1H, H-3a^E), 1.63-1.46 (m, 2H, CH_2CH_2N$_3$).

^{13}C NMR (101 MHz, $CDCl_3$) δ 172.1, 170.9, 170.5, 170.3, 170.0, 168.4, 167.5, 166.9, 166.2, 164.9, 164.4, 153.5 (12 x CO), 137.7-122.7 (C-Ar), 100.9 (*C*HPh), 100.9 (C-1^B), 100.7 (C-1^C), 100.4 (C-1^A), 99.6 (C-1^D), 97.2, 83.4, 79.9, 77.9, 77.8, 77.2, 75.3, 75.2, 75.1, 75.0, 74.9, 74.9, 74.6, 74.4, 73.5, 73.0, 72.9, 72.8, 72.2, 71.4, 71.1, 70.4, 68.9, 68.8,68.7, 67.9, 66.0, 64.9, 63.7, 58.9, 55.9, 52.9 ($COOCH_3$), 47.9 (CH_2N3), 37.0 (C-3^E), 28.9 ($CH_2CH_2N_3$), 24.7, 21.4, 20.8, 20.7, 20.6 (5 x CH_3CO).

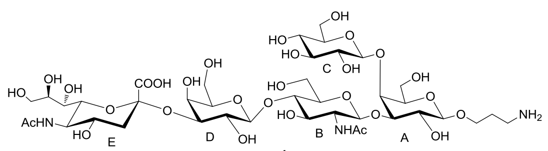

1

2-Aminopropyl *β*-D-glucopyranosyl-(1→4)-[3-*O*-(5-*N*-acetamido-3,5-dideoxy-D-glycero-*α*-D-galacto-2-nonulopyranosyl)-*β*-D-galactopyranosyl-(1→4)-2-

acetamido-2-deoxy-*β*-D-glucopyranosyl-(1→3)]-*β*-D-galactopyranoside 1.

Protocol A: A mixture of protected pentasaccharide **31** (0.1 mmol) and LiI (3 mmol) in pyridine (5 mL) was heated for 24h at 120°C. The reaction mixture was concentrated under vacuum, and the residue was purified by silica gel column chromatography (gradient 2% MeOH in DCM) to afford the demethylated product. This material was dissolved in ethanol (4 mL), and ethylenediamine (400 μL) was added. After being stirred for 16 h at 90 °C, the reaction mixture was then concentrated in vacuum, and the residue was coevaporated from toluene (2 x 10 mL) and EtOH (2 x 5 mL). The crude mixture was re-dissolved in pyridine (5 mL), and acetic anhydride (5 mL) was added. After being stirred for 16 h at room temperature, the reaction mixture was concentrated under reduced pressure. The residue was dissolved in MeOH and MeONa was added until pH=13. After 48 h the reaction was neutralized and the solvent removed under vacuum. The residue was dissolved in tBuOH and Pd/C (1:1 w/w in respect to the sugar) was added. The reaction mixture was stirred under pressure of H_2 (5 bar) for 72 h. Then, the catalyst was filtered off and the filtrate concentrated under reduced pressure. The reaction mixture was purified by G-10 size-exclusion column chromatography using water for elution. Fractions containing the sugar were quantified by sialic acid assay and freeze-dried to afford the deprotected oligosaccharide **1** as an amorphous powder (40% yield).

Protocol B: Protected pentasaccharide **32** (0.03 mmol) was dissolved in THF (3 mL) to which 3 M NaOH (0.3 mL) was added. After refluxing for 2 days, the mixture was neutralized with 0.1% HCl and concentrated. The residue was re-dissolved in 2:3 Ac_2O/MeOH (5 mL) and stirred overnight, when C18-TLC (2:3 H_2O/MeOH) showed disappearance of the starting material. After concentration, the residue was dissolved in tBuOH and Pd/C (1:1 w/w in respect to the sugar) was added. The reaction mixture was stirred under pressure of H_2 (5 bar) for 72 h. Then, the catalyst was filtered off and the filtrate concentrated under reduced pressure. The reaction mixture was purified by G-10 size-exclusion column chromatography using water for elution. Fractions containing the sugar were freeze-dried to afford the deprotected oligosaccharide **1** as an amorphous powder (45% yield).

$[\alpha]_D^{25}$ = +1.24° (c 0.2, H_2O). ESI HR-MS ($C_{40}H_{69}N_3O_{29}$) *m/z* $[M-H]^-$ found 1054.3866; calcd 1054.3971.

^{1}H NMR (400 MHz, D_2O) δ 4.88 (d, $J_{1,2}$ = 7.7 Hz, 1H, H-1^C), 4.69 (d, 1H, $J_{1,2}$ = 8.4 Hz, H-1^B), 4.54 (d, $J_{1,2}$ = 8.4 Hz, 1H, H-1^D), 4.39 (d, $J_{1,2}$ = 8.1 Hz, 1H, H-1^A), 4.37-4.35 (m, 1H), 4.09 (dd, $J_{3,4}$ = 2.9 Hz, $J_{2,3}$ = 9.7 Hz, 1H, H-3^A), 4.02-3.47 (m, 30H), 3.47-3.32 (m, 2H), 3.26 (dd, 1H, $J_{2,3}$ =9.4 Hz, H-2^C), 3.13 (dt, J = 8.2, 6.7 Hz, 1H), 2.74 (dd, $J_{3e,4}$ = 12.3 Hz, $J_{3e,3a}$ = 4.6, Hz, 1H, H-3e^E), 2.02 (s, 3H, $NCOCH_3$), 2.01 (s, 3H, CH_3CO), 2.04-1.93 (m, 2H, C$H_2CH_2NH_2$), 1.78 (t, 1H, H-3a^E).

^{13}C-NMR (101 MHz, $CDCl_3$) δ 175.0, 174.8, 173.8 (3 x CO), 103.1 (C-1^A), 102.9 (C-1^D), 102.5 (C-1^C), 102.0 (C-1^B), 82.1, 78.1, 75.8, 75.6, 75.5, 75.2, 74.8, 74.6, 74.2, 73.6, 72.8, 72.1, 71.7, 70.0, 69.7,

69.3, 68.3, 68.0, 67.9, 67.4, 62.5, 61.0, 60.6, 60.1, 55.3, 51.6, 39.6 (CH_2N_3), 37.6 (C-3^E), 26.7 ($CH_2CH_2N_3$), 22.2, 22.0 (2 x CH_3CO).

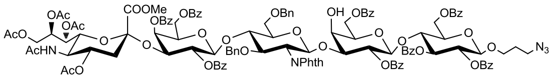

36

3-Azidopropyl 2,4,6-tri-*O*-benzoyl-3-*O*-(methyl-4,7,8,9-tetra-*O*-acetyl-5-*N*-acetamido-3,5-dideoxy-D-glycero-*α*-D-galacto-non-2-ulopyranosylonate)-*β*-D-galactopyranosyl-(1→4)-3,6-di-*O*-benzyl-2-deoxy-2-phthalimido-*β*-D-glucopyranoside-(1→3)-2,6-di-*O*-benzoyl-*β*-D-galactopyranosyl-(1→4)-2,3,6-tri-*O*-benzoyl-*β*-D-glucopyranoside 36. A solution of trisaccharide acceptor **35** (103 mg, 0.073 mmol) and disaccharide donor **29** (124 mg, 0.117 mmol) with activated 4 A molecular sieves (100 mg) in DCM (5 mL) was stirred for 20 min under nitrogen. TMSOTf (2.65 µL, 0.015 mmol) was added at 0 °C. After the reaction mixture was stirred for 2 h at rt, when TLC (3:2 Tol/Acetone) showed complete reaction. TEA was added until neutral pH, the solid filtered off and the solvent removed at reduced pressure. The crude was purified by flash chromatography (Tol/Acetone) to afford **36** (112 mg, 65% yield). $[\alpha]_D^{25}$ = +23.9° (c 0.1, $CHCl_3$). ESI HR-MS *m/z* ($C_{125}H_{121}N_5O_{42}$) *m/z* $[M+Na]^+$ found 2387.34, calcd 2387.73.

^{1}H-NMR (400 MHz, $CDCl_3$) δ 8.29-6.55 (m, 54H, H-Ar), 5.73-5.66 (m, 1H, H-8^E), 5.60-5.53 (m, 1H, H-3^A), 5.47 (dd, 1H, $J_{1,2}$ = 7.8 Hz, $J_{3,4}$= 9.9 Hz, H-2^D), 5.31-5.25 (m, 2H, H-2^A, H-7^E), 5.22-5.15 (m, 2H, H-2^B, H-4^D), 5.09 (d, $J_{1,2}$ = 7.5, 1H, H-1^D), 5.05 (t, $J_{1,2}$ = 7.5, 1H, H-1^C), 4.87-4.77 (m, 3H, incl. CH_2Ph, H-3^D), 4.51-4.42 (m, 3H, incl. CH_2Ph, H-1^A), 4.37-4.28 (m, 4H, H-1^B), 4.19-43.93 (m, 11H, include. H-2^C, H-4^B, H-4^A), 3.85-3.74 (m, 6H, include. $COOCH_3$, H-5^E), 3.61-3.53 (m, 4H), 3.49-3.45 (dd, 1H, $J_{3,4}$ = 3.3 Hz, $J_{2,3}$ = 9.6 Hz, H-3^B), 3.44-3.29 (m, 3H), 3.15-3.05 (m, 2H, CH_2N_3), 2.45-2.39 (m, 1H, H-3$_{eq}^E$), 2.14 (s, 3H, CH_3CO), 2.06-2.03 (m, 3H, CH_3CO), 1.97 (s, 3H, CH_3CO), 1.89 (s, 3H, CH_3CO), 1.76 (s, 3H, CH_3CO), 1.68-1.58 (m, 3H, C$H_2CH_2N_3$), 1.50 (s, 3H, CH_3CO).

^{13}C-NMR (101 MHz, $CDCl_3$) δ 170.78-163.87 (CO), 138.17-125.31 (C–Ar), 101.01 (C-1^A), 100.48 (C-1^B), 100.37 (C-1^C), 98.75 (C-1^D), 96.94, 80.99 (C-3^B),75.11, (C-4^A), 74.44, 74.34 (CH_2Ph), 72.98, 72.78, 72.42 (C-3^A), 72.17, 71.90 (C-2^D), 71.83, 71.78 (C-7^E), 71.63 (C-3^D), 70.72 (C-4^B), 70.52, 69.38, 68.17 (C-2^A), 68.04, 67.61, 67.10 (C-8^E), 66.66 (C-2^B), 66.50, 63.06, 62.58, 62.31, 61.79, 55.23 (C-2^C), 53.22 (C-5^E), 48.77 (COOCH_3), 47.81 (CH_2N_3), 37.37 (C-3^E), 29.70, 28.86 ($CH_2CH_2N_3$), 23.15, 21.38, 20.74, 20.67, 20.43 (6 × CH_3CO).

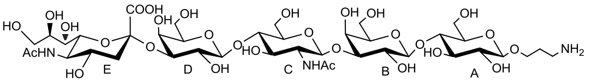

2

3-Aminopropyl 3-*O*-(5-*N*-acetamido-3,5-dideoxy-D-glycero-*α*-D-galacto-non-2-ulopyranosyl)-*β*-D-galactopyranosyl-(1→4)-2-acetamido-2-deoxy-*β*-D-glucopyranosyl-(1→3)-*β*-D-galactopyranosyl-(1→4)-*β*-D-glucopyranoside 2. A mixture of

protected pentasaccharide **36** (0.1 mmol) and LiI (3 mmol) in pyridine (5 mL) was heated for 24 h at 120°C. The reaction mixture was concentrated under vacuum, and the residue was purified by silica gel column chromatography (gradient 2% MeOH in DCM) to afford the demethylated product. This material was dissolved in ethanol (4 mL), and ethylenediamine (400 μL) was added. After being stirred for 16 h at 90 °C, the reaction mixture was then concentrated in vacuum, and the residue was coevaporated from toluene (2 x 10 mL) and EtOH (2 x 5 mL). The crude mixture was re-dissolved in pyridine (5 mL), and acetic anhydride (5 mL) was added. After being stirred for 16 h at room temperature, the reaction mixture was concentrated under reduced pressure. The residue was dissolved in MeOH and MeONa was added until pH=13. After 48 h the reaction was neutralized and the solvent removed under vacuum. The residue was dissolved in MeOH and Pd/C (1:1 w/w in respect to the sugar) was added. The reaction mixture was stirred under pressure of H_2 (5 bar) for 72 h. Then, the catalyst was filtered off and the filtrate concentrated under reduced pressure. The reaction mixture was purified by G-10 size-exclusion column chromatography using water for elution. Fractions containing the sugar were quantified by sialic acid assay and freeze-dried to afford the deprotected oligosaccharide **2** as an amorphous powder (33% yield). $[\alpha]_D^{25}$ = +46.0˚ (c 0.03, H_2O). ESI HR-MS ($C_{40}H_{69}N_3O_{29}$) *m/z* $[M+H]^+$ found 1056.3966; calcd 1056.3971. The compound was identical to the one previously reported in the literature[8].

2.7 Synthesis of tetrasaccharide 5

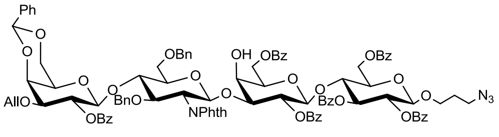

39

3-Azidopropyl 3-*O*-allyl-4,6-*O*-benzylidene-3-*O*-benzoyl-*β*-D-galactopyranosyl-(1→4)-3,6-di-*O*-benzyl-2-deoxy-2-phthalimido-*β*-D-glucopyranosyl-(1→3)-2,6-di-*O*-benzoyl-*β*-D-galactopyranosyl-(1→4)-2,3,6-tri-*O*-benzoyl-*β*-D-glucopyranoside 39. A solution of donor **38** (37 mg, 0.063 mmol) and acceptor **35** (60 mg, 0.042 mmol) with activated 4 Å molecular sieves (50 mg) in dry DCM (3 mL) was stirred under nitrogen. TMSOTf (1.5 µL, 0.008 mmol) was added at –5°C. After 2 h the analytical TLC (4:1 Toluene/EtOAc) showed the presence of a lower spot. The reaction was quenched with TEA, the solid filtered off and the solvent removed under reduced pressure. The crude was purified by column chromatography (Tol/EtOAc) to afford the tetrasaccharide **39** (56 mg, 0.031 mmol) in 72% yield. $[\alpha]_D^{25}$ = +11.2˚ (c 0.15, $CHCl_3$). ESI HR-MS **(**$C_{101}H_{94}N_4O_{28}$) *m/z* $[M+Na]^+$ found 1834.60, calcd 1834.69.

^{1}H-NMR (400 MHz, $CDCl_3$) δ 8.14-6.56 (m, 49H, H-Ar), 5.83-5.68 (m, 1H, C*H*=CH_2), 5.60-5.50 (m, 2H, H-3^D, H-3^A), 5.46 (s, 1H, C*H*Ph), 5.28 (t, *J* = 9.0 Hz, 1H, H-2^A), 5.22-5.14 (m, 2H, H-2^B, C*H*H=CH), 5.09 (d, 2H, C*H*H=CH, incl. $J_{1,2}$ = 8.6 Hz, H-1^C), 4.93 (d, ^2J = 12.0 Hz, 1H, *CH*H-CH=CH_2), 4.72 (d, $J_{1,2}$ = 8.2 Hz, 1H, H-1^D), 4.49 (d, $J_{1,2}$ = 8.2 Hz, 1H, H-1^A), 4,43 (d, *J* = 12.0 Hz,

1H, *C*H*H*-CH=CH_2), 4.42 (d, ^2J = 12.8 Hz, 1H, CH*H*Ph), 4.37 (d, $J_{1,2}$ = 8.2 Hz, 1H, H-1^B), 4.33-4.05 (m, 9H, including H-2^C), (m, 7H, including H-4^A), 3.84 (s, 1H, H-4^B), 3.78-3.71 (m, 1H, OCH_{2a}), 3.60-3.48 (m, 5H, incl. H-3^B, H-3^D), 3.39-3.33 (m, 3H, incl. OCH_{2b}), 3.27 (s, 1H, OH-4^B), 3.13-3.07 (m, 2H, CH_2N_3), 1.69-1.54 (m, 2H, C*H*$_2$$CH_2N_3$).

^{13}C-NMR (101 MHz, $CDCl_3$) 165.89-163.90 (CO), 138.48-126.45 (C-Ar, *CH*=CH_2), 117.38 (*C*H_2CH=CH_2), 101.24 (*C*HPh), 101.07 (C-1^D), 100.99 (C-1^A), 100.45 (C-1^{B,C}), 98.80, 80.99, 78.31, 77.36, 77.27, 76.97, 75.21, 75.10 (*C*H_2Ph), 74.60, 73.34, 73.25, 72.96, 72.49, 72.07 (C-2^B, C-3^A), 71.76 (C-2^A), 71.42, 70.58, 70.46 (C-2^D), 67.55 (C-4^B), 66.50, 66.47, 62.86, 55.32 (C-2^C), 47.81 (*C*H_2N_3), 29.71, 28.86 ($CH_2CH_2N_3$).

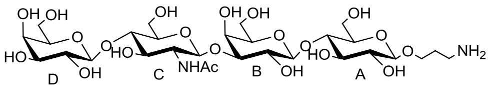

5

3-Aminopropyl *β*-D-galactopyranosyl-(1→4)-2-acetamido-2-deoxy-*β*-D-glucopyranosyl-(1→3)-*β*-D-galactopyranosyl-(1→4)-*β*-D-glucopyranoside 5.

Compound **39** was treated with $PdCl_2$ (2 eq) in methanol (2.5 mL) for 6 h. After filtration, the crude material was dissolved in ethanol (3 mL), and ethylenediamine (400 µL) was added. After being stirred for 16 h at 110 °C, the reaction mixture was concentrated under vacuum, and the residue was coevaporated from toluene (2 x 10 mL) and EtOH (2 x 5 mL). The crude mixture was re-dissolved in pyridine (1.5 mL), and acetic anhydride (1.5 mL) was added at 0°C. After stirring for 16 h at room temperature, the reaction mixture was quenched with MeOH and concentrated under reduced pressure. The mixture was dissolved in MeOH (2 mL) and MeONa was added until pH = 12. After 48h the reaction was neutralized and the solvent removed under vacuum. The compound was purified by flash chromatography in silica C-18 (MeOH/H_2O), and the obtained product was dissolved in MeOH (2 mL), to which Pd/C (1:1 w/w in respect to the sugar) was added. The reaction mixture was stirred under pressure of H_2 (5 bar) for 72 h. Then, the catalyst was filtered off, the filtrate concentrated under reduced pressure and the crude was purified by size exclusion column chromatography affording compound **5** with a yield of 42%. $[\alpha]_D^{25}$ = -28.20° (c 0.1, $CHCl_3$). ESI-HR MS **(**$C_{29}H_{52}N_2O_{21}$) *m/z* $[M+Na]^+$ found 765.3136, calcd 765.3135.

^{1}H-NMR (400 MHz, D_2O) δ 4.57 (d, $J_{1,2}$ = 8.1 Hz, 1H, H-1^C), 4.37 (d, $J_{1,2}$ = 7.8 Hz, 1H, H-1^B), 4.34 (d, $J_{1,2}$ = 7.8 Hz, 1H, H-1^A), 4.30 (d, $J_{1,2}$ = 7.8 Hz, 1H, H-1^D), 4.02 (d, 1H, *J* = 3.1 Hz), 3.95-3.77 (m, 4H), 3.76-3.36 (m, 20H, incl. H-3^A, OC*H*$_2$), 3.19 (t, J = 8.2 Hz, 1H, H-2^A), 3.05 (t, 1H, *J* = 7.2 Hz, C*H*$_2$$N_3$), 1.9 (s, 3H), 1.96-1.81 (m, 1H, C*H*$_2$$CH_2N_3$).

^{13}C-NMR (101 MHz, D_2O) δ 102.9 (C-1^D), 102.8 (C-1^C), 102.7 (C-1^A), 102.0 (C-1^B), 81.9, 78.5, 78.4, 75.2, 74.7, 72.8, 72.6, 72.1, 70.7, 70.1, 68.5, 68.2, 67.8, 61.0, 60.1, 55.2, 37.5, 25.8, 22.2.

2.8 Synthesis of GBS PSIb repeating units

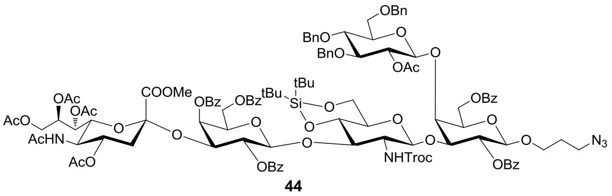

3-Azidopropyl 2-*O*-acetyl-3,4,6-tri-*O*-benzyl-*β*-D-glucopyranosyl-(1→4)-[2,4,6-tri-*O*-benzoyl-3-*O*-(methyl 4,7,8,9-tetra-*O*-acetyl-5-*N*-acetamido-3,5-dideoxy-D-glycero-α-D-galacto-non-2-ulopyranosylonate)-*β*-D-galactopyranosyl-(1→3)-4,6-*O*-di-*tert*-butylsilylidene-3-*O*-(9H-fluoren-9-ylmethylcarbonate)-2-deoxy-2-(2,2,2-trichloroethoxycarbonylamino-β-D-glucopyranosyl-(1→3)]-2,6-di-*O*-benzoyl-β-D-galactopyranoside 44. A solution of trisaccharide acceptor **43** (114 mg, 0.080 mmol) and disaccharide donor **29** (135 mg, 0.120 mmol) with 4Å molecular sieves in dry DCM (3.0 mL) was stirred for 20 minutes. TMSOTf (2.9 µL, 0.016 mmol) was added at 0°C and the reaction was stirred for 2 h and allowed to slowly reach room temperature. After 2 h (TLC 3:2 Tol/Acetone) the reaction was quenched by addition of TEA, and the crude purified by column chromatography using a gradient from 0 to 80% of acetone in toluene. Pure fractions were collected and evaporated affording **44** (150 mg, 80% yield). $[\alpha]_D^{25}$ = -28.20° (c 0.1, $CHCl_3$). ESI HR-MS ($C_{116}H_{132}Cl_3N_5O_{40}Si$) *m/z* $[M+Na]^+$ found 2390.7193, calcd 2390.7176.

^{1}H NMR (400 MHz, ACN-d_3) δ 8.13-7.12 (m, 40H, Ar-H), 5.97 (d, $J_{N,H}$ = 9.8 Hz, 1H, NH), 5.60-5.51 (m, 2H), 5.45-5.38 (m, 1H), 5.31-5.14 (m, 3H, H-1^C, H-2^{C,D}), 5.08 (t, *J* = 9.0 Hz, 1H, H-2^A), 4.85 (d, $J_{1,2}$ = 7.5 Hz, 1H, H-1^D), 4.80-4.59 (m, 6H, incl. H-1^B, H-3^B), 4.58-4.40 (m, 6H, incl. H-1^A), 4.40-4.31 (m, 3H), 4.24 (bs, 1H), 4.22-3.98 (m, 7H), 3.9 (dd, *J* = 9.4, 2.0 Hz, 1H), 3.91-3.81 (m, 2H), 3.81-3.79 (m, 4H, incl. H-2^B) 3.66-3.58 (m, 4H, incl. $COOCH_3$), 3.58-3.49 (m, 3H), 3.48-3.39 (m, 2H), 3.39-3.32 (m, 1H), 3.14-2.98 (m, 2H, OCH_2), 2.24 (dd, $J_{3e,4}$ = 4.5 Hz, $J_{3a,3e}$ = 12.2 Hz, 1H, H-3e^E), 2.01 (s, 3H, CH_3CO), 1.96 (s, 6H, 2 x CH_3CO), 1.78 (s, 3H, CH_3CO), 1.69-1.56 (m, 8H, 2 x CH_3CO, C*H*$_2CH_2N_3$), 1.35 (t, 1H, H-3a^E), 1.04 (s, 9H, tBu), 0.87 (s, 9H, tBu).

^{13}C-NMR (101 MHz, ACN-d_3) δ 170.5, 170.4, 169.9, 169.8, 169.7, 169.5, 165.9, 165.7, 165.6, 165.1, 165.0, 153.7 (13 x CO), 138.6-127.4 (C-Ar), 102.7 (C_q), 101.3 (C-1^B), 100.6 (C-1^A), 98.9 (C-1^D), 97.3 (C-1^C), 95.8 (C_q), 82.4, 80.2, 78.2, 78.0, 75.3, 74.7, 74.5, 74.4, 73.5, 73.2, 72.9, 72.0, 71.8, 71.4, 70.9, 70.8, 70.4, 69.1, 68.9, 67.5, 66.6, 66.1, 63.9, 62.4, 61.9, 58.0, 52.7 ($COOCH_3$), 48.2, 47.6 (*C*H_2N_3), 37.4 (C-3^E), 28.6 (*C*$H_2CH_2N_3$), 27.0 (tBu), 26.4 (tBu), 22.2, 22.1, 20.7 (3 x CH_3CO), 20.5 (*C*$(CH_3)_3$), 20.2, 20.1, 20.0 (3 x CH_3CO), 19.5 (*C*$(CH_3)_3$).

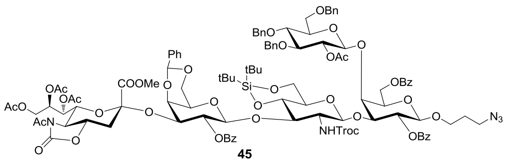

3-Azidopropyl 2-*O*-acetyl-3,4,6-tri-O-benzyl-*β*-D-glucopyranosyl-(1→4)-[3-*O*-(methyl 7,8,9-tri-*O*-acetyl-5-*N*-acetoamido,4-*O*-oxazolidinone-3,5-dideoxy-D-glycero-*α*-D-galacto-non-2-ulopyranosylonate)-*β*-D-

galactopyranosyl-(1→3)-4,6-*O*-di-*tert*-butylsilylidene-3-*O*-(9H-fluoren-9-ylmethylcarbonate)-2-deoxy-2-(2,2,2-trichloroethoxycarbonylamino)-*β*-D-glucopyranosyl-(1→3)]-2,6-di-*O*-benzoyl-*β*-D-galactopyranoside 45. A solution of donor **30** (0.050 g, 0.053 mmol) and acceptor **43** **(**0.050 g, 0.035 mmol) with 4 Å molecular sieves in dry DCM (3.0 mL) was stirred for 20 min under nitrogen. N-iodosuccinimide (0.016 g, 0.074 mmol) and TfOH (0.6 µL, 0.007 mmol) were added at -40°C. After 3 h (TLC 7:3 Tol/Acetone) the reaction was quenched with TEA, the solid was filtered off and the solvent removed under reduced pressure. The crude was purified by flash chromatography (Tol:Acetone) to afford pentasaccharide **45** in 65% yield (0.053 g). $[\alpha]_D^{25}$ = +9.7° (c 1.6, $CHCl_3$). ESI HR-MS **(**$C_{108}H_{124}Cl_3N_5O_{38}Si$) *m/z* $[M+Na]^+$ found 2254.6687, calcd 2254.6651.

^{1}H NMR (400 MHz, ACN-d_3) δ 8.13-7.31 (m, 35H, Ar-H), 5.90 (d, *J* = 9.0 Hz, 1H), 5.61 (s, 1H, C*H*Ph), 5.47-5.40 (m, 1H), 5.29 (d, $J_{N,H}$ = 8.2 Hz, 1H, NH), 5.24 (dd, $J_{1,2}$ = 7.9 Hz, $J_{2,3}$ = 9.9, Hz, 1H, H-2^D), 5.11 (dd, $J_{1,2}$ = 7.9 Hz, $J_{2,3}$ = 10.0 Hz, 1H, H-2^A), 5.01 (d, $J_{1,2}$ = 7.7 Hz, 1H, H-1^D), 4.98 (d, $J_{1,2}$ = 8.3 Hz, 1H, H-1^B), 4.92 (d, $J_{1,2}$ = 8.1 Hz, 1H, H-1^C), 4.87-4.80 (m, 3H), 4.67 (t, *J* = 10.7 Hz, 2H), 4.62 (d, $J_{1,2}$ = 9.6 Hz, 1H), 4.58 (d, *J* = 5.4 Hz, 1H), 4.56-4.51 (m, 3H), 4.47 (d, *J* = 12.0 Hz, 1H), 4.42 (dd, *J* = 12.3, 2.8 Hz, 1H), 4.38 (d, *J* = 12.3 Hz, 1H), 4.35-4.30 (m, 2H), 4.30-4.12 (m, 5H), 4.12-4.06 (m, 2H), 4.06-3.80 (m, 6H), 3.77-3.60 (m, 6H), 3.61-3.55 (m, 4H, incl. $COOCH_3$), 3.54-3.47 (m, 2H), 3.34 (t, *J* = 8.0 Hz, 1H, H-2^B), 3.22-3.06 (m, 2H, CH_2N_3), 2.47-2.41 (m, 1H, H-3e^E), 2.43 (s, 3H, $COCH_3$), 2.17 (s, 3H, $COCH_3$), 2.05 (s, 6H, 2 x $COCH_3$), 1.97 (t, 1H, $J_{3a,3e}$ = 12.7 Hz, H-3a^E), 1.92 (s, 3H, $COCH_3$), 1.75-1.65 (m, 2H, C*H*$_2$$CH_2N_3$), 1.17 (s, 9H, tBu), 1,09 (s, 9H, tBu).

^{13}C-NMR (101 MHz, ACN-d_3) δ 171.2, 170.6, 170.2, 169.8, 169.7, 167.8, 165.8, 165.1, 164.8, 153.8, 153.4 (11xCO), 138.7-126.6 (C-Ar), 101.8 (C-1^B), 101.2 (C-1^A), 100.8 (*C*HPh), 100.5 (C-1^C), 100.0 (C-1^D), 98.6 (C_q), 95.9 (C_q, CCl_3), 82.2, 80.1, 79.2, 78.1, 75.6, 75.4, 74.7, 74.6, 74.5, 74.5, 74.4, 73.5, 73.0, 72.9, 71.7, 71.1, 70.9, 70.5, 70.3, 69.0, 68.6, 68.2, 66.1, 63.8, 62.9, 58.3, 58.1 (C-2^B), 52.8 ($COOCH_3$), 47.6 (*C*H_2N_3), 34.4 (C-3^E), 28.5 (*C*$H_2CH_2N_3$), 26.9 (tBu), 26.5 (tBu), 23.9, 20.5, 20.5, 20.4 (5 x *C*H_3CO), 20.1 (*C*$(CH_3)_3$), 19.5 (*C*$(CH_3)_3$).

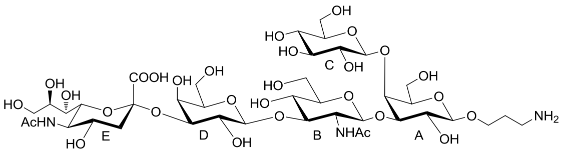

3

2-Aminopropyl *β*-D-glucopyranosyl-(1→4)-[3-*O*-(5-acetamido-3,5-dideoxy-D-glycero-*α*-D-*galacto*-non-2-ulopyranosyl)-*β*-D-galactopyranosyl-(1→3)-2-acetamido-2-deoxy-*β*-D-glucopyranosyl-(1→3)]-*β*-D-galactopyranoside 3.

*Protocol followed for both compound **44** and compound **45***: the protected pentasaccharide (0.07 mmol) was dissolved in THF (5 mL) to which 3 M NaOH (0.5 mL) was added. After refluxing for 2 d, the mixture was neutralized with 0.1% HCl and concentrated. The residue was re-dissolved in 2:3

Ac_2O-MeOH (5 mL) and stirred overnight, when C18-TLC (2:3 H_2O/MeOH) showed disappearance of the starting material. After concentration, the residue was dissolved in tBuOH and Pd/C (1:1 w/w in respect to the sugar) was added. The reaction mixture was stirred under pressure of H_2 (5 bar) for 72 h. Then, the catalyst was filtered off and the filtrate concentrated under reduced pressure. The reaction mixture was purified by G-10 size-exclusion column chromatography using water for elution. Fractions containing the sugar were quantified by sialic acid assay and freeze-dried to afford the deprotected oligosaccharide **3** as an amorphous powder (40% yield). $[\alpha]_D^{25}$ = +1.95° (c 0.1, H_2O). ESI HR-MS ($C_{40}H_{69}N_3O_{29}$) *m/z* $[M-H]^-$ found 1054.3847; calcd 1054.3971.

^{1}H NMR (400 MHz, D_2O) δ 4.87 (d, $J_{1,2}$ = 7.6 Hz, 1H, H-1^C), 4.72 (d, $J_{1,2}$ = 8.1 Hz, 1H, H-1^B), 4.47 (d, $J_{1,2}$ = 8.1 Hz, 1H, H-1^D), 4.38 (d, $J_{1,2}$ = 7.6 Hz, 1H, H-1^A), 4.25 (d, $J_{3,4}$ = 2.9 Hz, 1H, H-4^D), 4.05 (dd, 1H, $J_{2,3}$ = 9.9 Hz, H-3^D), 4.03-3.97 (m, 1H), 3.94-3.44 (m, 27H), 3.41-3.30 (m, 2H), 3.23 (dd, 1H, $J_{2,3}$ = 9.2 Hz, H-2^C), 3.12 (dt, J = 6.9, 8.2 Hz, 2H), 2.73 (dd, $J_{3e,4}$ = 4.7, 12.5, Hz, 1H, H-3e^E), 2-03-1.93 (m, 2H, C*H*$_2$CH$_2$NH$_2$), 1.99 (s, 3H, CH_3CO), 1.98 (s, 3H, CH_3CO), 1.75 (t, 1H, H-3$^E_{ax}$).

^{13}C-NMR (101 MHz, D_2O) δ 174.9, 174.8, 173.8 (3 x CO), 103.3 (C-1^A), 102.9 (C-1^D), 102.7 (C-1^C), 102.0 (C-1^B), 100.0 (C_q), 99.6 (C_q), 82.1, 81.9, 75.8, 75.6, 75.3, 75.1, 74.8, (C-4^D) 74.2, 73.6 (C-2^C), 72.8, 71.8, 70.1, 69.7, 69.0, 68.6, 68.3, 68.0, 67.9, 67.2, 62.4, 61.0, 60.8, 60.7, 60.6, 54.7, 51.6, 39.7 (C-3^E), 37.7 (*C*H$_2$N$_3$), 26.6 (C*H*$_2$CH$_2$NH$_2$), 22.3 (*C*H$_3$CO), 22.0 (*C*H$_3$CO). See Figure 4 for assignments.

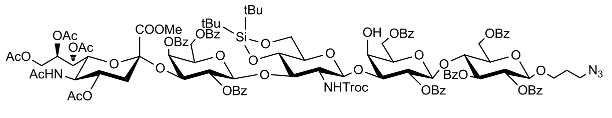

48

3-Azidopropyl 2,4,6-tri-*O*-benzoyl-3-*O*-(methyl-4,7,8,9-tetra-*O*-acetyl-5-*N*-acetamido-3,5-dideoxy-D-glycero-*α*-D-galacto-non-2-ulopyranosylonate)-*β*-D-galactopyranosyl-(1→3)-4,6-*O*-di-*tert*-butylsilylidene-3-*O*-(9H-fluoren-9-ylmethyl carbonate)-2-deoxy-2-(2,2,2-trichloroethoxycarbonylamino)-*β*-D-glucopyranosyl-(1→3)-2,6-di-*O*-benzoyl-*β*-D-galactopyranosyl-(1→4)-2,3,6-tri-*O*-benzoyl-*β*-D-glucopyranoside 48. A solution of trisaccharide acceptor **47** (95 mg, 0.067 mmol) and disaccharide donor **29** (114 mg, 0.100 mmol) with 4 Å molecular sieves (0.100 g) in dry DCM (3.0 mL) was stirred for 20 minutes. TMSOTf (2.4 μL, 0.0134 mmol) was then added at 0°C. The reaction was stirred for 2 h and allowed to slowly reach room temperature. The reaction was quenched by addition of TEA (TLC 6:4 Tol:Acetone) and the crude purified by column chromatography using a gradient from 0 to 70% of acetone in toluene. Pure fractions were collected and evaporated affording 110 mg (66% yield) of compound **48** as a pale solid. $[\alpha]_D^{25}$ = +11.27° (c 0.45, $CHCl_3$). ESI HR-MS ($C_{114}H_{124}Cl_3N_5O_{42}Si$) *m/z* $[M+Na]^+$ found 2290.6468; calcd 2290.6556

^{1}H NMR (400 MHz, ACN-d$_3$) δ 8.18-7.10 (m, 40H, Ar-H), 5.95 (t, J = 9.3 Hz, 1H), 5.67-5.58 (m, 2H, incl. H-3^A), 5.57-5.32 (m, 3H), 5.27-5.07 (m, 4H, incl. H-1^D, H-2^A), 4.86-4.65 (m, 3H, incl. H-1^A, H-4^E), 4.61 (d, 2H, incl. $J_{1,2}$ = 8.2 Hz, H-1^C), 4.48-4.26 (m, 6H), 4.21 (t, J = 9.5 Hz, 1H), 4.17-4. 09 (m, 2H), 4.03-3.85 (m, 5H), 3.84-3.78 (m, 1H), 3.78-3.73 (m, 3H), 3.73-3.65 (m, 3H), 3.65-3.55 (m, 4H, incl. COOCH$_3$), 3.53-3.45 (m, 1H, H-2^C), 3.43-3.28 (m, 1H), 3.10 (t, J = 6.6 Hz, 2H, OCH$_2$), 2.22 (dd, 1H, $J_{3e,4}$ = 4.5 Hz, $J_{3a,3e}$ = 12.5 Hz, H-3e^E), 2.17 (s, 3H, CH$_3$CO), 2.02 (s, 3H, CH$_3$CO), 1.97 (s, 3H, CH$_3$CO), 1.78 (s, 3H, CH$_3$CO), 1.65 (s, 3H, CH$_3$CO), 1.67-1.59 (m, 2H, CH_2CH$_2$N$_3$), 1.34 (t, $J_{3a,3e}$ = 12.2 Hz, 1H, H-3a^E), 0.99 (s, 9H, tBu), 0.82 (s, 9H, tBu).

^{13}C-NMR (101 MHz, ACN-d$_3$) δ 170.5, 169.9, 169.8, 169.8, 169.7, 169.5, 168.0, 165.7, 165.6, 165.5, 165.4, 165.1, 165.0, 164.9, 153.9 (15 x CO), 133.8-125.3 (C-Ar), 101.5 (C-1^{A,B}), 100.8 (C-1^C), 100.2 (C-1^D), 98.7, 97.2, 79.7, 77.6, 75.6, 74.7, 73.2, 73.0, 72.8, 72.4, 72.2, 72.1, 71.9, 71.9, 71.8, 71.4, 70.9, 70.6, 70.2, 70.0, 69.8, 69.6, 69.2, 69.1, 68.9, 68.0, 68.0, 67.9, 67.5, 67.0, 66.6, 65.8, 62.9, 62.6, 62.5, 62.0, 61.9, 61.8, 57.5, 52.7 (COOCH$_3$), 47.7 (CH$_2$N$_3$), 37.3 (C-3^E), 28.5, (CH$_2$CH$_2$N$_3$), 26.9 (tBu), 26.3 (tBu), 22.2 (CH$_3$CO), 22.1 (CH$_3$CO), 20.7 (CH$_3$CO), 20.5 (C(CH$_3$)$_3$), 20.1 (CH$_3$CO), 20.0 (CH$_3$CO), 19.4 (C(CH$_3$)$_3$).

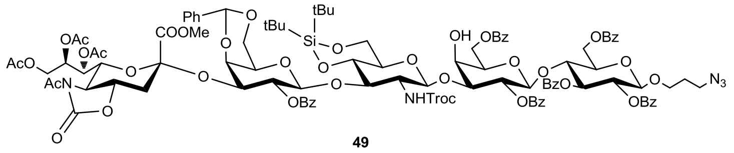

49

3-Azidopropyl 2,4,6-tri-*O*-benzoyl-3-*O*-(methyl-4,7,8,9-tetra-*O*-acetyl-5-*N*-acetamido-3,5-dideoxy-D-glycero-*α*-D-galacto-non-2-ulopyranosylonate)-*β*-D-galactopyranosyl-(1→3)-3,6-di-*O*-benzyl-2-deoxy-2-phthalimido-*β*-D-glucopyranosyl-(1→3)-2,6-di-*O*-benzoyl-*β*-D-galactopyranosyl-(1→4)-2,3,6-tri-*O*-benzoyl-*β*-D-glucopyranoside 49. A solution of donor **30** (40 mg, 0.042 mmol) and acceptor **47 (**40 mg, 0.028 mmol) with 4 Å molecular sieves (0.100 g) in dry DCM (3.0 mL) was stirred for 20 min under nitrogen. N-iodosuccinimide (0.013 g, 0.056 mmol) and TfOH (0.5 μL, 0.0056 mmol) were added at -40°C. After 3 h (TLC 6:4 Tol:Acetone) the reaction was quenched with TEA, the solid was filtered off and the solvent removed under reduced pressure. The crude was purified by flash chromatography (Tol/Acetone) to afford pentasaccharide **49** in 40% yield (0.025 g). $[\alpha]_D^{25}$ = +20.93° (c 0.3, CHCl$_3$). ESI HR-MS ($C_{106}H_{116}Cl_3N_5O_{40}Si$) *m/z* [M+ Na]$^+$ found 2253.63; calcd 2254.59.

^{1}H NMR (400 MHz, CDCl$_3$) δ 7.67-7.27 (m, 35H, Ar-H), 5.80 (dd, J = 9.1, 1.2 Hz, 1H), 5.61 (t, 1H, J =9.5 Hz, H-3^A), 5.50 (s, 1H, CHPh), 5.40 (d, 1H, $J_{N,H}$ = 8.2 Hz, NH), 5.37-5.30 (m, 1H), 5.23 (dd, $J_{1,2}$ = 8.1 Hz, 1H, H-2^A), 5.11-5.05 (m, 2H, H-2^B, H-2^D), 4.91 (d, $J_{1,2}$ = 8.0 Hz, 1H, H-1^D), 4.79-4.71 (m, 2H, H-1^C, H-1^A), 4.59 (d, $J_{1,2}$ = 8.1 Hz, 1H, H-1^B), 4.56-4.49 (m, 1H), 4.45-4.40 (m, 1H), 4.40-4.29 (m, 3H), 4.26-4.20 (m, 2H), 4.19-4.09 (m, 4H), 4.08-3.97 (m, 3H), 3.97-3.89 (m, 2H), 3.84-3.79

(m, 1H), 3.79-3.71 (m, 4H), 3.58-3.50 (m, 2H), 3.49 (s, 3H, $COOCH_3$), 3.48-3.43 (m, 1H), 3.38 (dt, J = 4.9, 9.8 Hz, 1H, Hz), 3.19-3.09 (m, 3H, H-2^C, OCH_2), 2.34 (s, 3H, $COCH_3$), 2.33 (s, 3H, $COCH_3$), 2.35-2.30 (m, 1H, H-3^E_{eq}), 2.08 (s, 3H, $COCH_3$), 1.96 (s, 3H, $COCH_3$), 1.87 (t, 1H, J=12.3 Hz, H-1^E_{ax}), 1.68-1.58 (m, 2H, $CH_2CH_2N_3$), 1.04 (s, 9H, tBu), 0.96 (s, 9H, tBu).

^{13}C-NMR (101 MHz, ACN-d_3) δ 172.1, 171.5, 170.7, 170.6, 168.7, 166.6, 166.5, 166.3, 166.0, 165.8, 154.7, 154.6, 139.4 (13 x CO), 134.3-127.4 (C-Ar), 101.6, 101.3 (*C*HPh), 101.6 (C-1^B), 101.3 (*C*HPh), 101.1 (C-1^A), 100.9 (C-1^C), 100.7 (C-1^D), 99.5 (C_q), 80.0, 79.7, 76.5, 75.4, 75.4, 75.3, 74.1, 73.9, 73.7, 73.1, 73.0, 72.0, 71.6, 71.4, 71.0, 69.4, 69.1, 68.8, 67.5, 67.1, 66.8, 63.8, 63.4, 59.2, 58.2 (C-2^C), 53.7, 48.7 (O*C*H_2), 35.3 (C-3^E), 29.4 (*C*$H_2CH_2N_3$), 27.7 (tBu), 27.4 (tBu), 24.8 (*C*H_3CO), 23.0 (*C*($CH_3)_3$), 21.4 (*C*OCH_3), 21.3 (*C*H_3CO), 21.0 (*C*H_3CO), 20.3 (*C*($CH_3)_3$).

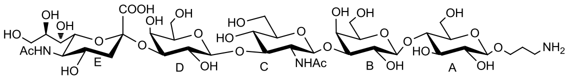

4

3-Aminopropyl 3-*O*-(5-*N*-acetamido-3,5-dideoxy-D-glycero-*α*-D-galacto-non-2-ulopyranosyl)-*β*-D-galactopyranosyl-(1→3)-2-acetamido-2-deoxy-*β*-D-glucopyranosyl-(1→3)-*β*-D-galactopyranosyl-(1→4)-*β*-D-glucopyranoside 4.

*Protocol followed for both compound **48** and compound **49***: the protected pentasaccharide (0.07 mmol) was dissolved in THF (5 mL) to which 3 M NaOH (0.5 mL) was added. After refluxing for 2 d, the mixture was neutralized with 0.1% HCl and concentrated. The residue was re-dissolved in 2:3 Ac_2O-MeOH (5 mL) and stirred overnight. After concentration, the residue was dissolved in tBuOH and Pd/C (1:1 w/w in respect to the sugar) was added. The reaction mixture was stirred under pressure of H_2 (5 bar) for 72 h. Then, the catalyst was filtered off and the filtrate concentrated under reduced pressure. The reaction mixture was purified by G-10 size-exclusion column chromatography using water for elution. Fractions containing the sugar were quantified by sialic acid assay and freeze-dried to afford the deprotected oligosaccharide **4** as an amorphous powder (40% yield). $[\alpha]_D^{25}$ = -4.37° (c 0.05, H_2O). ESI HR-MS ($C_{40}H_{69}N_3O_{29}$) *m/z* $[M-H]^-$ found 1054.3867; calcd 1054.3971.

^{1}H NMR (400 MHz, D_2O) δ 4.68 (d, $J_{1,2}$ = 8.3 Hz, 1H, H-1^C), 4.48 (d, $J_{1,2}$ = 8.3 Hz, 2H, H-$1^{A,B}$), 4.41 (d, $J_{1,2}$ = 7.5 Hz, 1H, H-1^D), 4.12 (d, $J_{3,4}$ = 2.9 Hz, 1H, H-4^B), 4.07-3.46 (m, 33H), 3.33-3.26 (m, 1H, H-2^A), 3.11 (t, J = 6.8 Hz, 2H, CH_2N_3), 2.72 (dd, $J_{3e,4}$ = 4.1 Hz, $J_{3e,ea}$ = 11.9 Hz, 1H, H-3e^E), 1.99 (s, 6H, 2 x CH_3CO), 2.03-1.91 (m, 2H, C*H_2*CH_2N_3), 1.75 (t, 1H, H-3a^E).

^{13}C-NMR (101 MHz, D_2O) δ 174.9 (CO), 173.9 (CO), 103.3 (C-1^A), 102.9 (C-1^B), 102.5 (C-1^D), 102.0 (C-1^C), 81.9, 81.7, 78.3, 75.5, 75.0, 74.8, 74.7, 74.3, 72.6, 71.8, 69.9, 69.0, 68.4, 68.3, 67.8 (O*C*H_2), 67.2, 62.4, 61.0, 60.9, 60.4, 59.9, 54.5, 51.6, 39.7 (C-3^E), 37.5 (*C*H_2N_3), 26.6 (*C*$H_2CH_2N_3$), 22.2 (*C*H_3CO), 22.0 (*C*H_3CO).

3. NMR Spectra of synthesized compounds

Compound 52: ^{1}H NMR, $CDCl_3$, 400 MHz

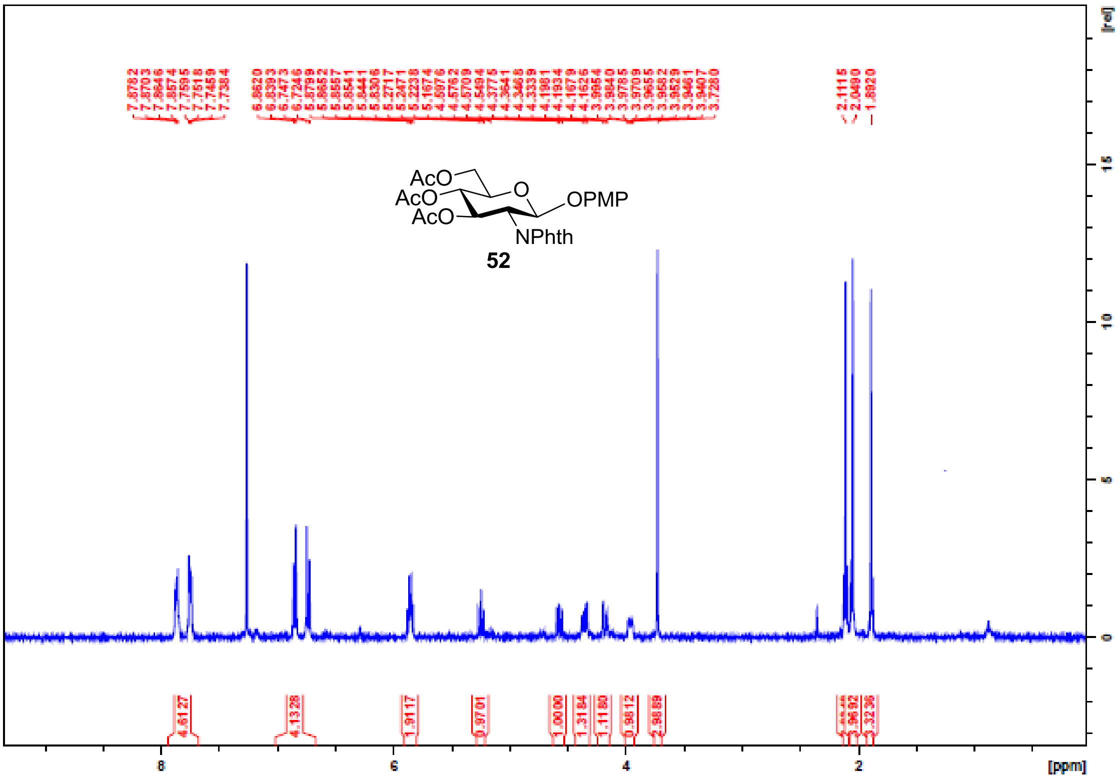

Compound 52: ^{13}C NMR, $CDCl_3$, 101 MHz

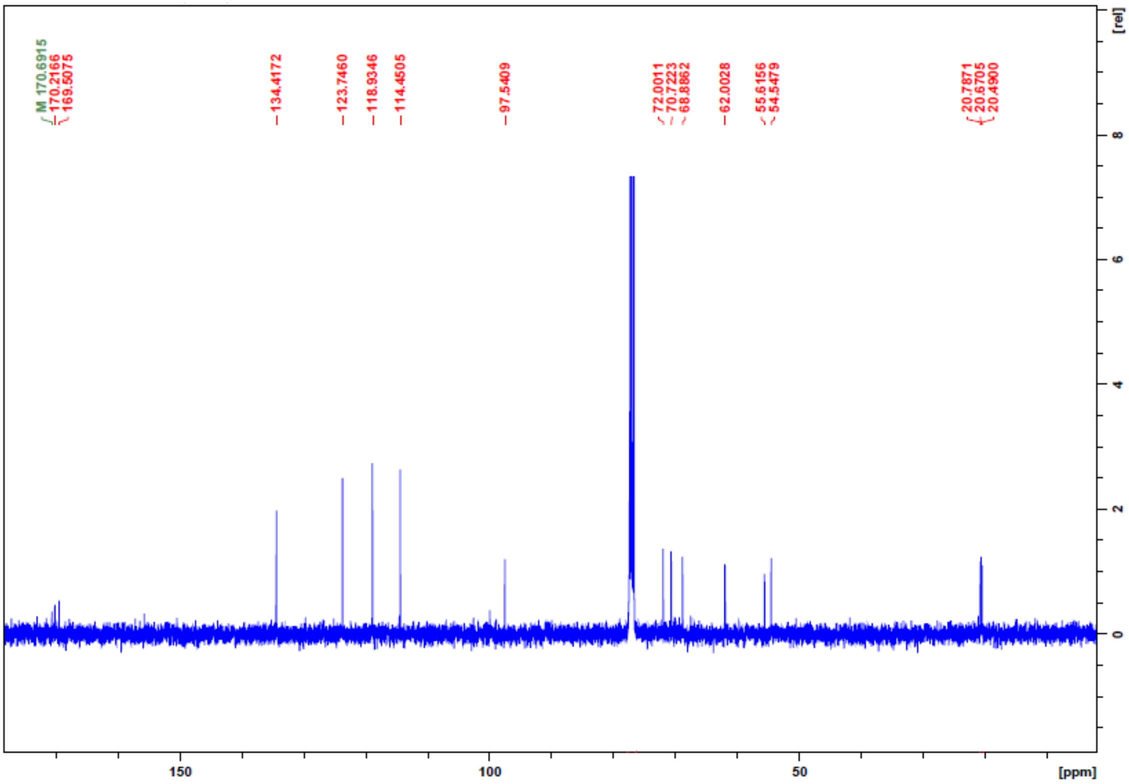

Compound 53: ^{1}H NMR, $CDCl_3$, 400 MHz

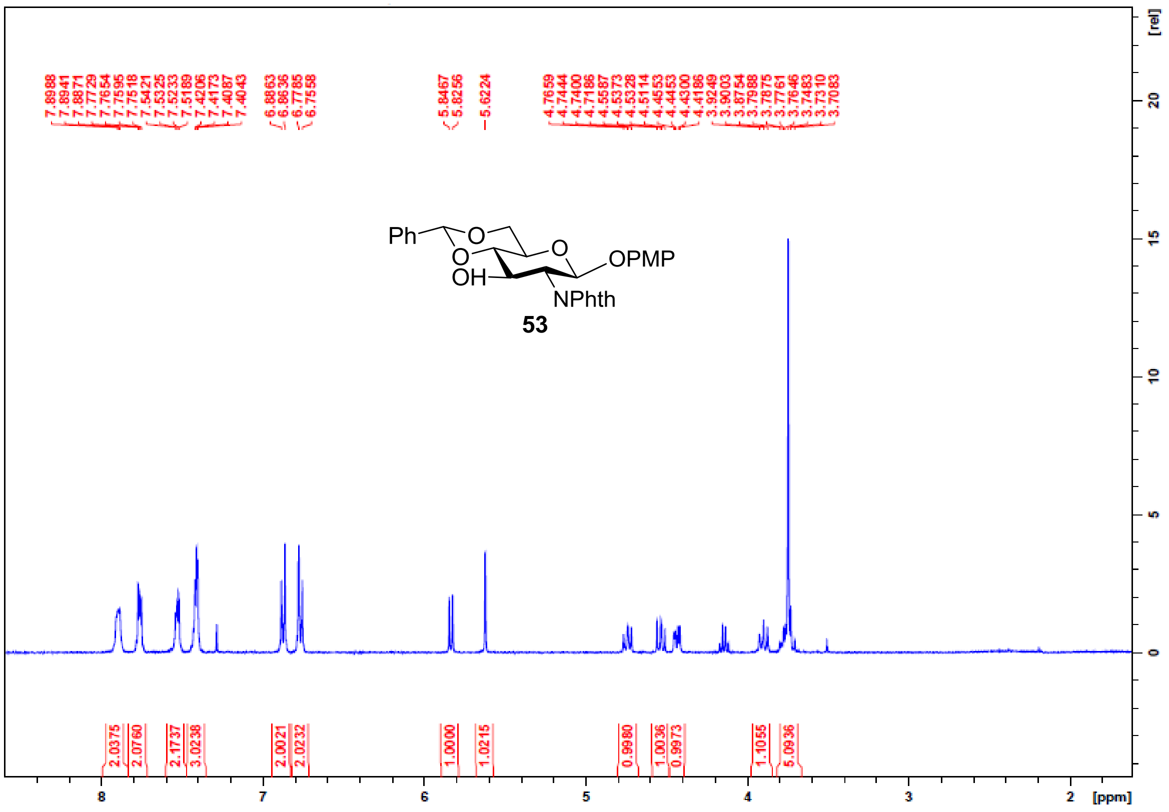

Compound 53: ^{13}C NMR, $CDCl_3$, 101 MHz

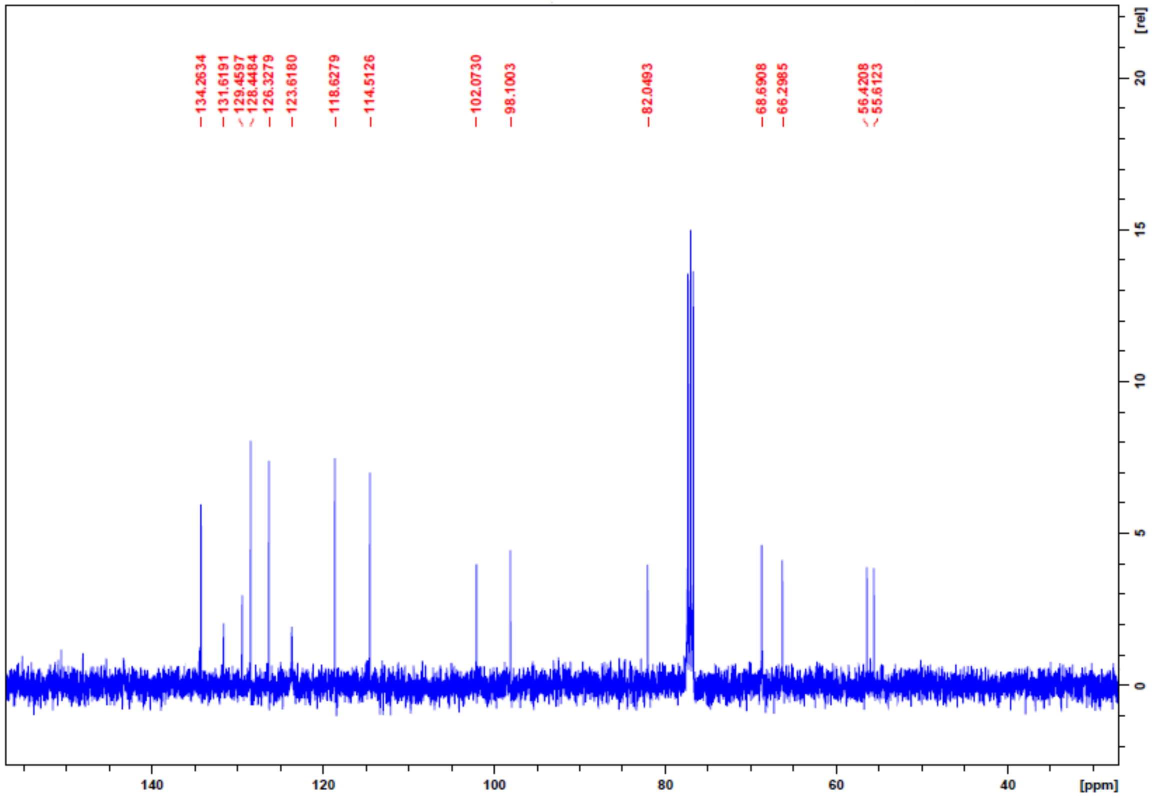

Compound 54: ^{1}H NMR, $CDCl_3$, 400 MHz

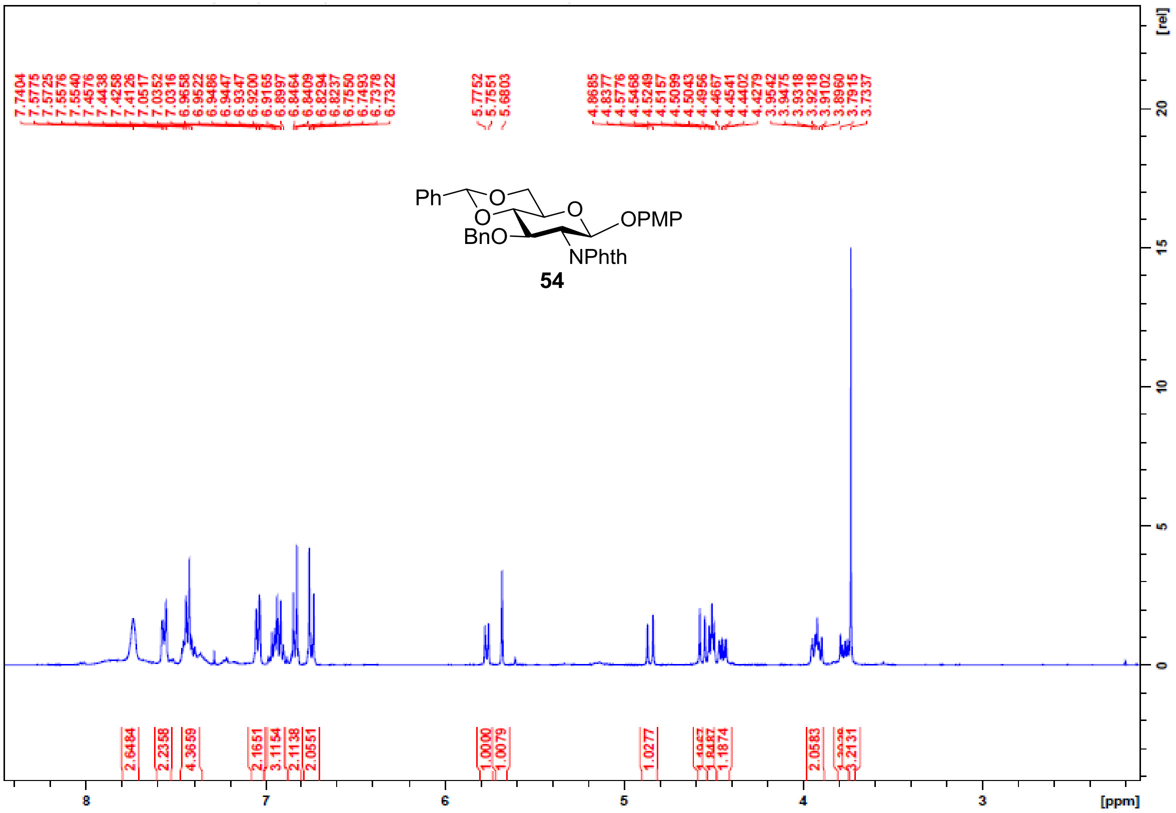

Compound 54: ^{13}C NMR, $CDCl_3$, 101 MHz

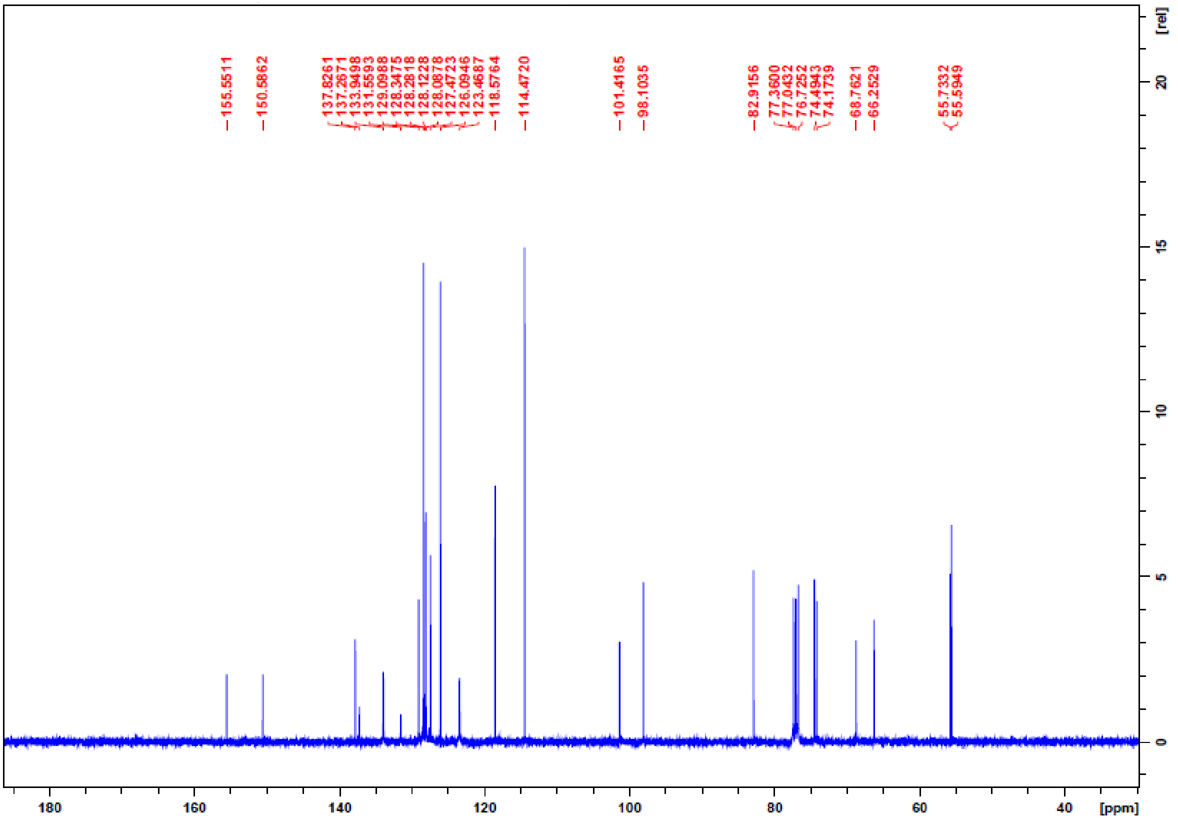

Compound 8: ^{1}H NMR, $CDCl_3$, 400 MHz

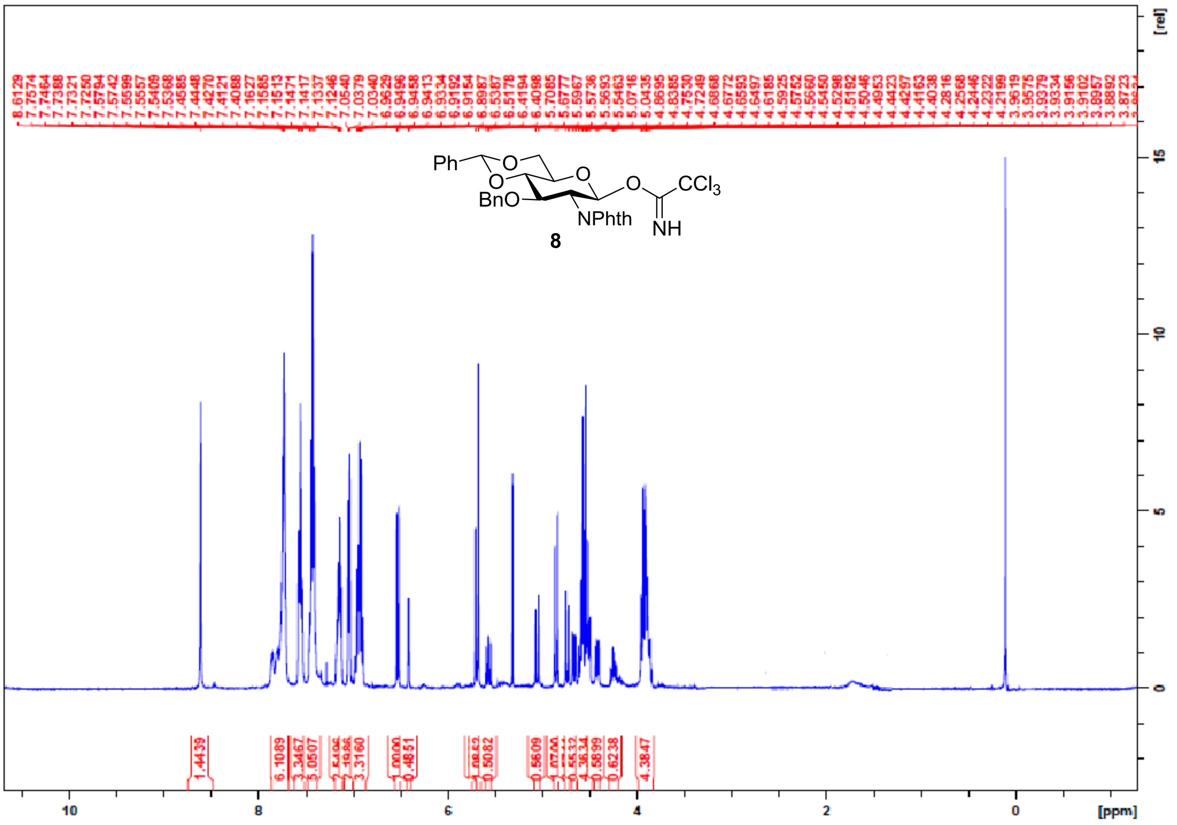

Compound 8: ^{13}C NMR, $CDCl_3$, 101 MHz

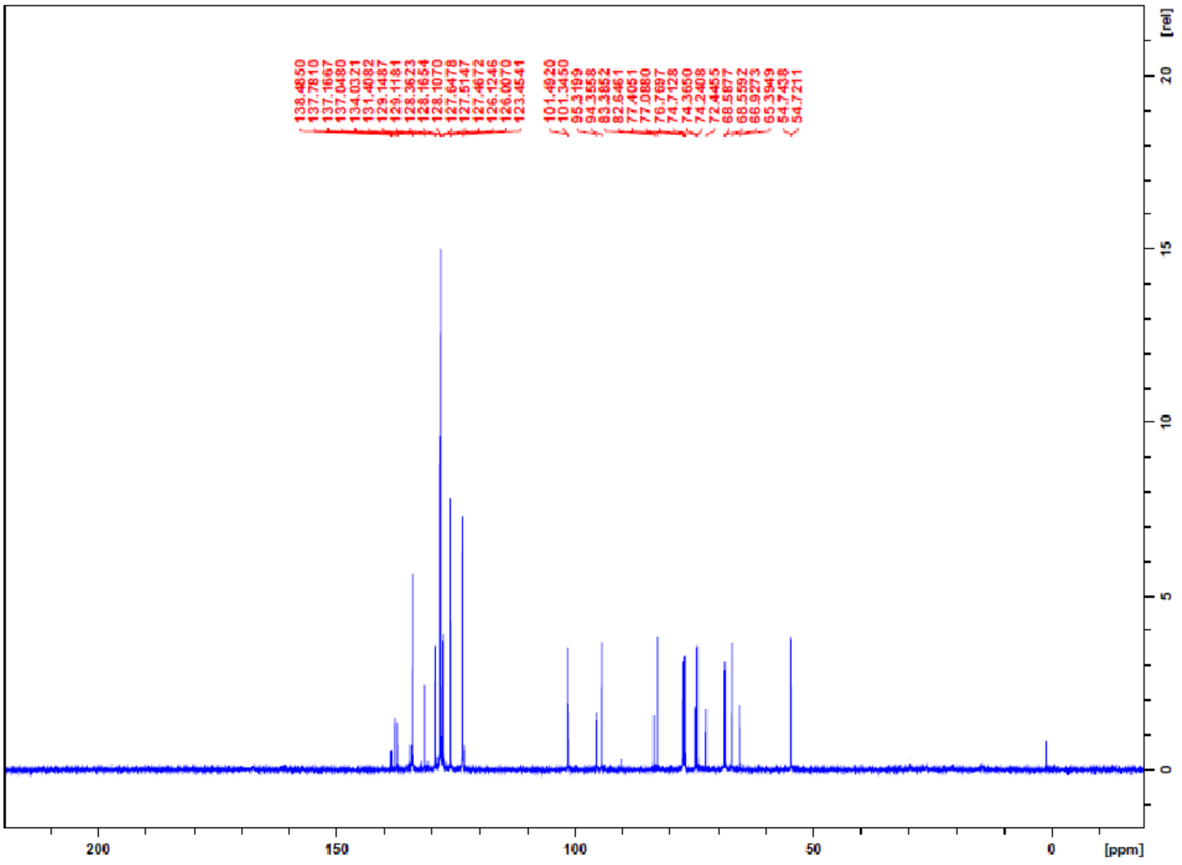

Compound 55: ^{1}H NMR, $CDCl_3$, 400 MHz

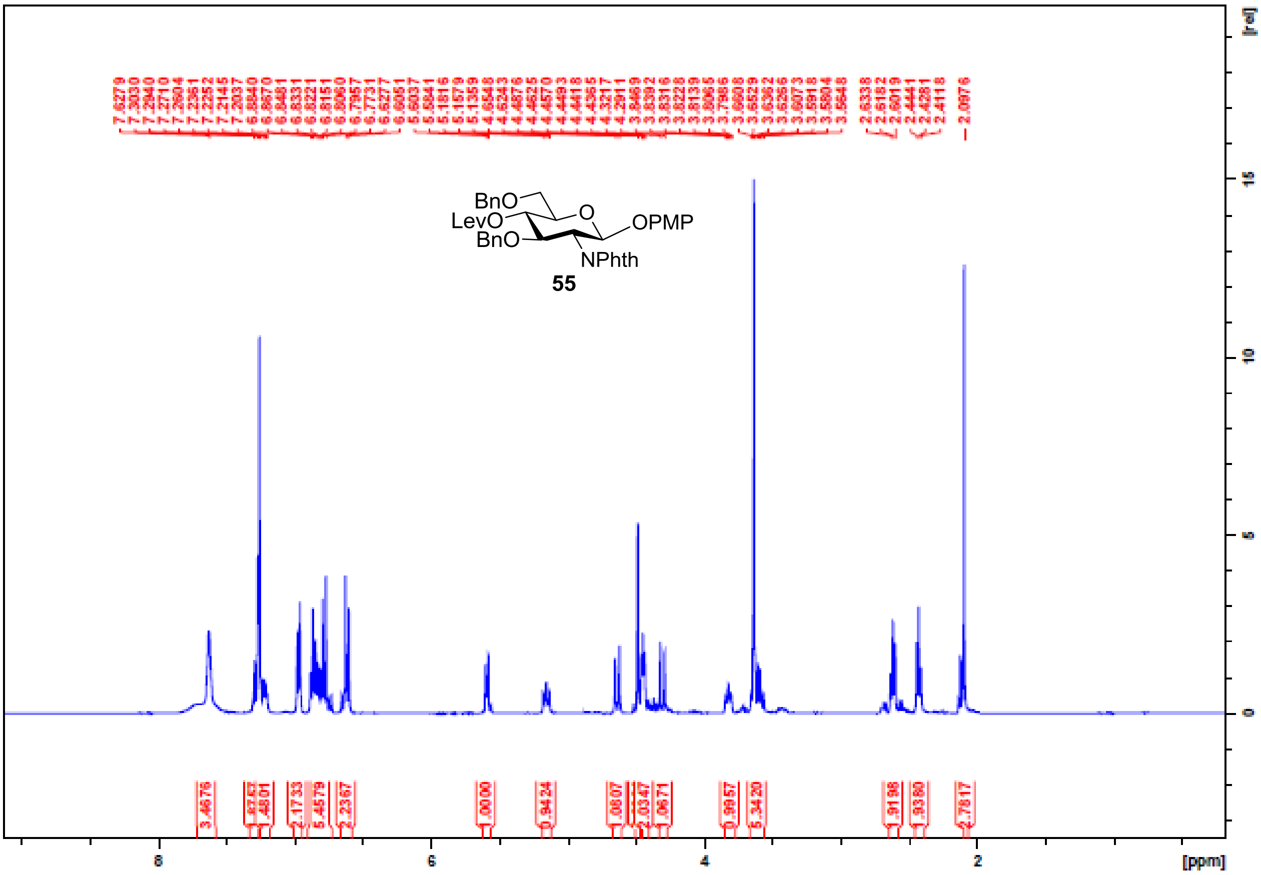

Compound 55: ^{13}C NMR, $CDCl_3$, 101 MHz

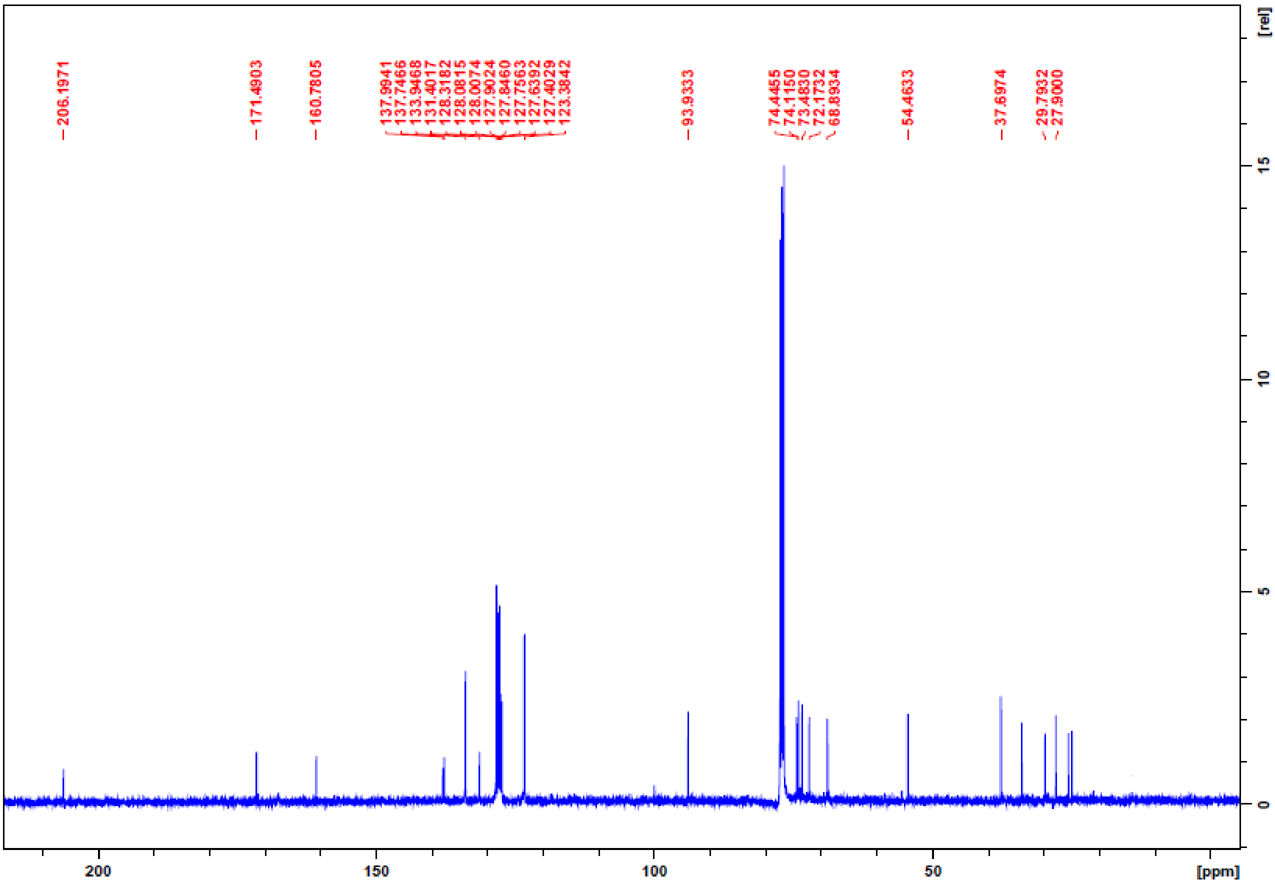

Compound 9: ^{1}H NMR, $CDCl_3$, 400 MHz

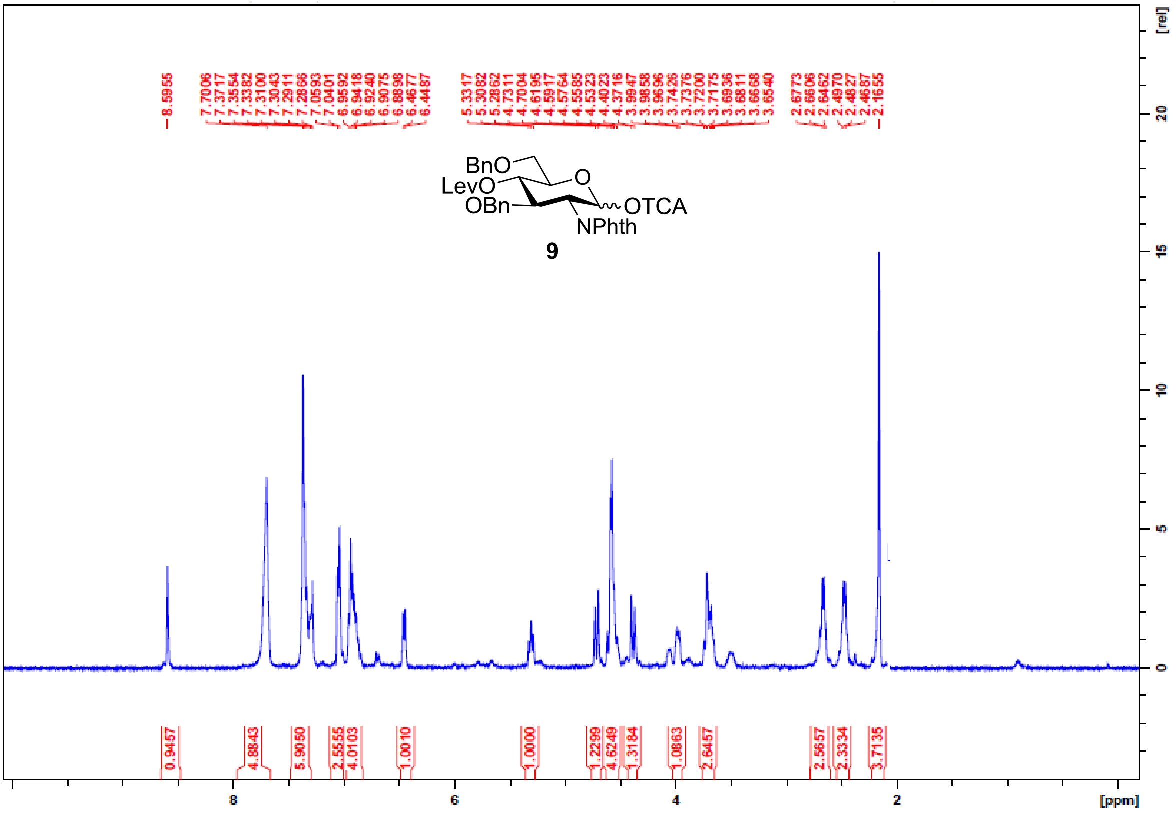

Compound 9: ^{13}C NMR, $CDCl_3$, 101 MHz

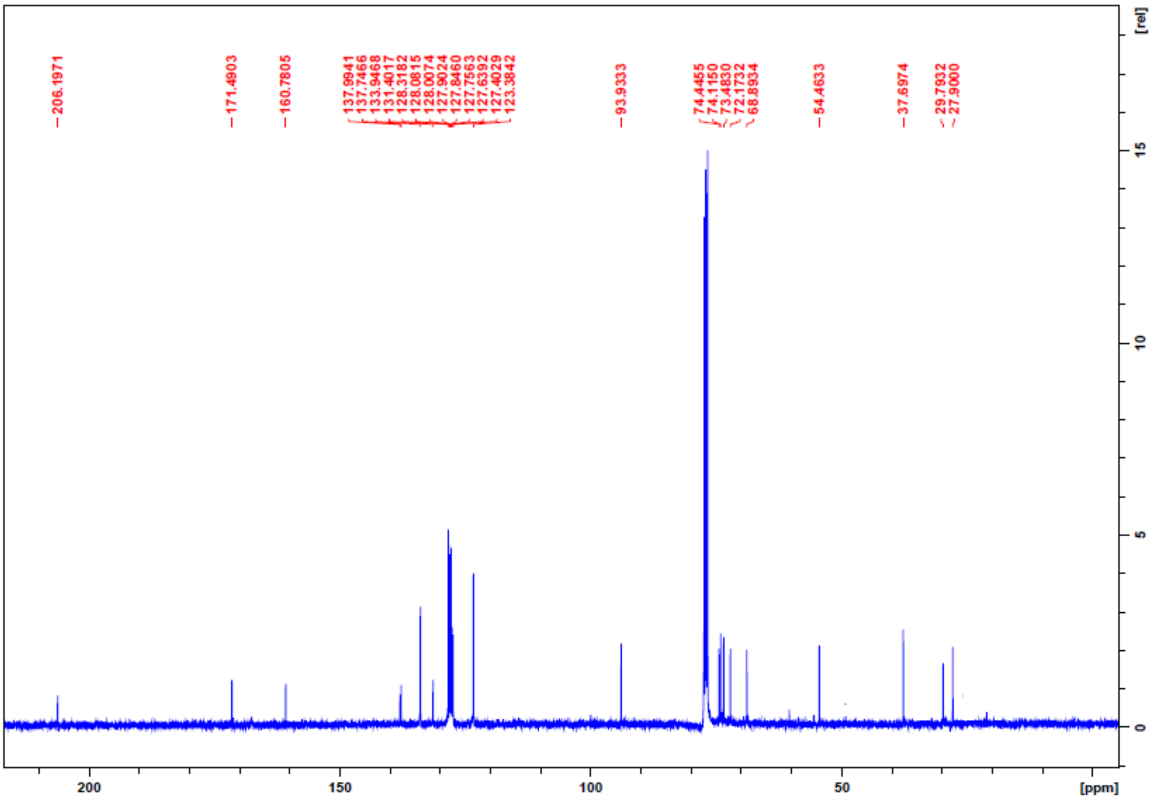

Compound 16: ^{1}H NMR, $CDCl_3$, 400 MHz

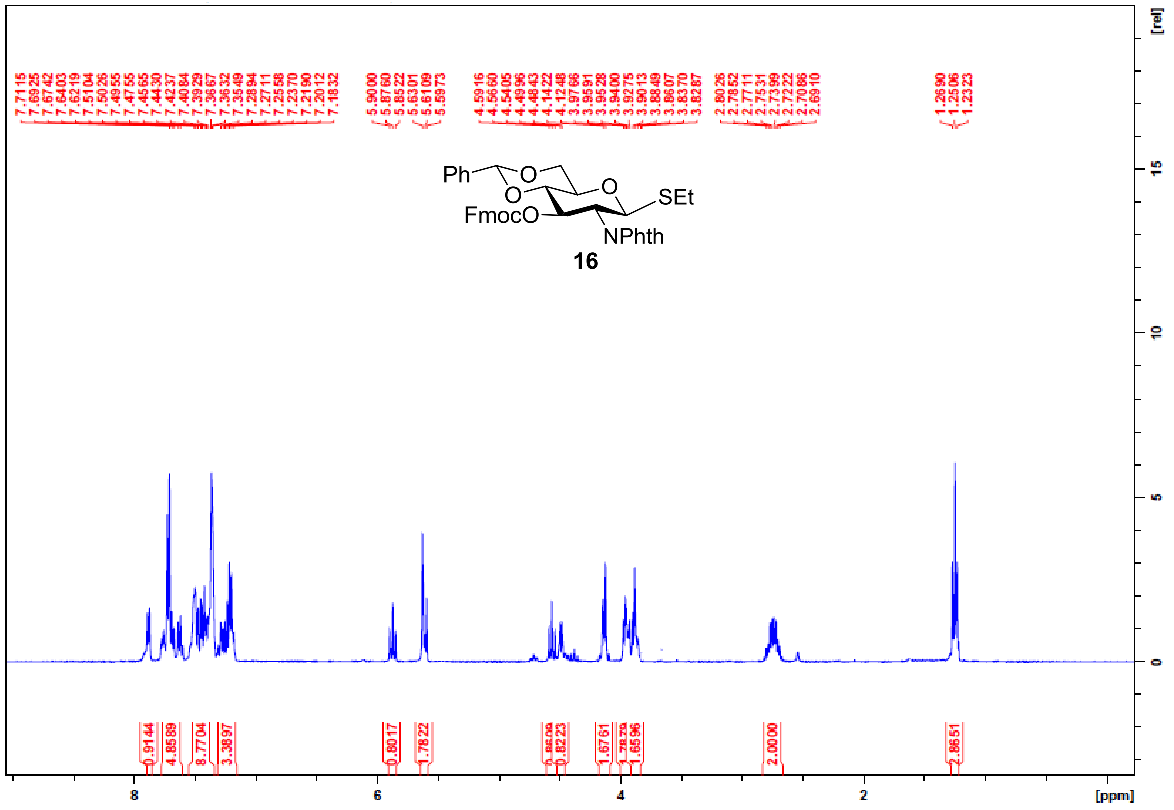

Compound 16: ^{13}C NMR, $CDCl_3$, 101 MHz

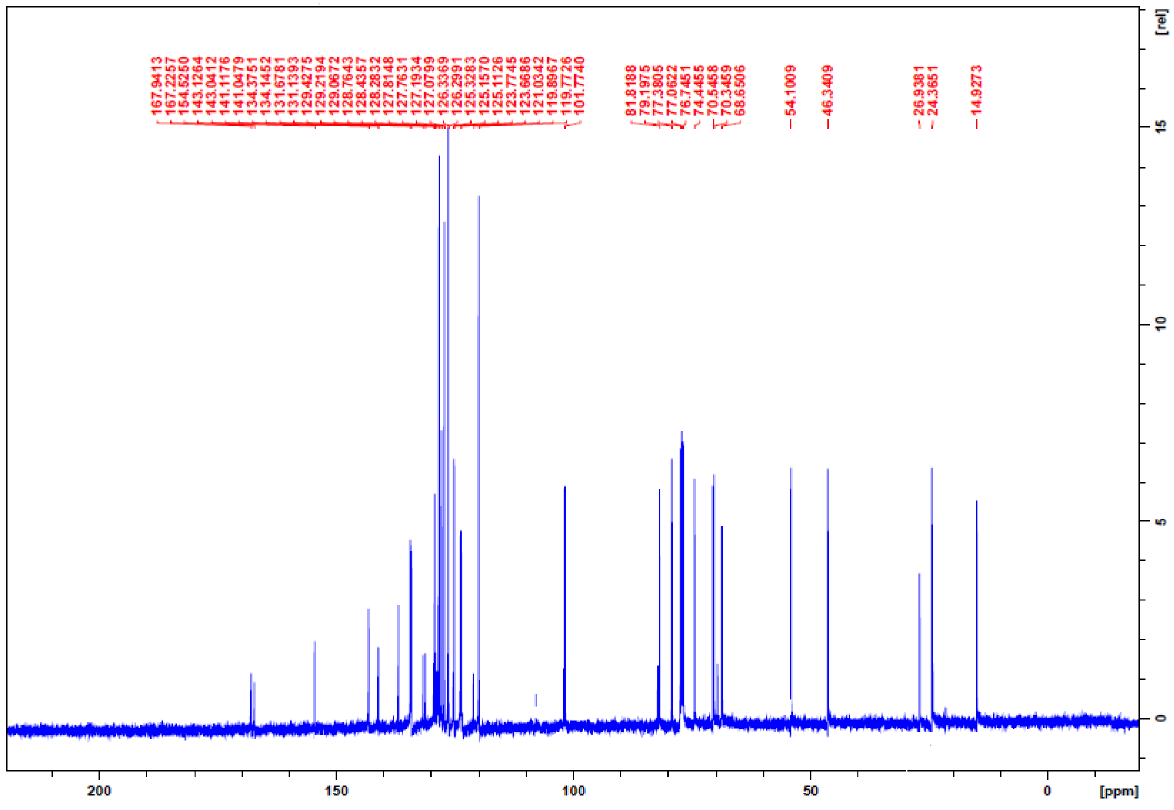

Compound 17: ^{1}H NMR, $CDCl_3$, 400 MHz

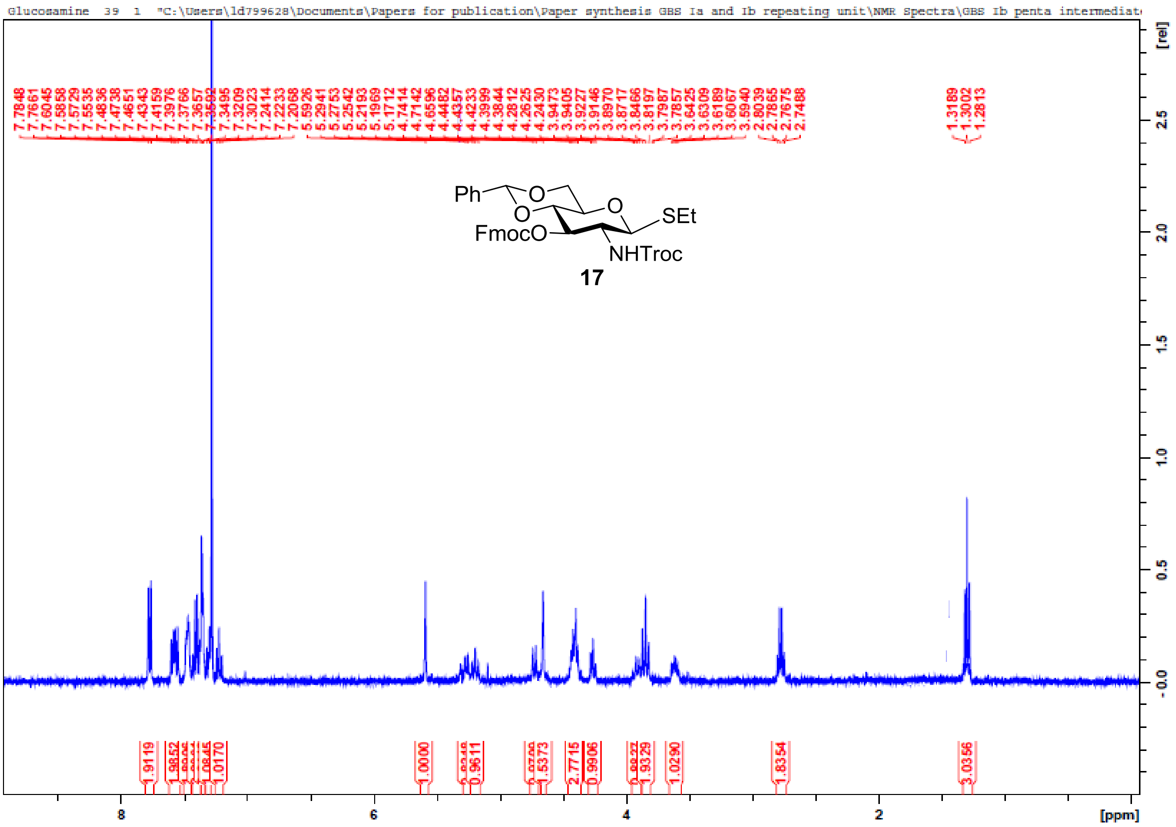

Compound 17: ^{13}C NMR, $CDCl_3$, 101 MHz

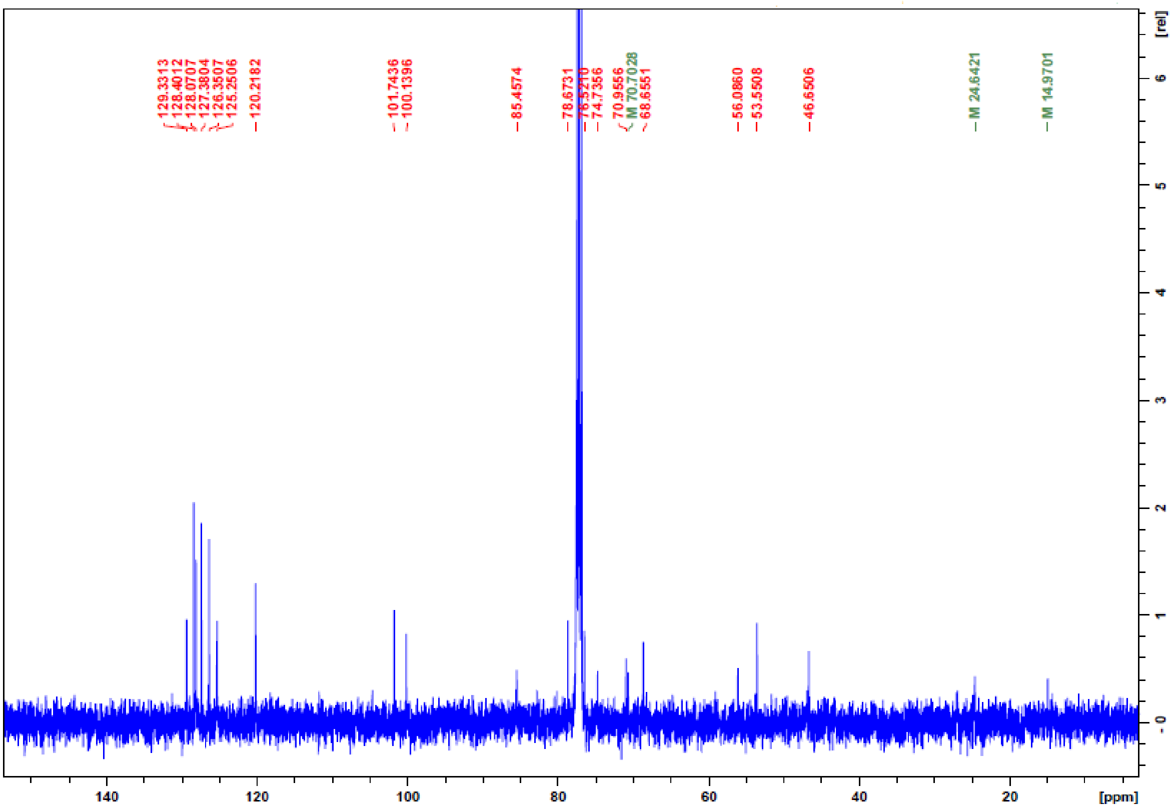

Compound 61: ^{1}H NMR, CDCl$_3$, 400 MHz

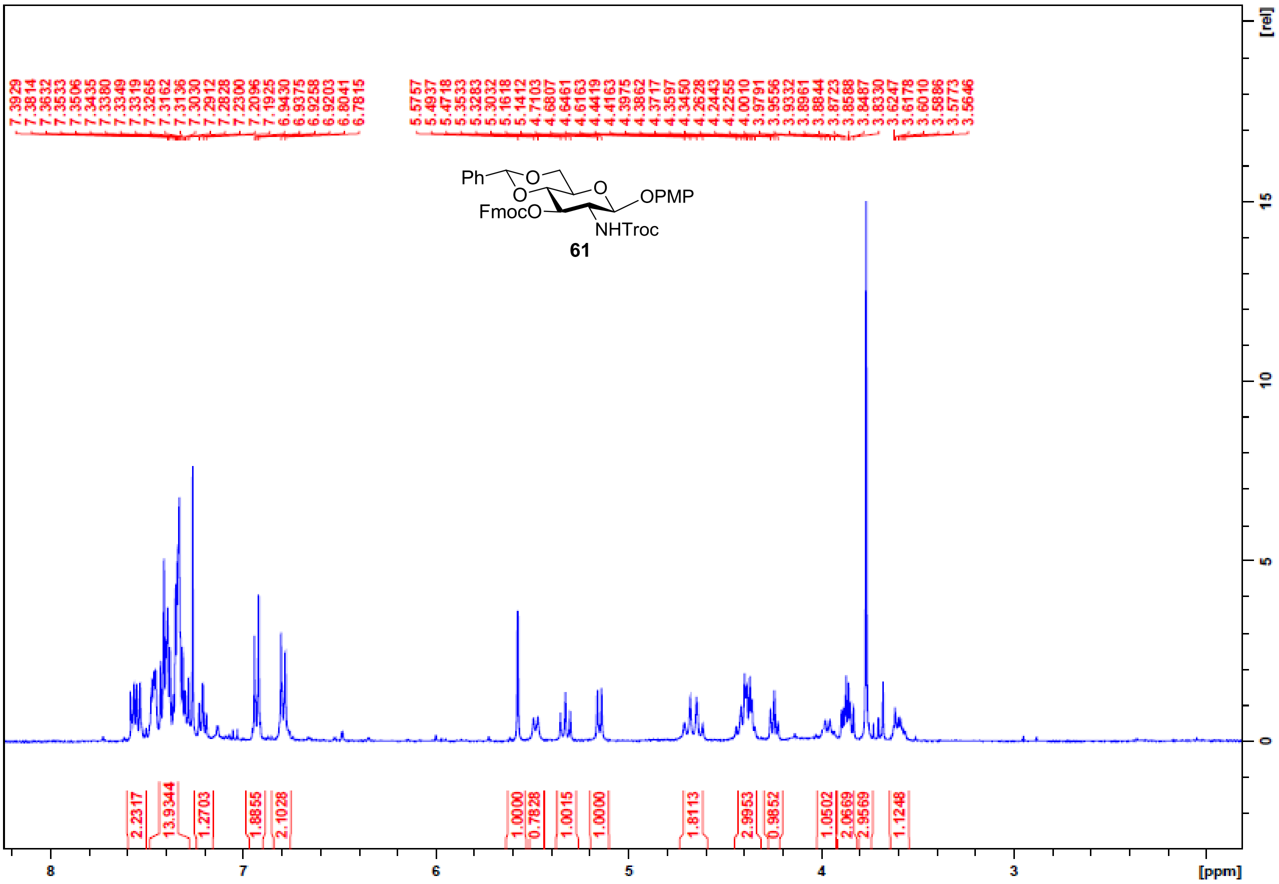

Compound 61: ^{13}C NMR, CDCl$_3$, 101 MHz

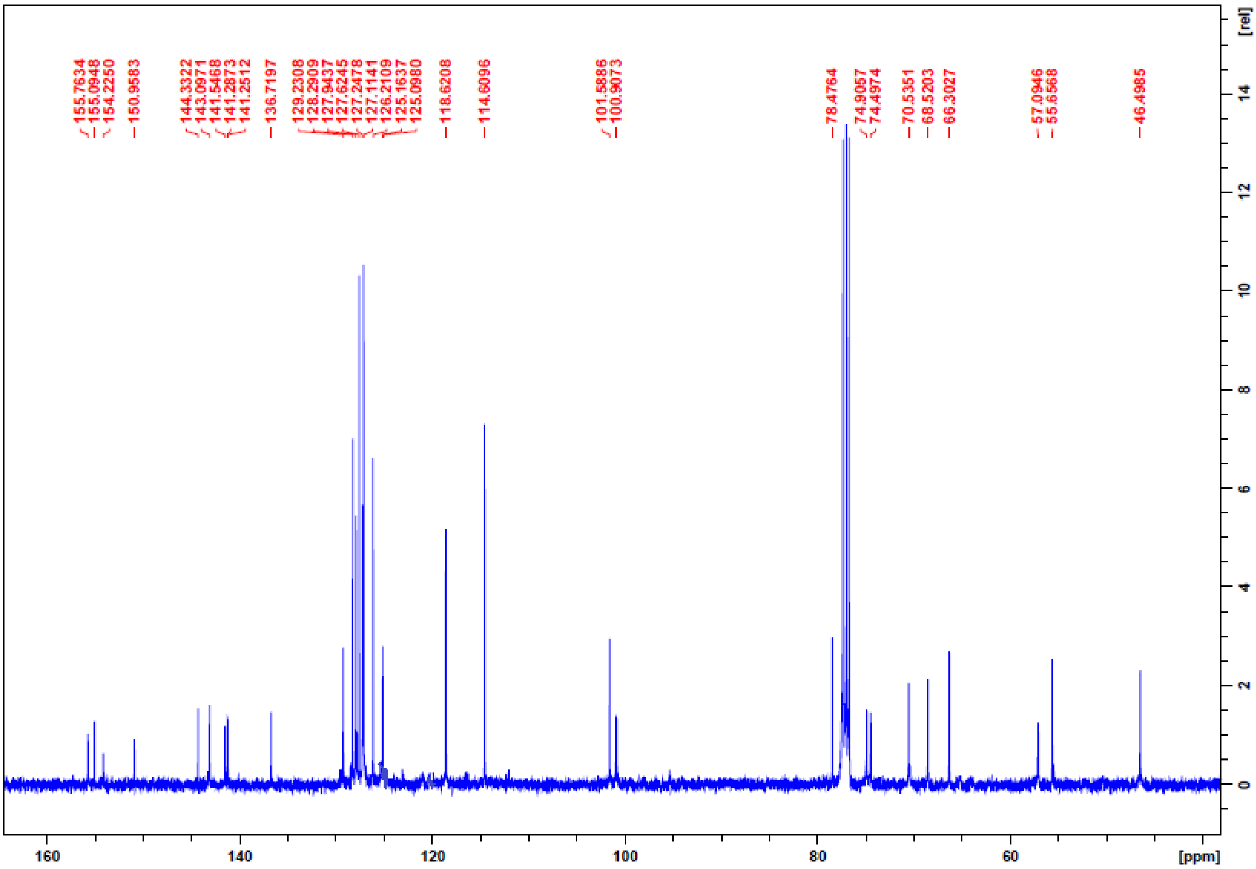

Compound 18: ^{1}H NMR, $CDCl_3$, 400 MHz

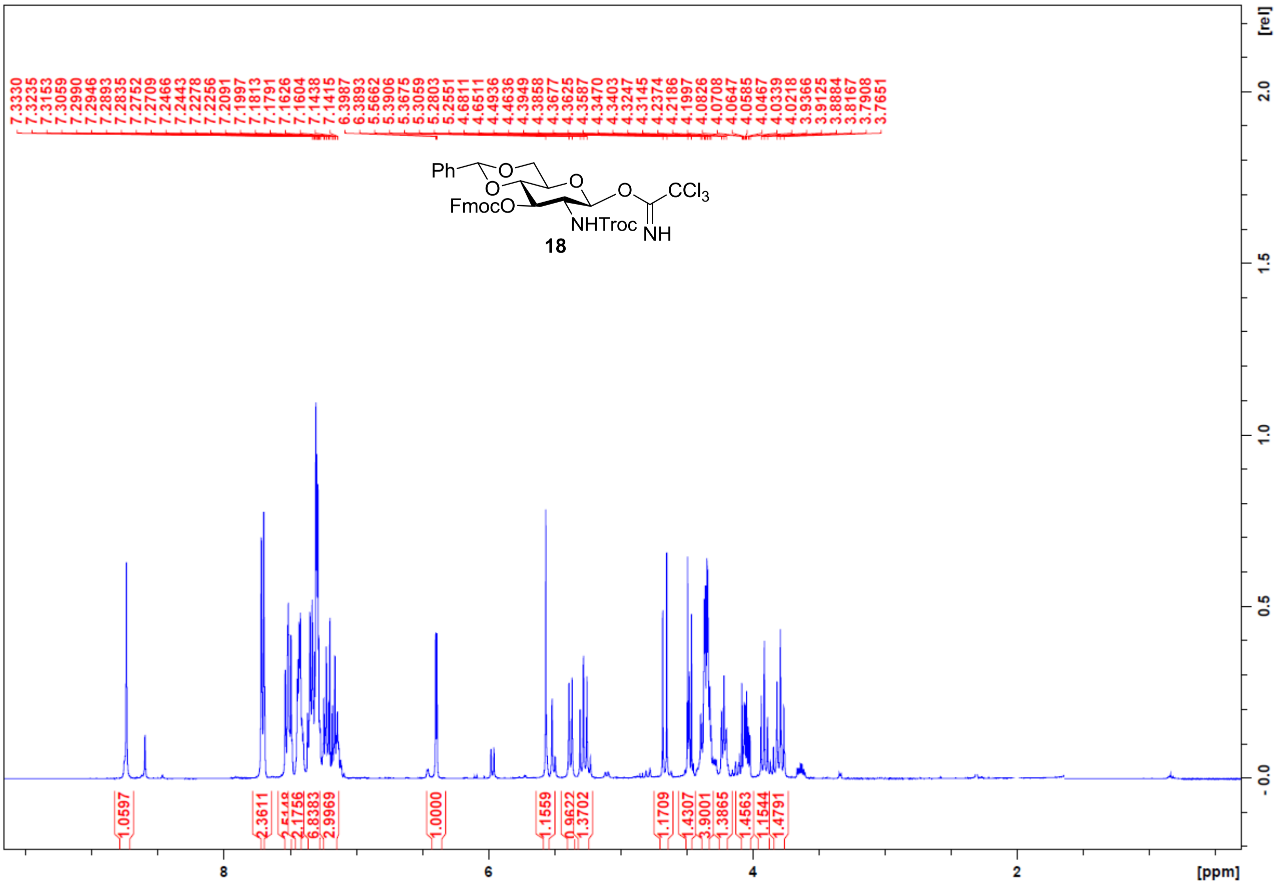

Compound 18: ^{13}C NMR, $CDCl_3$, 101 MHz

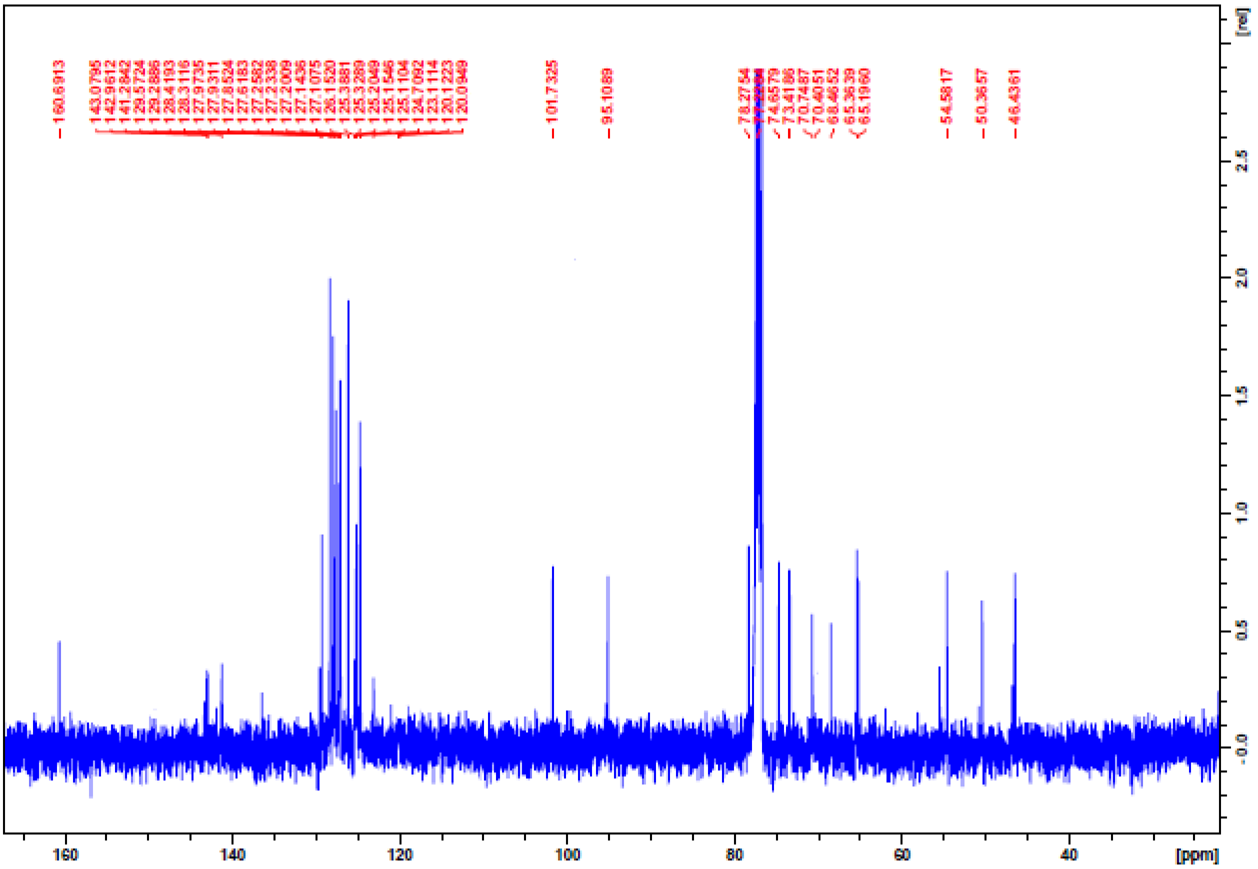

Compound 62: ^{1}H NMR, $CDCl_3$, 400 MHz

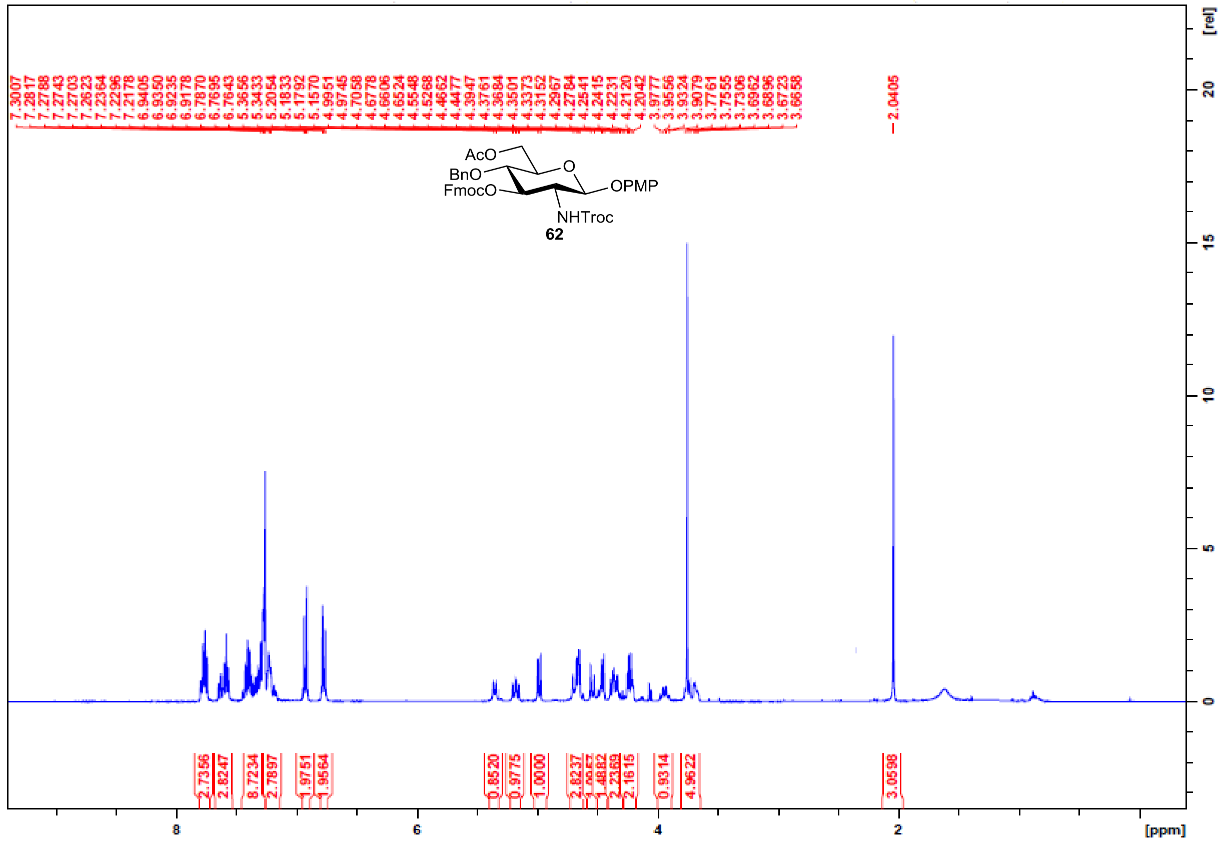

Compound 62: ^{13}C NMR, $CDCl_3$, 101 MHz

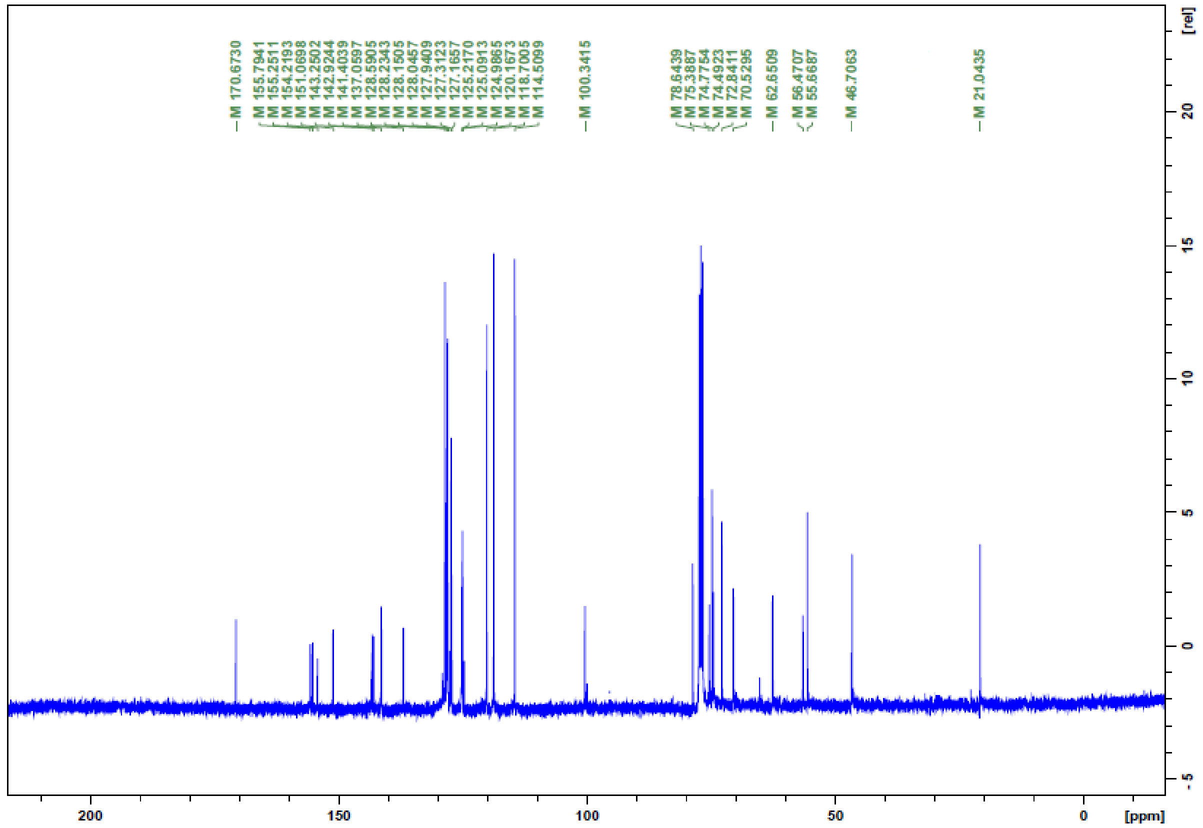

Compound 19: ^{1}H NMR, $CDCl_3$, 400 MHz

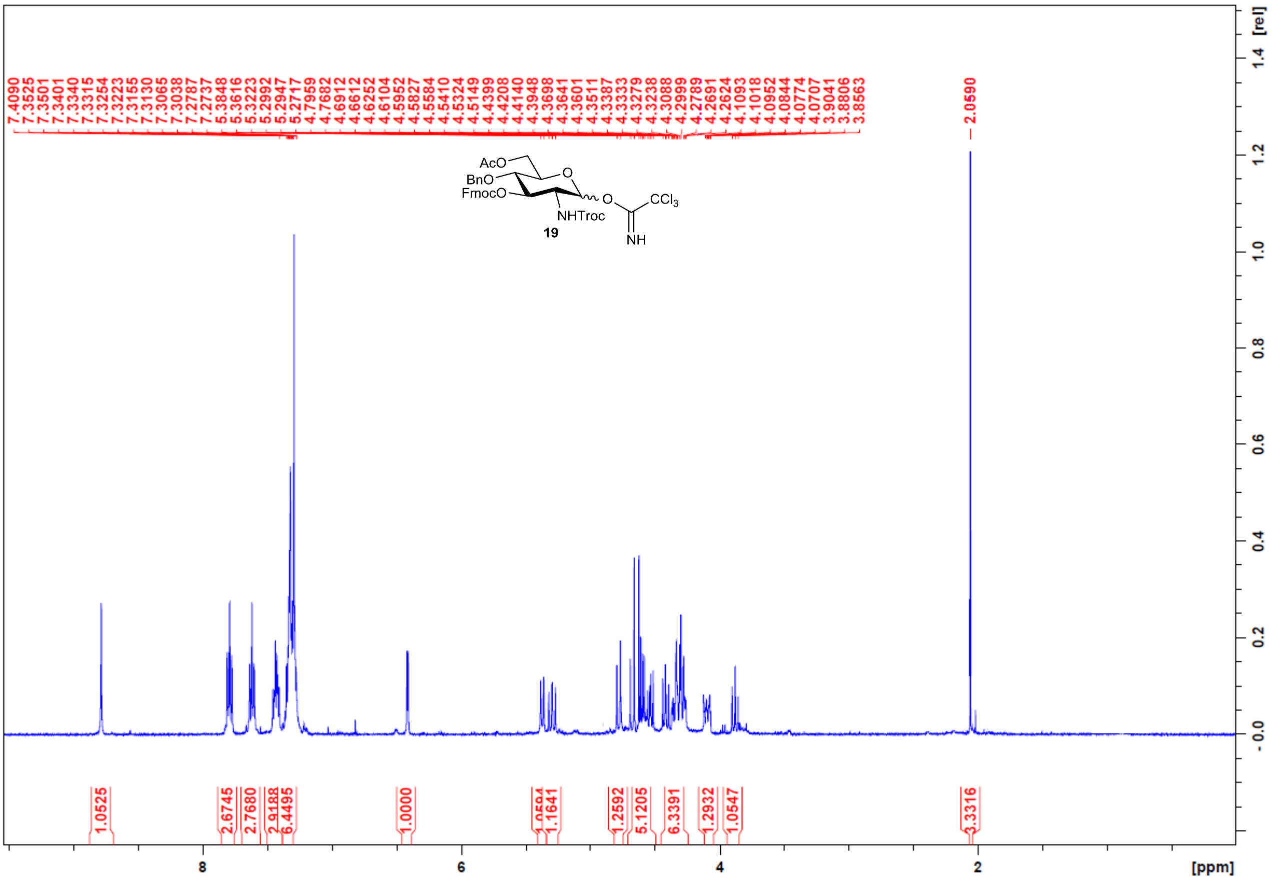

Compound 19: ^{13}C NMR, $CDCl_3$, 101 MHz

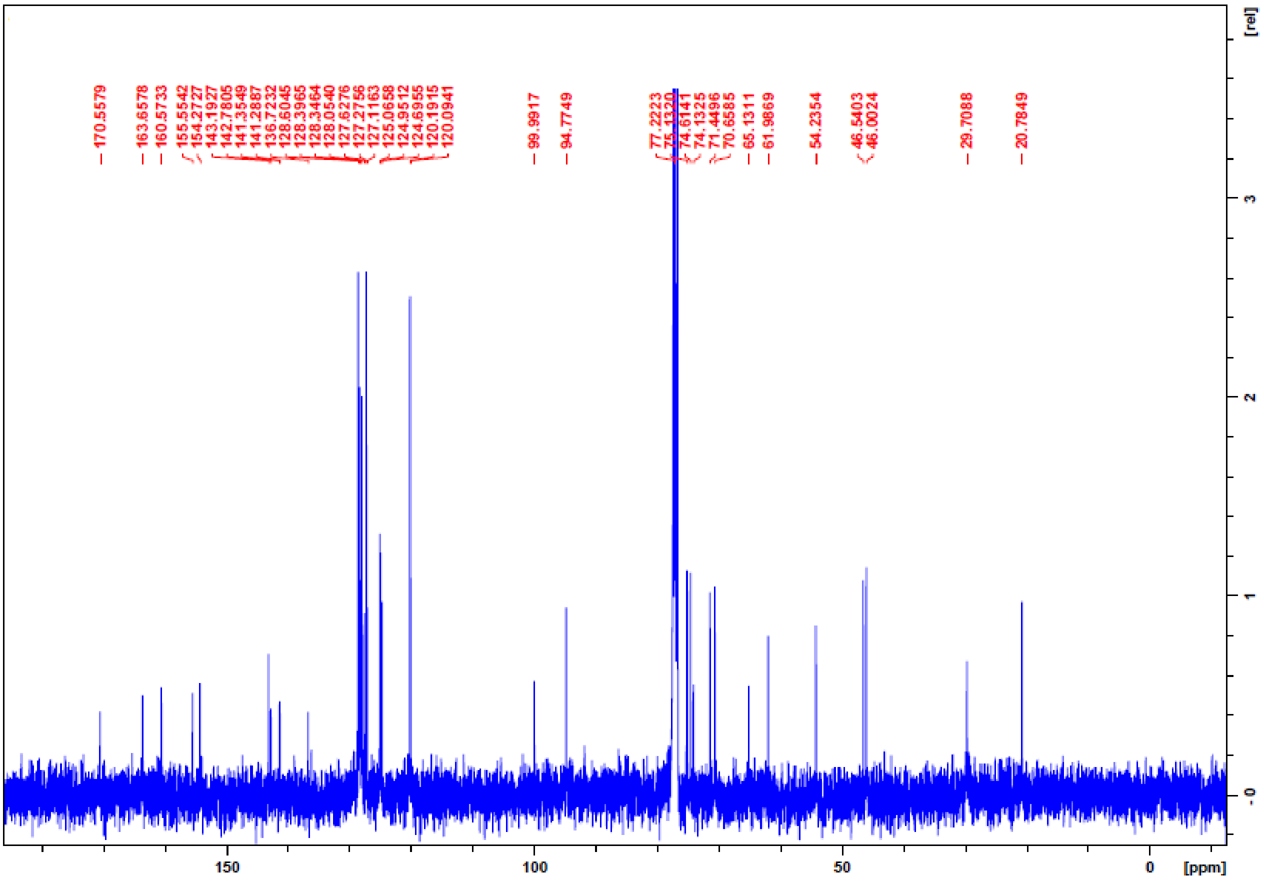

Compound 20: ^{1}H NMR, $CDCl_3$, 400 MHz

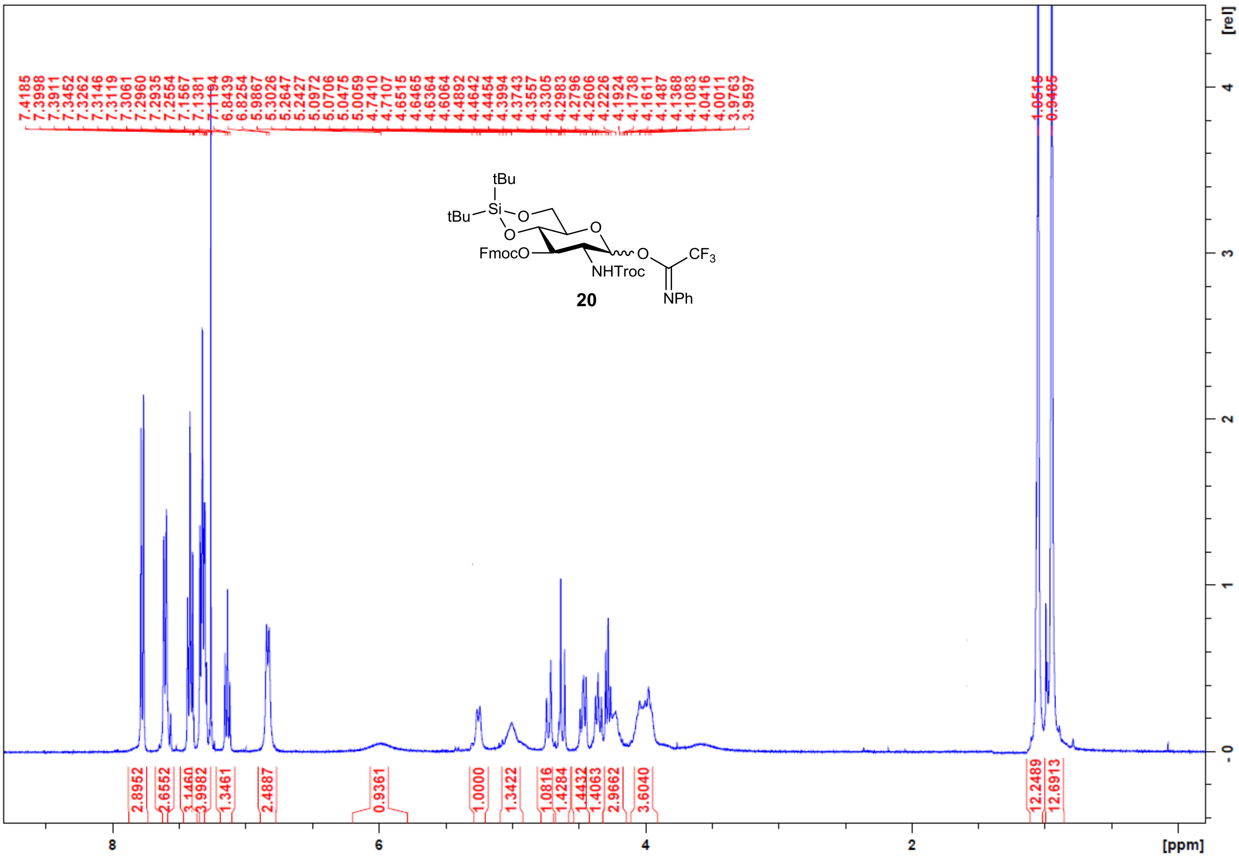

Compound 20: ^{13}C NMR, $CDCl_3$, 101 MHz

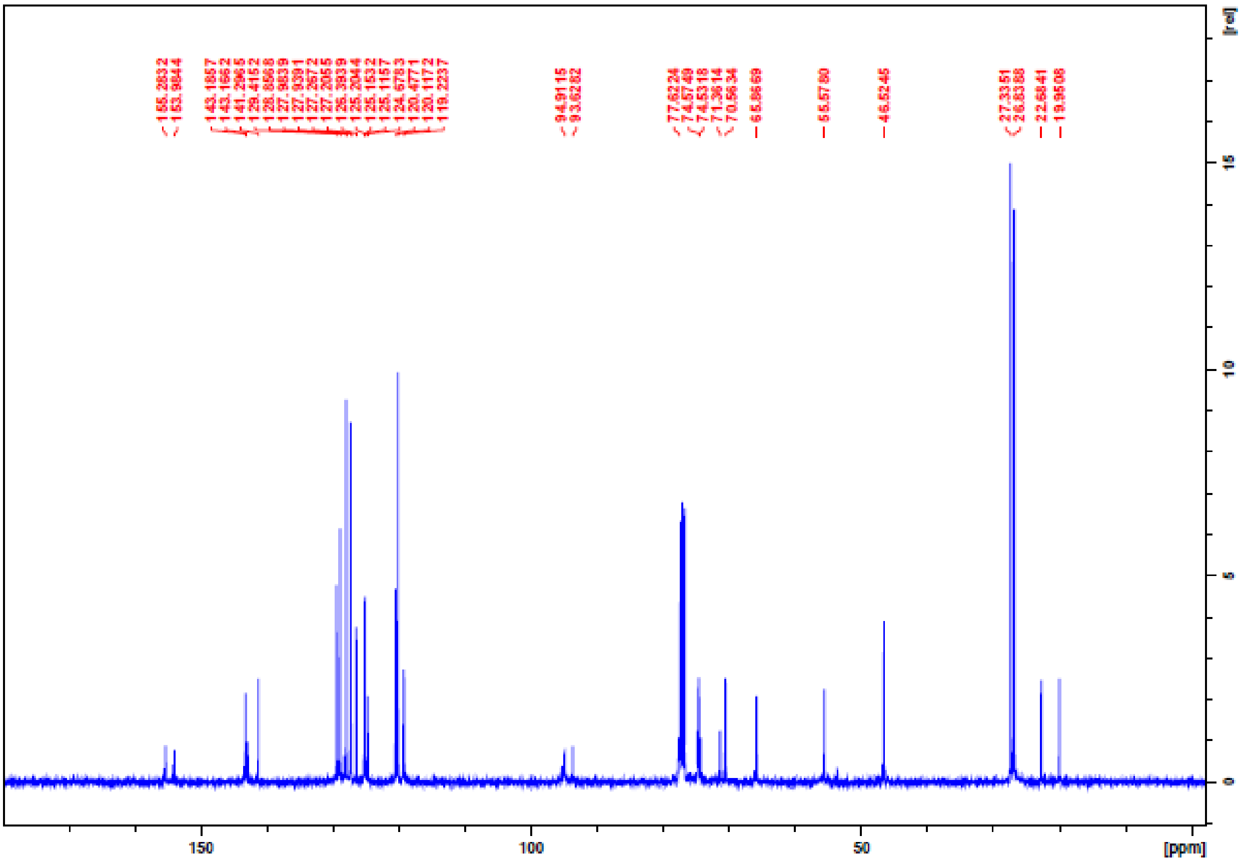

Compound 10: ^{1}H NMR, $CDCl_3$, 400 MHz

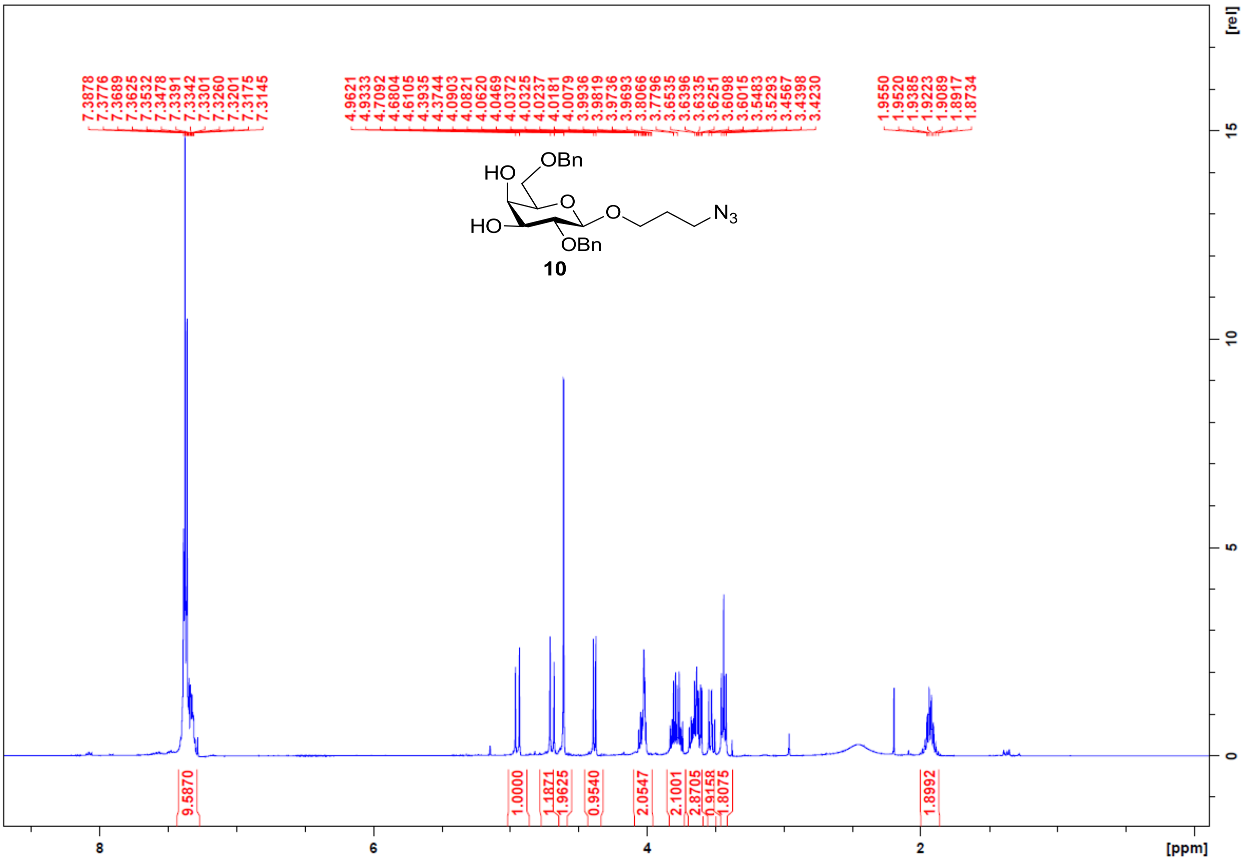

Compound 10: ^{13}C NMR, $CDCl_3$, 101 MHz

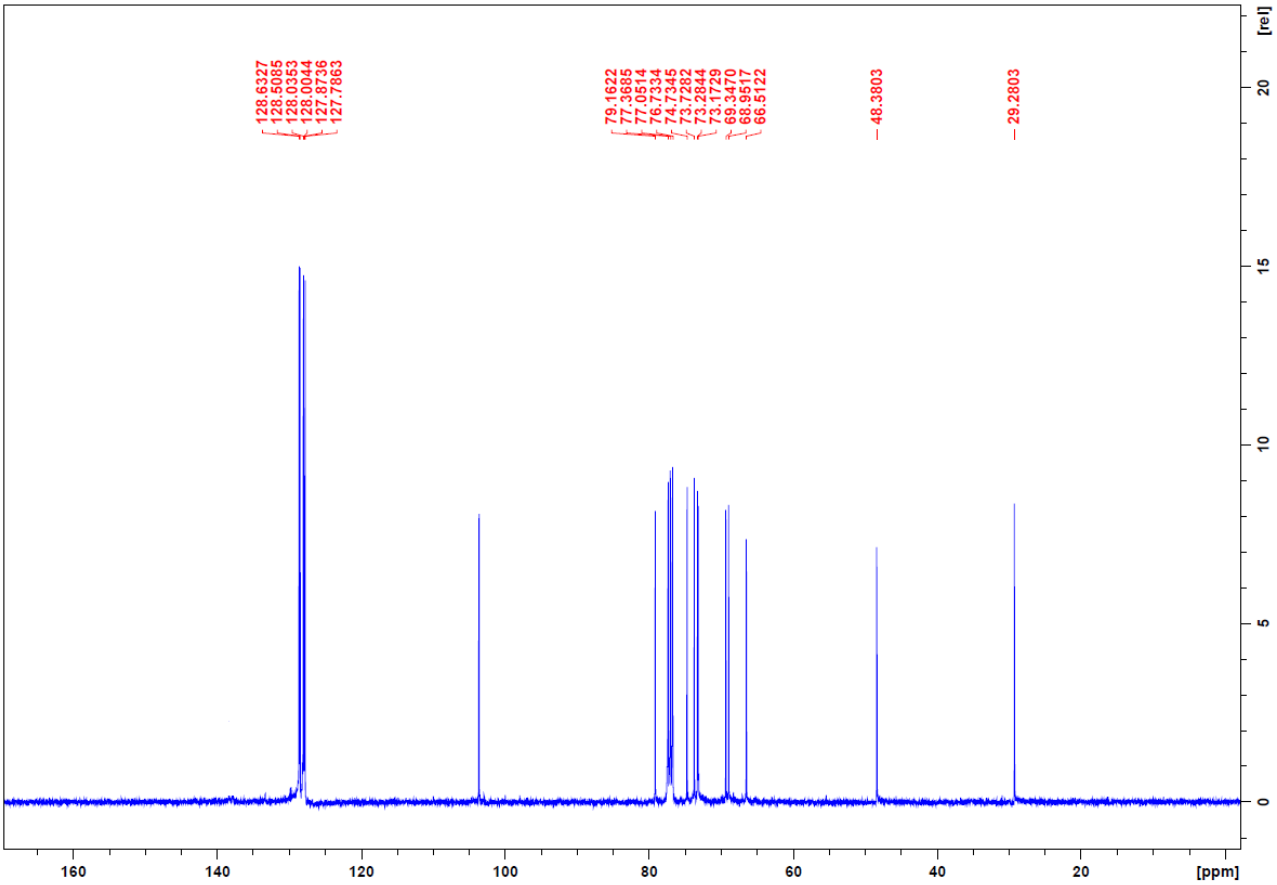

Compound 11: ^{1}H NMR, $CDCl_3$, 400 MHz

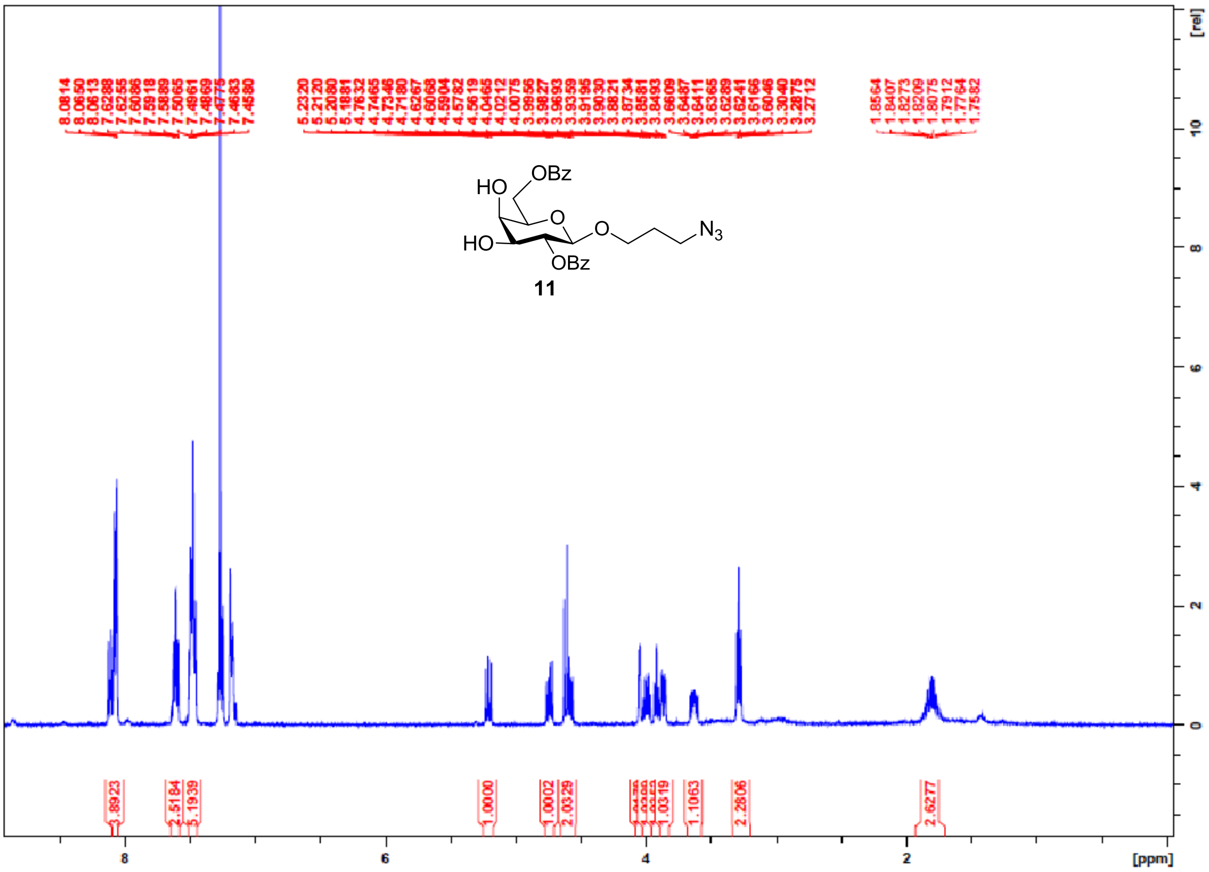

Compound 11: ^{13}C NMR, $CDCl_3$, 101 MHz

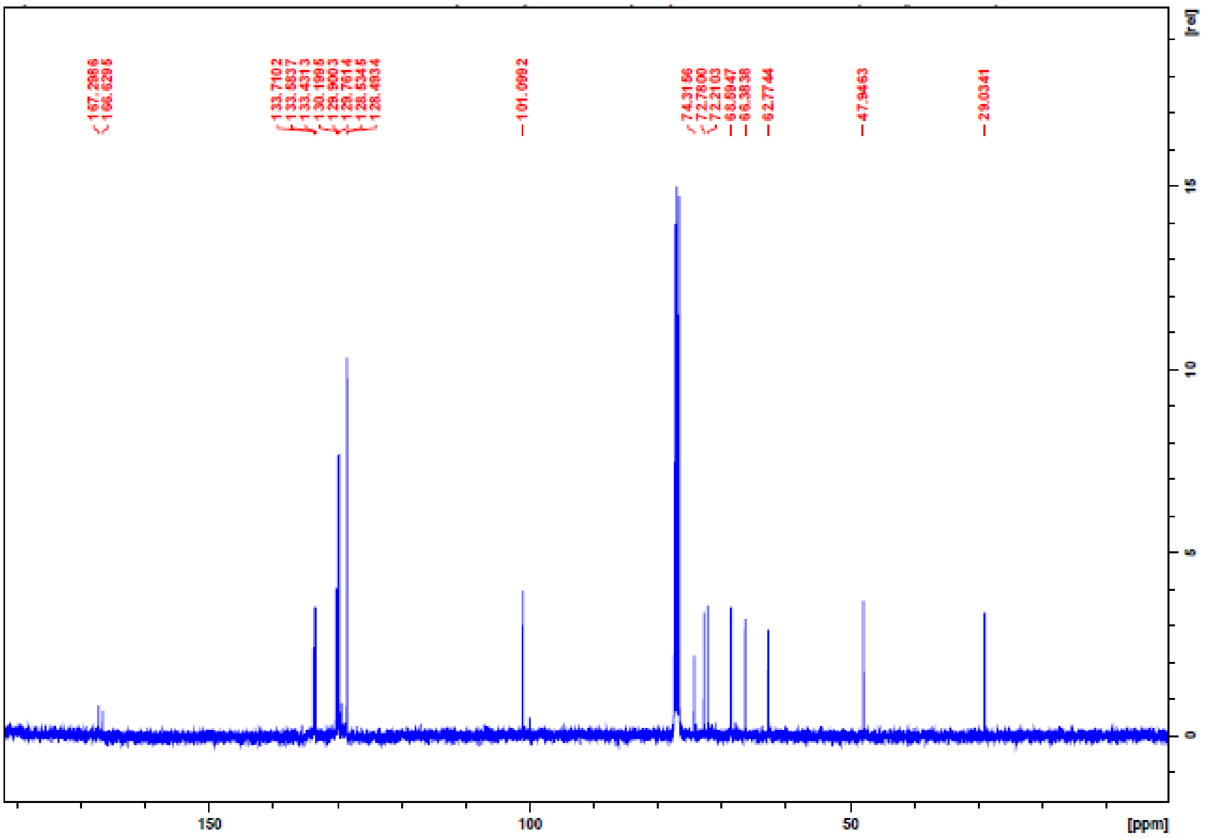

Compound 71: ^{1}H NMR, $CDCl_3$, 400 MHz

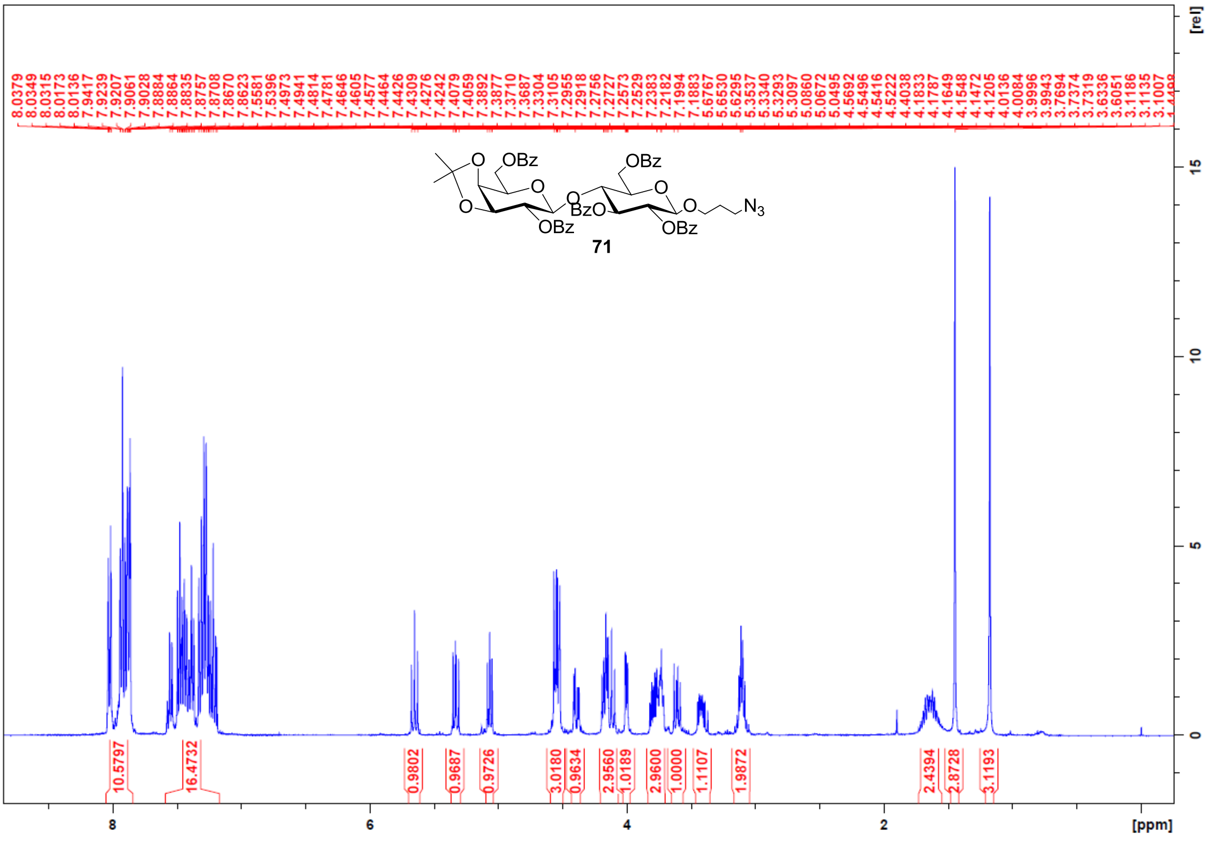

Compound 71: ^{13}C NMR, $CDCl_3$, 101 MHz

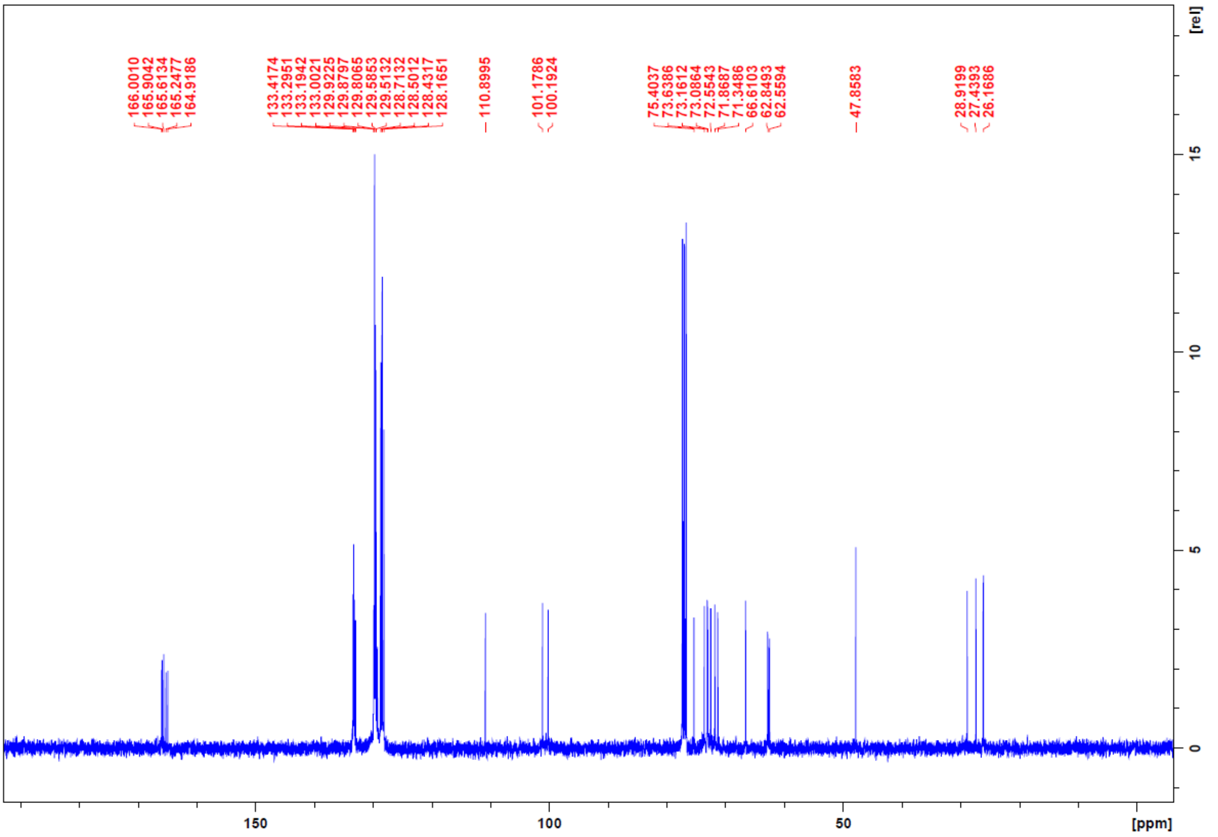

Compound 33: ^{1}H NMR, CDCl$_3$, 400 MHz

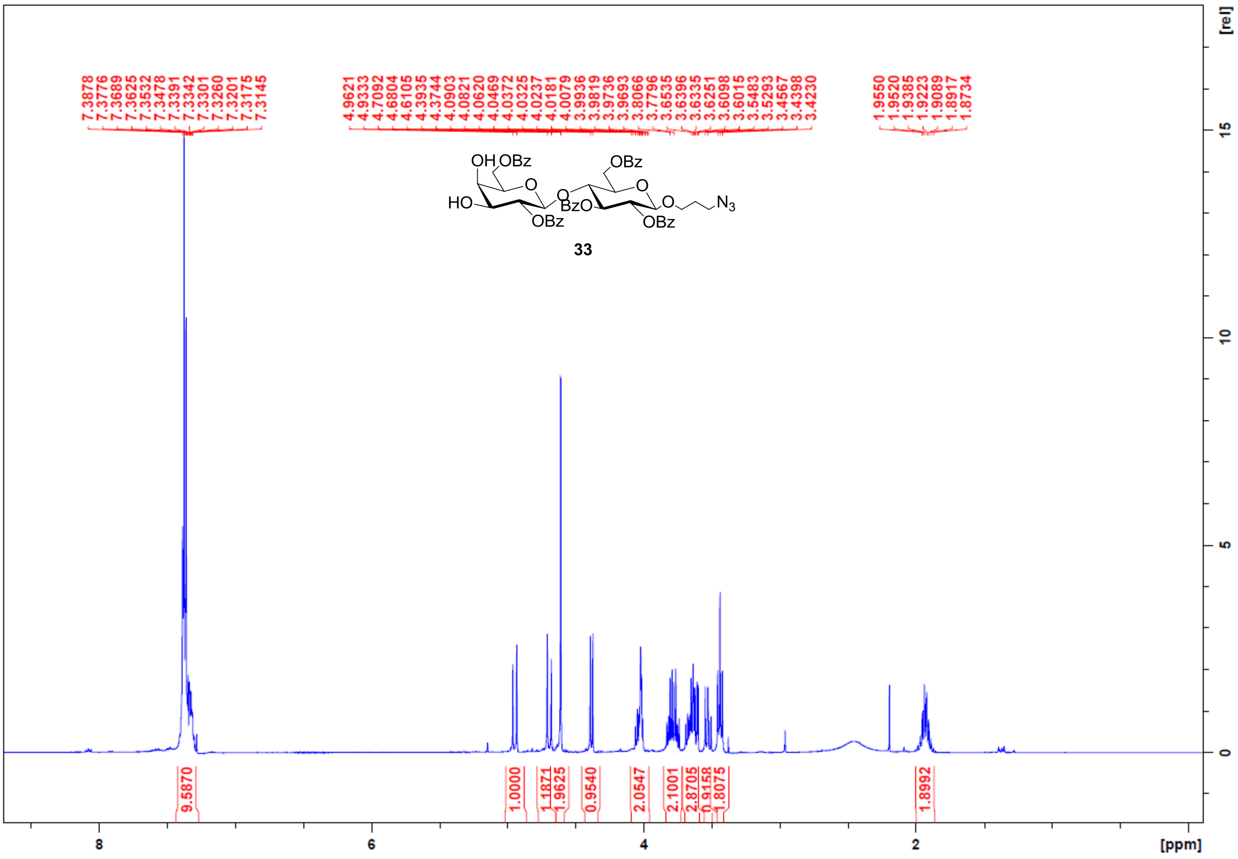

Compound 33: ^{13}C NMR, CDCl$_3$, 101 MHz

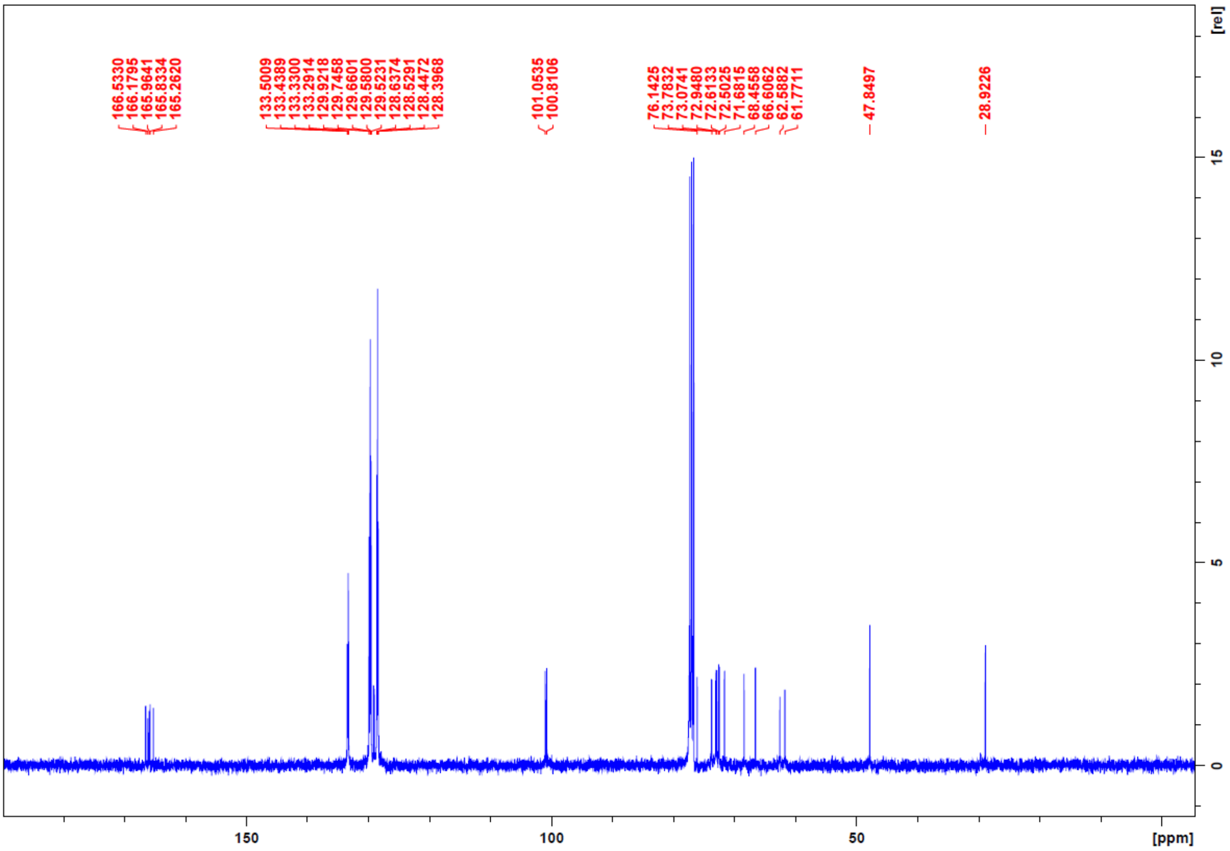

Compound 73: ^{1}H NMR, $CDCl_3$, 400 MHz

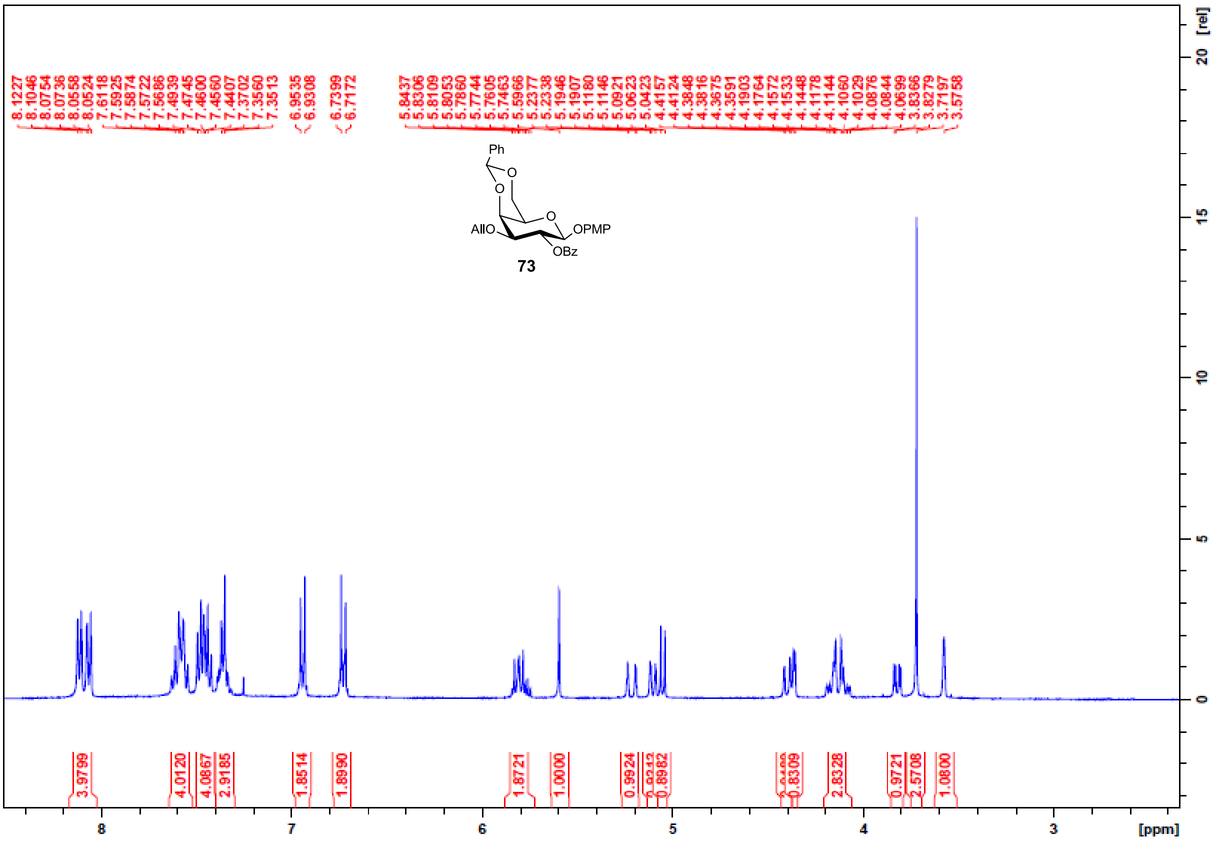

Compound 73: ^{13}C NMR, $CDCl_3$, 101 MHz

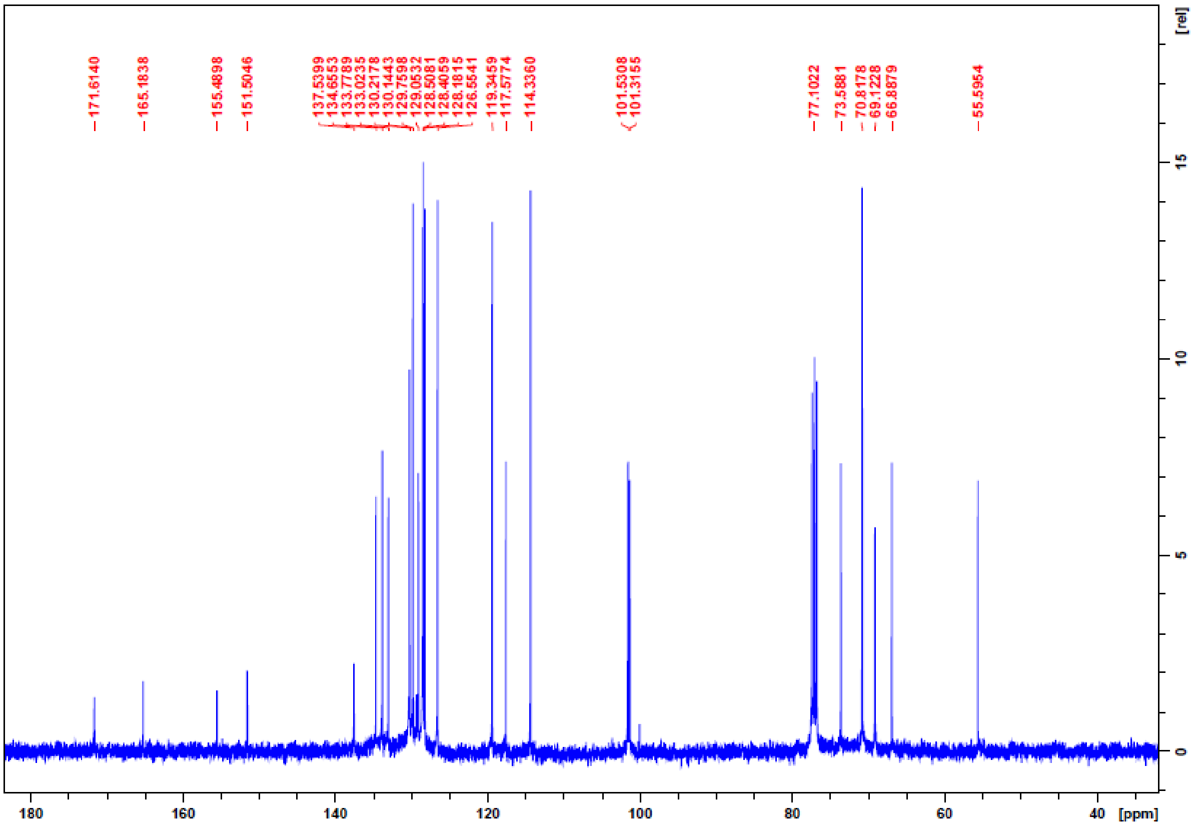

Compound 38: ^{1}H NMR, $CDCl_3$, 400 MHz

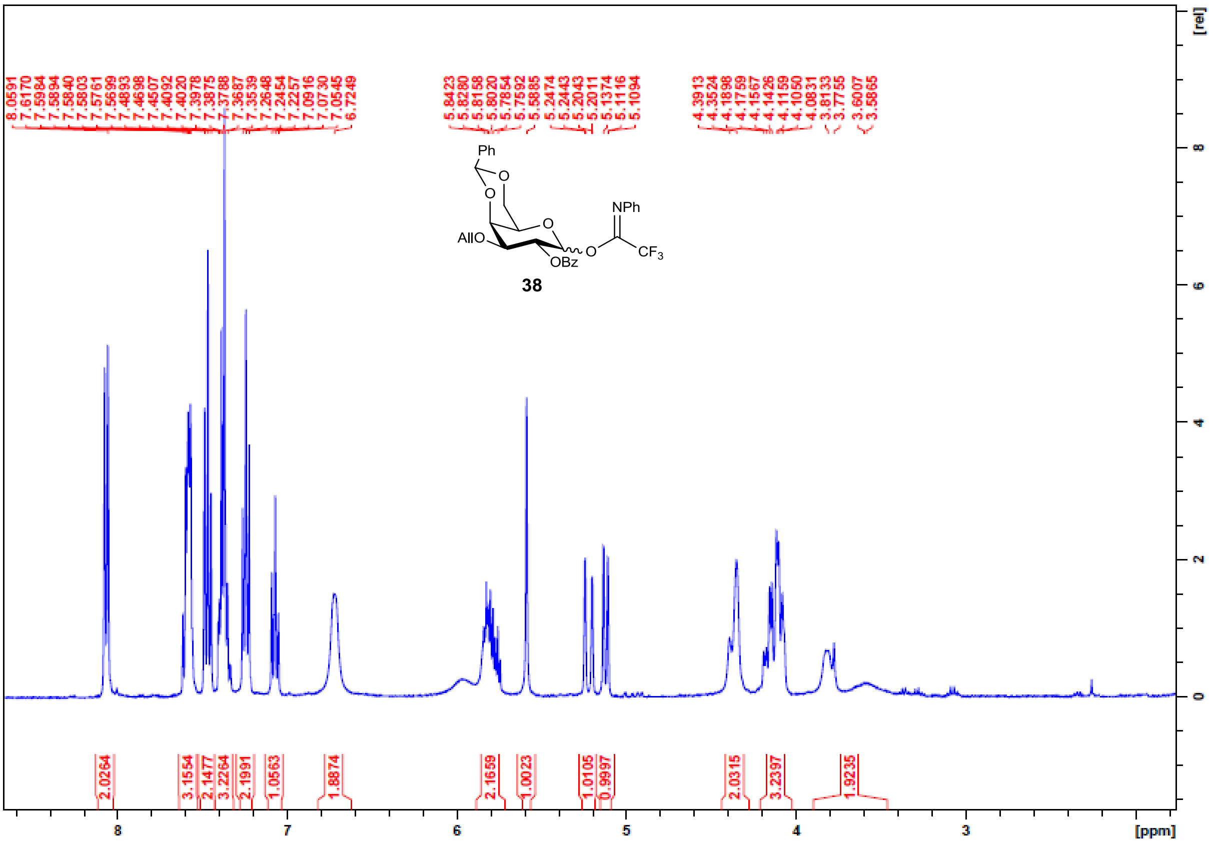

38

Compound 38: ^{13}C NMR, $CDCl_3$, 101 MHz

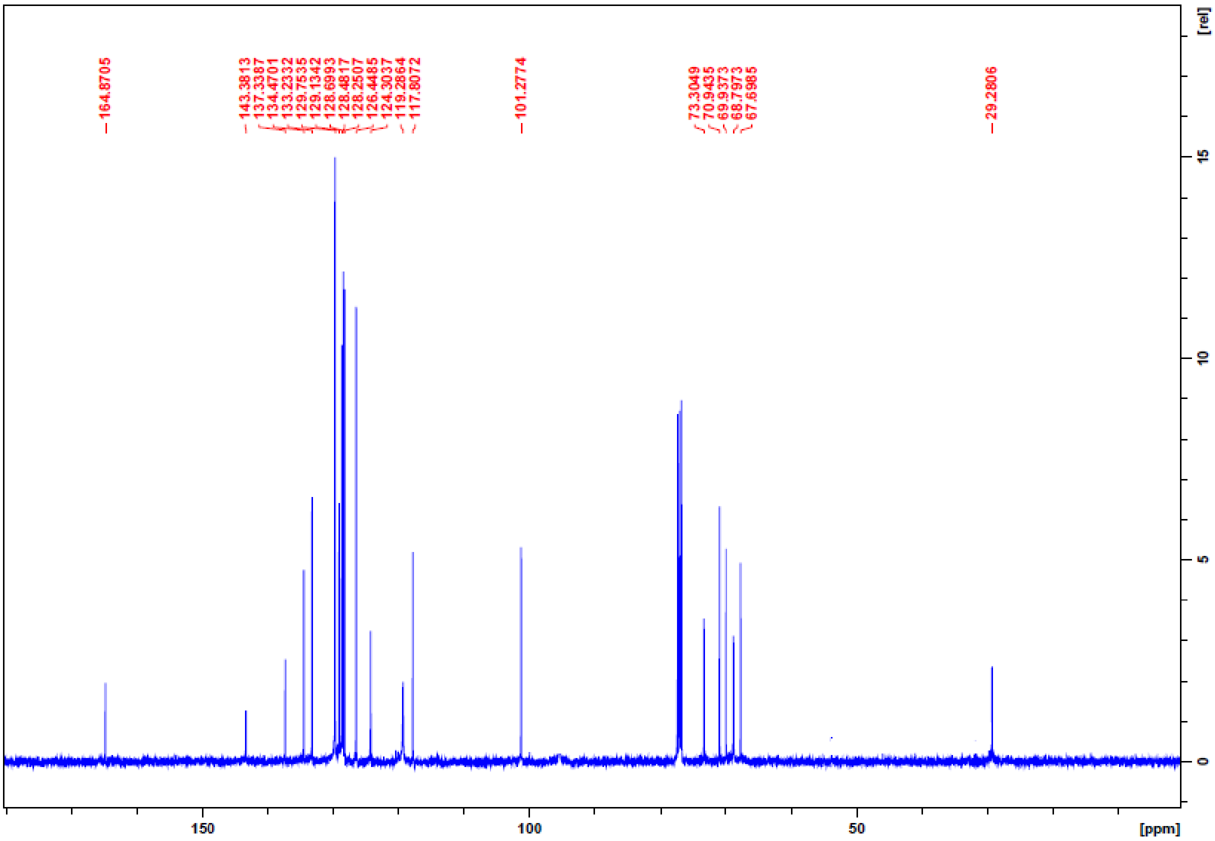

Compound 12a: ^{1}H NMR, CDCl$_3$, 400 MHz

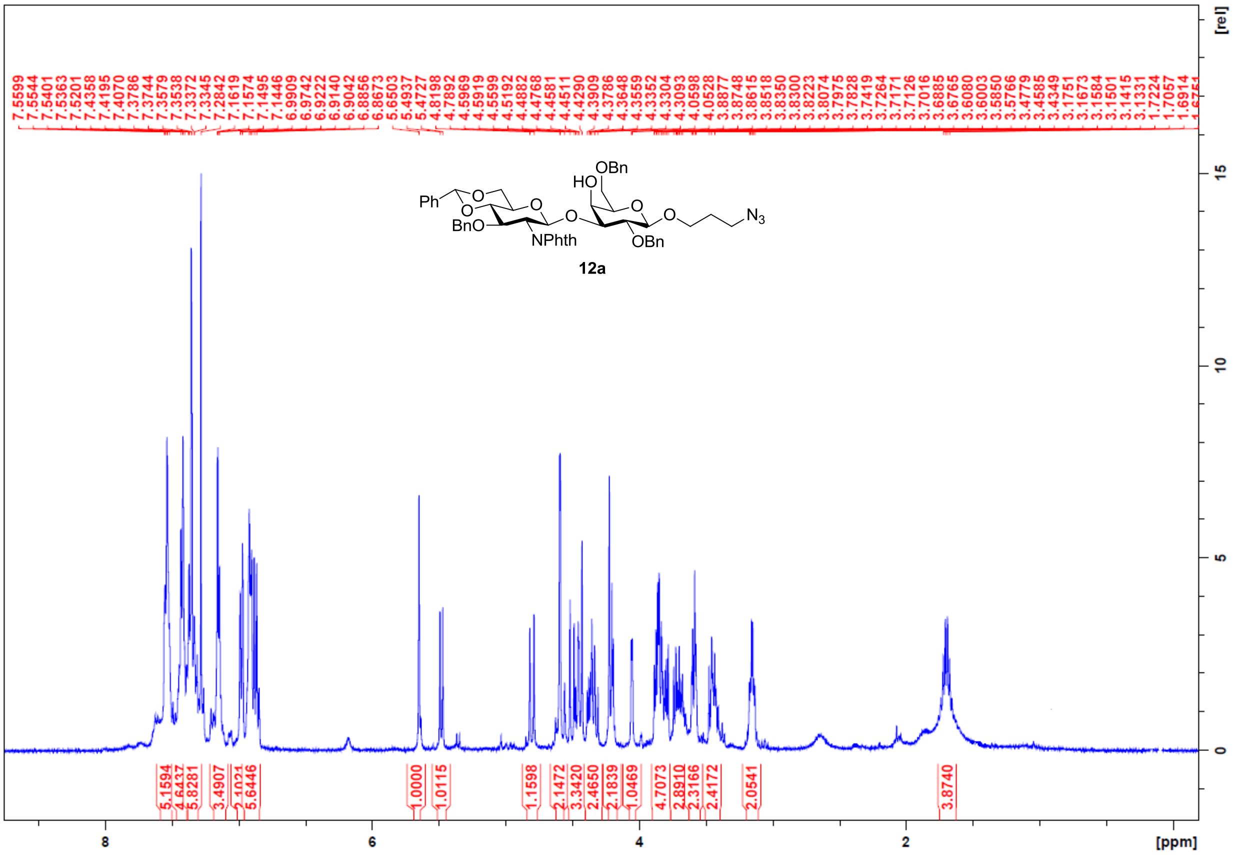

Compound 12a: ^{13}C NMR, CDCl$_3$, 101 MHz

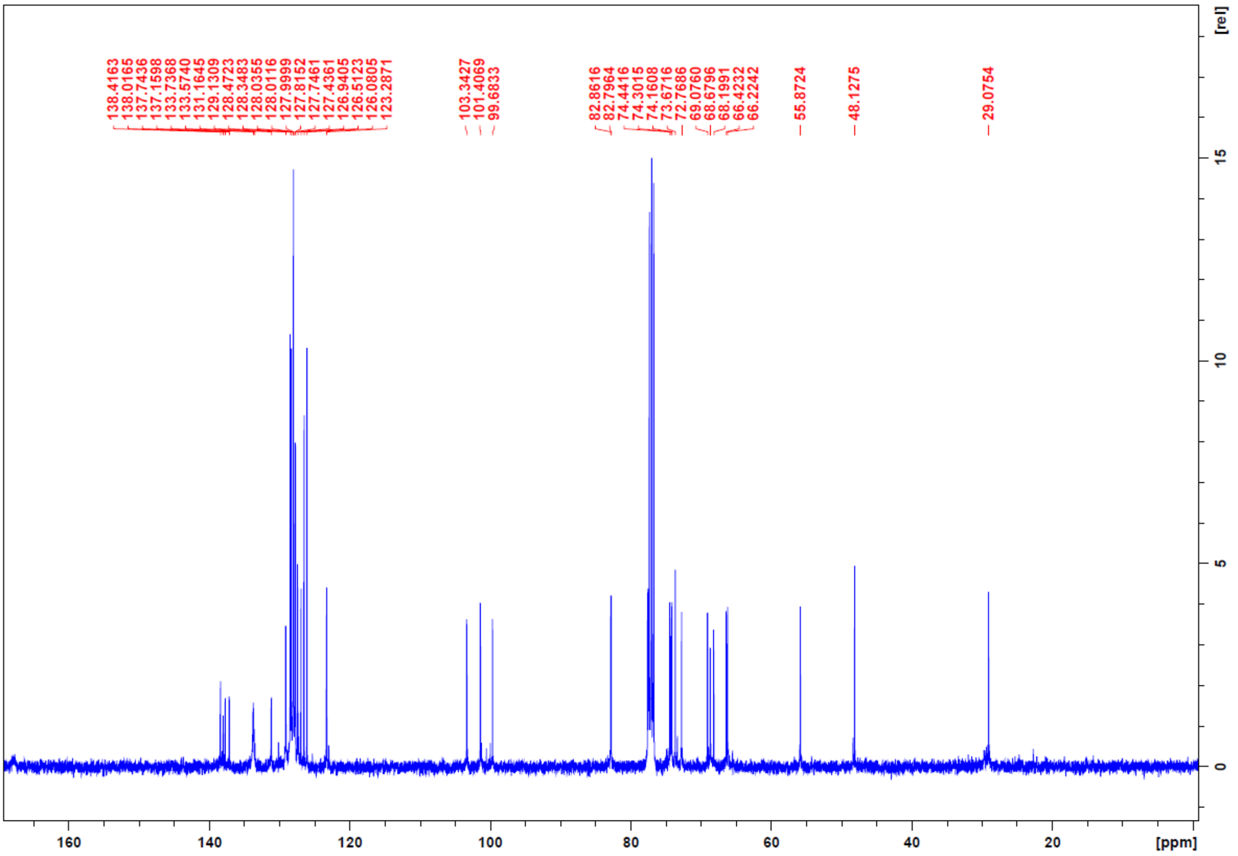

Compound 13a: ^{1}H NMR, $CDCl_3$, 400 MHz

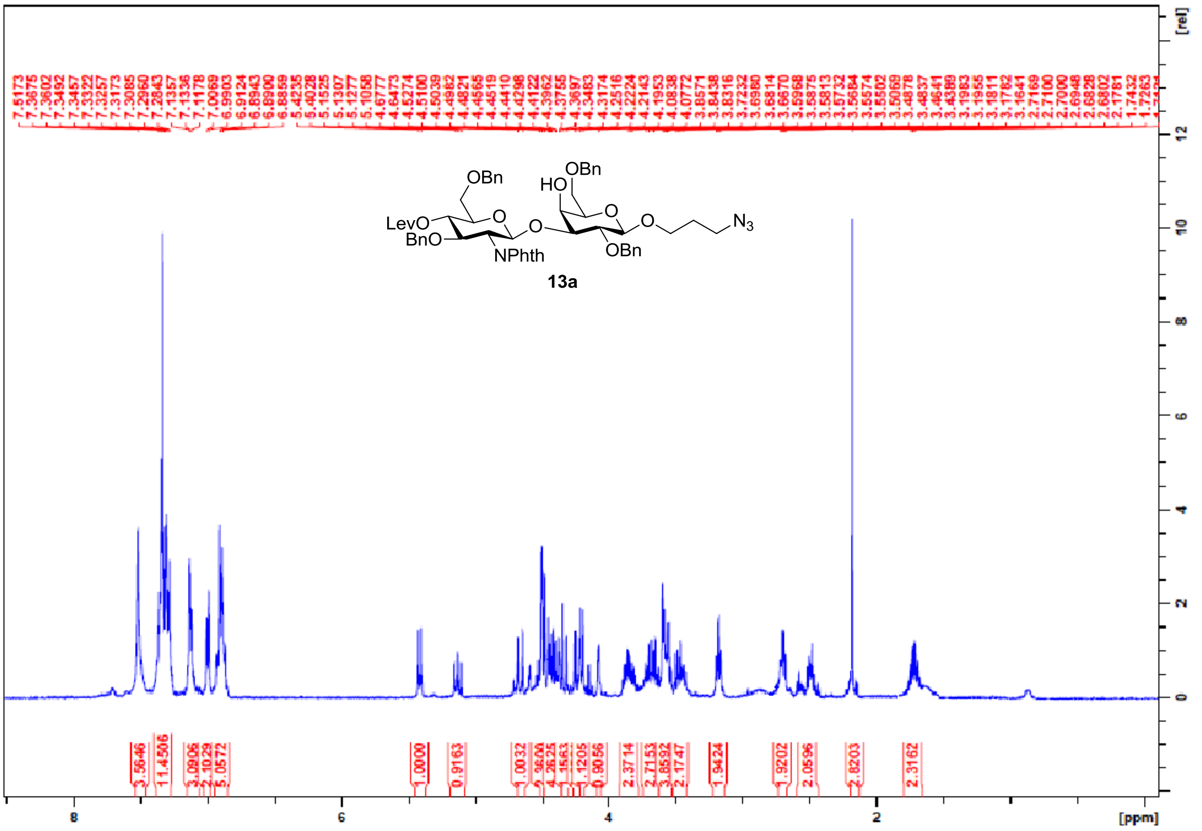

Compound 13a: ^{13}C NMR, $CDCl_3$, 101 MHz

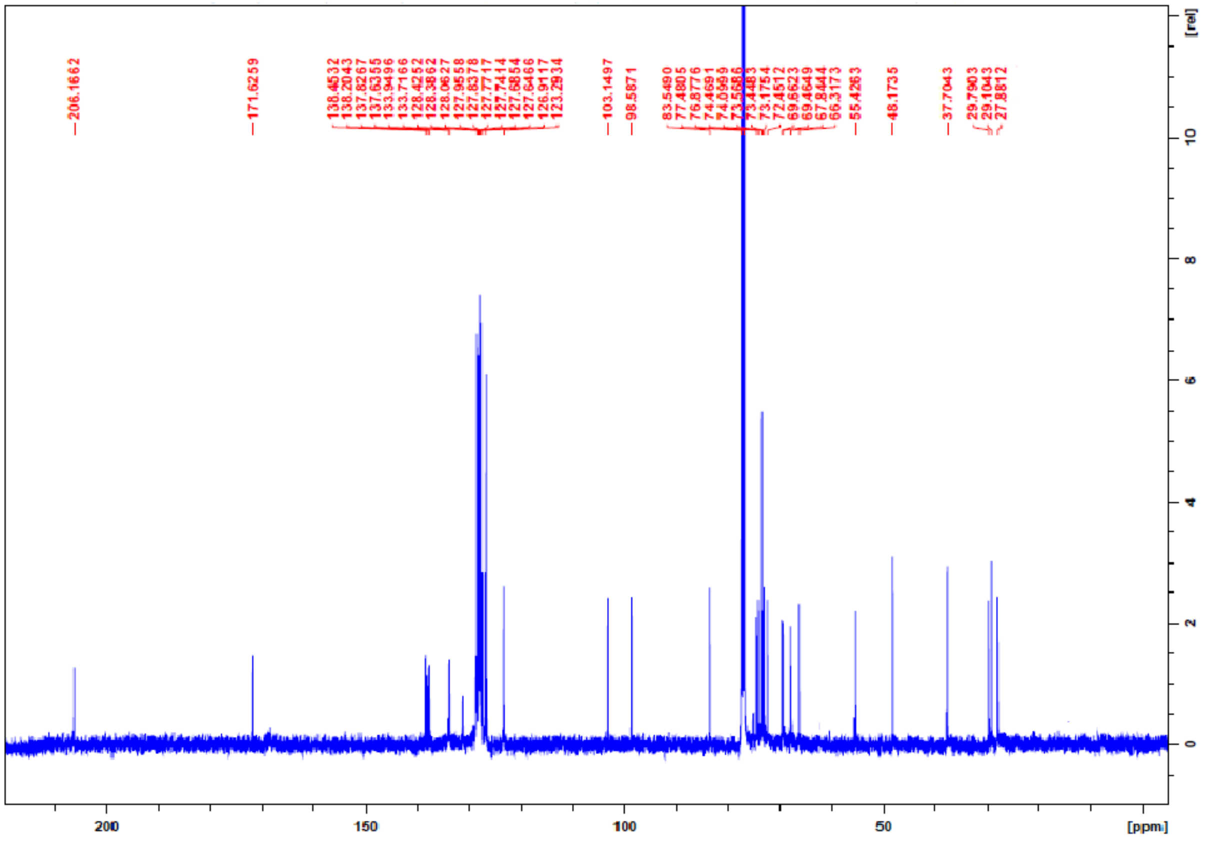

Compound 13b: ^{1}H NMR, CDCl$_3$, 400 MHz

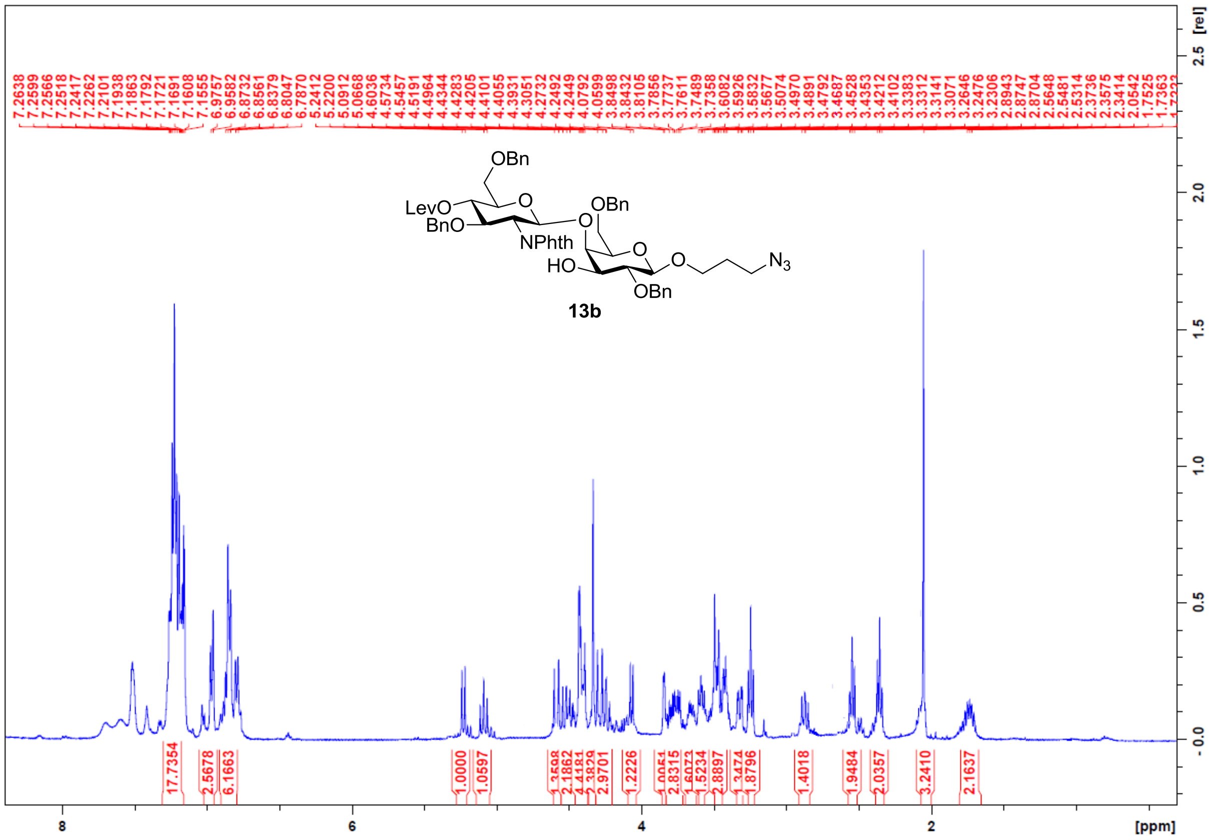

Compound 13b: ^{13}C NMR, CDCl$_3$, 101 MHz

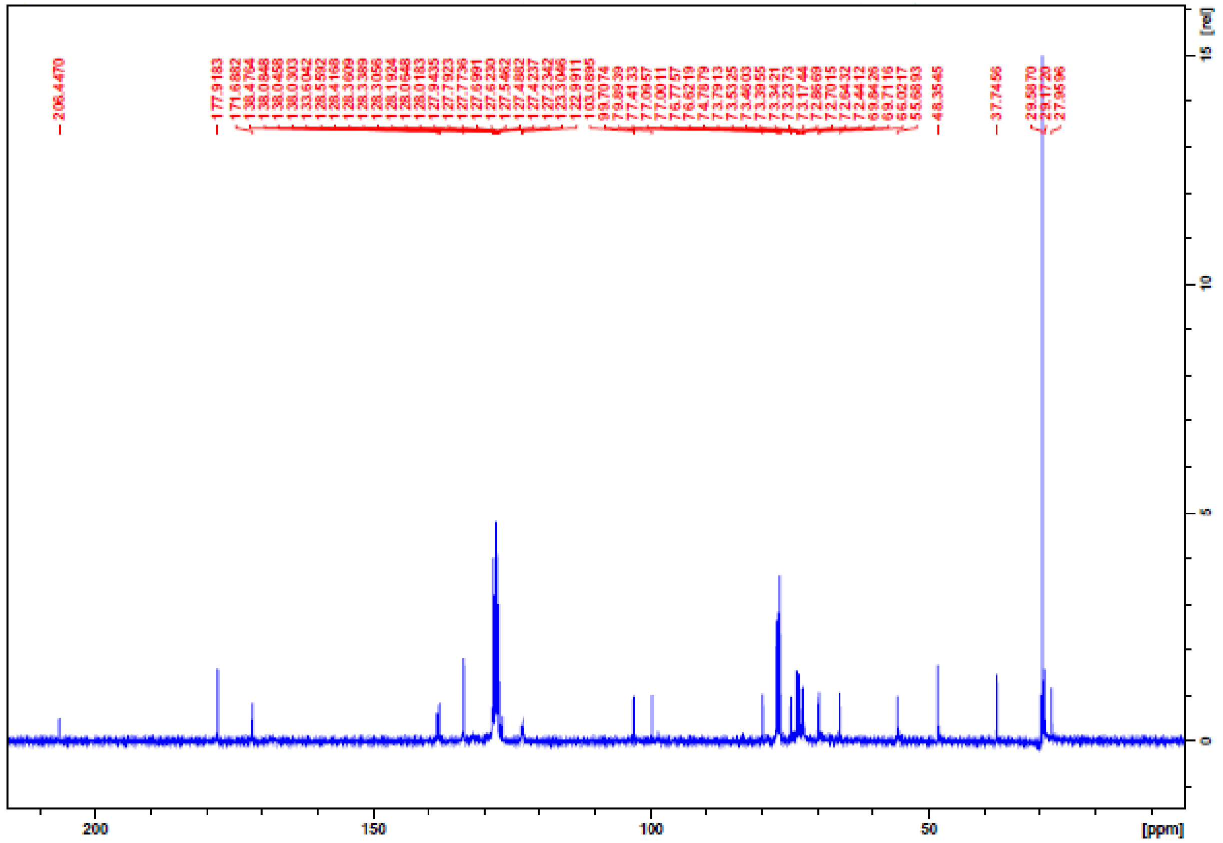

Compound 14a: ^{1}H NMR, $CDCl_3$, 400 MHz

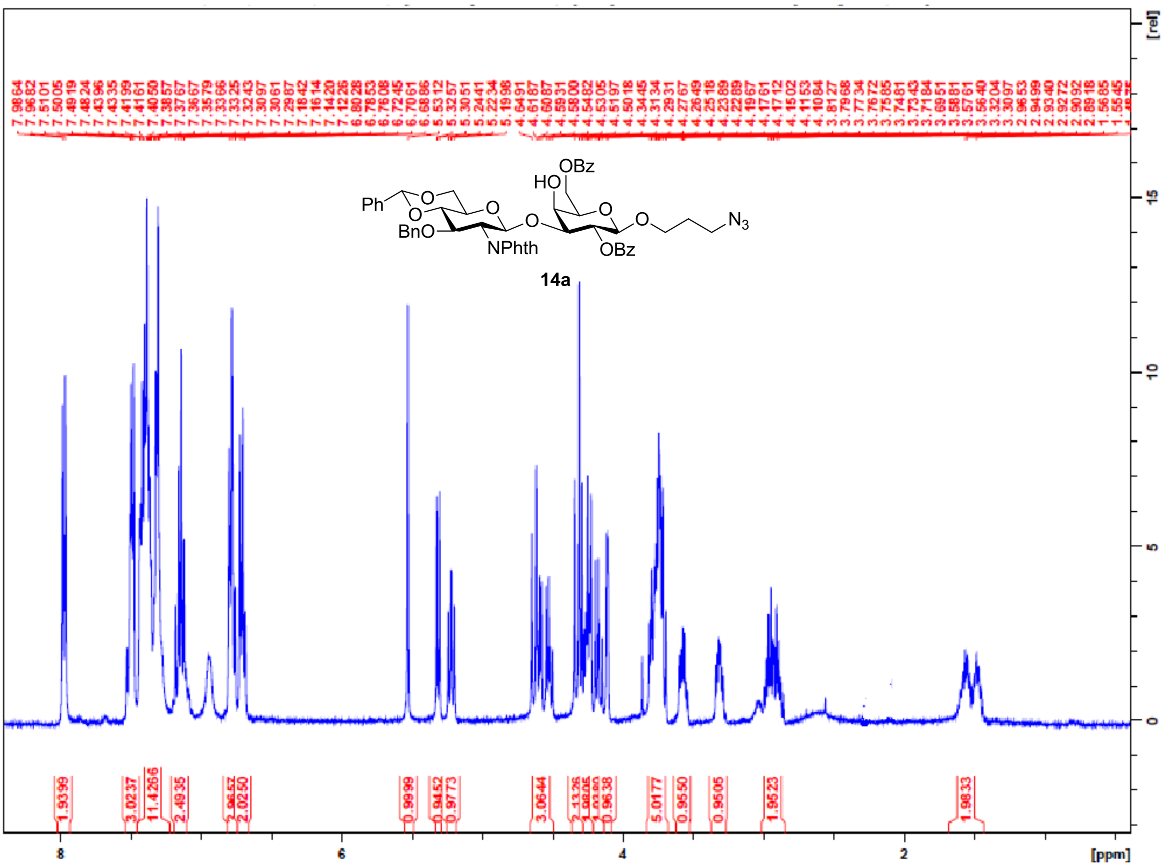

Compound 14a: ^{13}C NMR, $CDCl_3$, 101 MHz

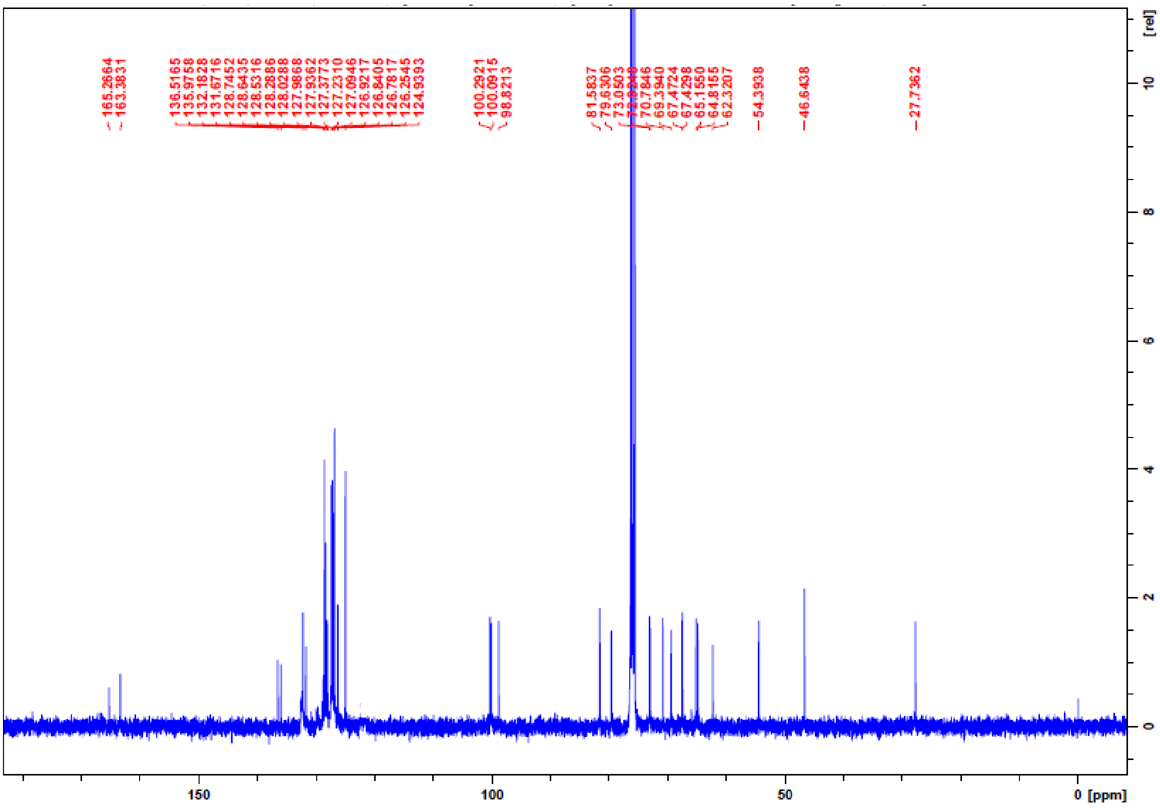

Compound 15a: ^{1}H NMR, $CDCl_3$, 400 MHz

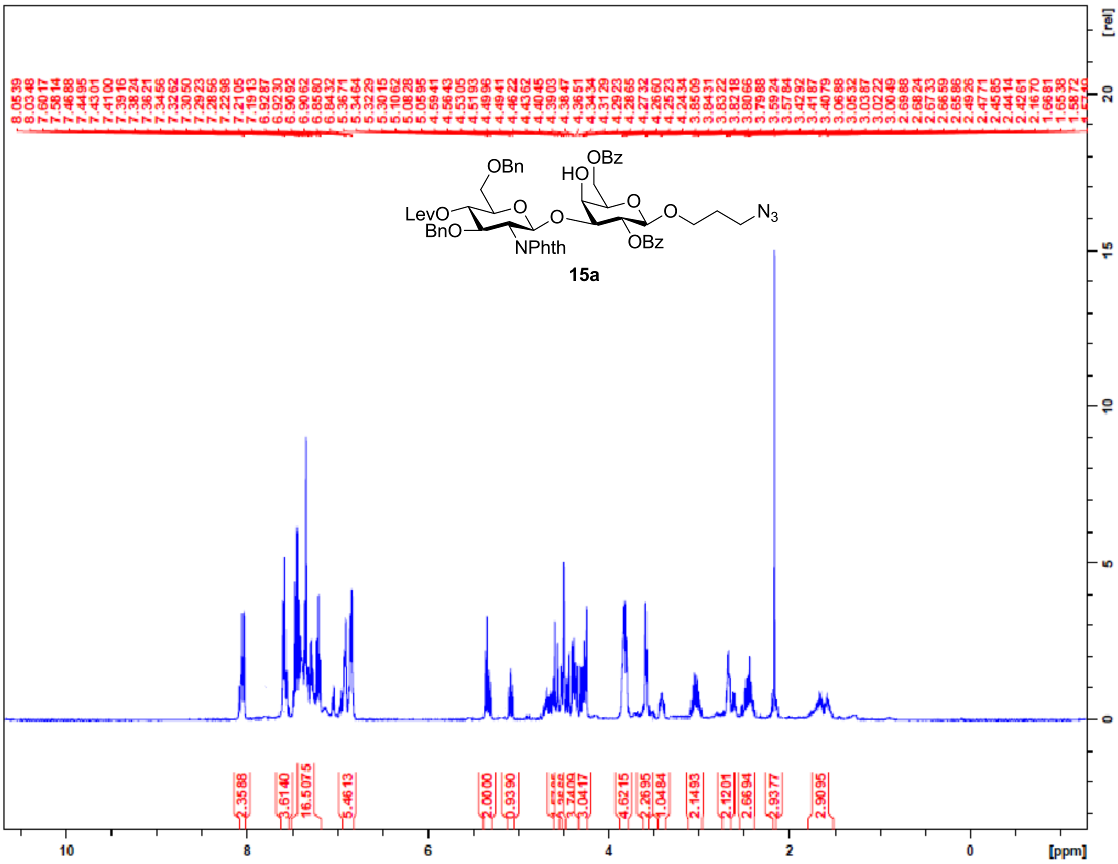

Compound 15a: ^{13}C NMR, $CDCl_3$, 101 MHz

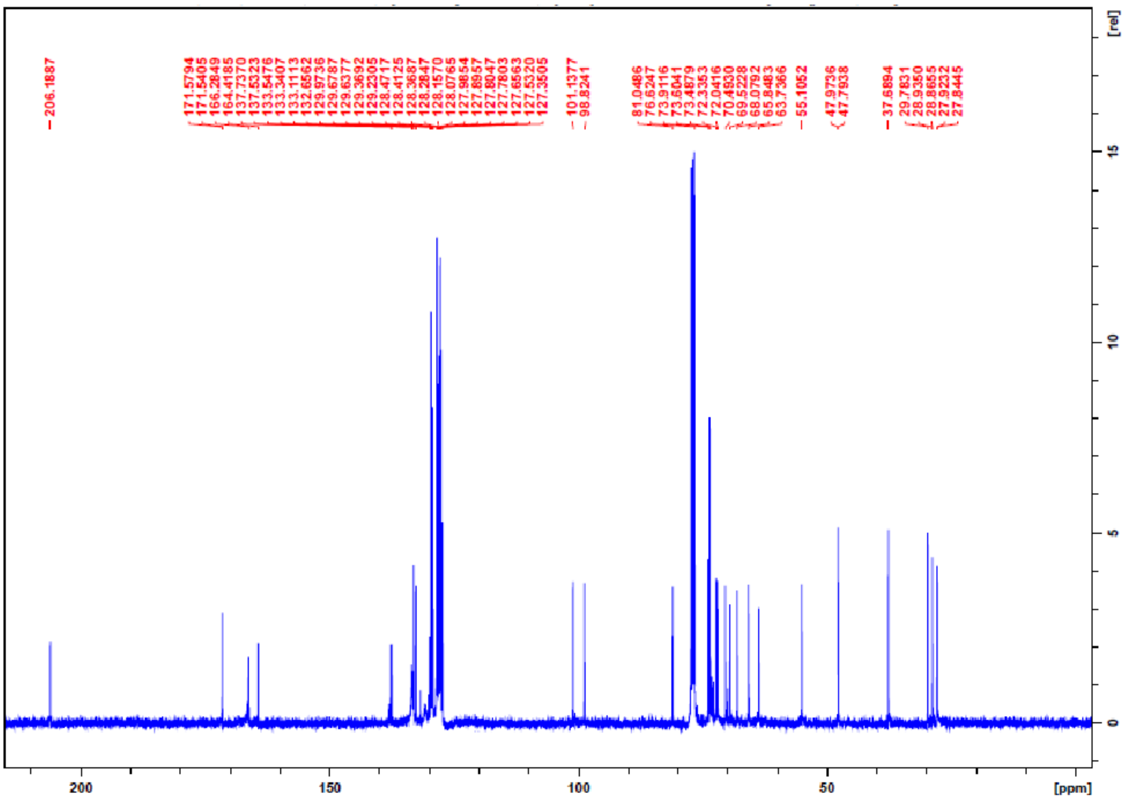

Compound 21a: ^{1}H NMR, CDCl$_3$, 400 MHz

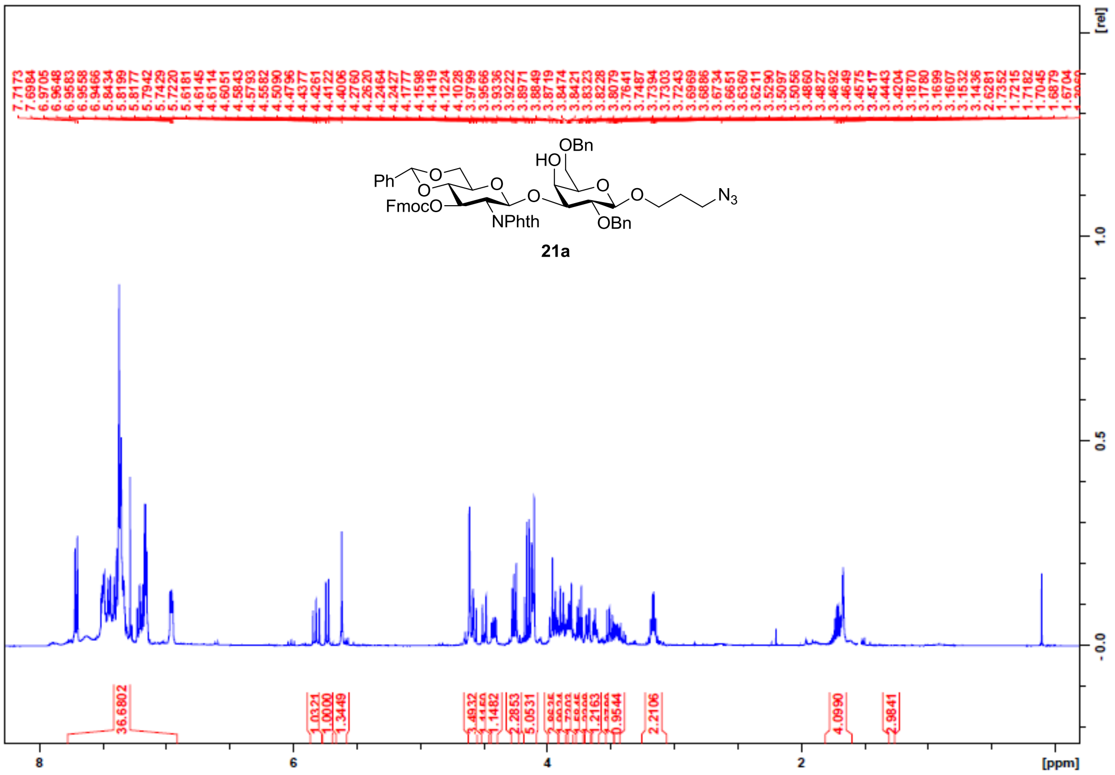

Compound 21a: ^{13}C NMR, CDCl$_3$, 101 MHz

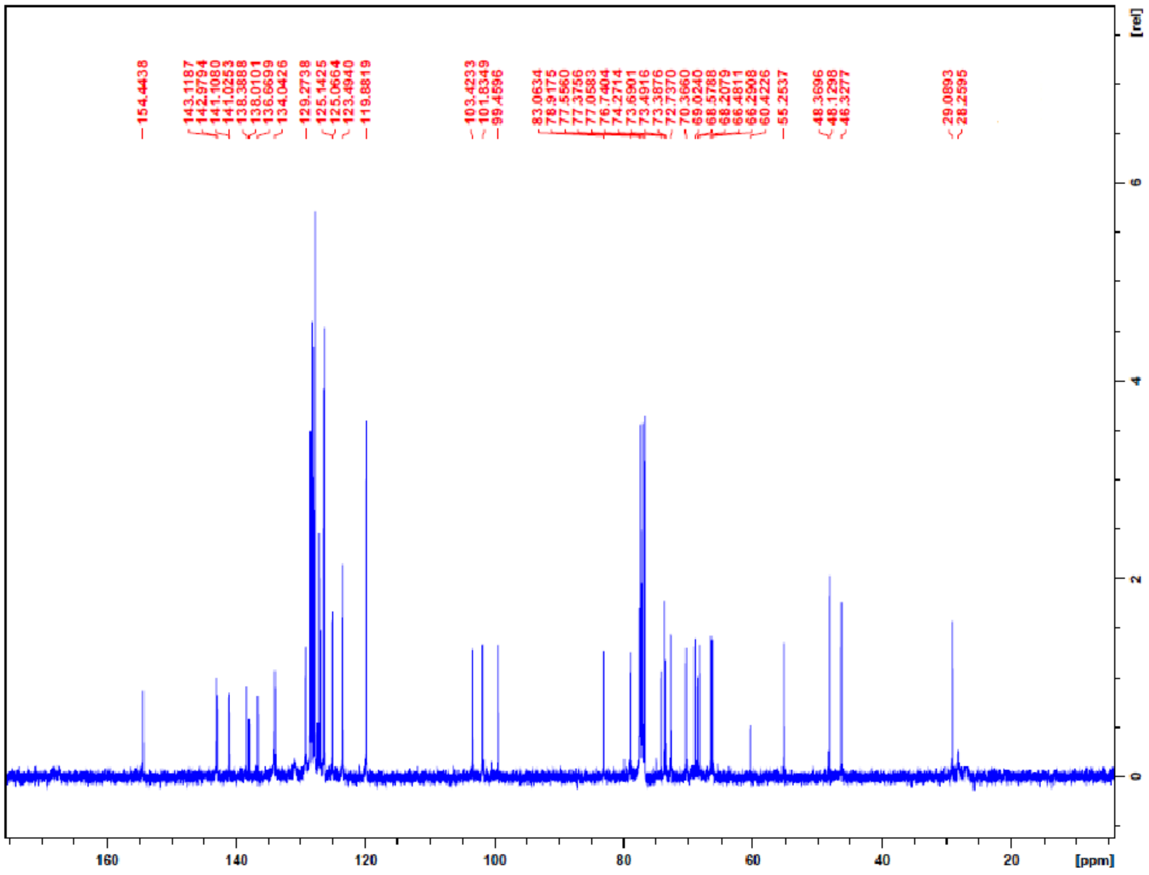

Compound 21b: ^{1}H NMR, $CDCl_3$, 400 MHz

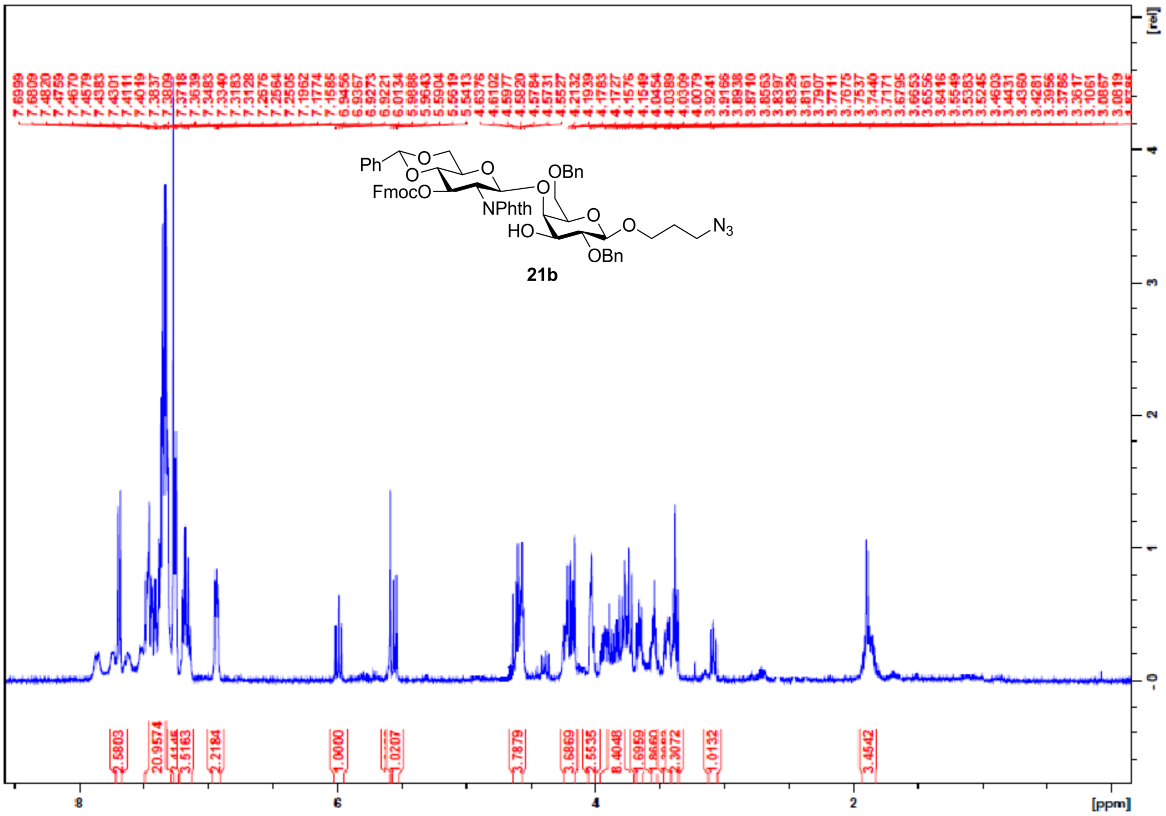

Compound 21b: ^{13}C NMR, $CDCl_3$, 101 MHz

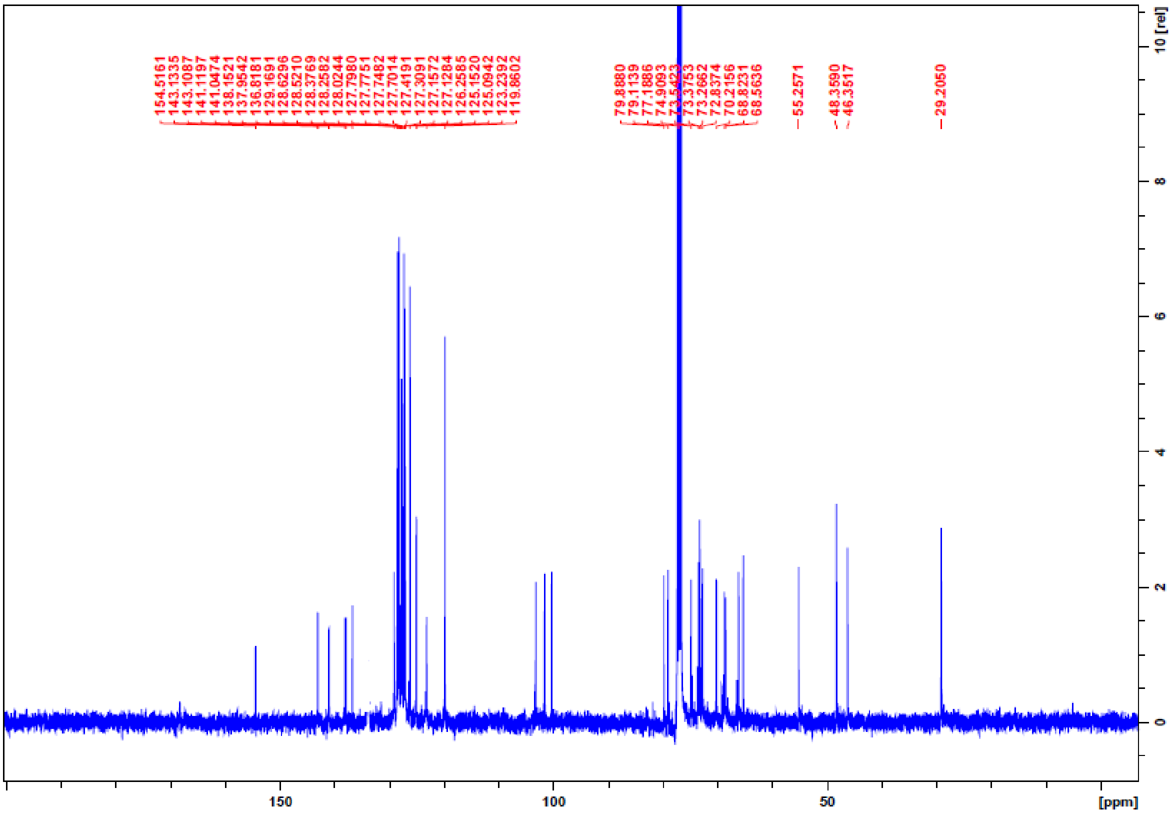

Compound 22a: ^{1}H NMR, $CDCl_3$, 400 MHz

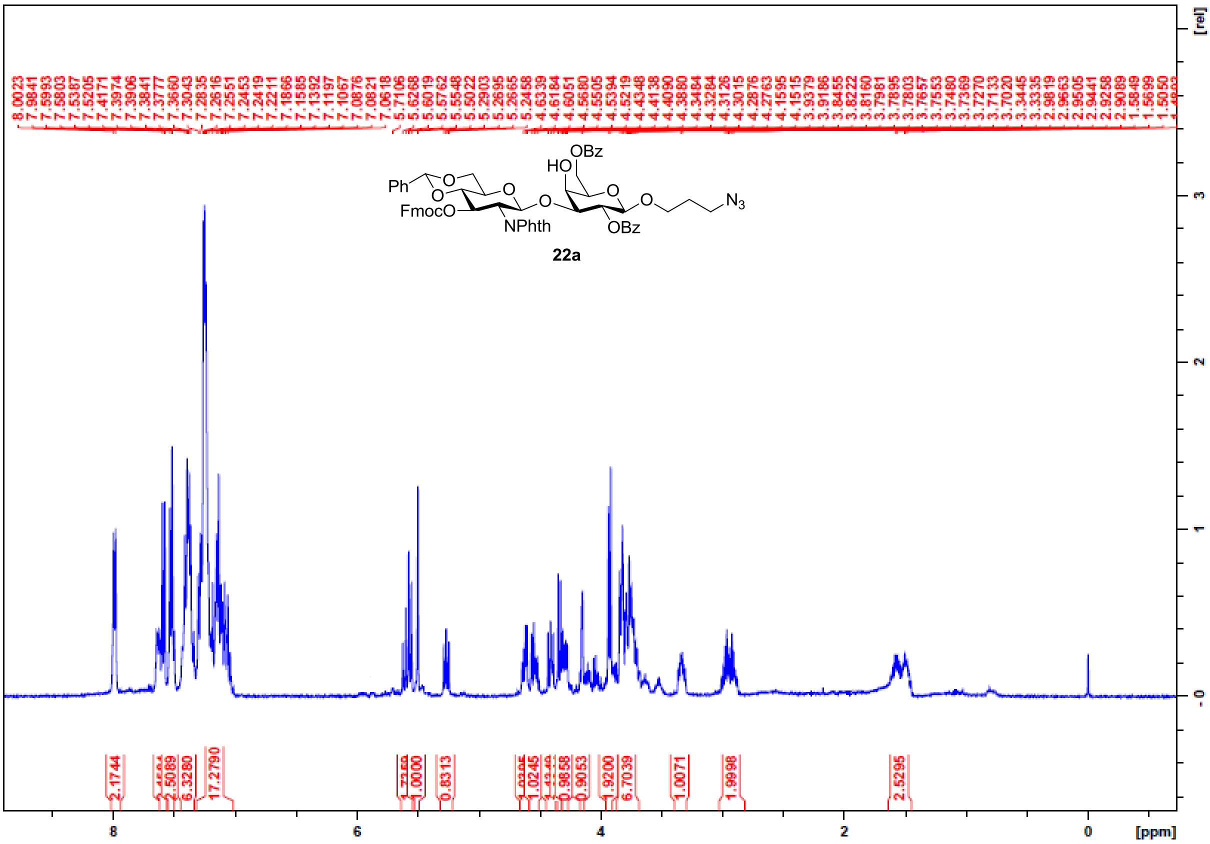

Compound 22a: ^{13}C NMR, $CDCl_3$, 101 MHz

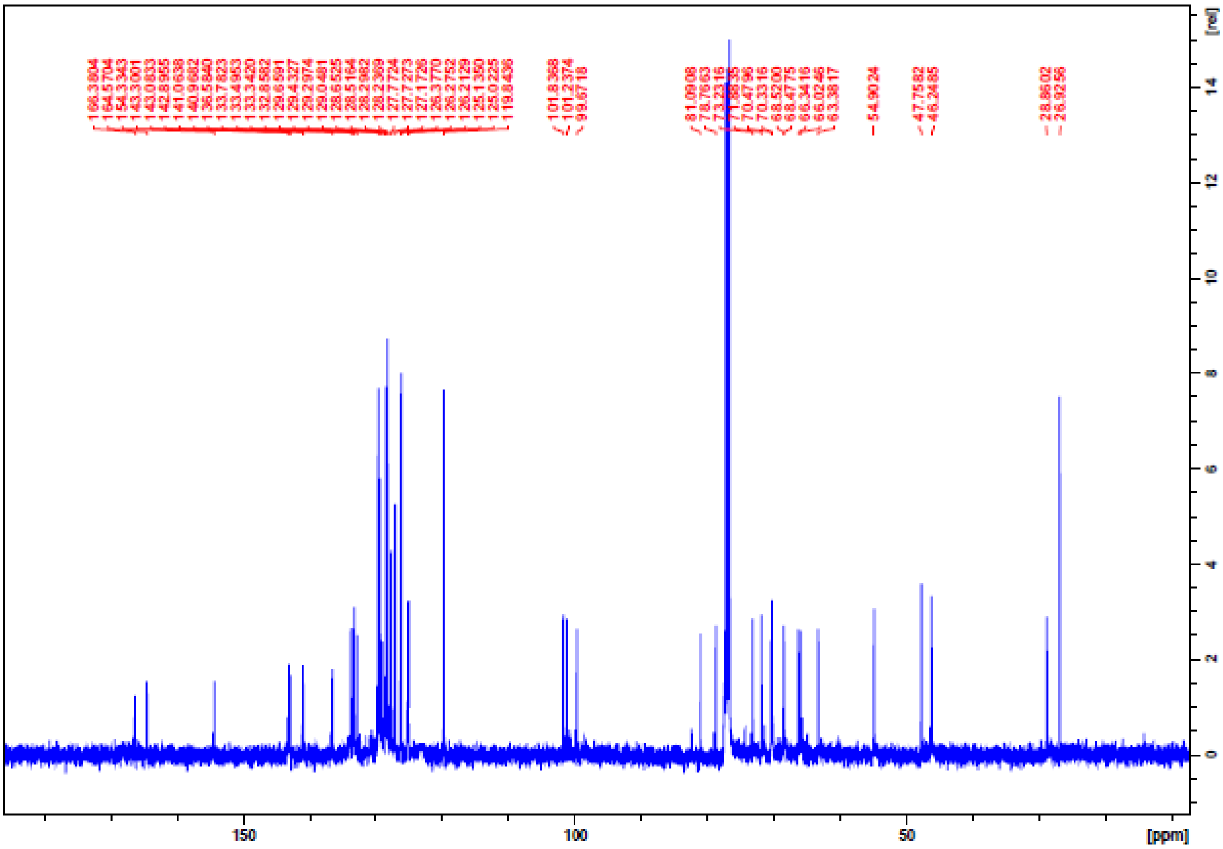

Compound 23a: ^{1}H NMR, $CDCl_3$, 400 MHz

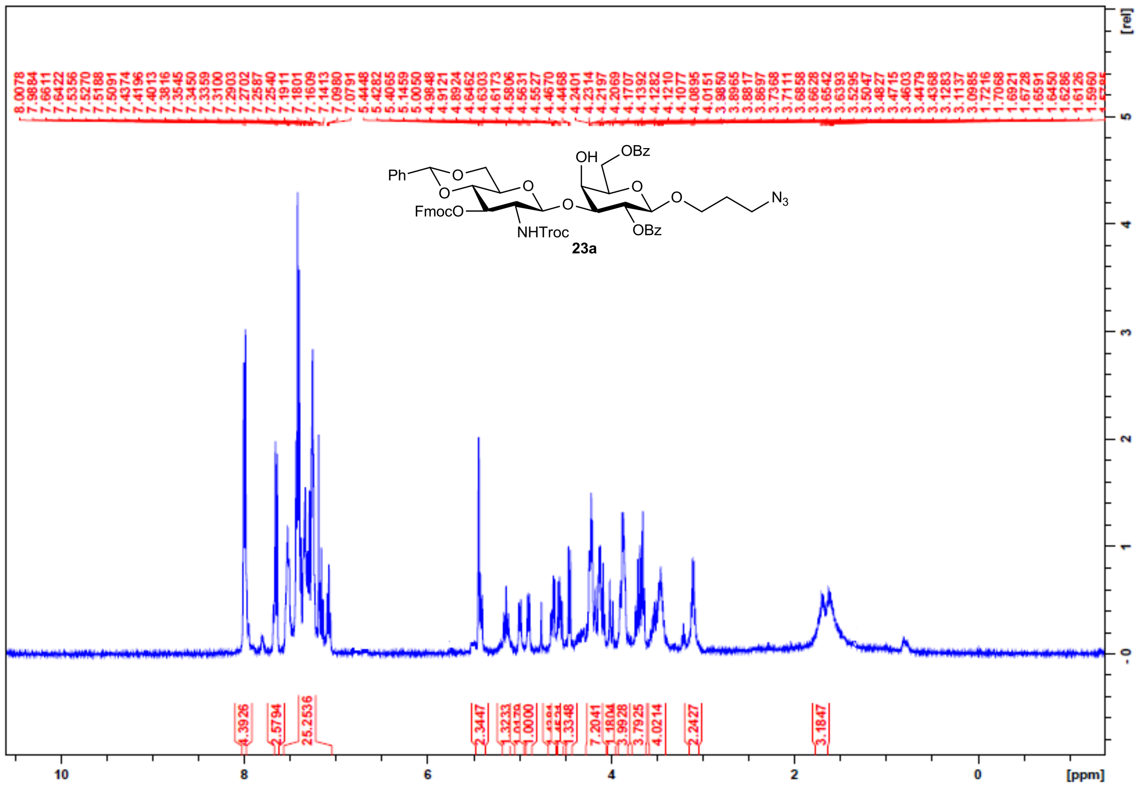

Compound 23a: ^{13}C NMR, $CDCl_3$, 101 MHz

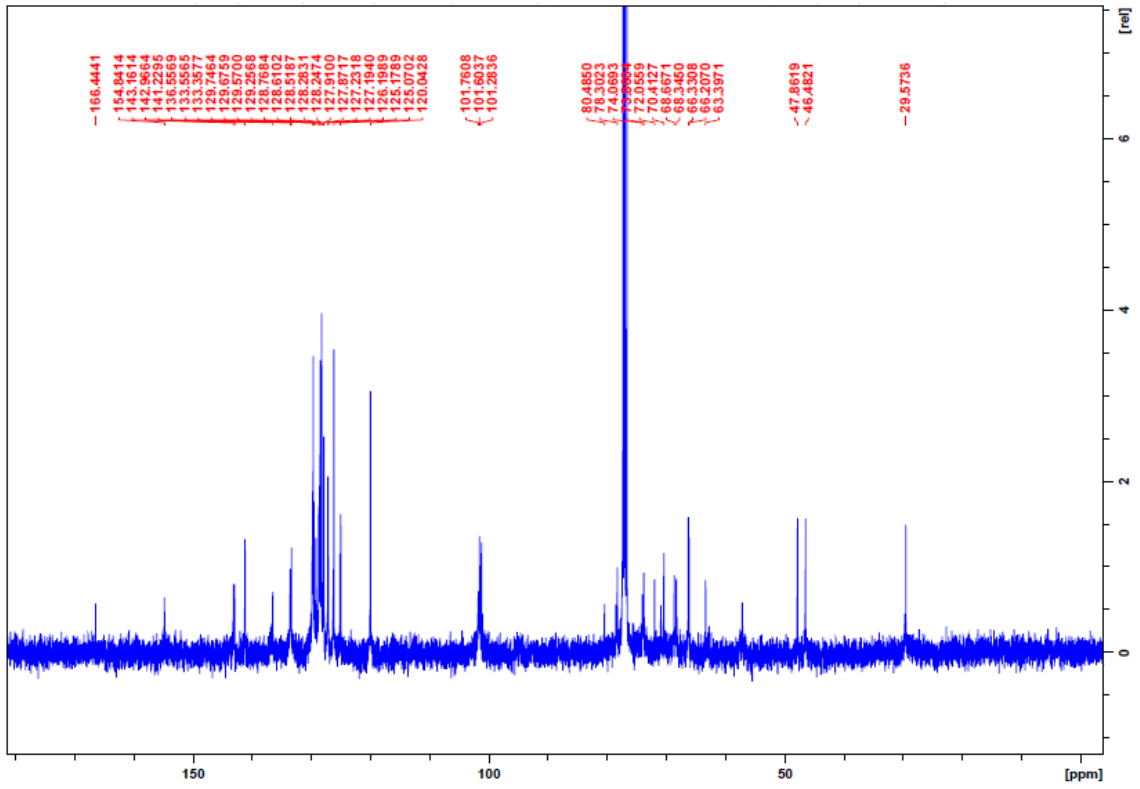

Compound 24a: ^{1}H NMR, $CDCl_3$, 400 MHz

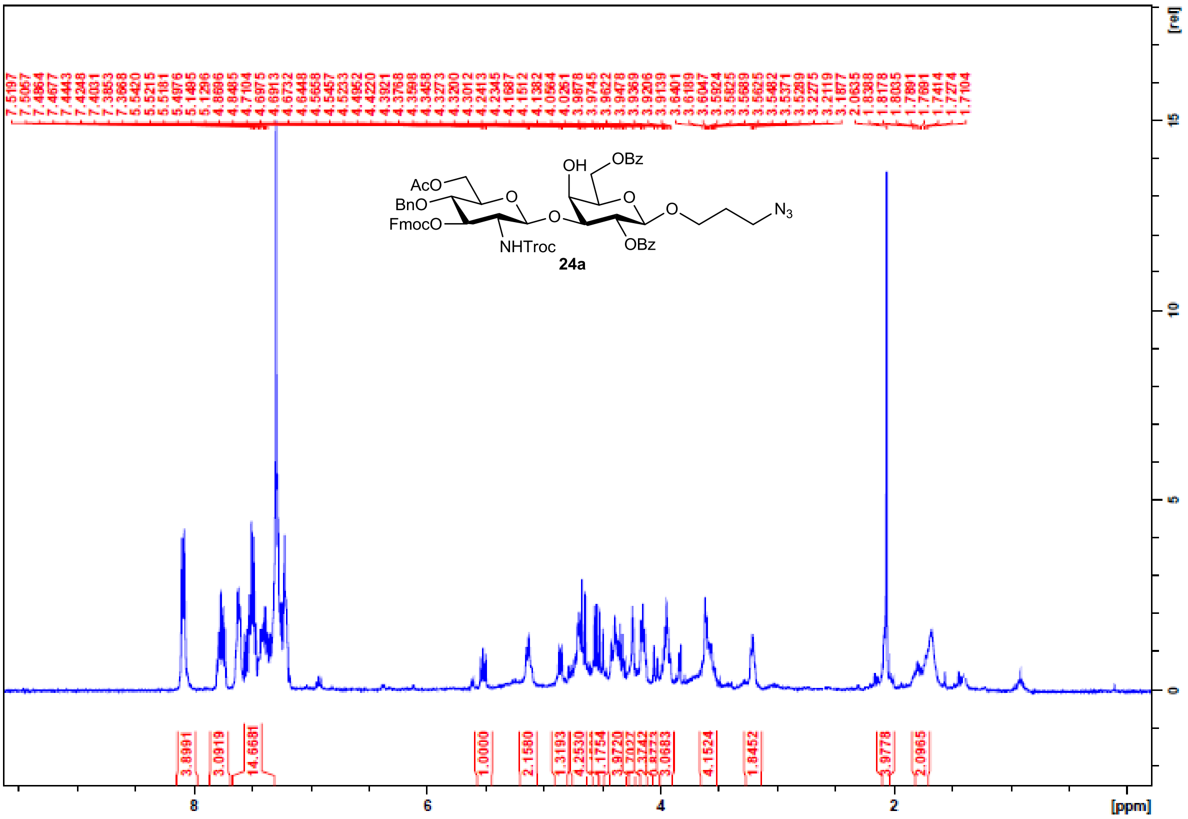

Compound 24a: HSQC, $CDCl_3$, 101 MHz

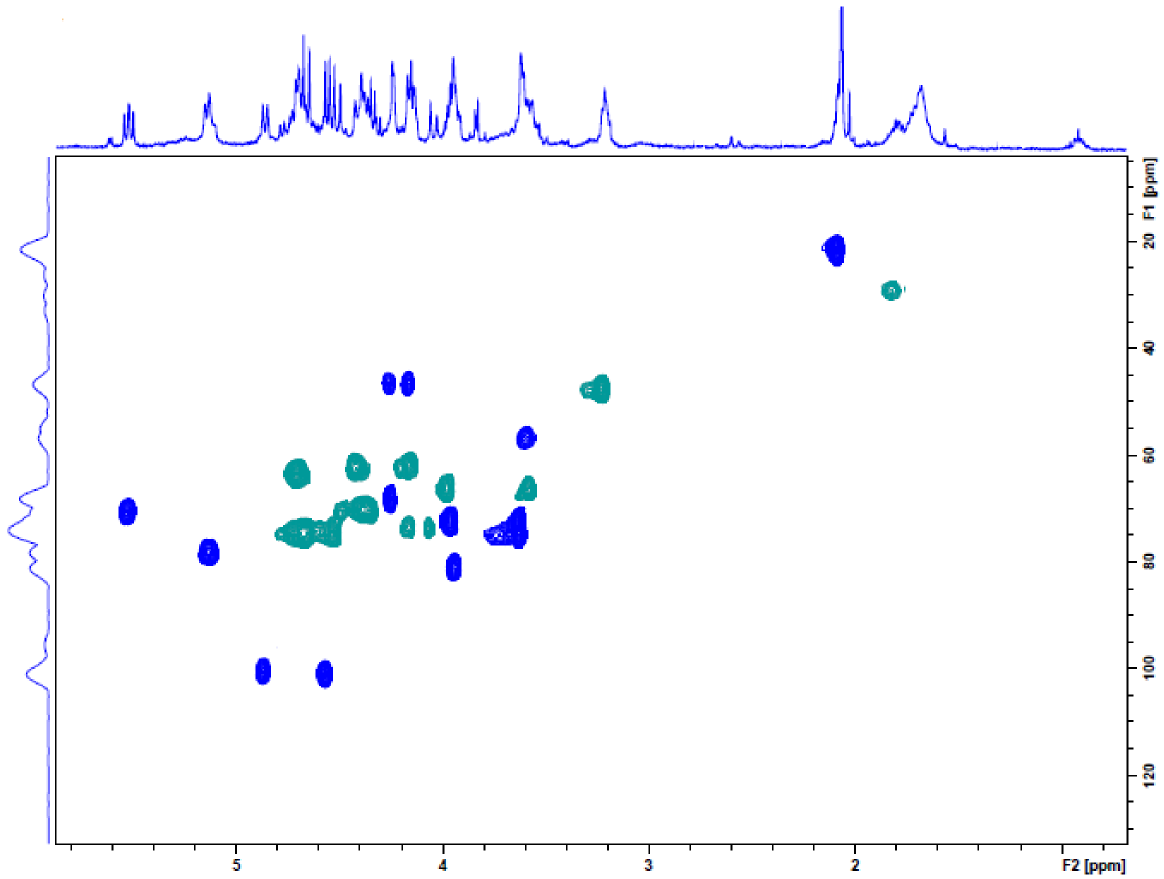

Compound 25a: ^{1}H NMR, $CDCl_3$, 400 MHz

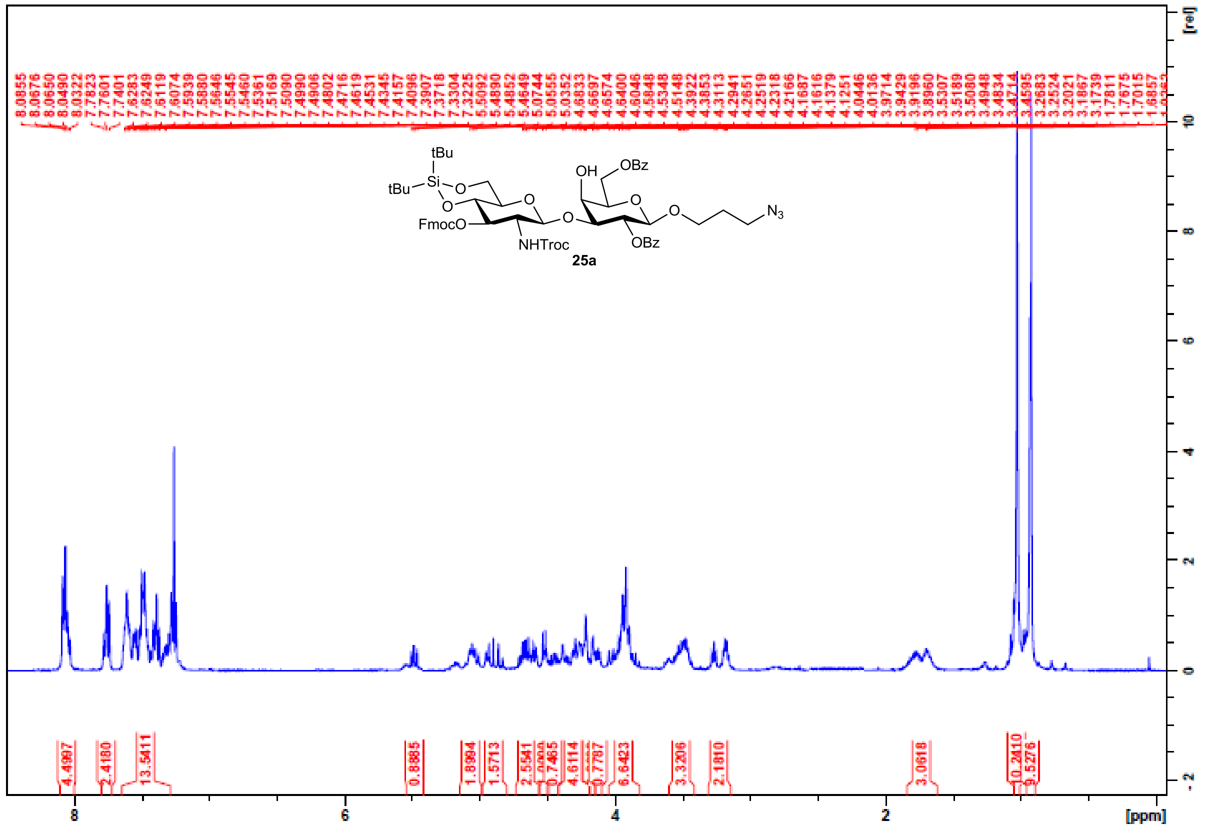

Compound 25a: ^{13}C NMR, $CDCl_3$, 101 MHz

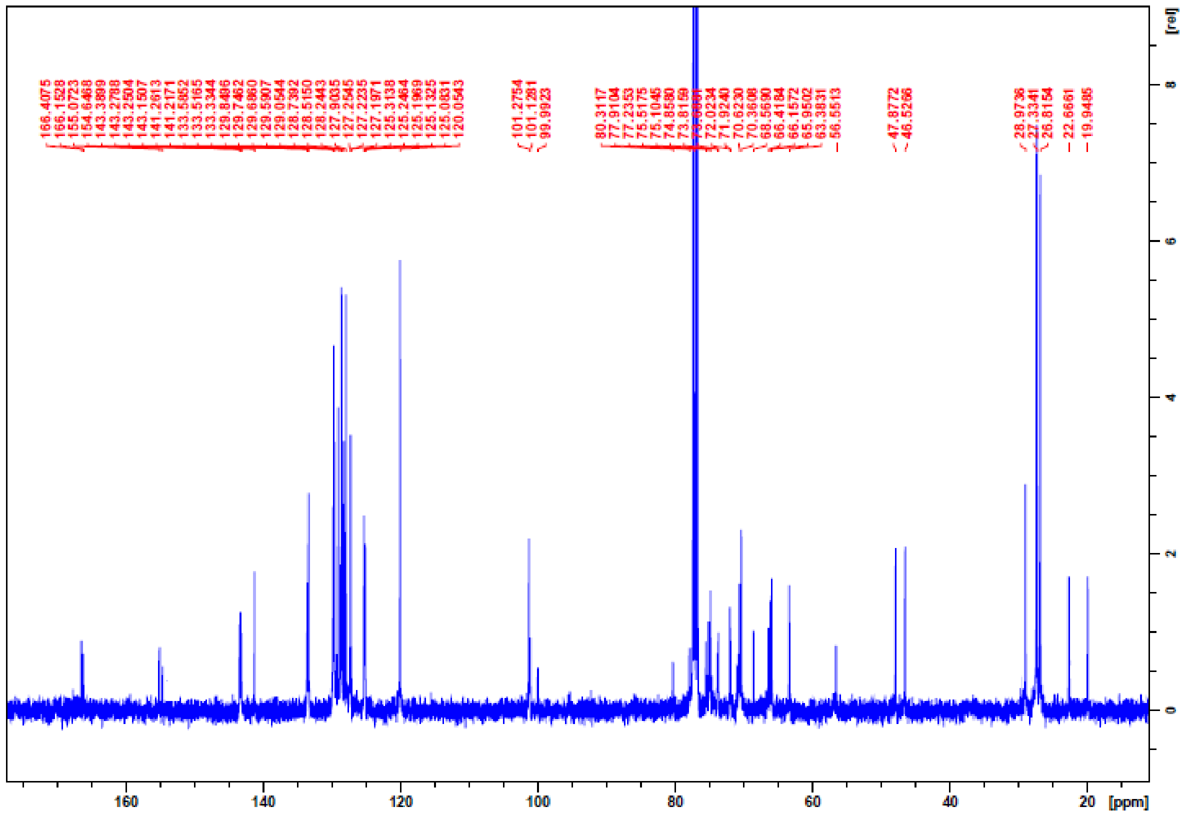

Compound 27a: ^{1}H NMR, $CDCl_3$, 400 MHz

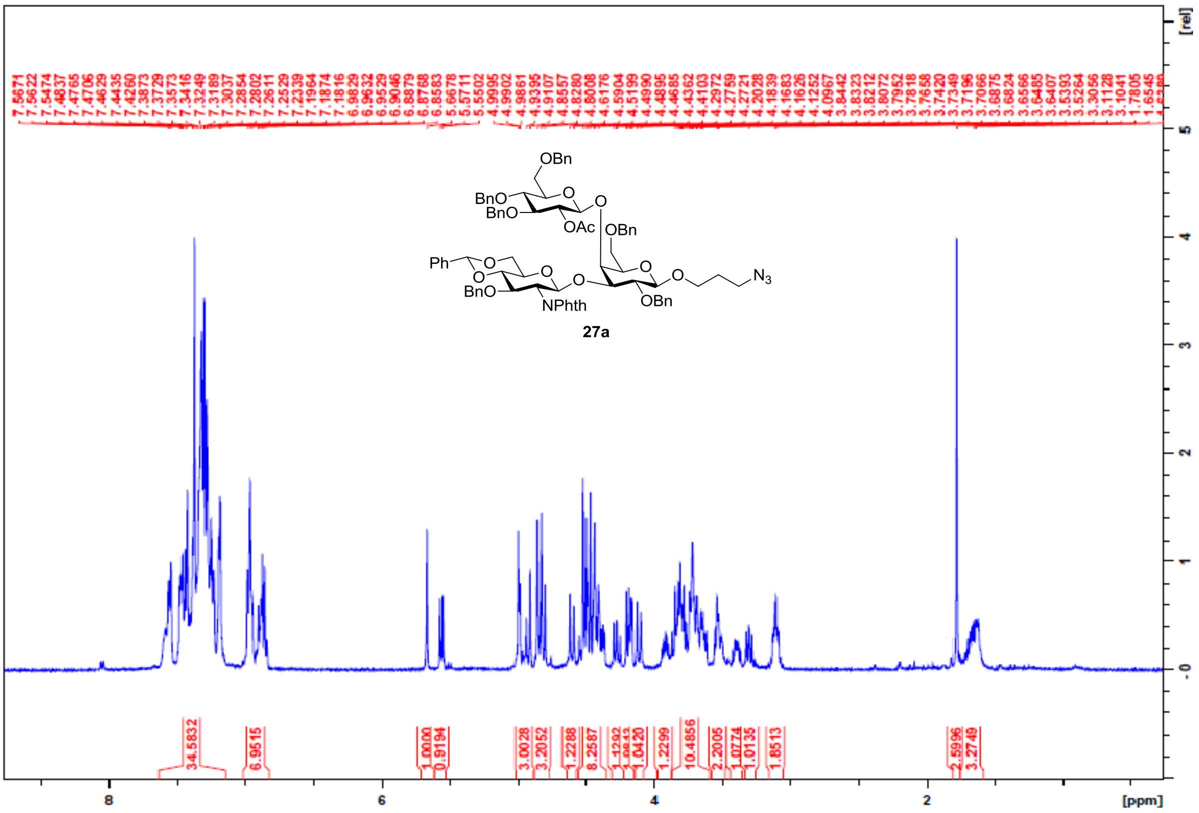

Compound 27a: ^{13}C NMR, $CDCl_3$, 101 MHz

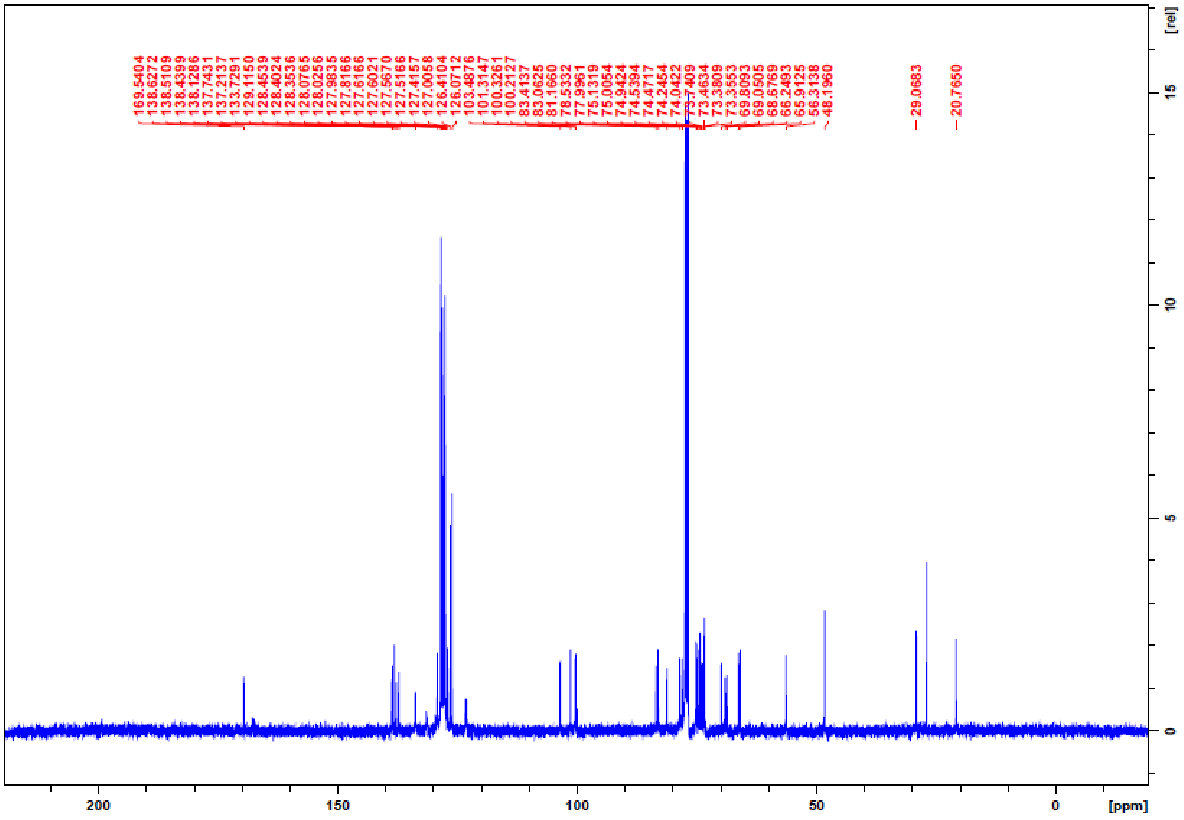

Compound 27b: ^1H NMR, $CDCl_3$, 400 MHz

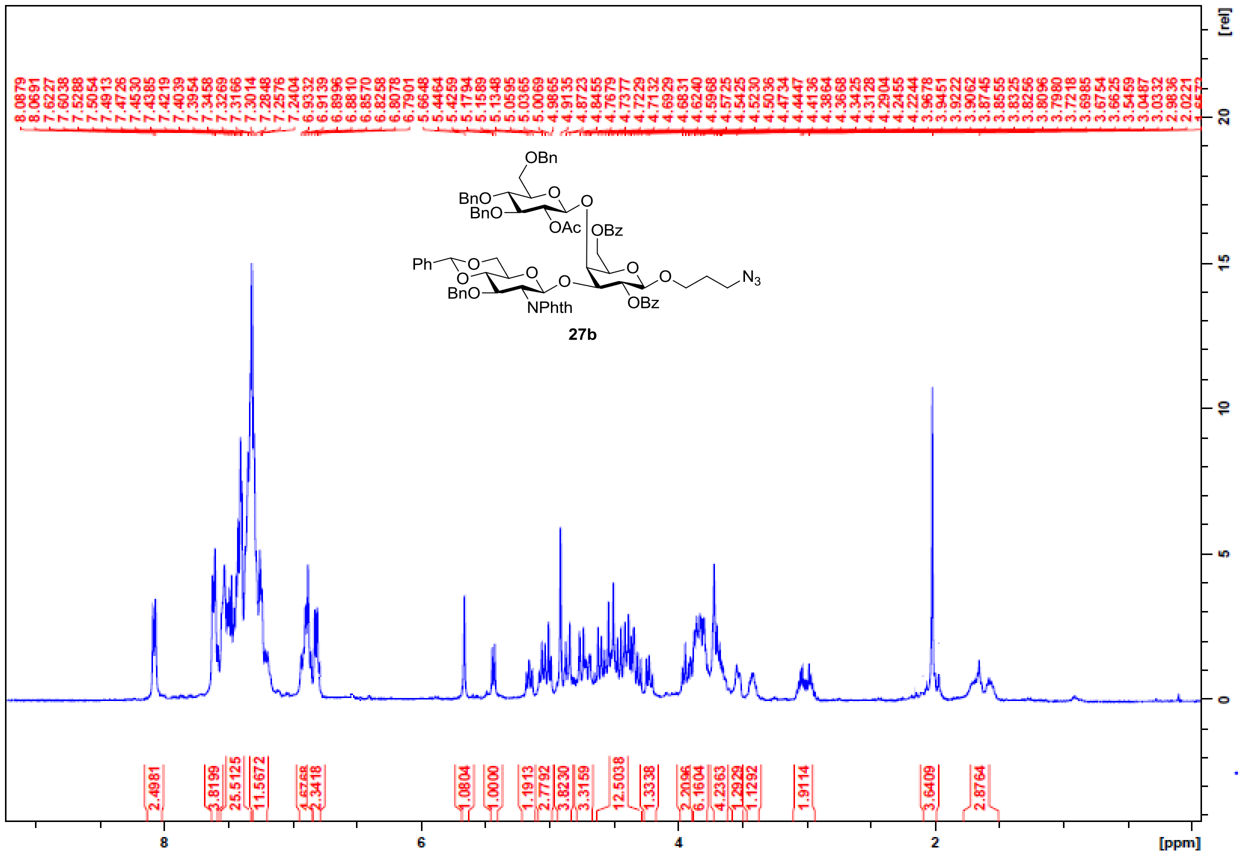

Compound 27b: ^{13}C NMR, $CDCl_3$, 101 MHz

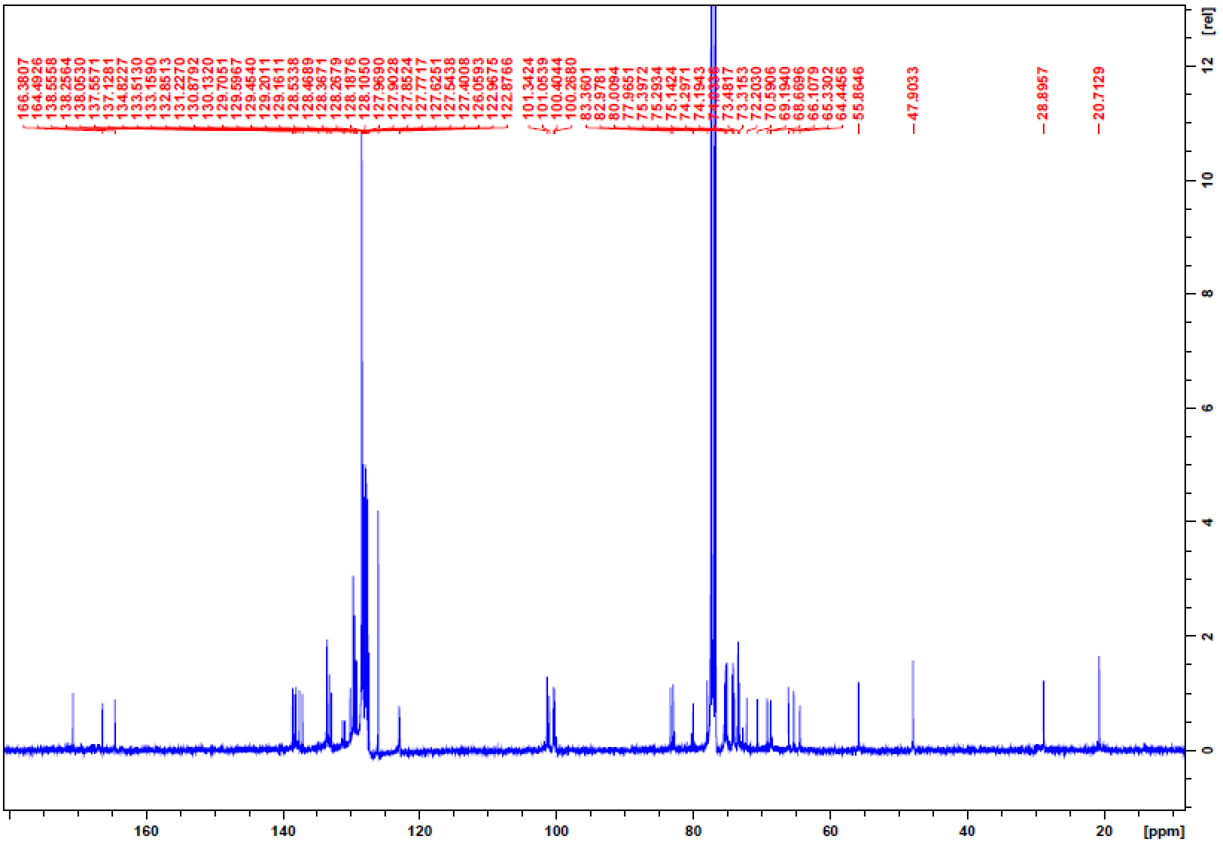

Compound 28: ^{1}H NMR, $CDCl_3$, 400 MHz

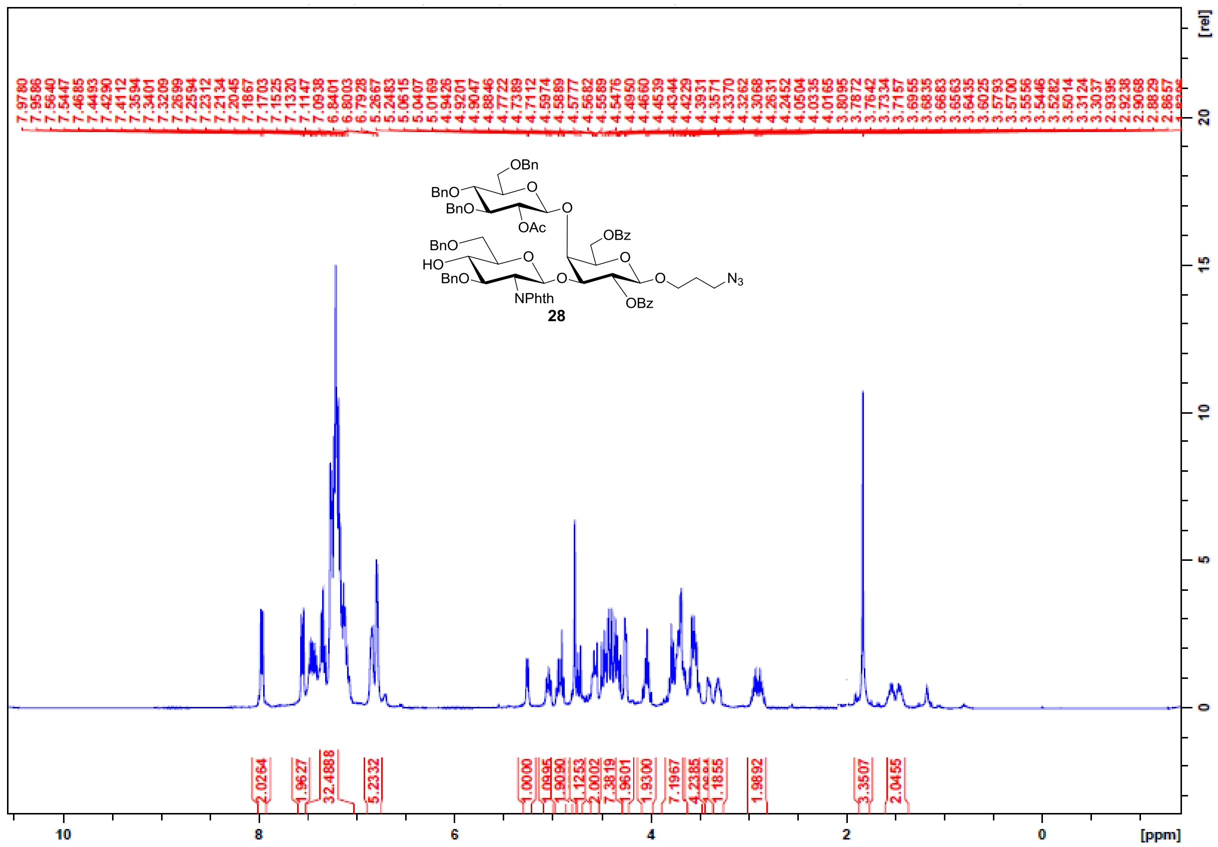

Compound 28: ^{13}C NMR, $CDCl_3$, 101 MHz

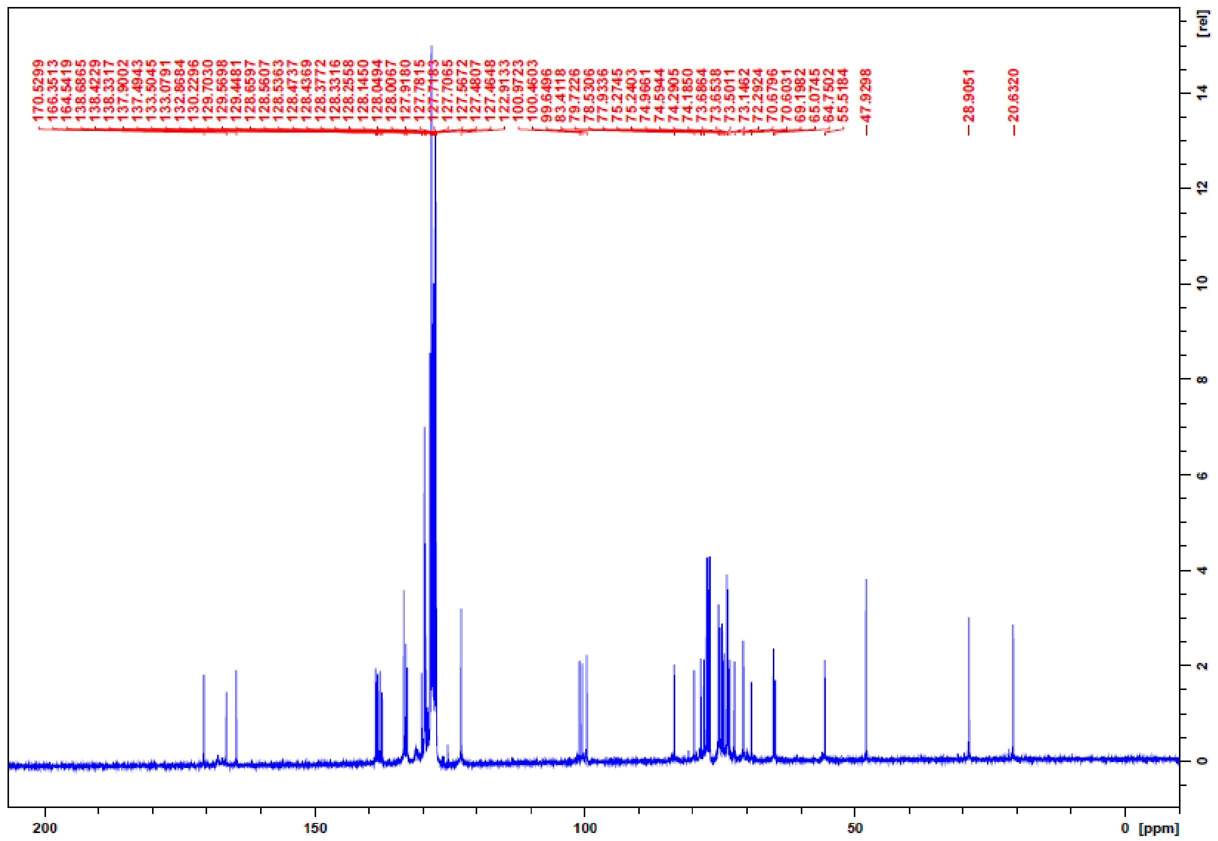

Compound 34: ^{1}H NMR, $CDCl_3$, 400 MHz

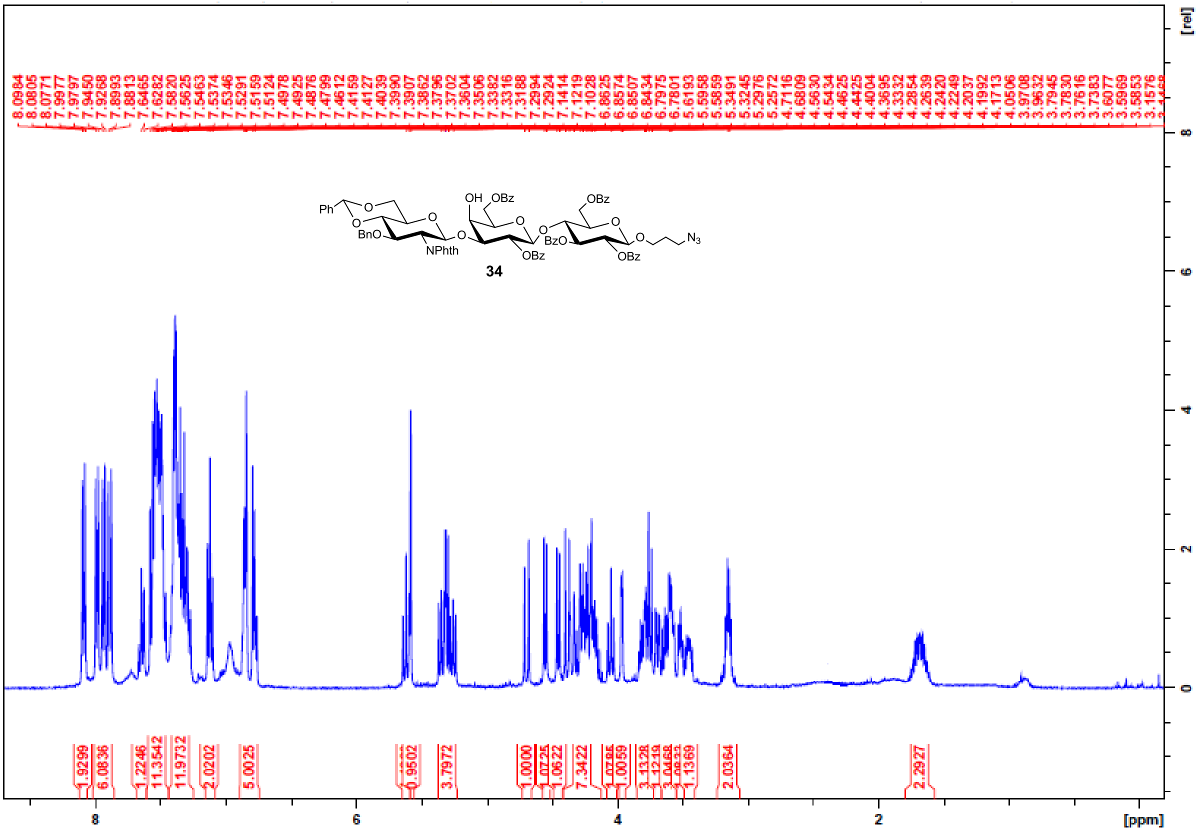

Compound 34: ^{13}C NMR, $CDCl_3$, 101 MHz

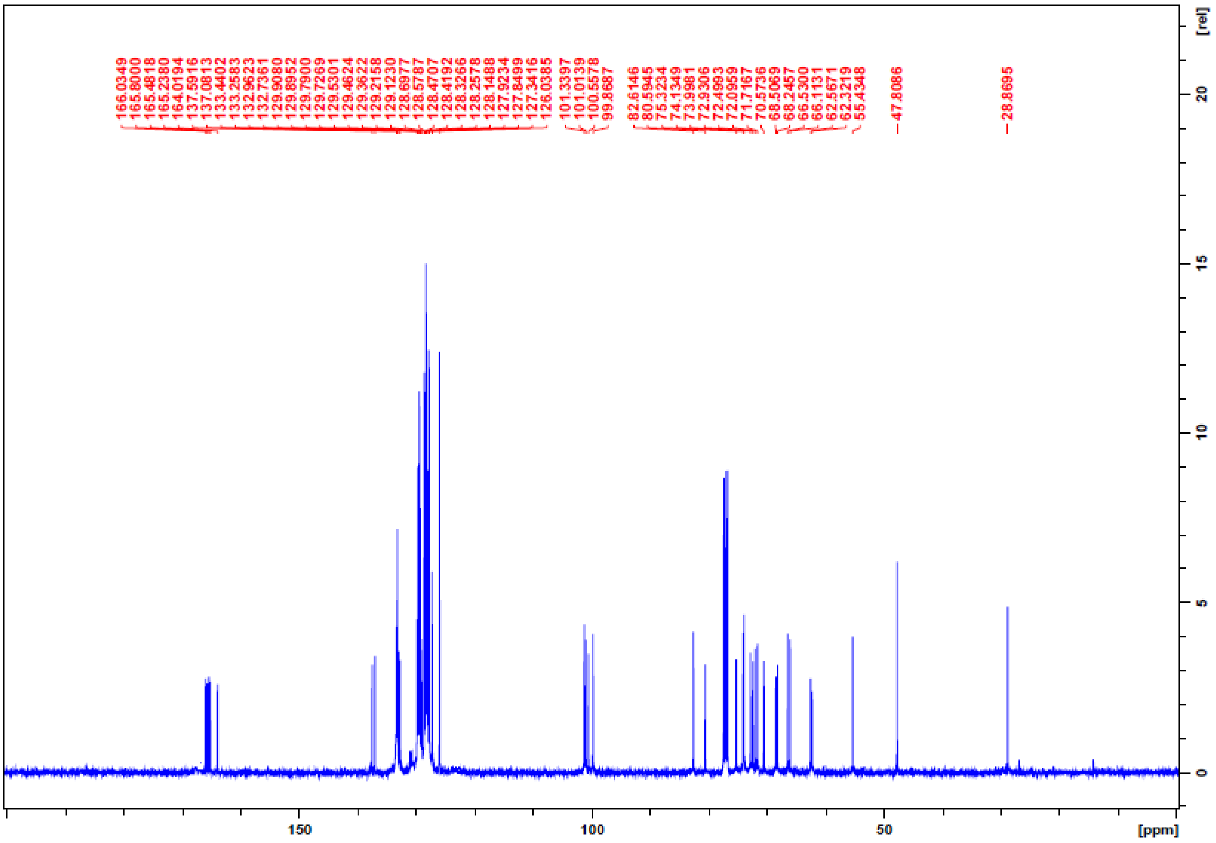

Compound 35: ^{1}H NMR, $CDCl_3$, 400 MHz

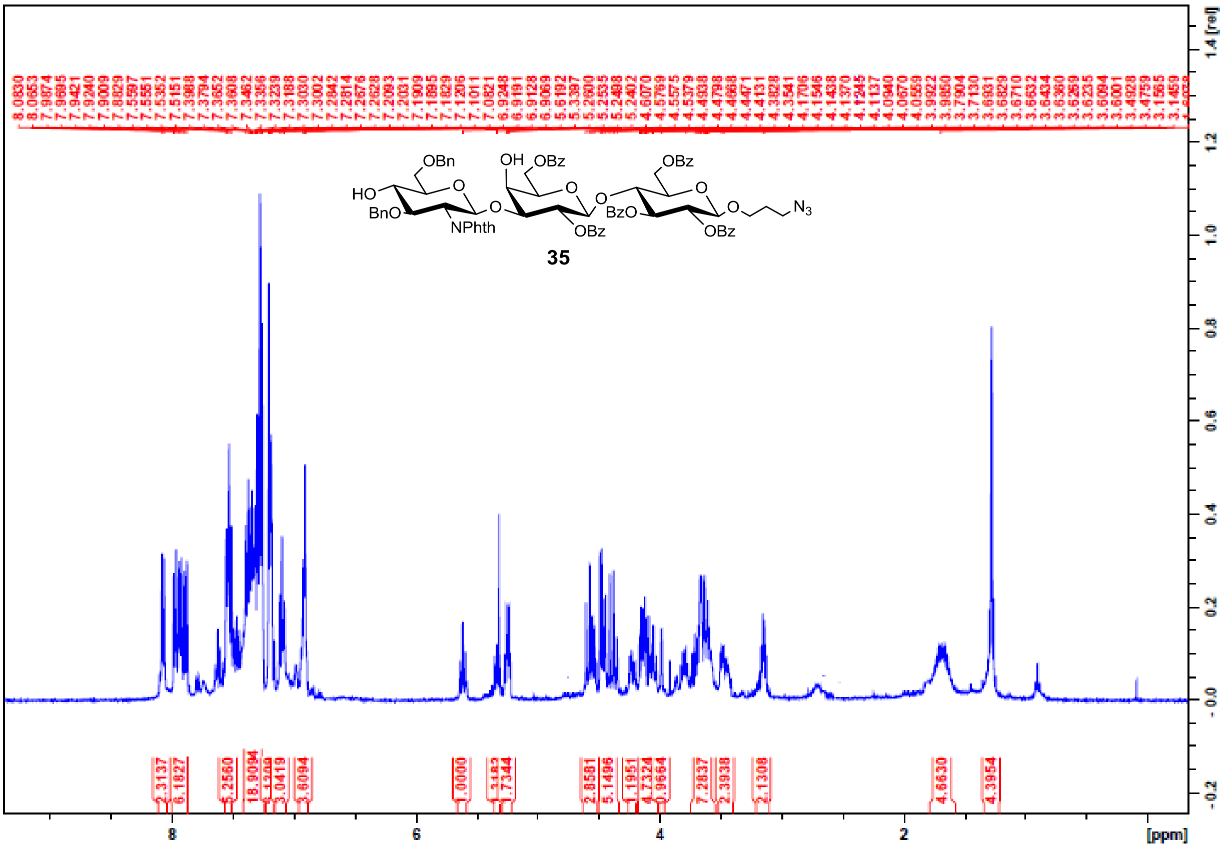

Compound 35: ^{13}C NMR, $CDCl_3$, 101 MHz

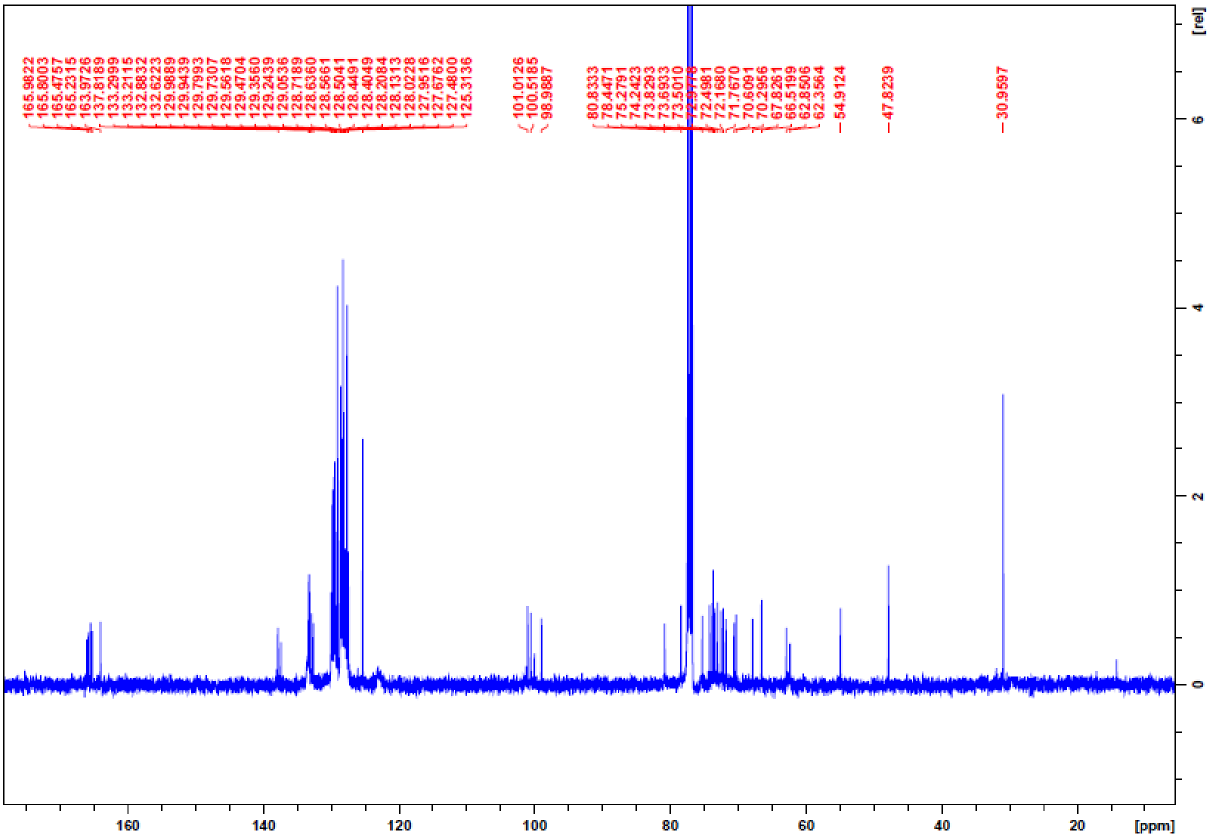

Compound 76: ^{1}H NMR, $CDCl_3$, 400 MHz

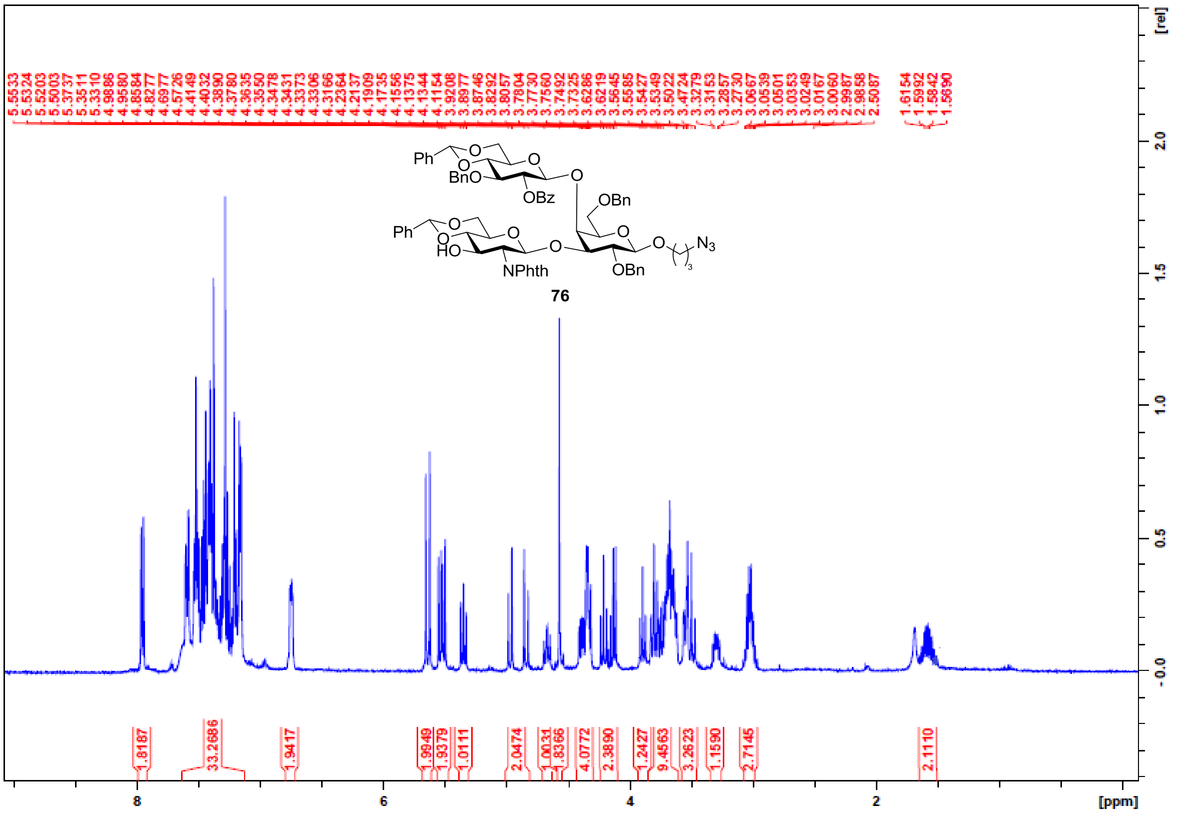

Compound 76: ^{13}C NMR, $CDCl_3$, 101 MHz

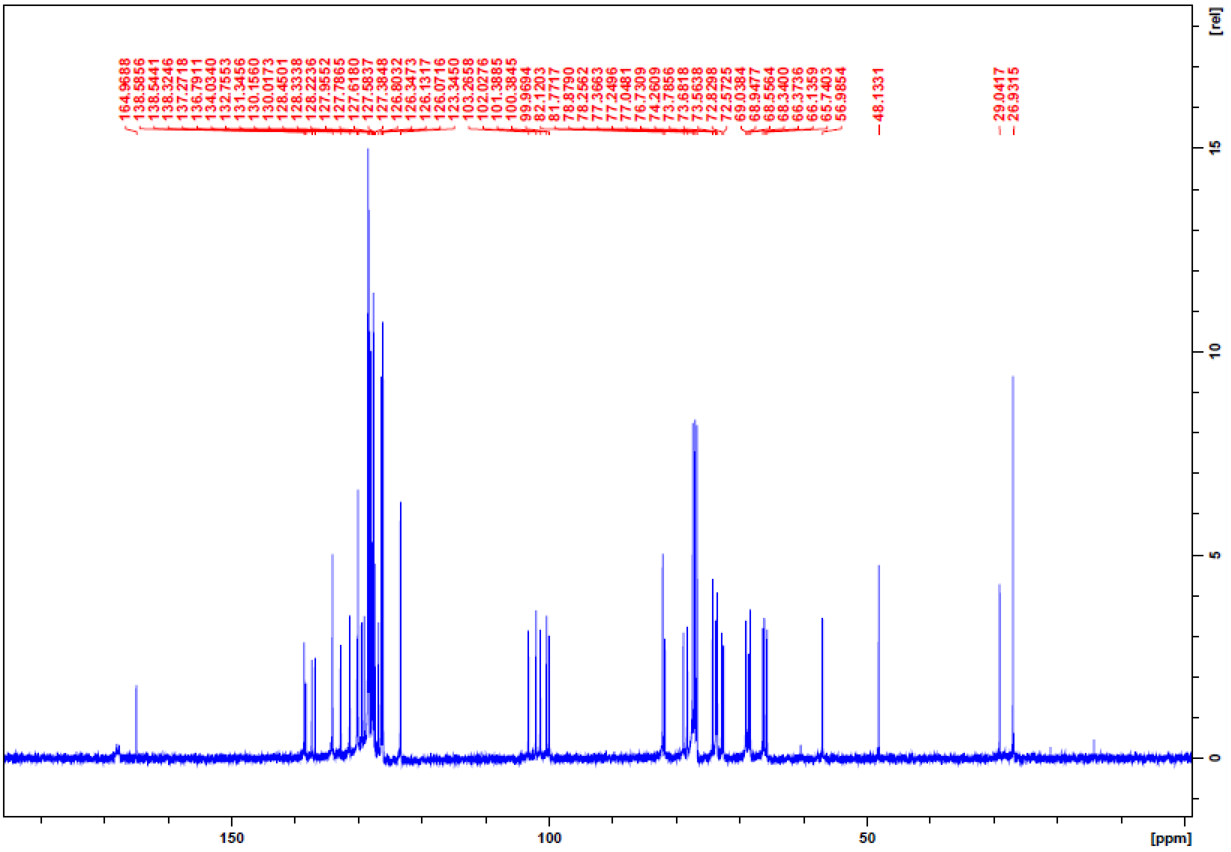

Compound 40: ^{1}H NMR, $CDCl_3$, 400 MHz

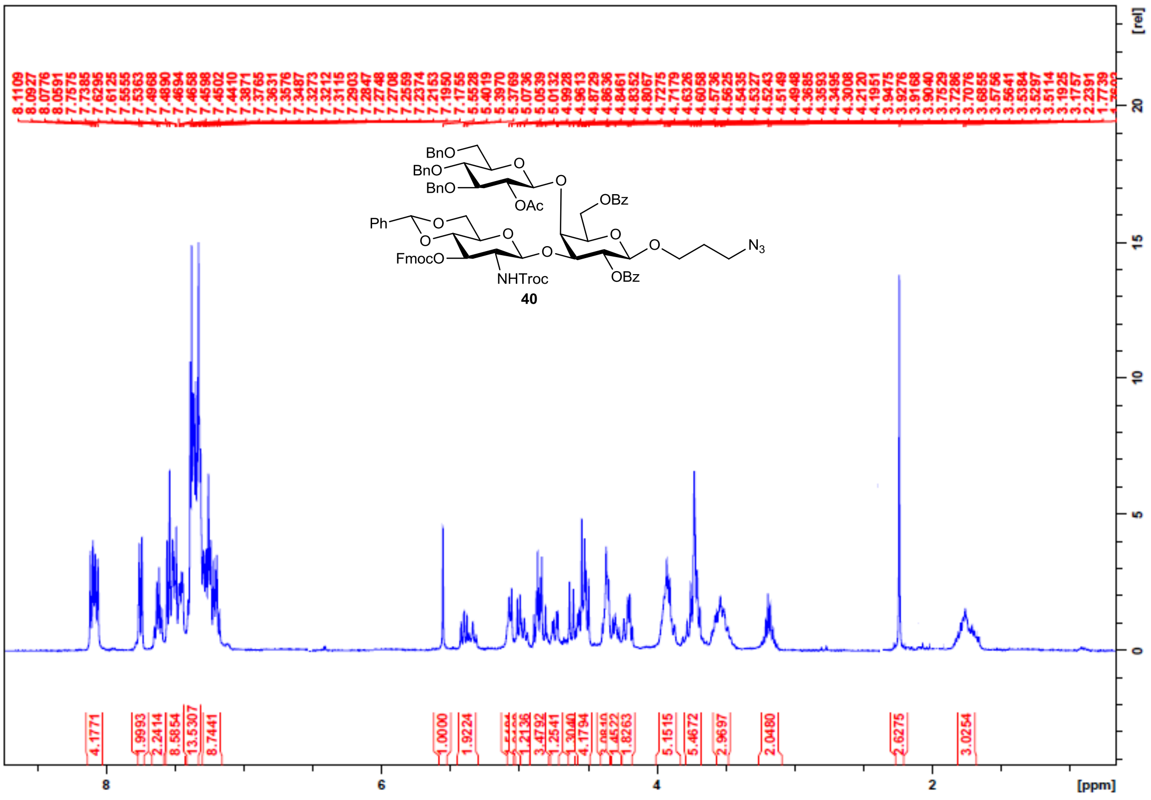

Compound 40: ^{13}C NMR, $CDCl_3$, 101 MHz

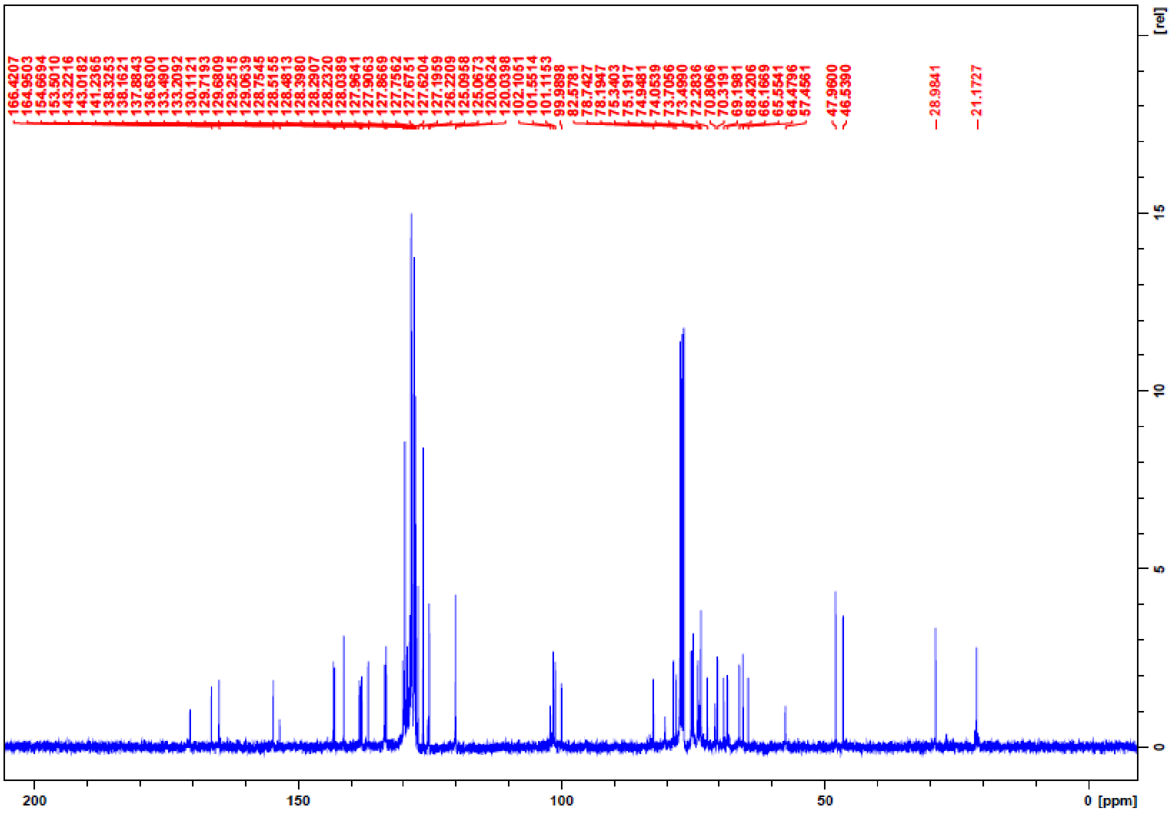

Compound 41: ¹H NMR, CDCl₃, 400 MHz

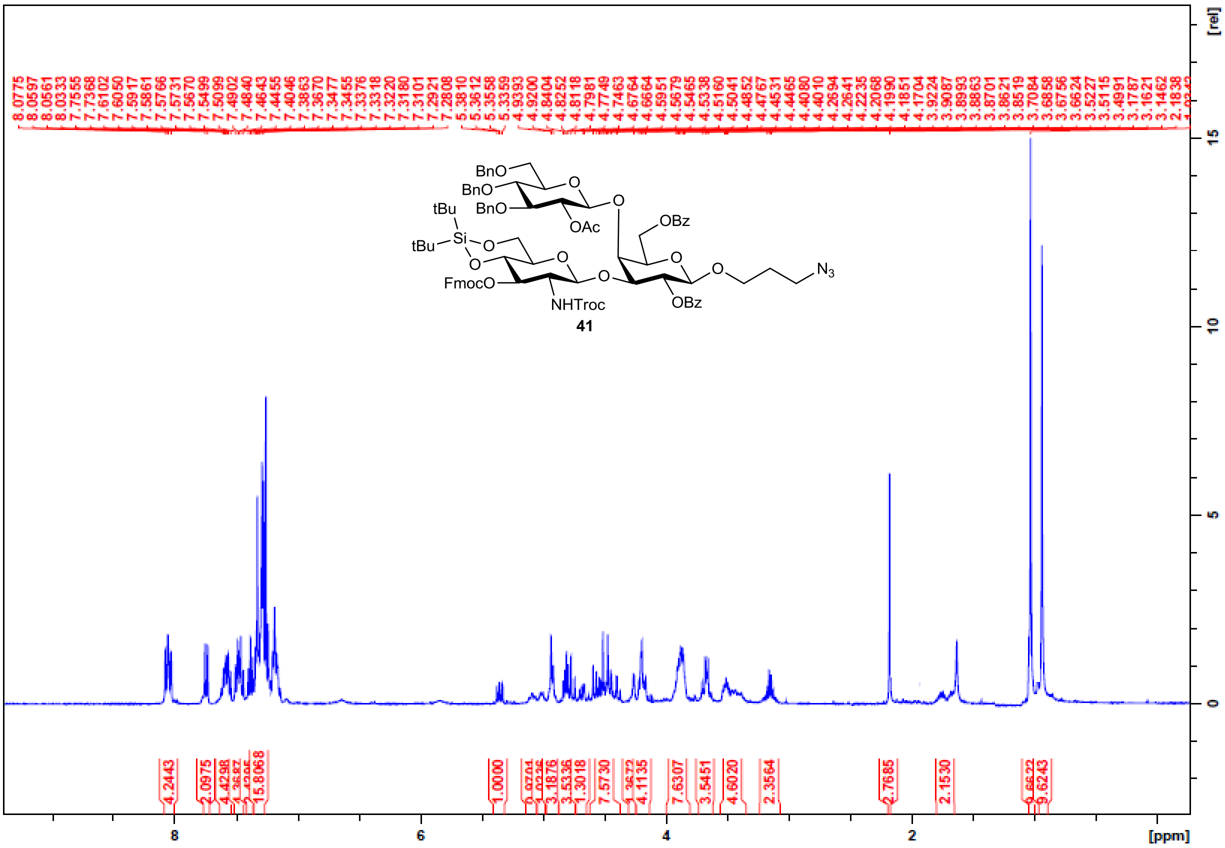

Compound 41: ¹³C NMR, CDCl₃, 101 MHz

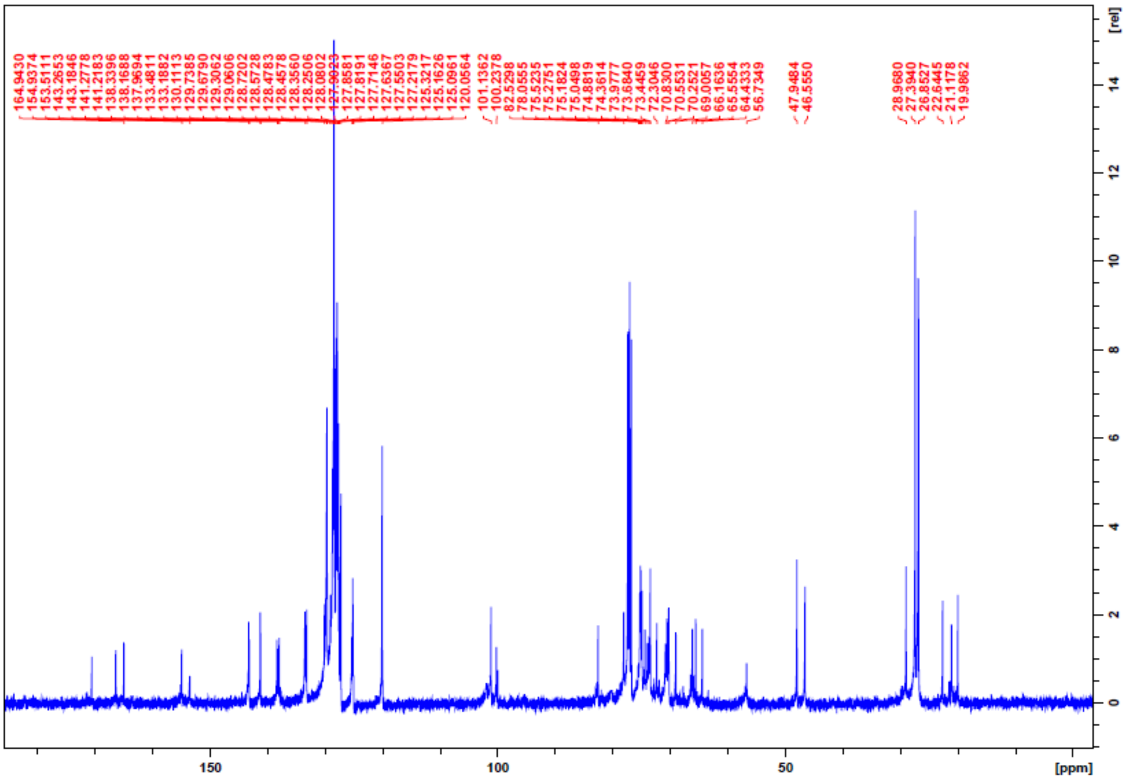

Compound 42: ^{1}H NMR, $CDCl_3$, 400 MHz

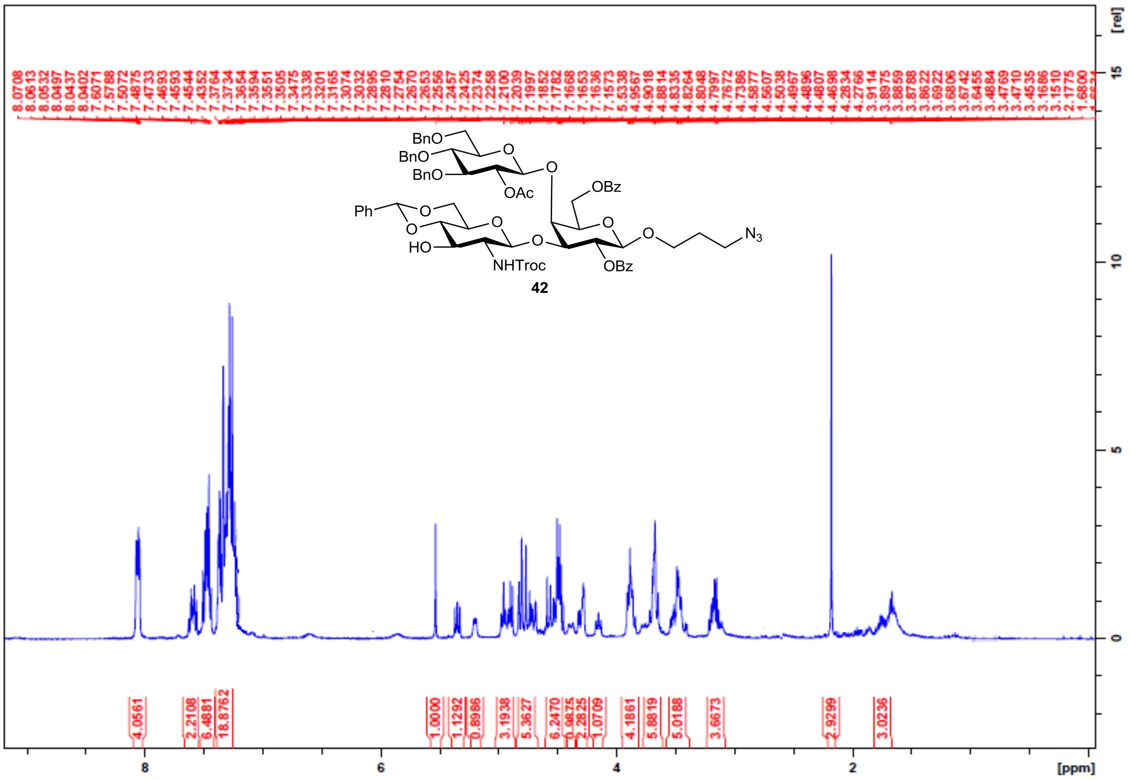

Compound 42: ^{13}C NMR, $CDCl_3$, 101 MHz

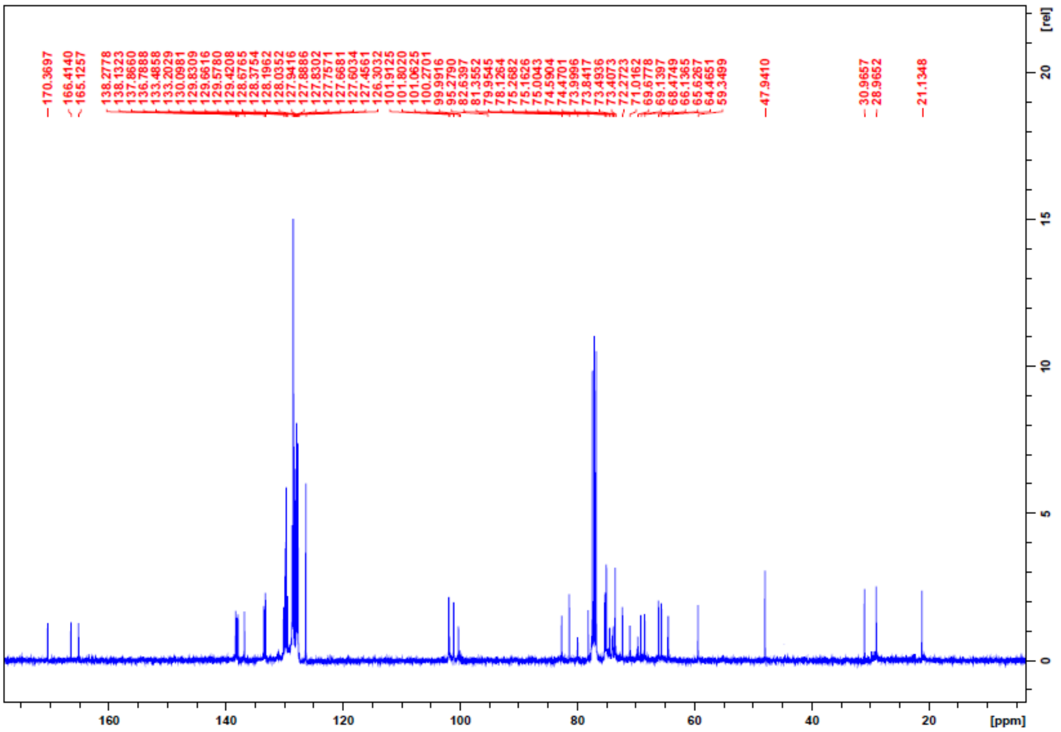

Compound 43: ^{1}H NMR, $CDCl_3$, 400 MHz

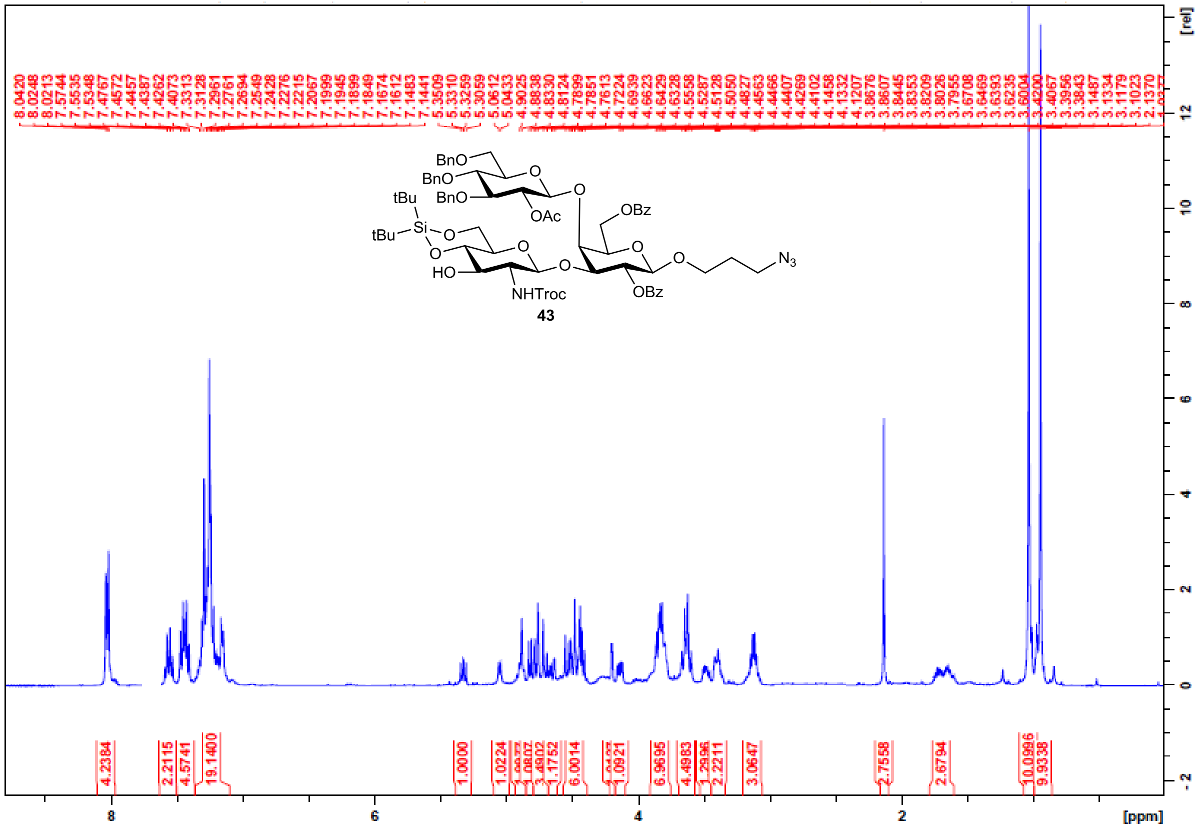

Compound 43: ^{13}C NMR, $CDCl_3$, 101 MHz

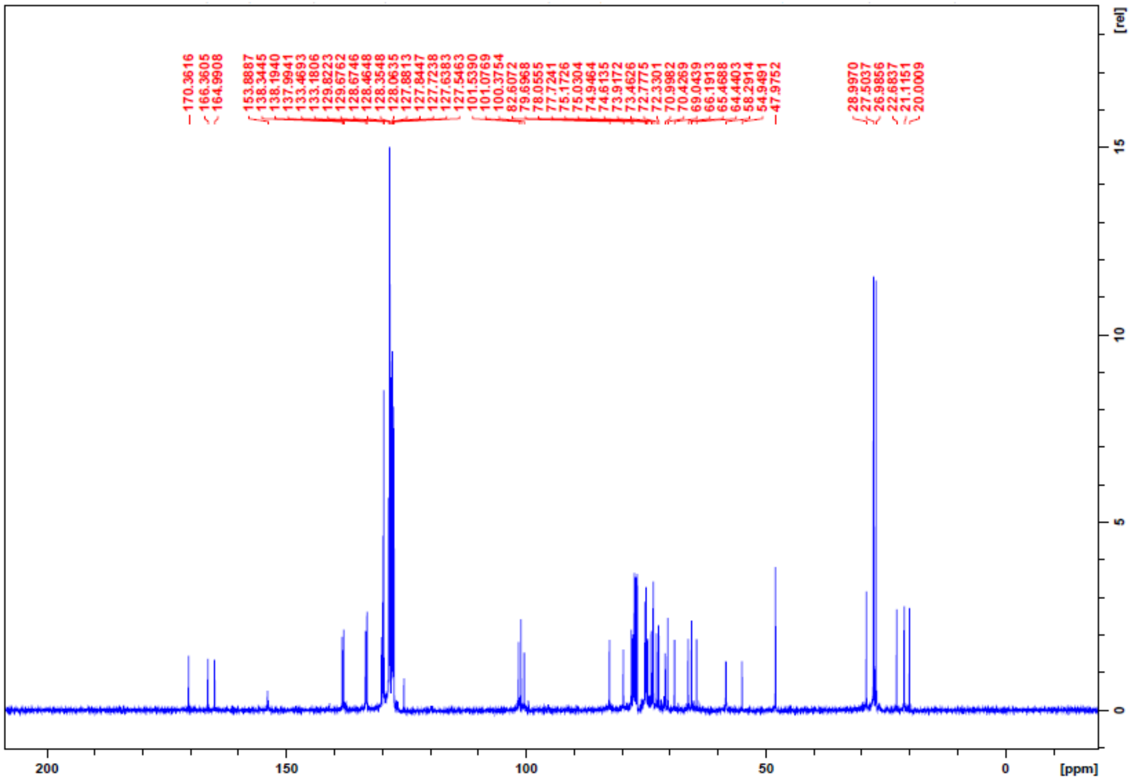

Compound 46: ^{1}H NMR, $CDCl_3$, 400 MHz

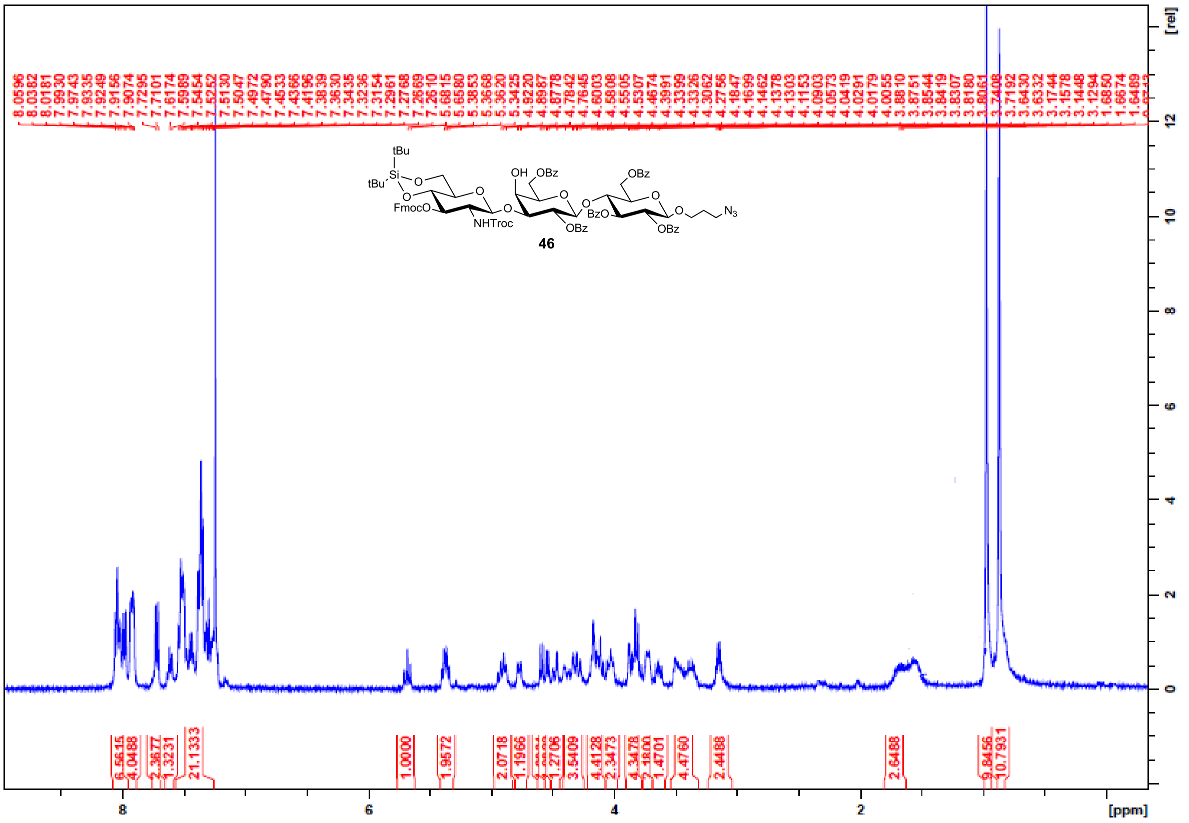

Compound 46: ^{13}C NMR, $CDCl_3$, 101 MHz

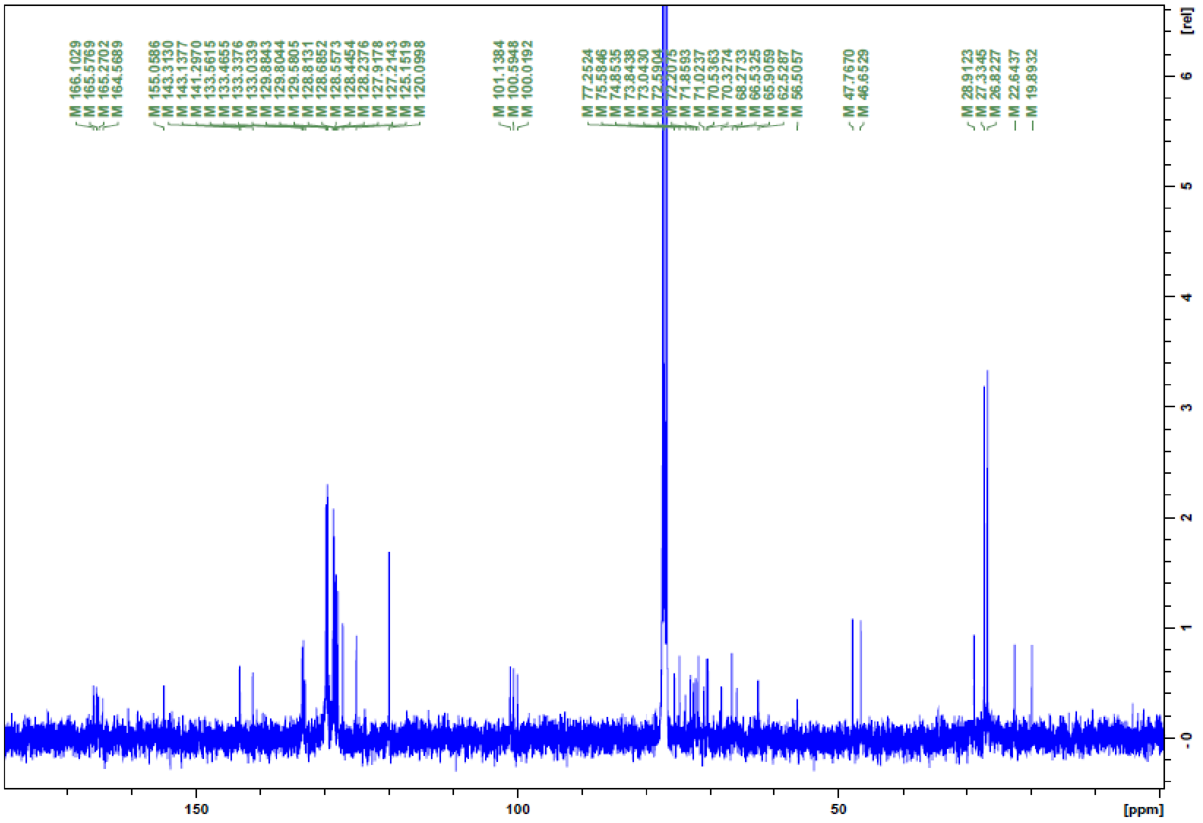

Compound 47: ¹H NMR, $CDCl_3$, 400 MHz

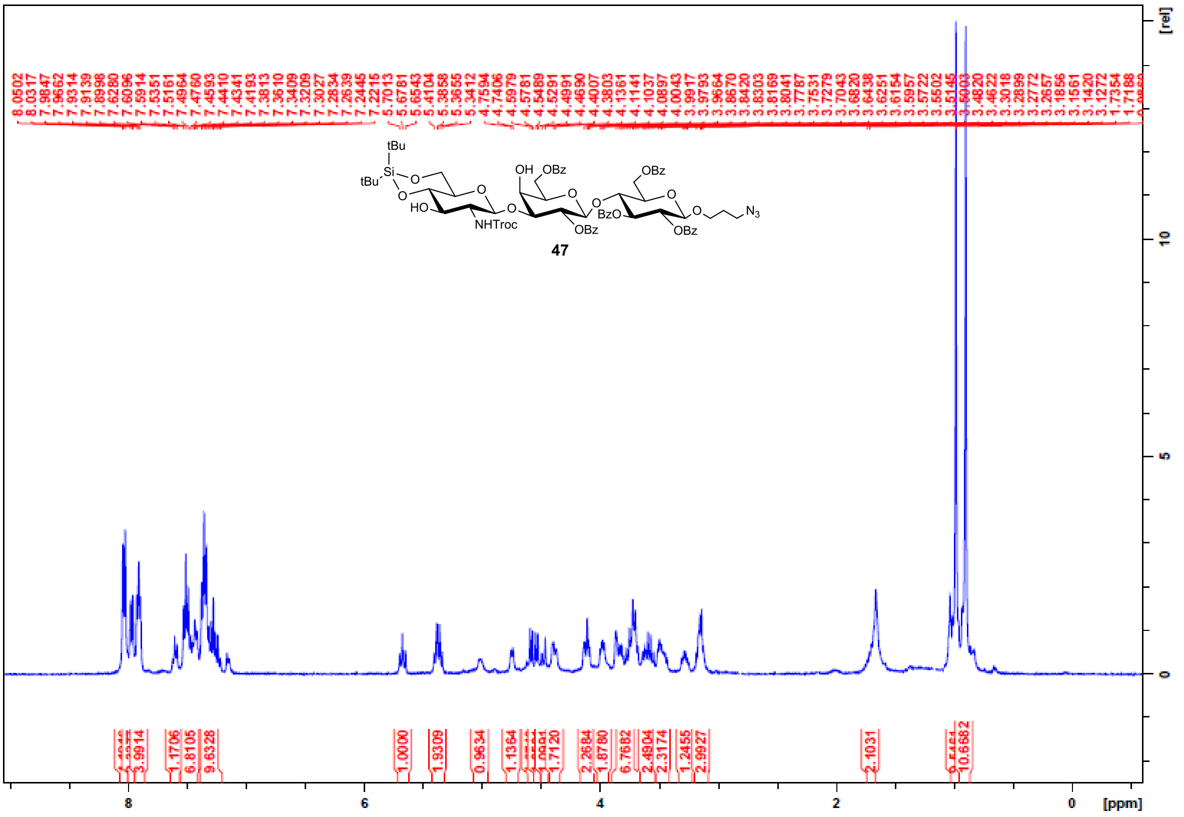

Compound 47: ¹³C NMR, $CDCl_3$, 101 MHz

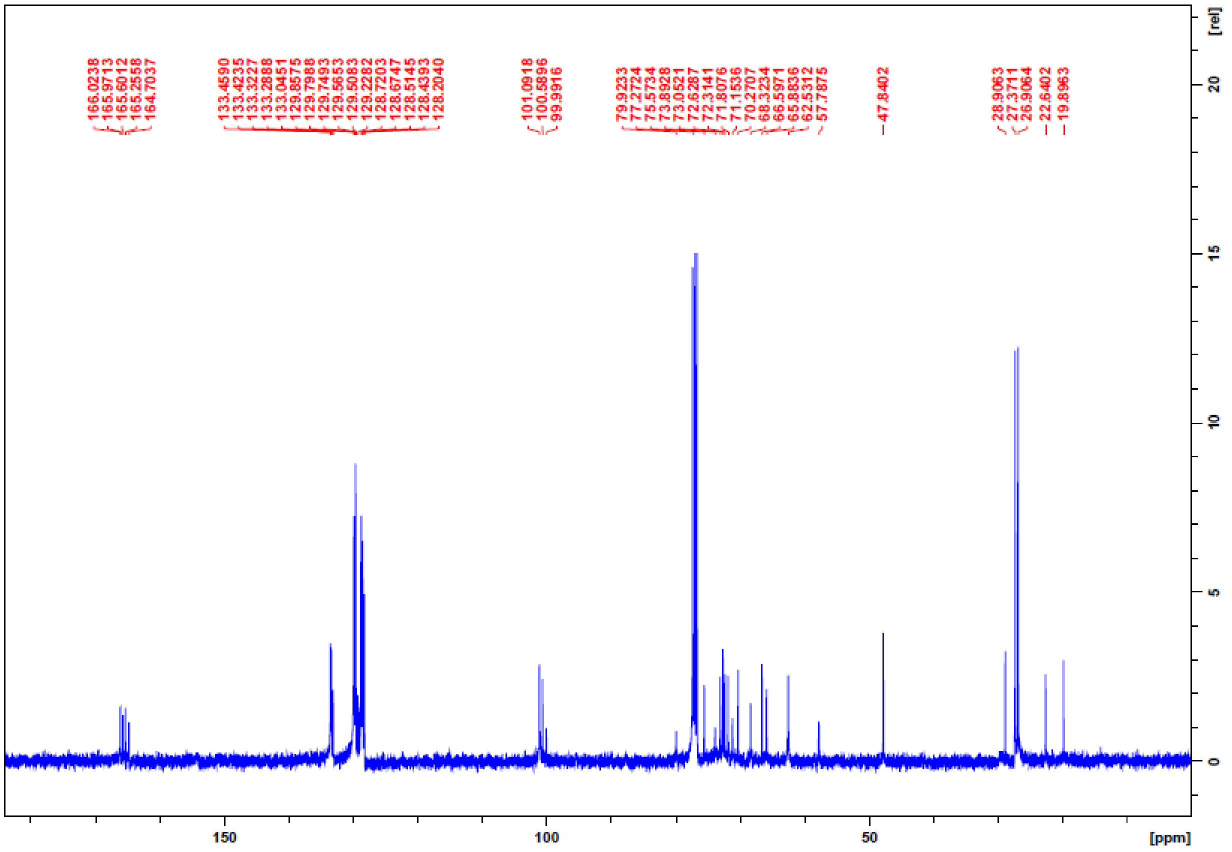

Compound 31: ^{1}H NMR, $CDCl_3$, 400 MHz

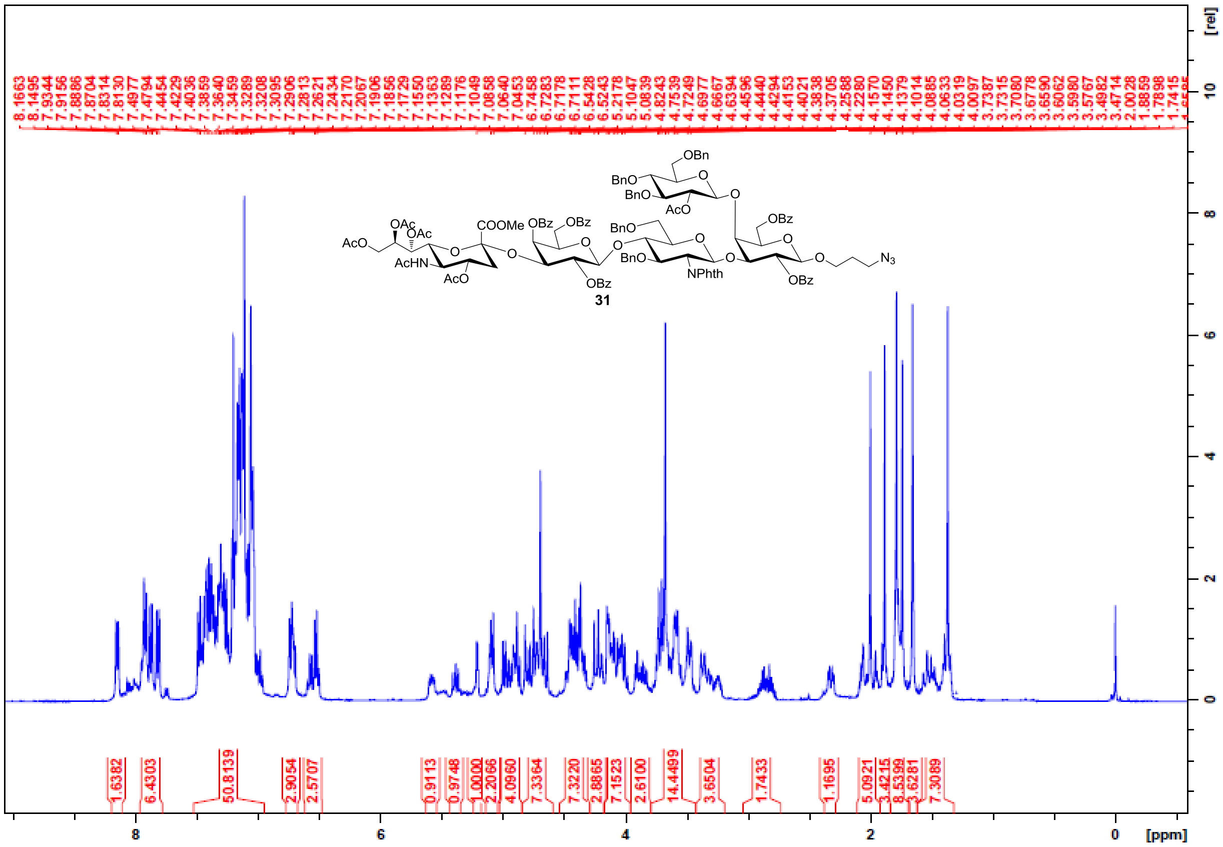

Compound 31: ^{13}C NMR, $CDCl_3$, 101 MHz

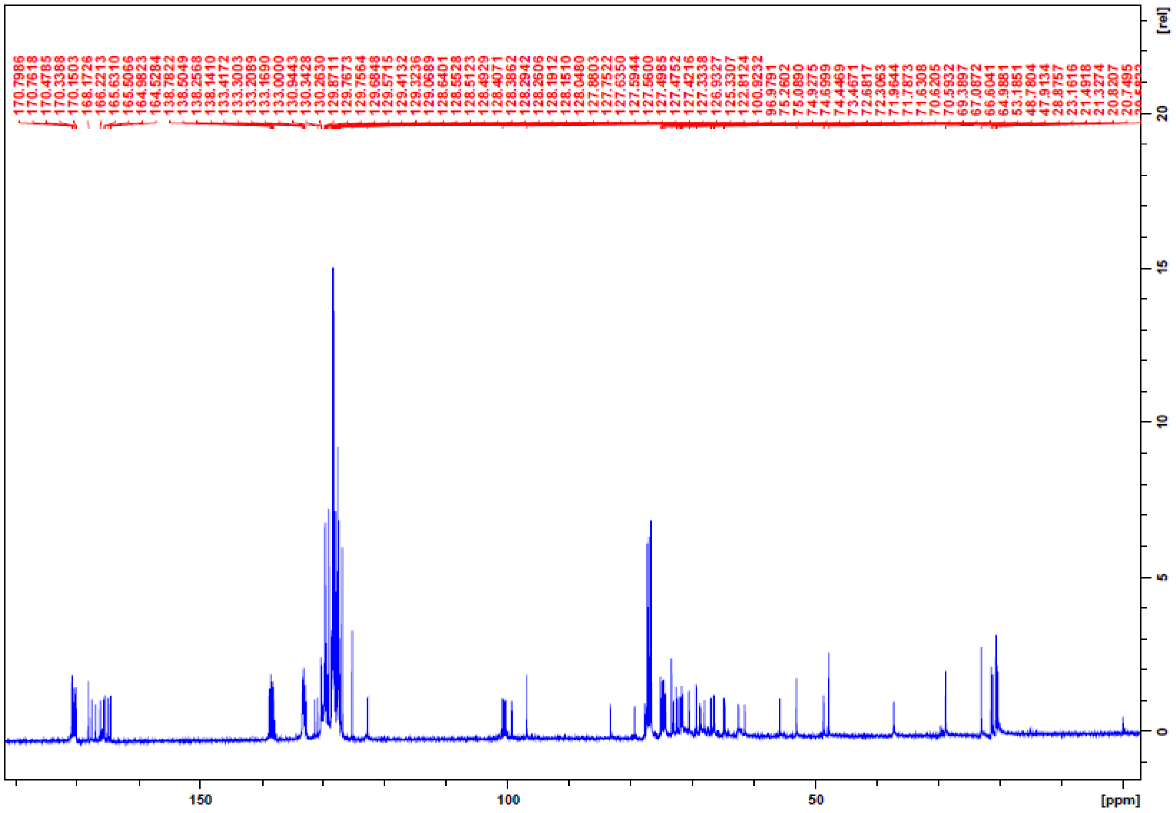

Compound 32: ^{1}H NMR, $CDCl_3$, 400 MHz

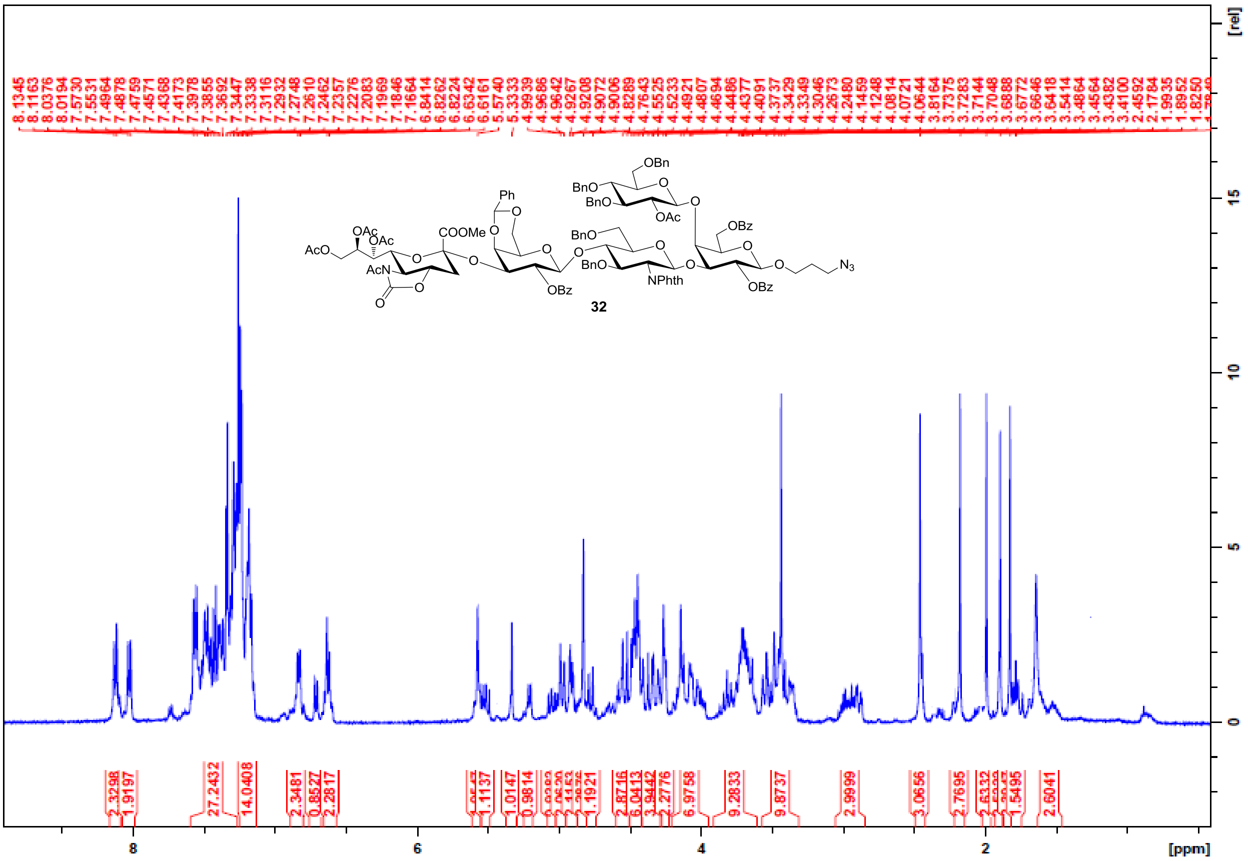

Compound 32: ^{13}C NMR, $CDCl_3$, 101 MHz

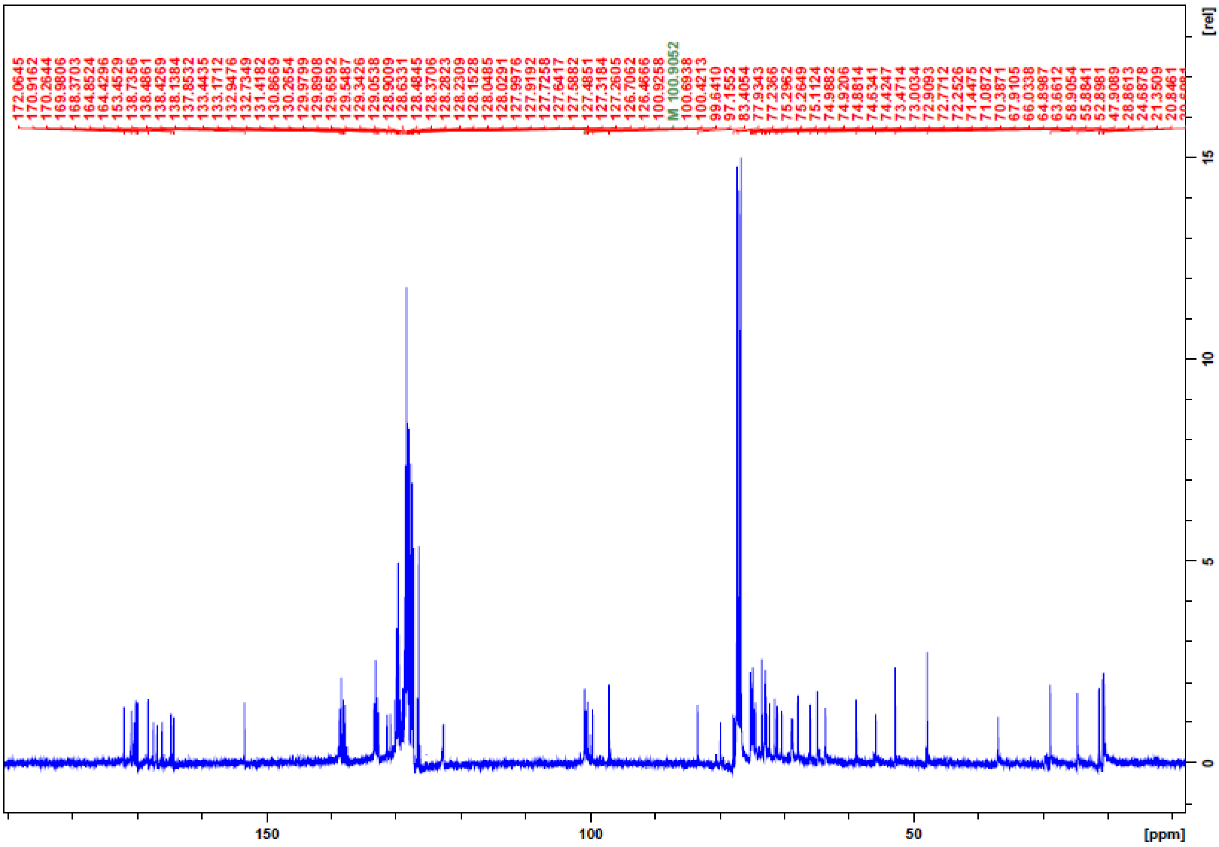

Compound 1: ^{1}H NMR, D_2O, 400 MHz

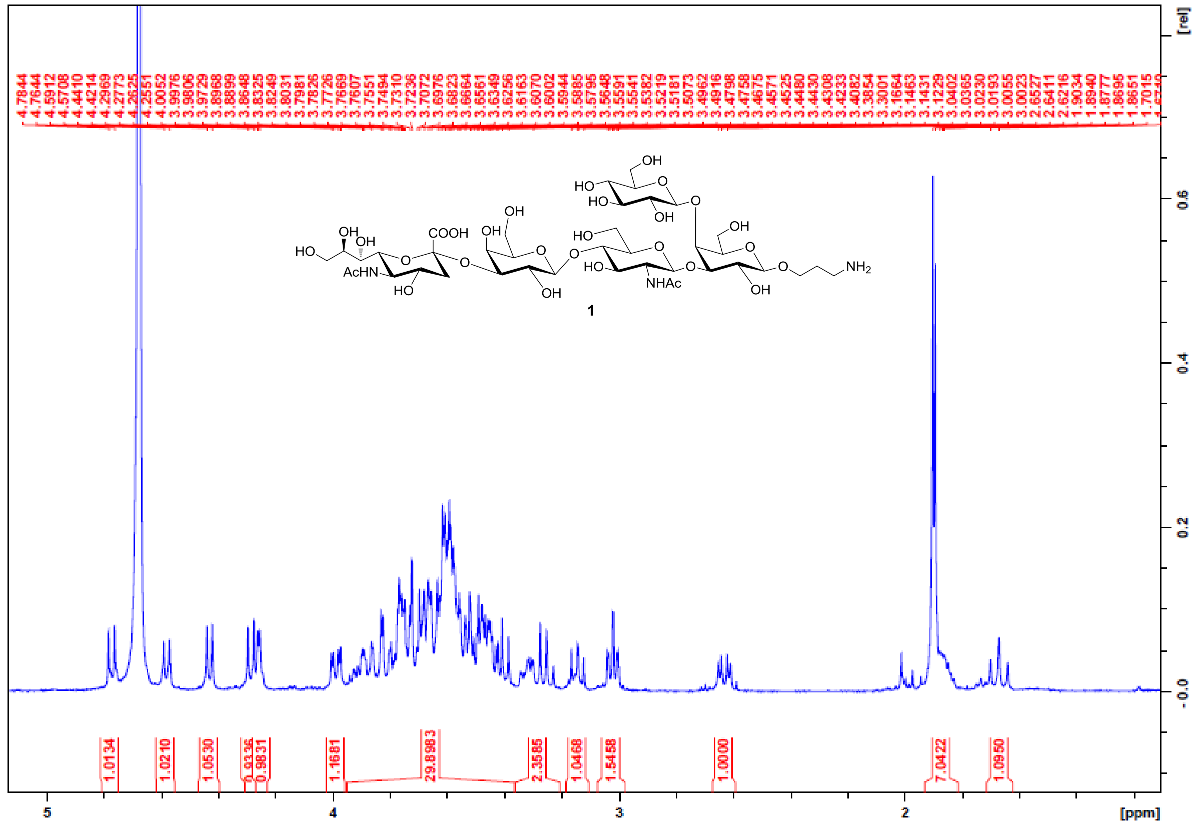

Compound 1: DEPT135, D_2O, 101 MHz

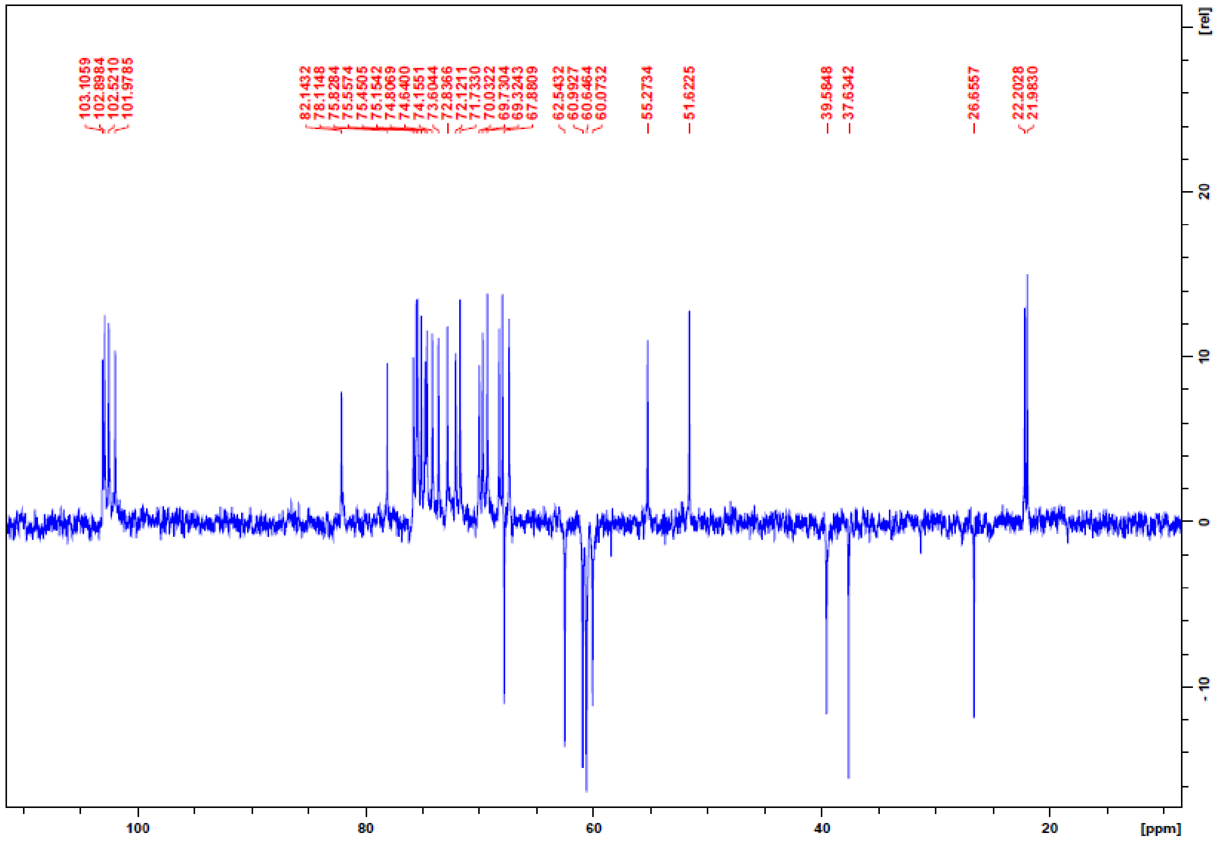

Compound 36: ^{1}H NMR, $CDCl_3$, 400 MHz

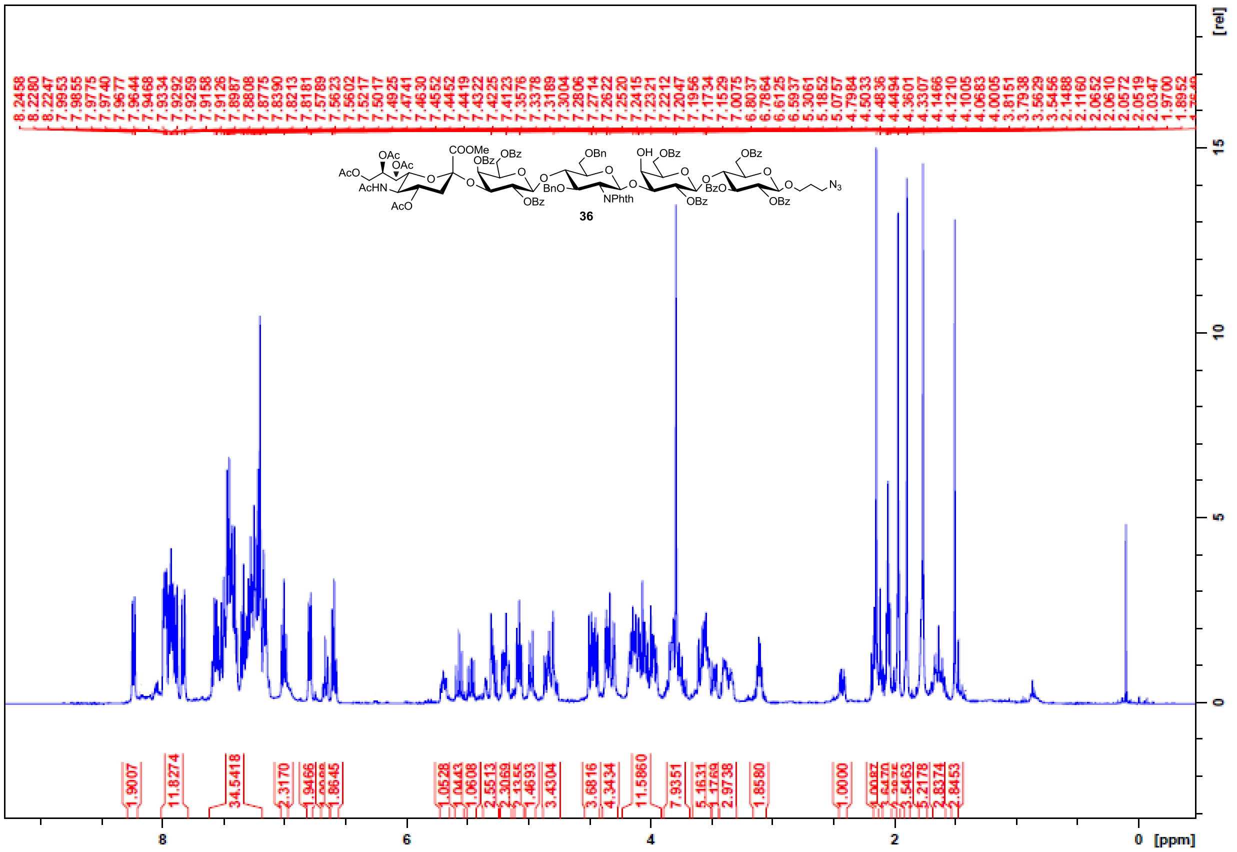

Compound 36: ^{13}C NMR, $CDCl_3$, 101 MHz

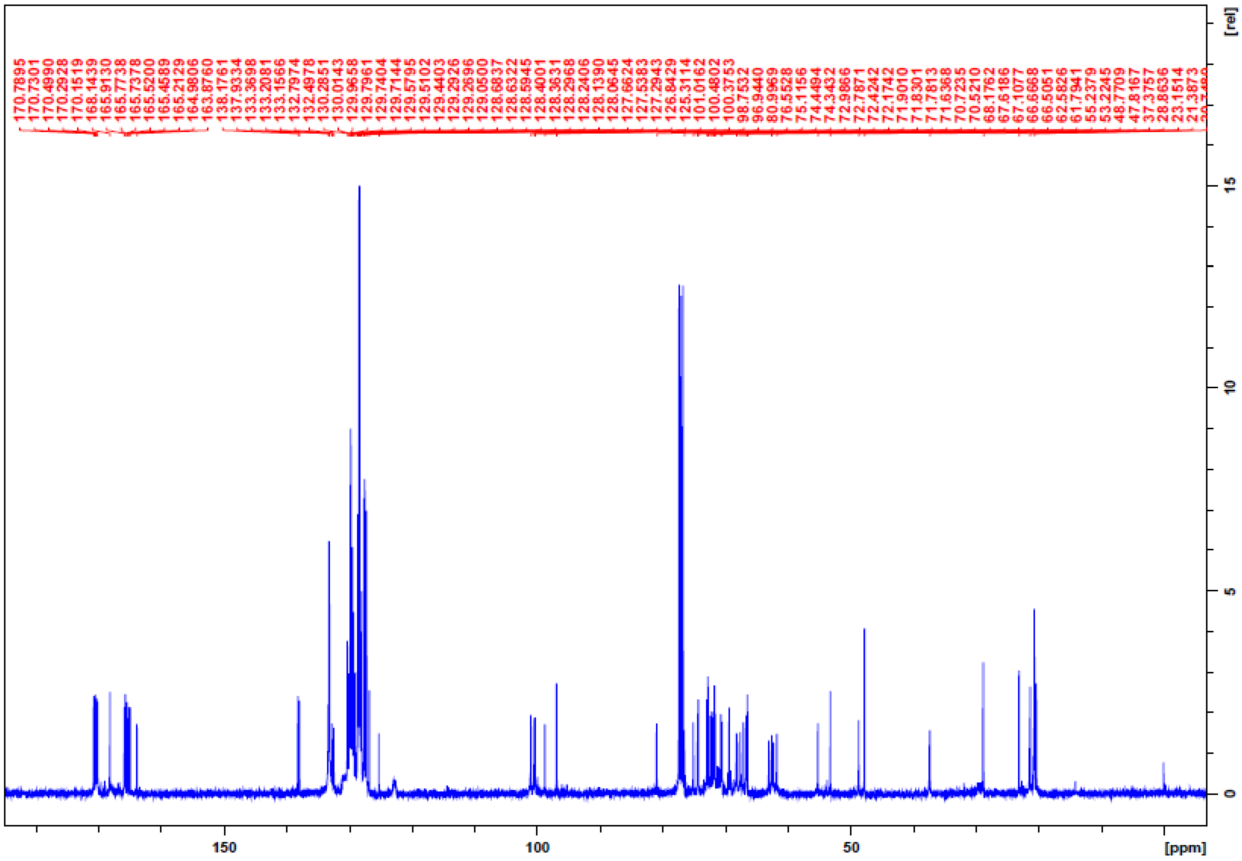

Compound 2: ^{1}H NMR, D_2O, 400 MHz

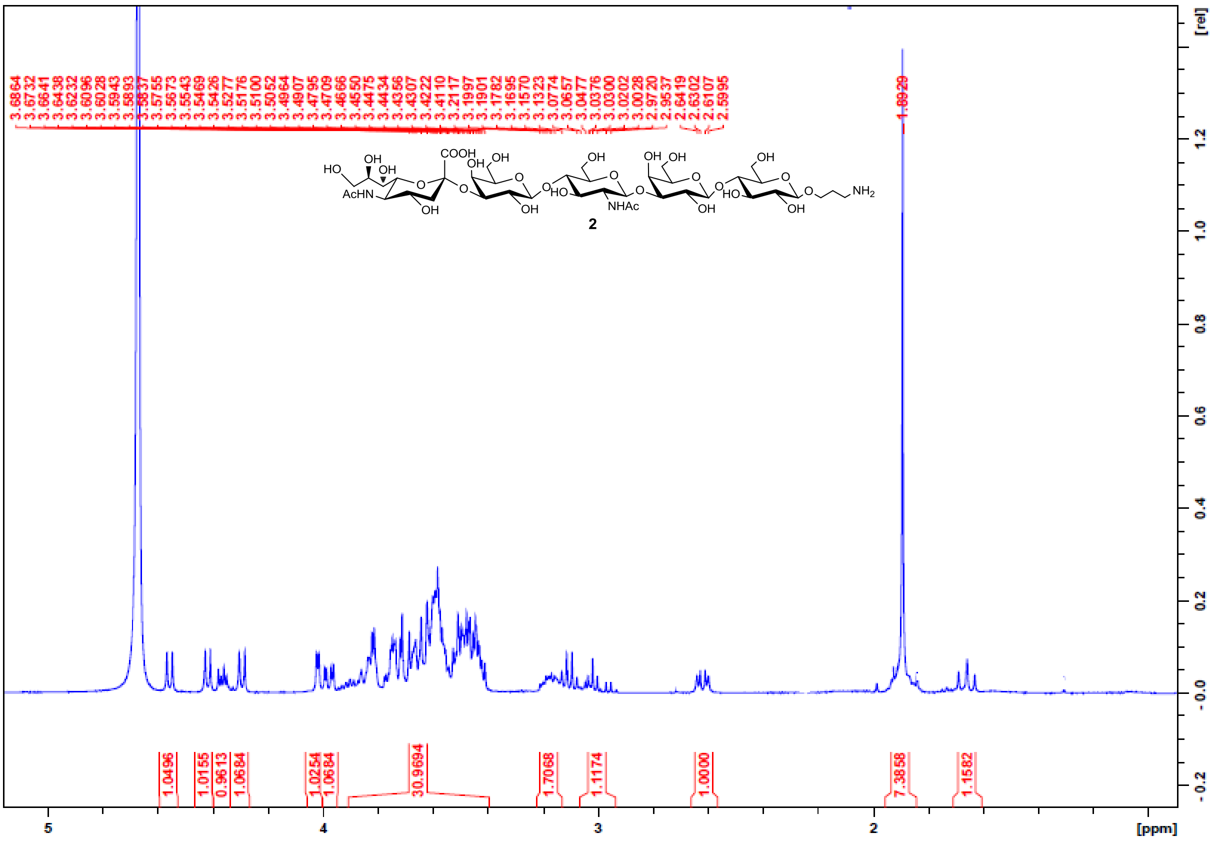

Compound 2: DEPT135, D_2O, 101 MHz

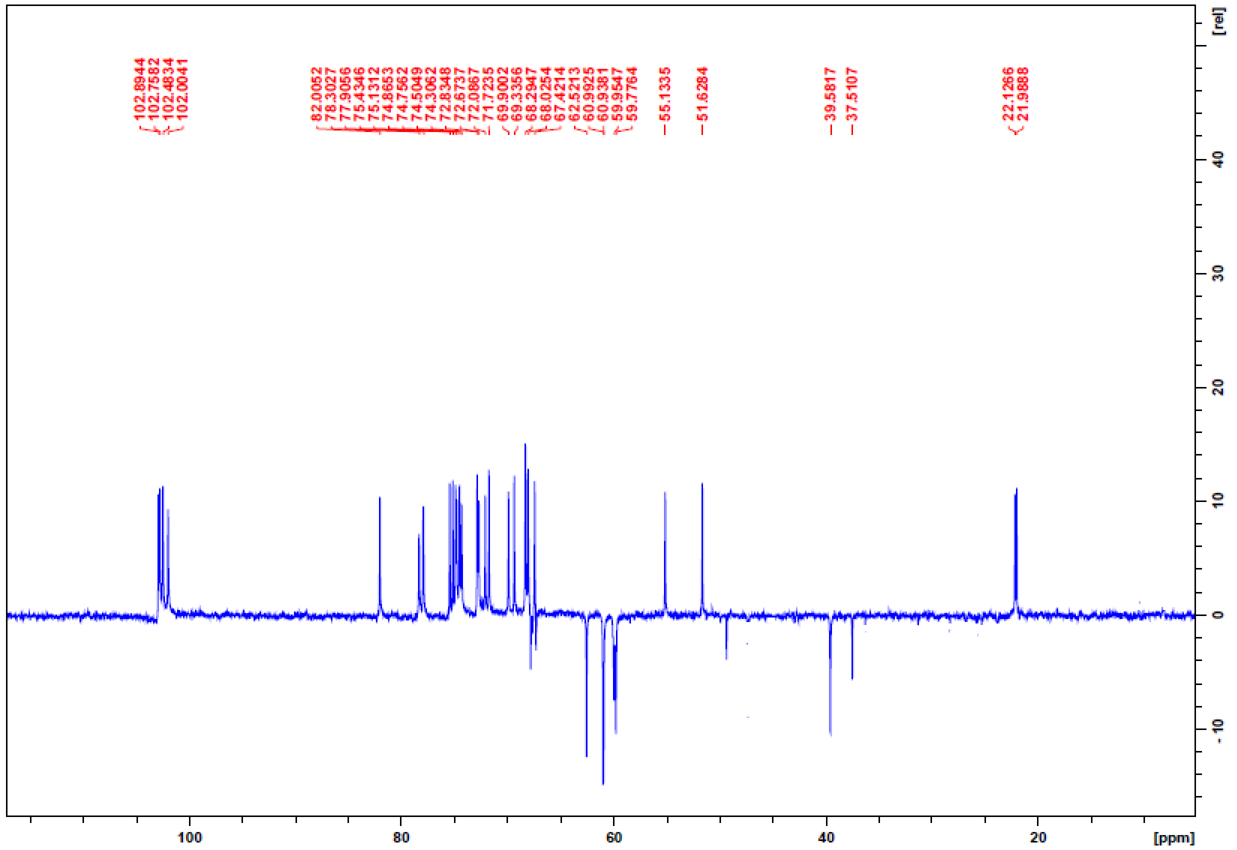

Compound 39: ^{1}H NMR, $CDCl_3$, 400 MHz

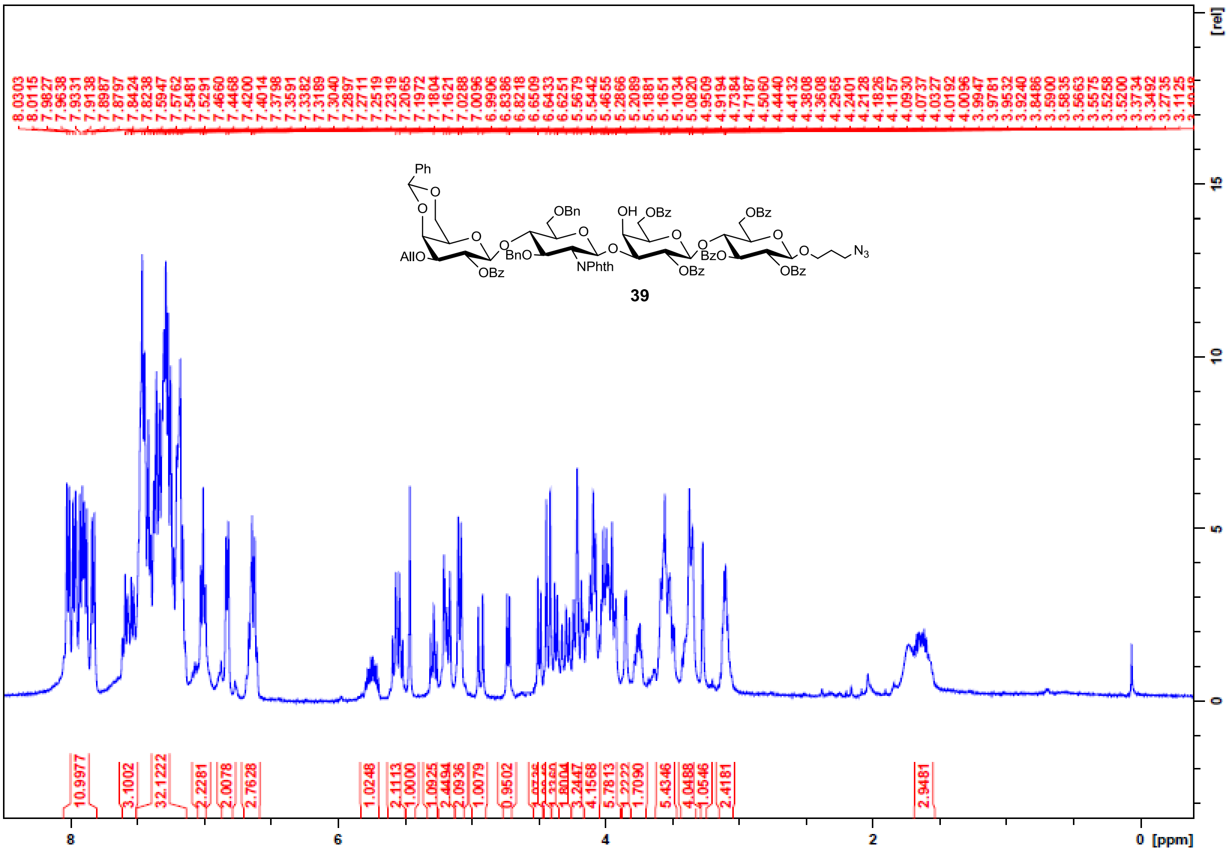

Compound 39: ^{13}C NMR, $CDCl_3$, 101 MHz

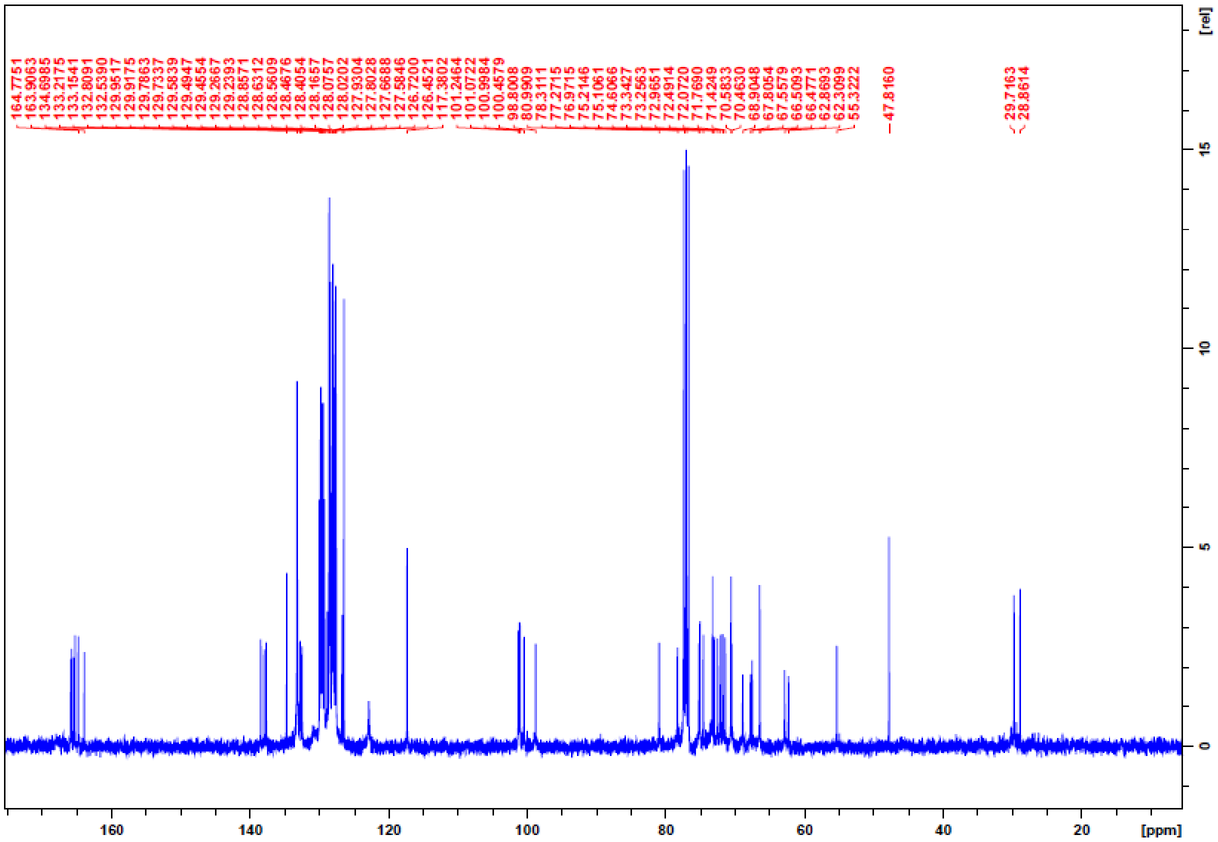

Compound 5: ^{1}H NMR, D_2O, 400 MHz

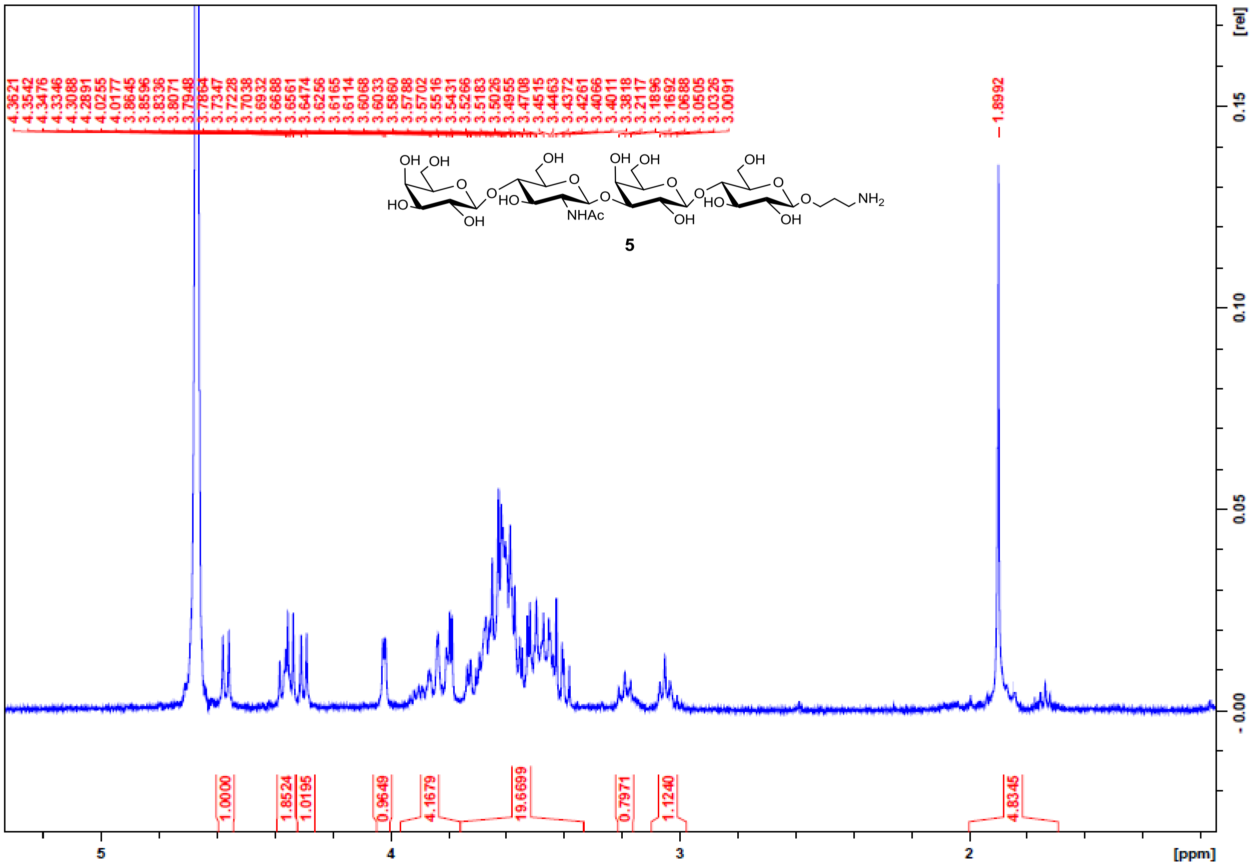

Compound 5: DEPT135, D_2O, 101 MHz

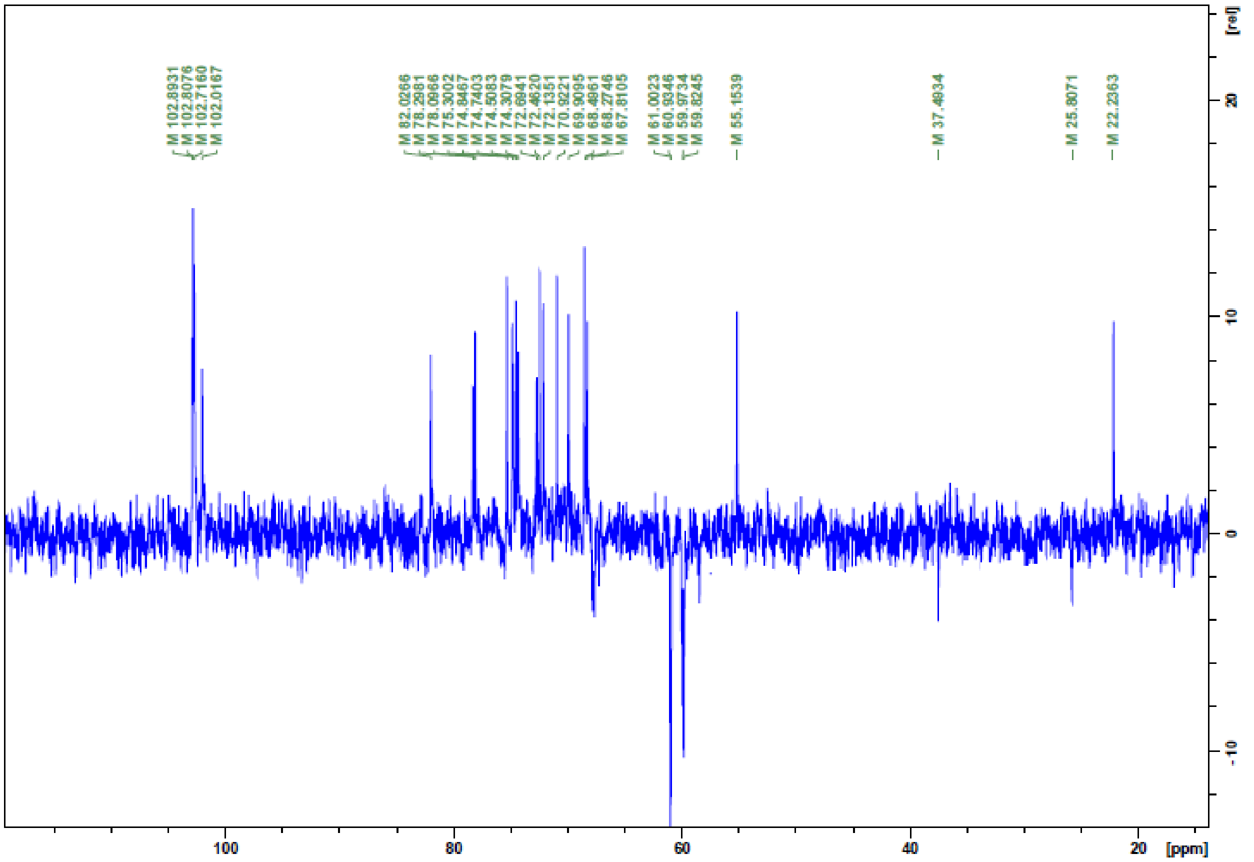

Compound 44: ^{1}H NMR, ACN-d$_3$, 400 MHz

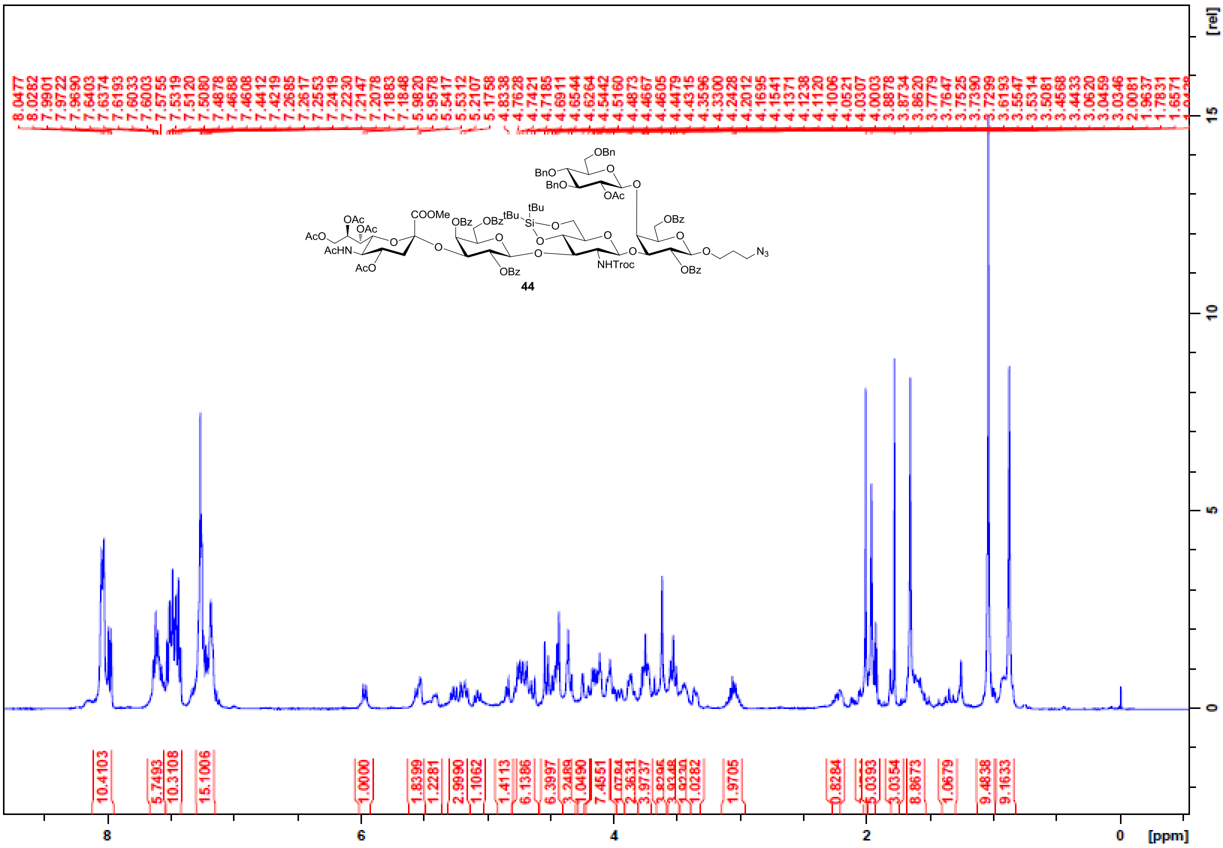

Compound 44: ^{13}C NMR, ACN-d$_3$, 101 MHz

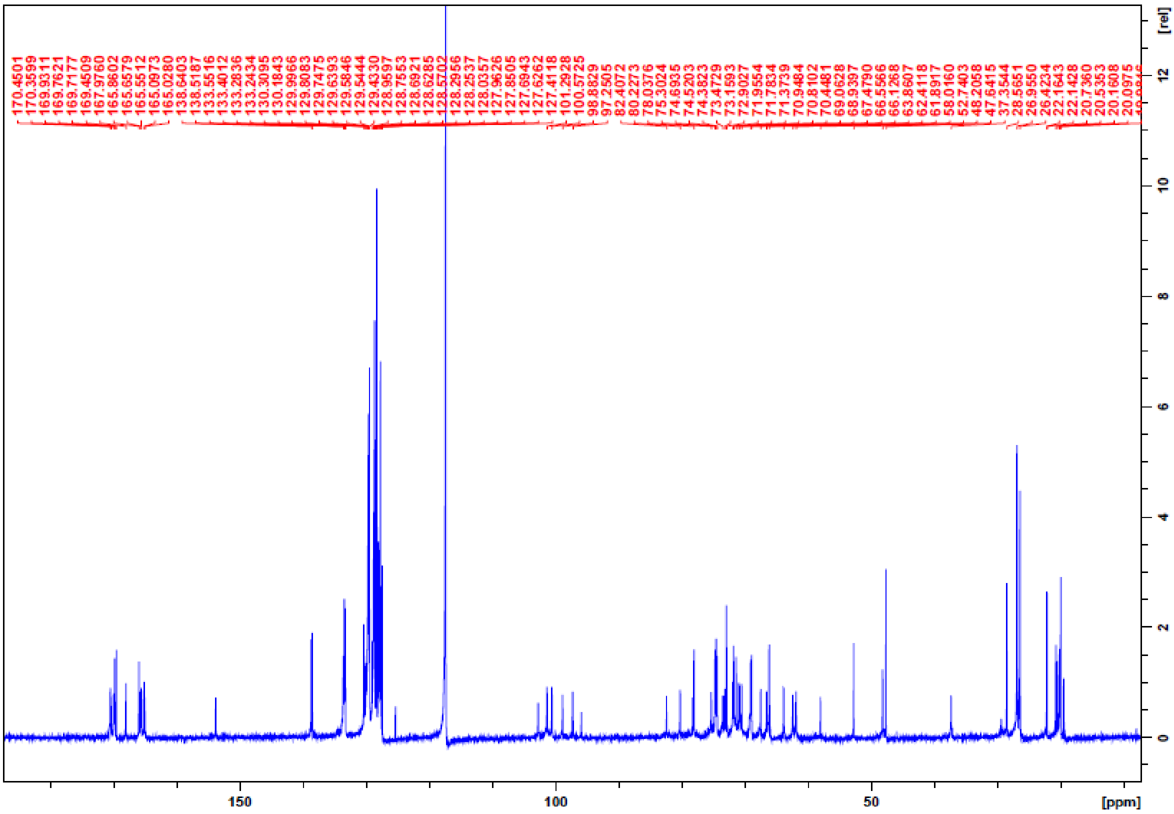

Compound 45: ^{1}H NMR, ACN-d$_3$, 400 MHz

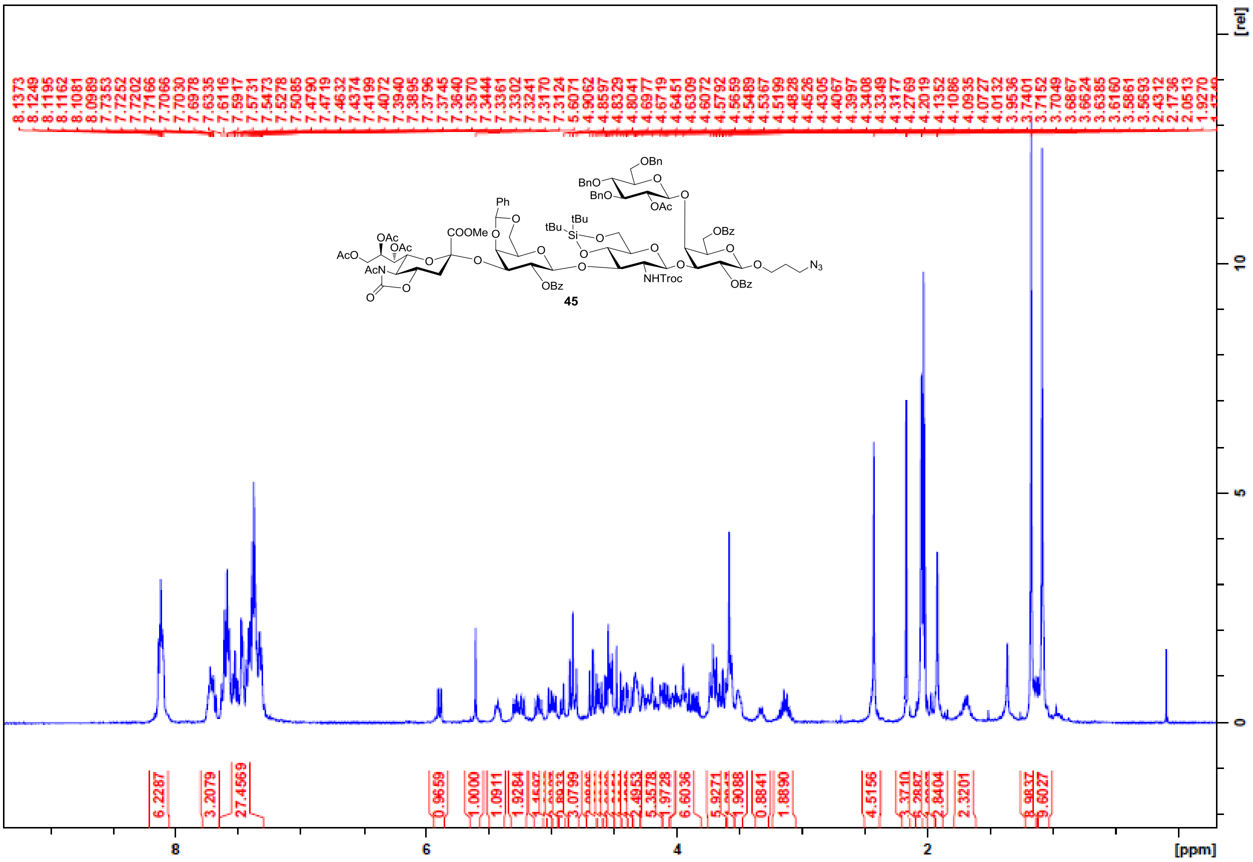

Compound 45: ^{13}C NMR, ACN-d$_3$, 101 MHz

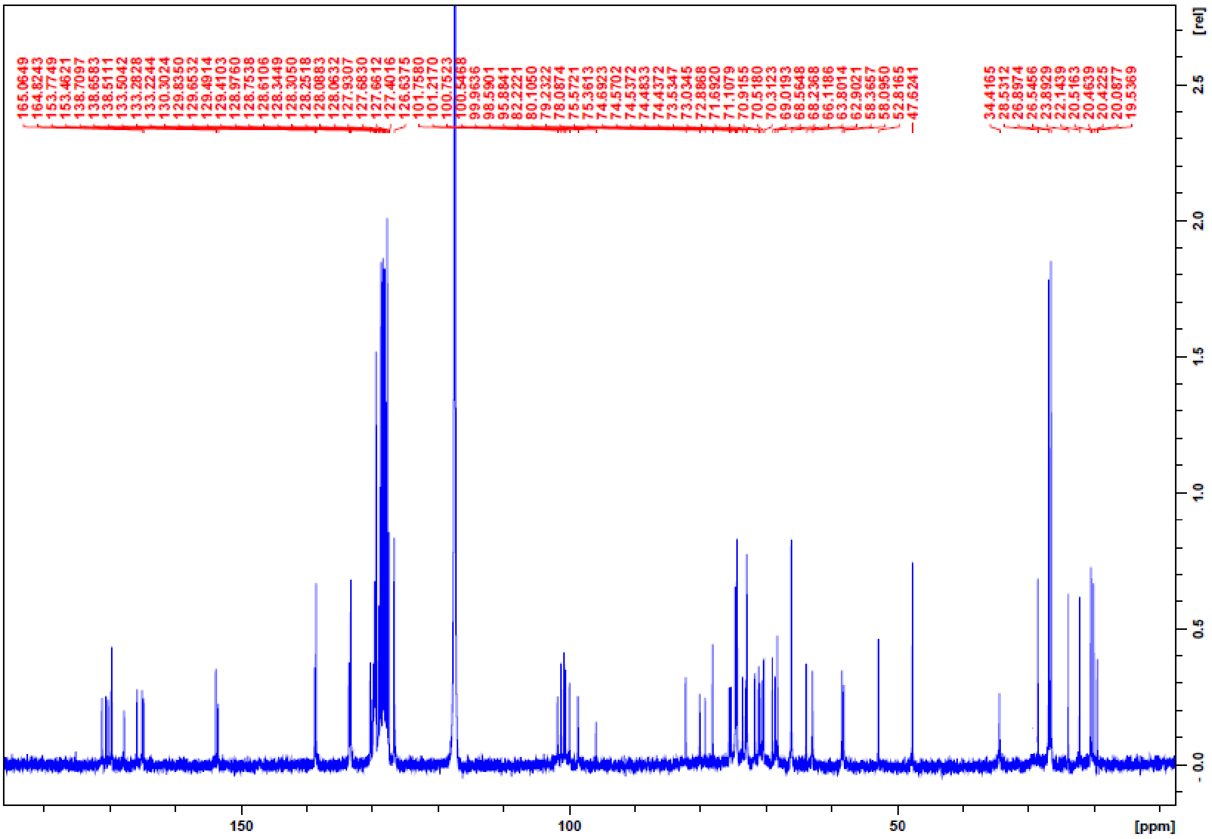

Compound 3: ^{1}H NMR, D_2O, 400 MHz

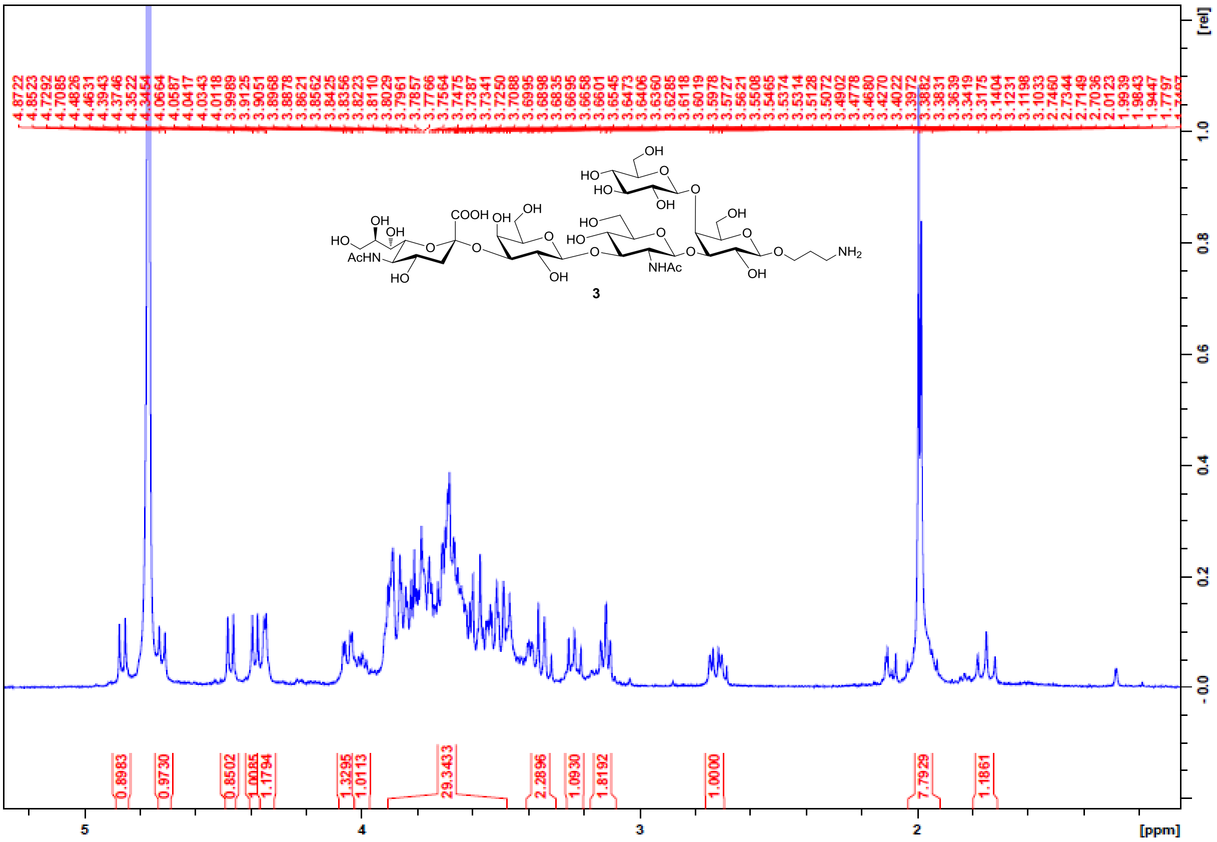

Compound 3: ^{13}C NMR, D_2O, 101 MHz

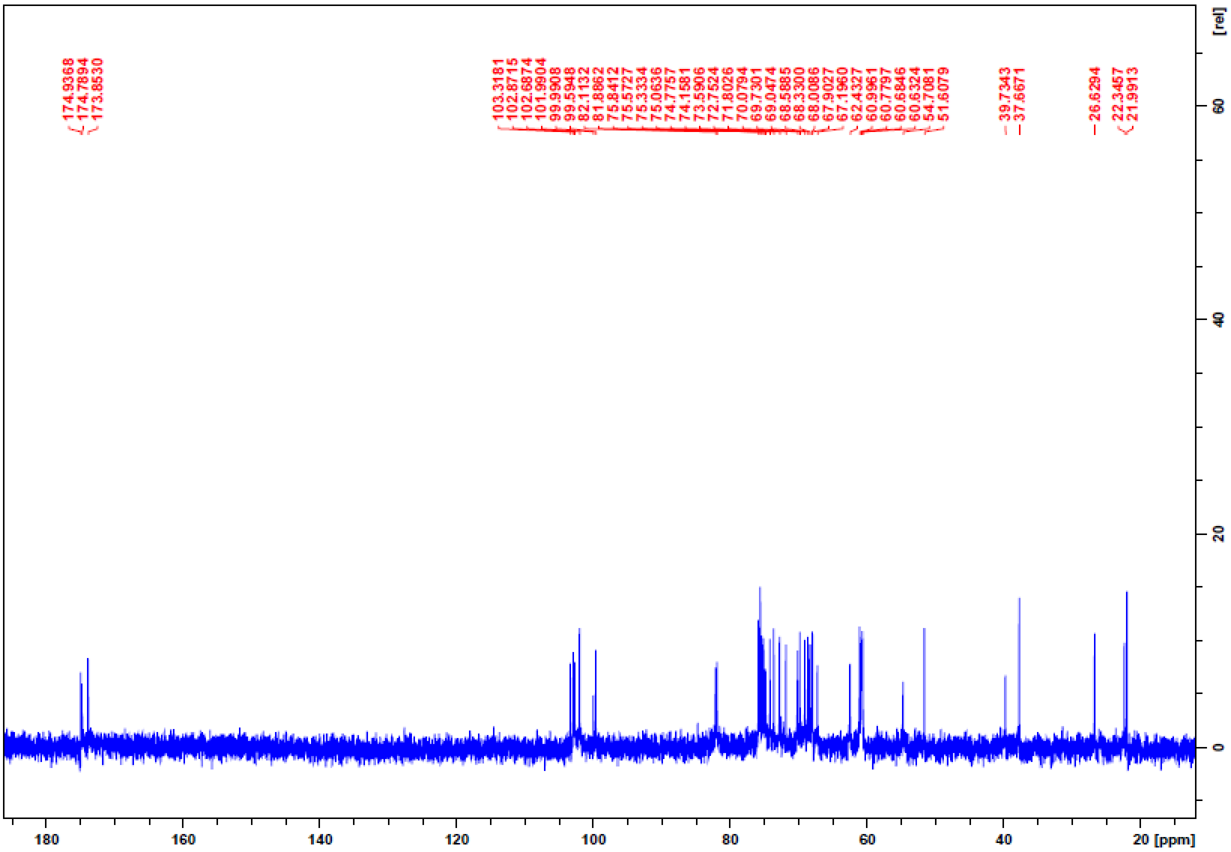

Compound 48: ^{1}H NMR, ACN-d$_3$, 400 MHz

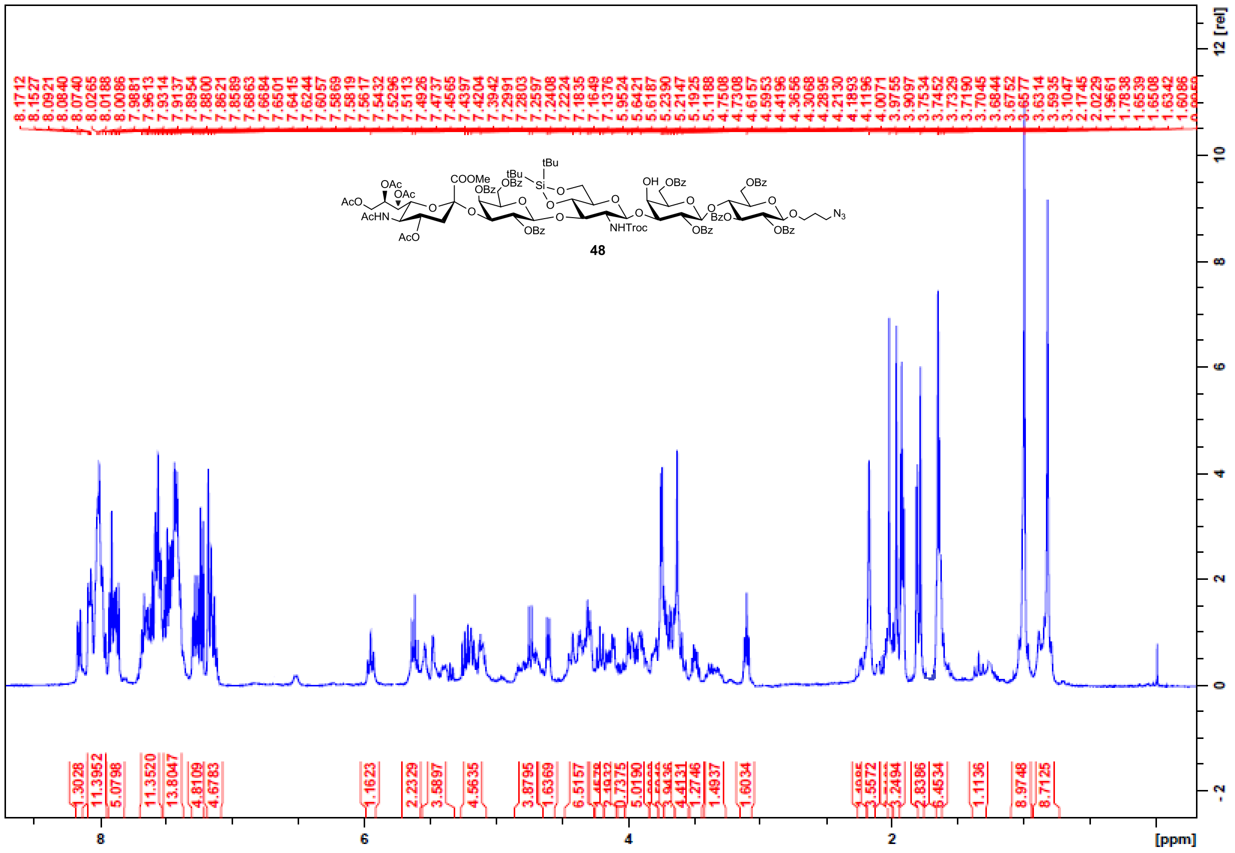

Compound 48: ^{13}C NMR, ACN-d$_3$, 101 MHz

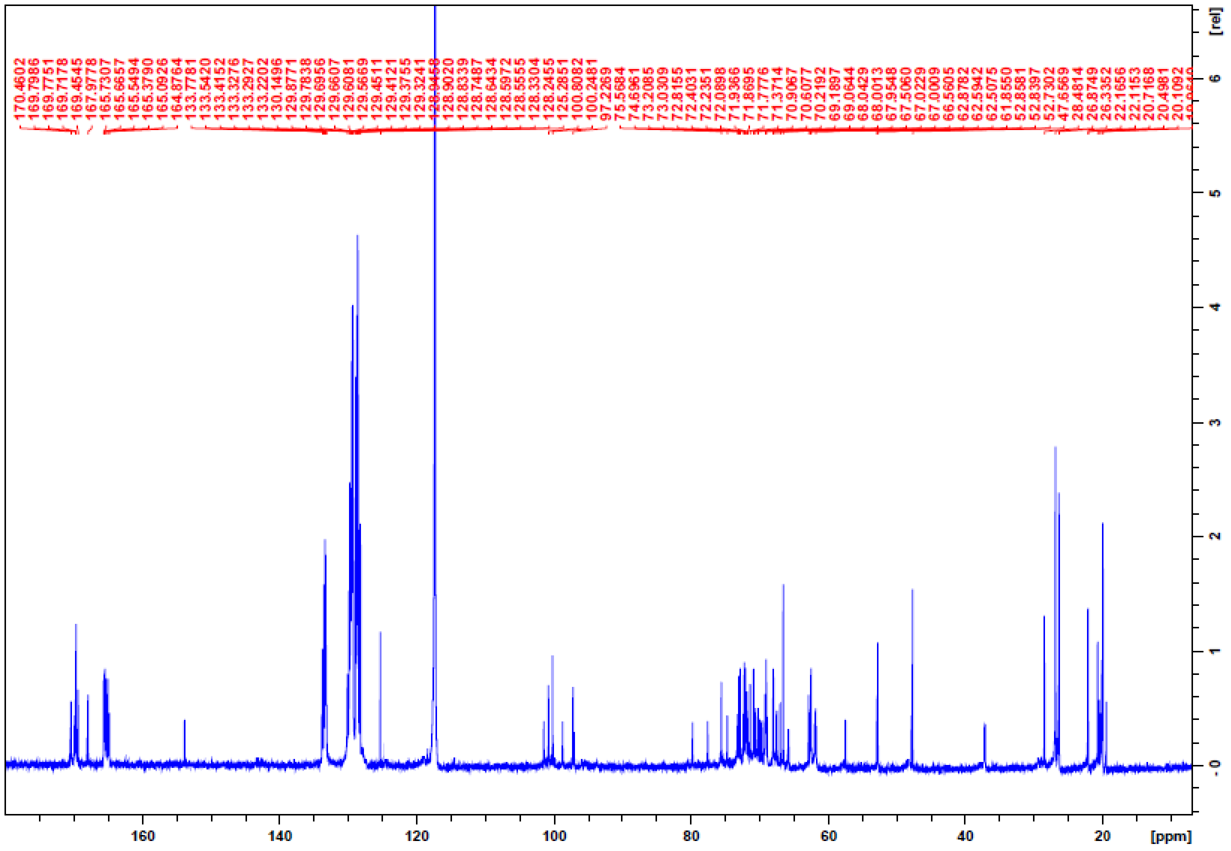

Compound 49: ^{1}H NMR, ACN-d$_3$, 400 MHz

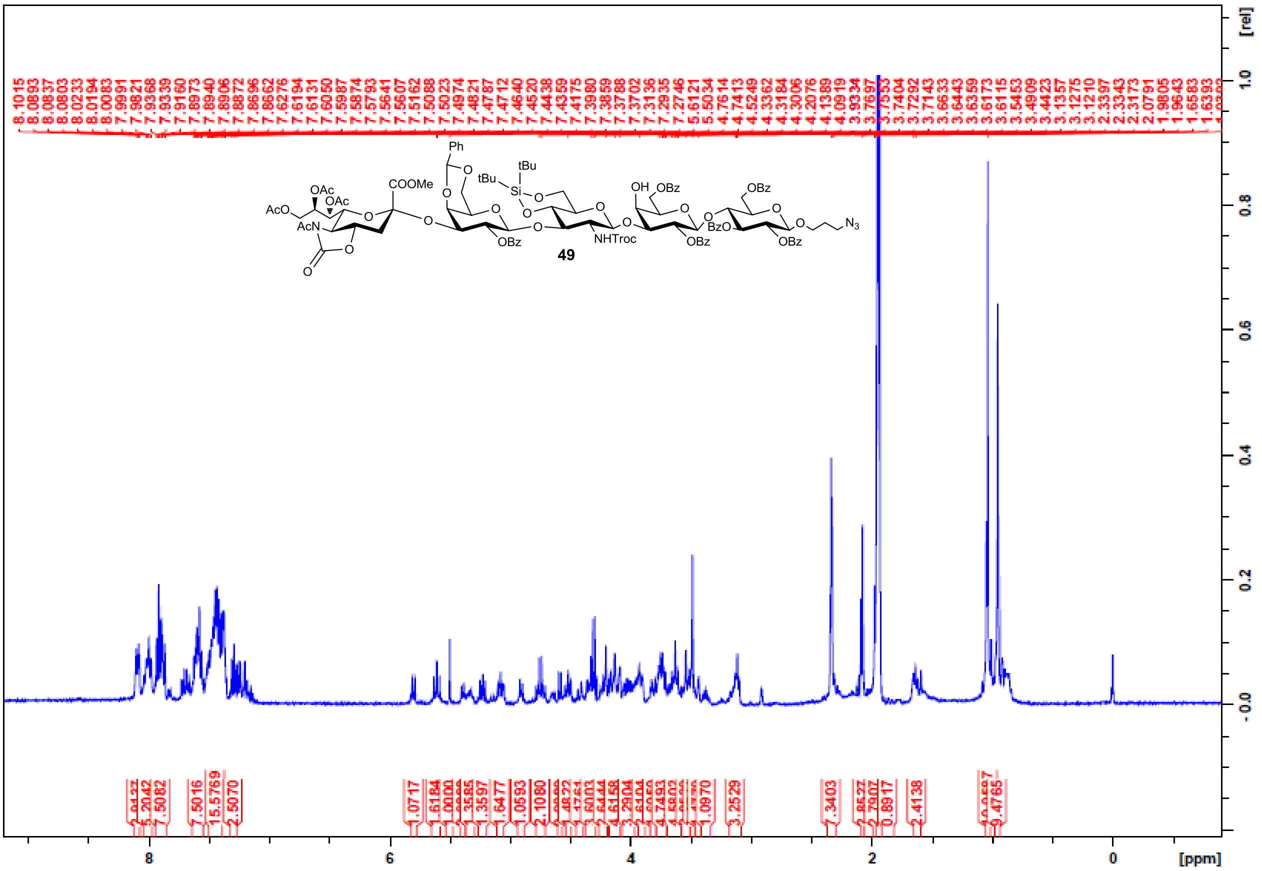

Compound 49: ^{13}C NMR, ACN-d$_3$, 101 MHz

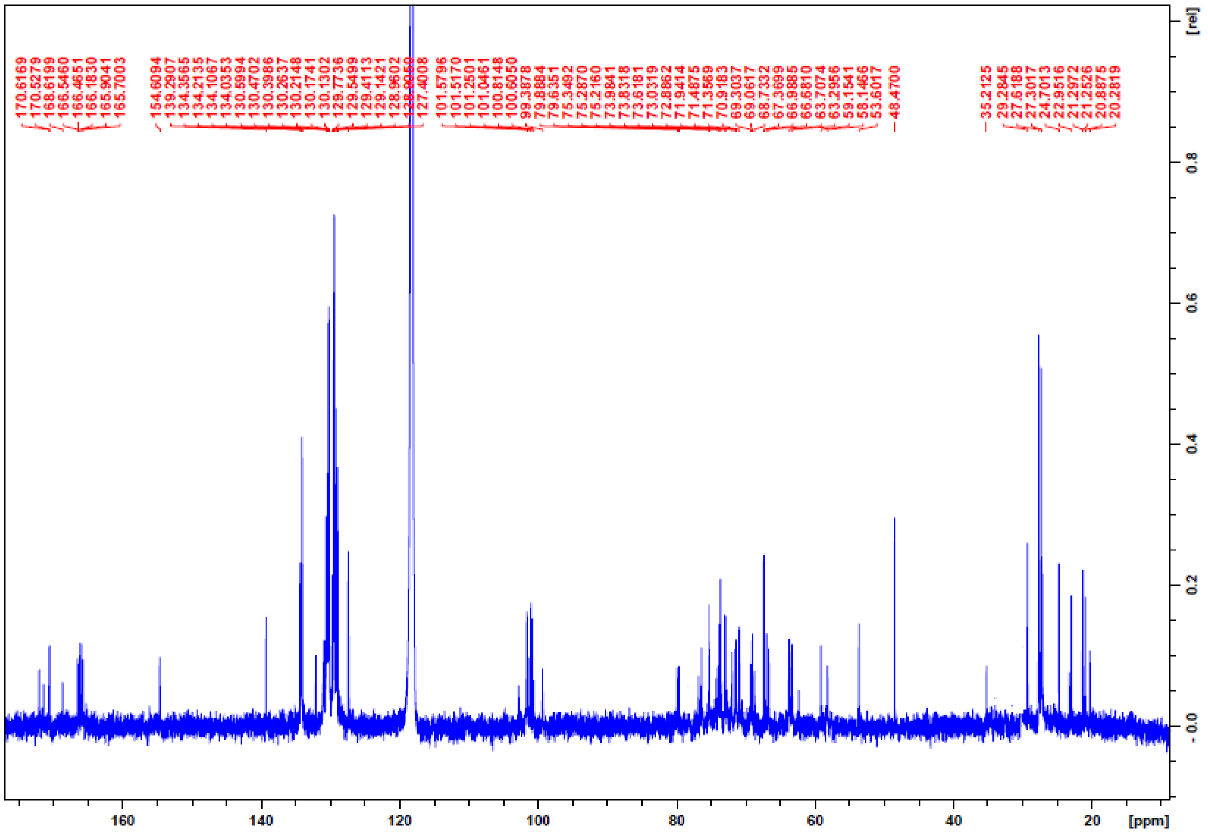

Compound 4: ^1H NMR, D_2O, 400 MHz

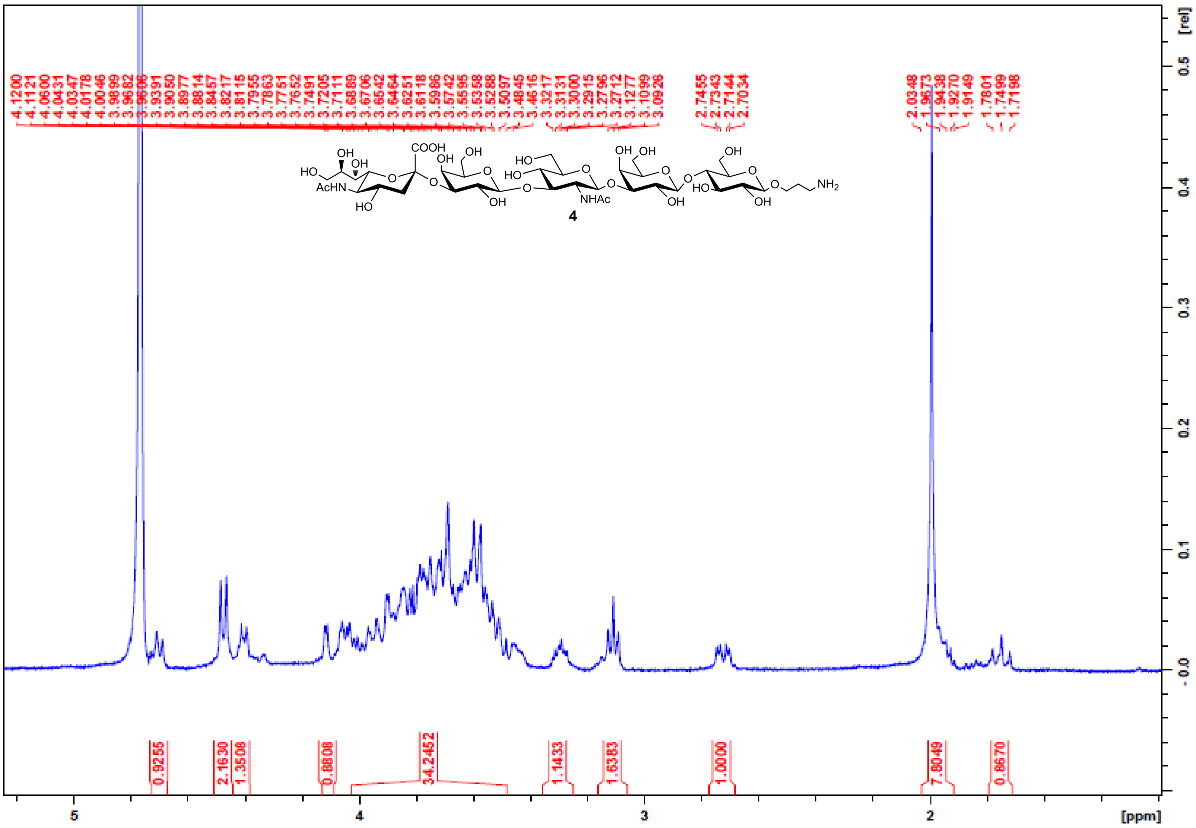

Compound 4: ^{13}C NMR, D_2O, 101 MHz

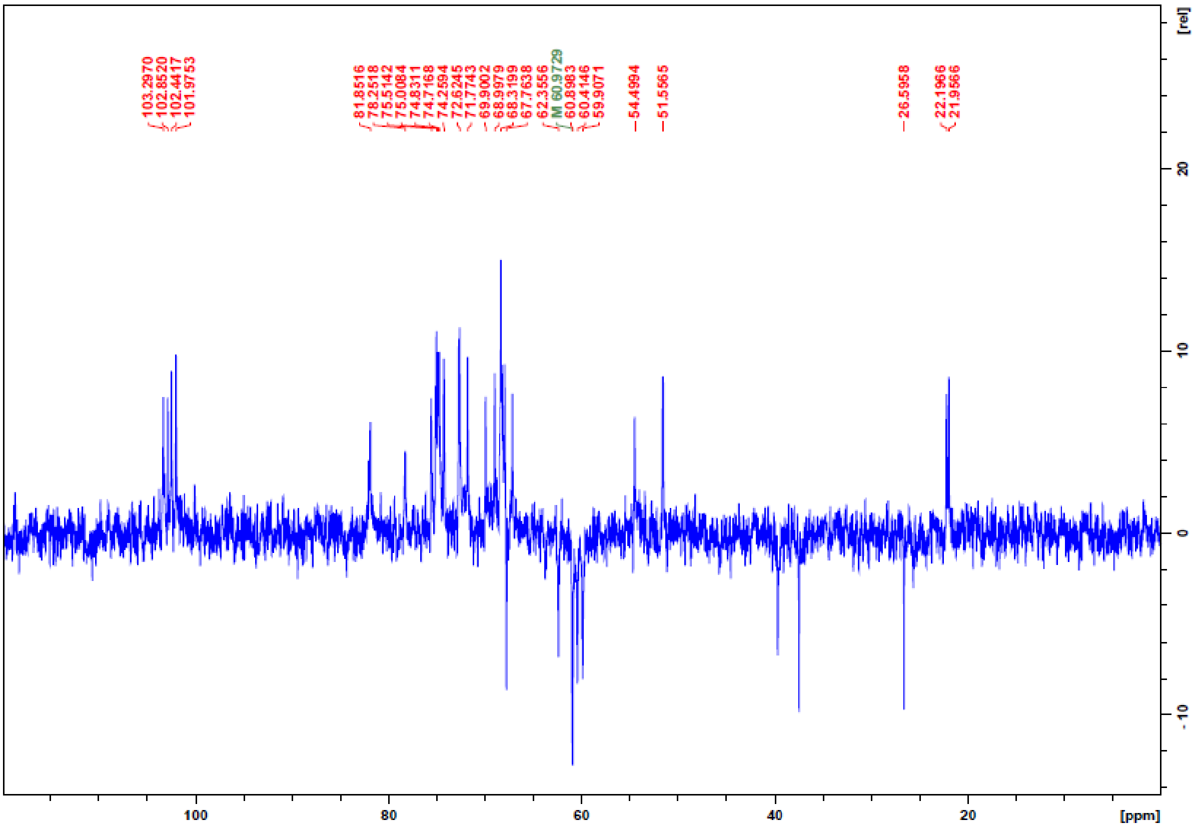

4. Conformational studies

Molecular Dynamics (MD) simulations. MD simulations were carried out for the GBS Ia and Ib pentasaccharides **1** and **3**, and for the polysaccharides (ten repeating units, 50 monosaccharide moieties). The key torsion angles are defined as follows:

Φ = H1-C1-O-Cx and Ψ= C1-O-Cx-Hx for the Gal/GlcNAc, Glc/Gal and GlcNAc/Gal linkages.

Φ = C1-C2-O-C3 and Ψ = C2-O-C3-H3 for the Neu5Ac/Gal linkage.

MD simulations were performed using the AMBER12[9] and AMBER16[10] with the GLYCAM06j-1[11] force field. Long MD simulations of 2.5 μs were employed to access to the conformational and dynamic information of the polysaccharides, while 200ns were used for the pentasaccharides. The molecules were built using the GLYCAM-web carbohydrate builder web tool.[12] Then, the resulting geometries were extensively minimized using conjugate gradients and the resulting structures were used as starting geometries for the MD simulations in explicit solvent. The molecules were solvated in a theoretical box of explicit TIP3P waters and the solute atoms were positioned at least at 10 Å from the solvent box edge. The equilibration phase consisted on energy minimization of the solvent followed by an energy minimization of the entire system without restraints. The system was then heated up to 300 K during 100 ps followed by 2 ns dynamics at constant temperature of 300 K, controlled by the Langevin thermostat, and constant pressure of 1 atm. During the simulations, the SHAKE algorithm[13] was turned on and applied to all hydrogen atoms. A cut-off of 8 Å for all non-bonded interactions was adopted. An integration time step of 2 fs was employed and periodic boundaries conditions were applied throughout. During all simulations, the particle mesh Ewald (PME) method was used to compute long-range electrostatic interactions.[14-17] Minimization, equilibration and production phases were carried out by the pmemd.cuda[18-20] module of AMBER 12, while the analyses of the simulations were performed using cpptraj module from AMBERTOOLS 12 and 16. Data processing and 2D plots were carried out using GNUplot Software and Excel.

NMR experiments. NMR spectra of the pentasaccharides were acquired at 298K on a Bruker Avance 600 MHz spectrometer, while the polysaccharides were acquired at 313K on a Bruker Avance 800 MHz spectrometer. Standard Bruker pulse sequences were used for assignment of the for ^{1}H and ^{13}C resonances. NOE-derived distances for the pentasaccharides were obtained from ROESY spectra with 200ms spin-lock, while for the polysaccharides NOESY spectra with 20 ms mixing times were used. NMR samples were prepared dissolving 1 mg of material in 500 µL of D_2O. Spectra were processed and analysed in TOPSPIN 2.0 (Bruker) software.

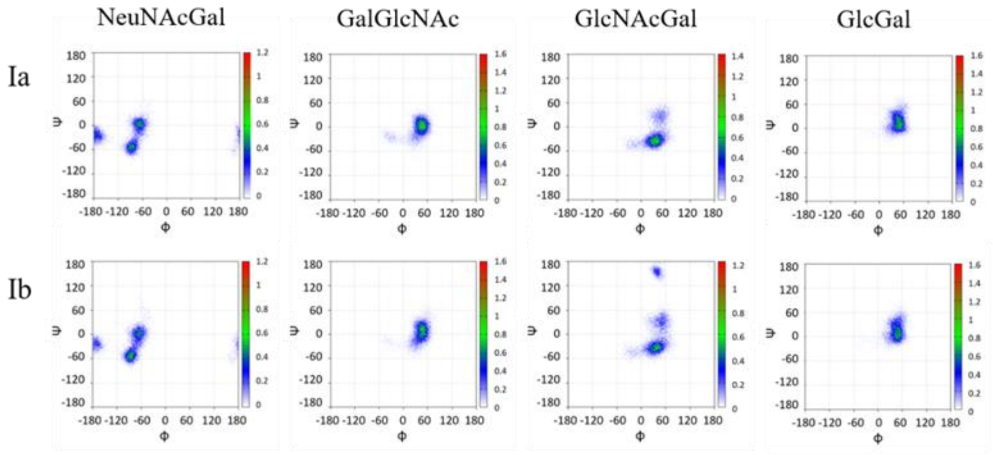

Figure S1 Glycosidic linkage analysis for GBS Ia and Ib pentasaccharides: φ/ψ plots for representative glycosidic bonds along 200 ns of MD simulation.

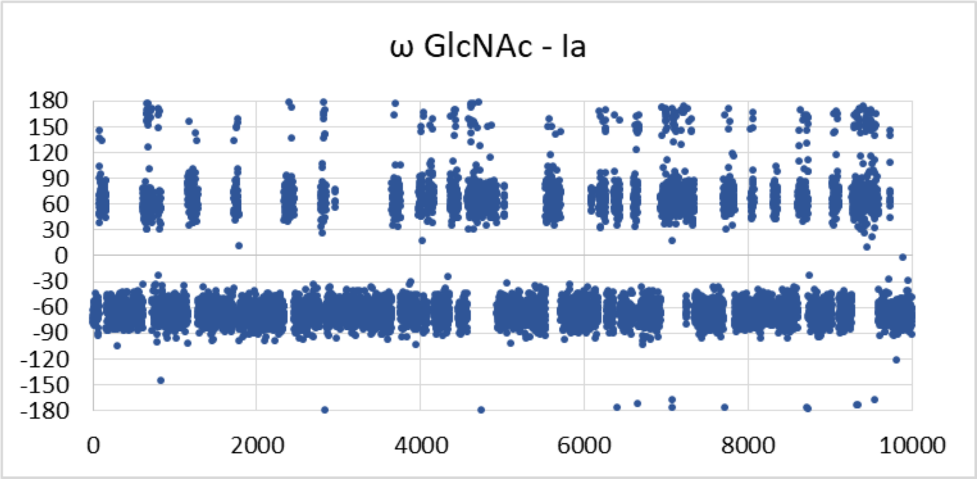

Figure S2 ω dihedral angle analysis for GlcNAc residue of GBS Ia pentasaccharide along 200 ns of MD simulation. The ω torsion angle is define as O5-C5-C6-O6.

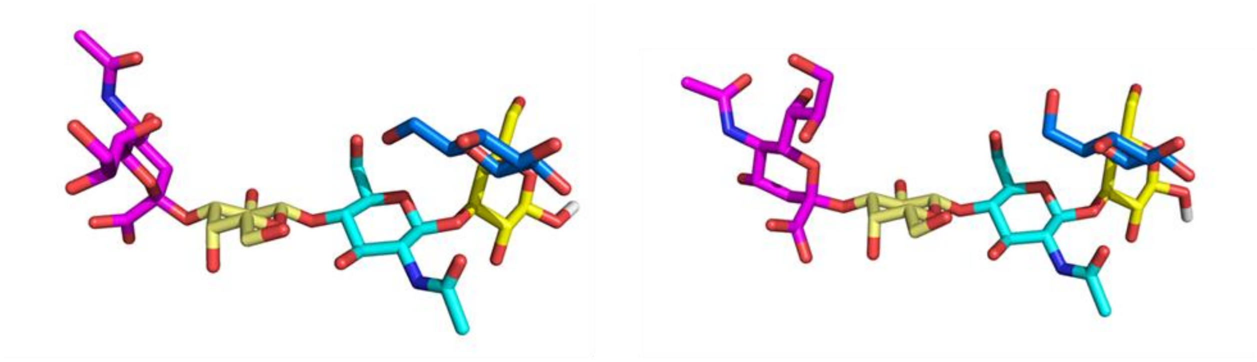

Figure S3 Perspectives of the two populations, deduced by ROESY NMR experiments, which define the conformational behaviour of the pentasaccharide repeating unit of GBS Ia. (Left) the φ_S torsion angle adopts the major trans (t) geometry. (Right) the φ_S torsion angle shows the minor -g conformation. The computed proton-proton distances are given in Table 3.

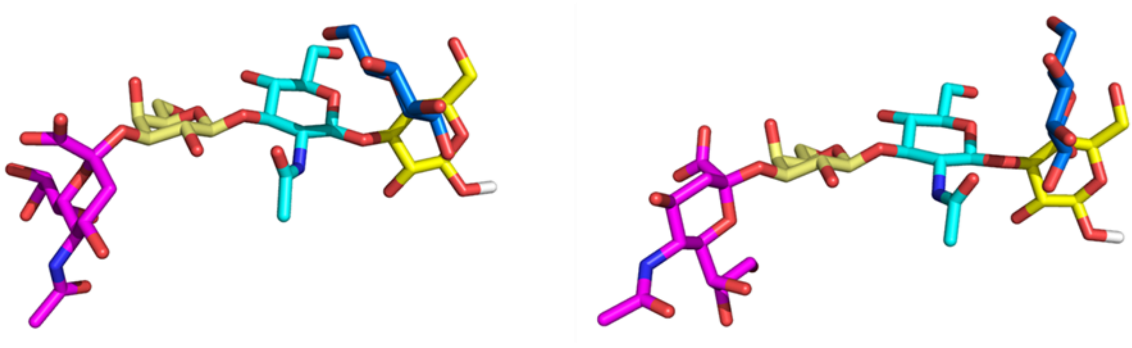

Figure S4 Perspectives of the two populations, deduced by ROESY NMR experiments, which define the conformational behaviour of the pentasaccharide repeating unit of GBS Ib. (Left) the φ_S torsion angle adopts the major trans (t) geometry. (Right) the φ_S torsion angle shows the minor -g conformation. The computed proton-proton distances are given in Table 4.

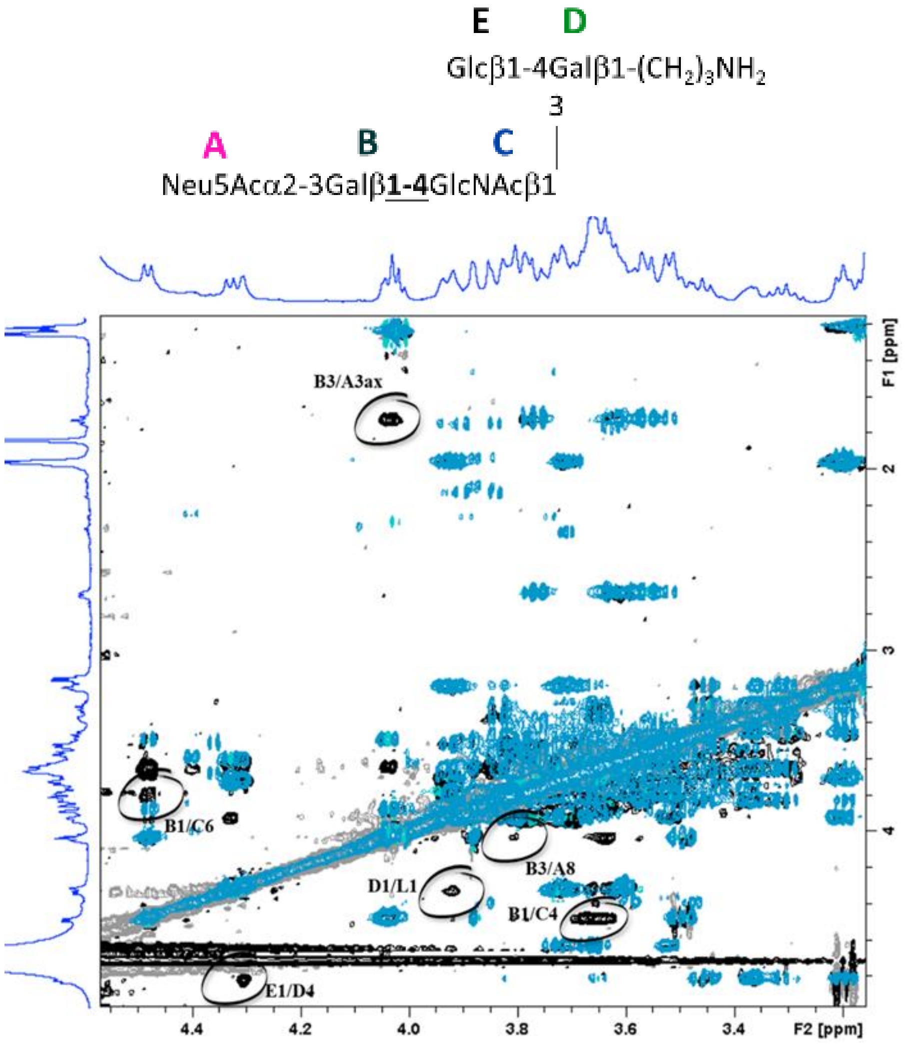

Figure S5 ROESY spectrum (200 ms mixing time, in black) and TOCSY (80 ms mixing time, in blue) of the pentasaccharide repeating unit of of GBS Ia (600MHz). The key inter-residue NOEs are indicated.

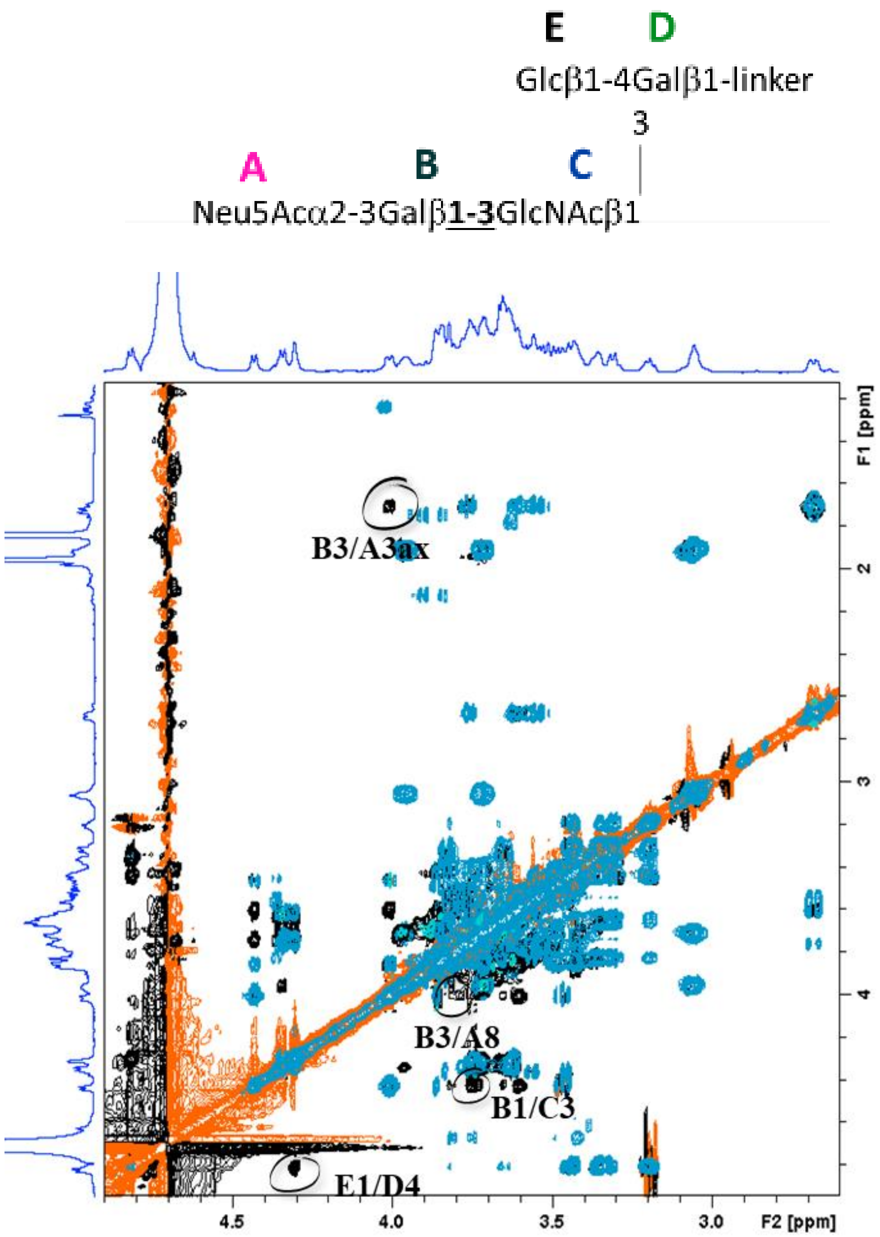

Figure S6 ROESY spectrum (200 ms mixing time, in black and orange) and TOCSY (80 ms mixing time, in blue) of the pentasaccharide repeating unit of of GBS Ib (600MHz). The key inter-residue NOEs are indicated.

Table S1. Comparison between the interglycosidic interproton (Å) distances for the CPS Ia pentasaccharide **1** and polysaccharide.

	MD distance	
	Total average penta	Total average poly
H3Gal-H3eqNeuNAc	4,3	4,3
H3Gal-H3axNeuNAc	3,7	3,7
H3Gal-H8NeuNAc	3,9	3,9
H1Gal-H4GlcNAc	2,4	2,4
H1Gal-H6GlcNAc	3,0	3,1
H1Gal-H6bGlcNAc	3,9	4,0
H1Glc-H4Gal	2,5	2,6
H1GlcNAc-H3Gal	2,4	2,4

Table S2. Comparison between the interglycosidic interproton (Å) distances for the CPS Ib pentasaccharide **3** and polysaccharide.

	MD distance	
	Total average penta	Total average poly
H3Gal-H3axNeuNAc	3,8	3,8
H3Gal-H3eqNeuNAc	4,3	4,3
H3Gal-H8NeuNAc	3,9	3,8
H1Gal-H3GlcNAc	2,4	2,4
H1Gal-H2GlcNAc	4,0	4,5
H1Glc-H4Gal	2,5	2,7
H1GlcNAc-H3Gal	2,6	2,4
H1Gal-H4Glc	\	2,4

Table S3. Interglycosidic interproton distances (Å) for the CPS Ib pentasaccharide **3**.

	NMR	MD			
		Total average	180/-30	-60/20	-60/-50
H3Gal-H3eqNeuNAc	none	4.3	3.4	4.4	4.6
H3Gal-H3axNeuNAc	2.8	3.8	2.1	4.4	4.0
H3Gal-H8NeuNAc	Very weak	3.9	4.8	3.9	3.5
H1Gal-H3GlcNAc	2.3	2.4			
H1Gal-H2GlcNAc	3.4	4.0			
H1Glc-H4Gal	2.5	2.5			
H1GlcNAc-H3Gal	overlapped	2.5			

5. References

1. C. S. Barry, et al., *J. Am. Chem. Soc*., 2013, 135(45), 16895-16903.
2. U. Ellervik, et al.,. *J. Org. Chem*. 1998, 63: p. 9323-9338.
3. J. Dinkelaar, et al. *J. Org. Chem*, 2009. 74(11): p. 4208-4216.
4. D. Budhadev, et al.. *Carbohydr. Res.*, 2014, 394, 26-31.
5. A.V.B. Demchenko, et al., *J. Org. Chem.*, 2001. 66(8), 2547-2554.
6. W. F. Lichtenthaler, et al. *Eur. J. Org. Chem*. 2001, 3849-3869.
7. Calle, L.P., et al., *Chemistry*, 2015, 21(32), 11408-11416.
8. Cattaneo, V. et al. *Pure Appl. Chem*. 2017, 89, 855-875.
9. D. A. Case et al. *Amber 12*, 2012, University of California, San Francisco.
10. D. A. Case et al., Amber 16, 2016, University of California, San Francisco, doi:10.13140/RG.2.2.27958.70729.
11. K. N. Kirschner et al., *J. Comput. Chem*. 2008, 29 (4), 622-55.
12. GLYCAM Carbohydrate Builder web tool. Woods Group. (2005-2017) GLYCAM Web. Complex Carbohydrate Research Center, University of Georgia, Athens, GA. http://glycam.org.
13. S. Miyamoto, P. A. Kollman, *J. Comput. Chem*. 1992, 13 (8), 952-962.
14. T. Darden, D. York, L. Pedersen, *J. Chem. Phys*. 1993, 98 (12), 10089-10092.
15. U. Essmann et al., *J. Chem. Phys.* 1995, 103 (19), 8577-8593.
16. M. Crowley, et al., *J. Supercomput*. 1997, 11 (3), 255-278.
17. R. P. Laurence, H. Gerhard, Eds. American Institute of Physics Inc. 1999, 534.
18. S. Le Grand, A. W. Götz, R. C. Walker, Comp Phys Comm 2013, 184 (2), 374-380.
19. W. Götz, et al., *J. Chem. Theor. Comput*. 2012, 8 (5), 1542-1555.
20. R. Salomon-Ferrer, et al., *J. Chem. Theor. Comput*. 2013, 9 (9), 3878-3888.
